# DNA methylation in adolescents with anxiety disorder: a longitudinal study

**DOI:** 10.1038/s41598-018-32090-1

**Published:** 2018-09-14

**Authors:** Andressa Bortoluzzi, Giovanni Abrahão Salum, Eduarda Dias da Rosa, Vinicius de Saraiva Chagas, Mauro Antônio Alves Castro, Gisele Gus Manfro

**Affiliations:** 1 Anxiety Disorders Outpatient Program for Children and Adolescents, Universidade Federal do Rio Grande do Sul, UFRGS/Hospital de Clínicas de Porto Alegre, HCPA, Porto Alegre, Brazil; 2 Post Graduate Program in Neuroscience, Institute of Basic Sciences/Health, Universidade Federal do Rio Grande do Sul, UFRGS, Porto Alegre, Brazil; 3 Graduate Program in Psychiatry and Behavioral Sciences, Universidade Federal do Rio Grande do Sul, UFRGS, Porto Alegre, Brazil; 4 Basic Research and Advanced Investigations in Neurosciences, BRAIN Laboratory, Hospital de Clínicas de Porto Alegre, HCPA, Porto Alegre, Brazil; 5 Post Graduate Program in Bioinformatics, Universidade Federal do Paraná, UFPR, Curitiba, Brazil

## Abstract

Anxiety disorders (AD) typically manifest in children and adolescents and might persist into adulthood. However, there are still few data concerning epigenetic mechanisms associated with onset, persistence or remission of AD over time. We investigated a cohort of adolescents and young adults at baseline (age; 13.19 ± 2.38) and after 5 years and classified them according to the AD diagnosis and their longitudinal trajectories into 4 groups: (1) Typically Developing Comparisons (TDC; control group, n = 14); (2) Incident (AD in the second evaluation only, n = 11); (3) Persistent (AD in both evaluations, n = 14) and (4) Remittent (AD in the first evaluation only, n = 8). DNA methylation was evaluated with the Infinium HumanMethylation450 BeadChip from saliva samples collected at both evaluations. Gene set enrichment analysis was applied to consider biological pathways. We found decreased DNA methylation in TDC group while the chronic cases of AD presented hypermethylation in central nervous system development pathways. Moreover, we showed that this persistent group also presented hypermethylation while the other three groups were associated with hypomethylation in nervous system development pathway. Incidence and remission groups were associated with increased and decreased methylation in neuron development pathways, respectively. Larger studies are likely to detect specific genes relevant to AD.

## Introduction

Anxiety disorders are a group of syndromes characterized by maladaptive responses to threats, that develop as a result of the interplay between biological factors, psychological mechanisms and environmental influences^1–4^. These disorders usually have their onset during childhood and adolescence and frequently persist into adulthood^5–7^. Understanding the genetically mediated factors that lead to persistence (or remission) of anxiety over time might reveal new ways of preventing and treating those disorders.

Epigenetic mechanisms, mainly DNA methylation, are the result of the interface between genes and environmental factors^4,8,9^. Some studies have suggested that these mechanisms might be able to assure enduring environmental influences on the body, thus mediating the increased susceptibility for psychiatric disorders^10,11^. Studies in rodents described that the negative impact of early stress on behavioral responses as well as on brain changes may influence the regulation of DNA methylation across generations^12,13^. Furthermore, a study in humans found that changes in the offspring’s perceptions of maternal bonding were also related to DNA hypermethylation in cell signaling process over time^14^. Therefore, these findings are consistent with the idea that epigenetic mechanisms may be associated to the behavioral and emotional response to environmental factors in long term.

Several candidate genes (e.g: *BDNF; FKBP5; SLC6A4; AVP; NR3C1; CRH; COMT; MAOA; OXTR* and *APOE*) to psychiatric disorders have been quoted in epigenetic studies using locus-specific assays^11,15–18^. In general, these studies reported that the effects of adverse environment might generate epigenetic modifications that, in turn, can alter physiological processes important to the development of mental disorders. However, no hub genes have consistently been associated to diagnosis or the course of anxiety disorders. Given the current state of knowledge on the genetics of anxiety disorders, unbiased hypothesis-free methodology might be more fruitful for investigating the DNA methylation fingerprints related to the course of these disorders^19–21^. To our knowledge there is a paucity of studies involving genome-wide DNA methylation and anxiety in humans^22–25^ and in animals^26^ studies.

This is an unbiased hypothesis-free study with no predictions with respect to specific genes aiming to investigate which biological pathways are associated with the course of anxiety disorders based on gene ontology. This analysis allows for the investigation of sets of genes instead of looking for isolated genes^27^. Our aim is to explore biological pathways that may confer an epigenetic signature according to favorable and unfavorable trajectories of anxiety disorders.

## Methods

### Sample selection and Participants

This is a longitudinal 5-year follow-up study that involves a subsample of adolescents and young adults from a community cohort enriched for participants with anxiety disorders originated in 2008 that were re-evaluated 5 years later, in 2013.

In 2008, 240 non-medicated adolescents from a total of 2.457 students that answered a screening scale for anxiety disorders (Screen for Child and Anxiety Related Emotional Disorders - SCARED)^28^ in their schools underwent an extensive psychiatric diagnostic assessment (Schedule for Affective Disorder and Schizophrenia for School-Age Children-Present and Lifetime Version - K-SADS-PL)^29,30^ and evaluation in the anxiety outpatient research program at Hospital de Clínicas de Porto Alegre. Exclusion criteria included: (1) a significant organic illness; (2) a history of bipolar disorder, pervasive developmental disorder, or any psychotic disorder; (3) a history of alcohol or drug dependence or abuse; or (4) a clinical suspicion of intellectual disability. Of these selected individuals, six were excluded due to intellectual disability remaining a total of 234 adolescents: 134 with anxiety disorders and 100 not anxious individuals classified as controls to anxiety disorders. Details about initial selection procedures are described elsewhere^31^.

The initial 2008 sample (n = 234) was contacted again in 2013 and of these, 76 adolescents agreed to participate in the 5-year follow-up survey. Adolescents were re-evaluated throughout the K-SADS-PL or the Mini International Neuropsychiatric Interview (MINI)^32^ depending on their ages. After the second evaluation, 47 subjects (Table 1) were selected to undergo genome-wide DNA methylation analysis due to logistical strategies and financial limitations. These anxious and non-anxious individuals were categorized into four groups of subjects according to their anxiety disorder trajectories from 2008 to 2013, carefully paired by age and gender:

1. Typically Developing Comparisons (TDC; n = 14; mean age = 17.96; SD = 2.38; 57.14% females). This group is considered control to anxiety disorders because they were not diagnosed with anxiety disorder in both psychiatric evaluations (2008 and 2013), according to the instruments used (described in section 2.2).
2. Incident (n = 11; mean age = 17.27; SD = 1.95; 45.45% females). This group is composed by those who were controls for anxiety disorders in 2008 but were considered cases for any anxiety disorder in 2013, which means that these participants were not previously anxious (2008), but have the onset of anxiety disorder in the second evaluation (2013).
3. Persistent (n = 14; mean age = 18.32; SD = 2.34; 57.14% females). This group is comprised by participants who had anxiety disorder diagnoses in both evaluations, 2008 and 2013, being, therefore, considered chronic cases for anxiety disorder.
4. Remittent (n = 8; mean age = 19.73; SD = 2.71; 50% females). This group is composed by youths that had anxiety disorder in 2008, but not in 2013. They remitted from the disorder.

**Table 1:** Descriptive data about adolescents evaluated in 2008 and 2013.

Variables	2008			2013		
	Anxiety Individuals (n = 22)	Non-Anxious (n = 25)	P-value	Anxiety Individuals (n = 25)	Non-Anxious (n = 22)	P-value
Gender (female)	12 (54.5%)	13 (52%)	0.861^a^	13 (52%)	12 (54.5%)	0.861^a^
Age (mean ± SD)	13.79 ± 2.5	12.66 ± 2.18	0.506^b^	17.86 ± 2.19	18.56 ± 2.57	0.273^b^
**Ethnicity**						
Caucasians	13 (59.1%)	14 (58.3%)	0.481^c^	18 (72%)	09 (40.9%)	0.057^c^
Brazilian Africans	04 (18.2%)	02 (8.3%)		01 (4%)	05 (22.7%)	
Other	05 (22.7%)	07 (29.2%)		06 (24%)	06 (27.3%)	
**Psychiatric diagnosis**						
Generalized Anxiety Disorder	16 (72.7%)	—		17 (68%)	—	
Social Anxiety Disorder	08 (36.4%)	—		08 (32%)	—	
Separation Anxiety Disorder	09 (40.9%)	—		03 (12%)	—	
Agoraphobia	—	—		01 (4%)	—	
Panic Disorder	03 (13.6%)	—		01 (4%)	—	
Specific Phobia	07 (31.8%)	—		03 (12%)	—	

^a^Chi-squared. Differences between anxiety individuals and non-anxious considering gender.

^b^Test t student. Differences between anxiety individual and non-anxious considering age.

^c^Chi-squared. Differences between anxiety individuals and non-anxious considering ethnicity. Statistical significance: P < 0.05.

In 2008, 07 (31.82%) had only one AD, 11 (50%) had two AD and 04 (18.18%) had three or four AD. In 2013, 16 (64%) had only one AD, 06 (24%) had two AD and 03 (12%) had three AD. The ethnicity was determined by self-report, according to individual skin color. Three people do not provide ethnicity data.

This study was approved by the Research Ethics Committee of Hospital de Clínicas de Porto Alegre (HCPA; protocol number 12-0254) and all participants and the caregivers who were participant’s legal guardians provided written informed consent in order to participate in the study. All methods were performed in accordance with the relevant guidelines and regulations.

### Anxiety Disorder Diagnosis

The psychiatric diagnosis was assessed in 2008 using the Brazilian version of K-SADS-PL^29^ and in 2013 with the same instrument or with the MINI^32^ depending on the age of the individual (K-SADS for subjects with age lower than 18 years old and MINI for those with 19 years old or higher).

The K-SADS-PL is a semi-structured diagnostic interview that ascertains both lifetime and current psychiatric diagnostic status in children and adolescents based on Diagnostic and Statistical Manual of Mental Disorders, 4^th^ (DSM-IV) criteria^30^. The Brazilian version of this instrument presents adequate psychometric properties (reliability and validity) and can be used in both clinical practice and research in order to evaluate children and adolescents mental health^29^. Our Inter-rater reliability in this sample was 0.932 (kappa value) for anxiety diagnoses^33^.

The MINI is a structured clinical diagnostic psychiatric interview that evaluates the main diagnoses according DSM-IV criteria in individuals above 18 years old^32^. This instrument also has good psychometric properties with kappa coefficients ranging between 0.65 and 0.85^34^.

### Array-Based Genome-Wide DNA Methylation Assays

DNA was extracted from saliva sample, collected in both evaluations, 2008 and 2013, using Oragene^™^ kits (DNA Genotek, Ottawa, Ontario, Canada). We treated the extracted genomic DNA (500 ng) with sodium bisulphite using the EZ-96 DNA Methylation-Gold^™^ Kit (Zymo Research, Orange, CA, USA) according to the manufacturer’s protocol. Bisulfite-converted DNA was subsequently assessed for DNA methylation status at 485.577 CpG loci with the Infinium HumanMethylation450 (IHM450) BeadChip^35,36^. The IHM450 BeadChip covers 99% of Ref Seq genes regions and involves targeted gene regions with sites in the promoter region, 3′and 5′untranslated regions (3′UTR and 5′UTR), first exon, and gene body in order to explore the genome-wide DNA methylation. This multiple-site approach is extended to CpG islands/CpG island regions for which 96% of islands were covered overall, with multiple sites within islands and island shores, as well as those regions flanking island shores (island shelves)^35,37,38^.

### Pre-Processing of Raw Data of IHM450 BeadChip

Raw data was evaluated according to the quality control of samples and probes; background correction; normalization; type 1 and 2 probe scaling and batch/plate/chip/confounder adjustment^36^ following the processing workflows described in the *R* packages *lumi*^39^ *methyAnalysis*^40^ and *sva*^41^. *A*reas with single nucleotide polymorphisms and sexual chromosomes were removed. After this cleaning process, we had 382.264 probes of IHM450 BeadChip. All data including DNA methylation values/subject, and methylated vs. unmethylated probes are deposited in GEO and are accessible via the GEO identifier GSE78975.

### Differential Methylation in Signatures

We used limma R package to evaluate differential methylation signatures comparing the longitudinal and the case-control contrasts^42^. In the longitudinal study, we compared times *t1* and *t2* to (A) controls, (B) persistent (cases), (C) incident and (D) remittent groups resulting in four contrasts: A_t2_ -A_t1_, B_t2_ -B_t1_, C_t2_ -C_t1_ and D_t2_ -D_t1_. In the case-control study, we compared two contrasts, (A) controls and (B) persistent (cases) groups at each time: B_*t1*_ - A_*t1*_ and B_*t2*_ - A_*t2*_. Therefore, each probe has been evaluated by 6 different contrasts. Results from each contrast, for each probe, are summarized in log2-fold change values. The probe with the highest variance across samples was selected to represent a gene when we had multiples probes mapped to the promoter region. A “differential methylation signature” represents the differential methylation of a given contrast mapped to 19.556 unique genes. The differential methylation signatures have been ranked by log2-fold change values for the enrichment analysis described bellow.

### Gene Set Enrichment and statistical analysis

Package HTSanalyzeR was used to perform Gene Set Collection Analysis (GSCA) on the differential methylation signatures^43^. We used the Sub catalog C5 of Molecular Signature Database (MSigBD) composed by Gene Ontology (GO; http://geneontology.org/) that describes gene products in terms of their associated biological pathways in a species-independent manner^27,44^.

Gene set enrichment analysis (GSEA) was applied to GO. GSEA is a method that groups genes that share common biological functions or regulation. It has three keys elements: calculation of an Enrichment Score (ES); estimation of significance level of ES and adjustment for multiple hypothesis testing^45^.

We used the high scoring gene sets that were grouped according to the basis of leading-edge subsets of shared genes. Leading-edge subset is used to extract the core members of high scoring gene sets that contribute to the enrichment signal and thus can reveal a biologically important subset within a gene set^45^. The enrichment analysis was performed with differential methylation signatures mapped to 19.556 genes. The GSEA scores reflected the difference in DNA methylation pattern between two groups (contrasts) and did not aim to identify hub genes. Moreover, it avoids any conclusion at a single gene level. One advantage of this approach is that it does not rely on arbitrary statistical thresholds to assign significance for individual genes, but it uses all genes as a differential methylation signature.

We performed two sets of analysis. *First*, we compared differences in DNA methylation patterns from GSEA analysis considering time (baseline versus 5-year follow-up) in each of the 4 groups (TDC, incident, persistent and remittent). *Second*, we performed case-control analysis comparing anxious *vs*. non-anxious cases across both time points. We analyzed 825 biological pathways. We considered 30 as the minimum gene set size in the gene set enrichment analysis^43^. The significant gene set cutoff p-value (adjusted) was set to <0.001. To estimate the p value we considered 1000 permutations in GSEA and P-values were corrected by BH method^46^.

## Results

### Gene Signatures in longitudinal approach: “2013–2008 contrast”

The sample re-evaluated in 2013 can be considered representative of the whole sample from 2008. There were also no differences regarding age, sex and ethnicity in the 4 groups with the different anxiety trajectories (TDC, incident, remittent and persistent). Furthermore, all analyses were controlled for age and sex. These analyses are available in the supplemental material.

**Figure 1:**
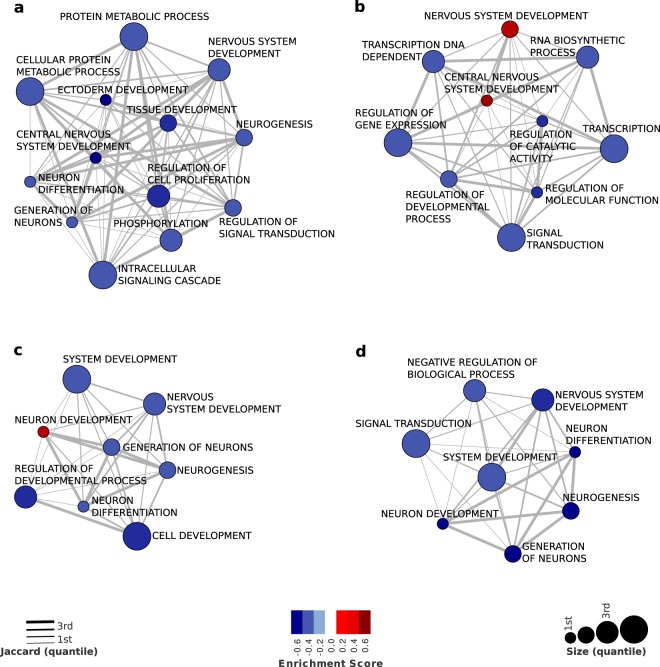
Association maps between gene sets enriched with DNA methylation signatures from the longitudinal contrasts. The blue-red scale shows the DNA methylation enrichment scores computed by GSEA analysis for (**a**) controls, (**b**) persistent (cases), (**c**) incident and (**d**) remittent longitudinal groups. Node size represents the number of genes annotated in a given Gene Ontology (GO) biological pathways, and edge width represents the overlap between GO terms as measured by the Jaccard coefficient (JC).

From the 825 biological pathways analyzed, we observed 50 differentially methylated pathways in TDC group, 13 in the incident group, 27 in the persistent group and 11 in the remittent group (Table 2). Overall we can see a pattern of DNA hypomethylation from several cellular processes (e.g., intracellular signaling cascade, regulation of signal transduction and regulation of metabolic process) in the TDC group that were not seen in the other groups. On the other hand, we found patterns of DNA hypomethylation in other biological pathways in the other three groups. These results are depicted in Fig. 1.

**Table 2:** GSEA of Biological pathways in groups defined by trajectories of anxiety disorders.

	Significant enrichment scores based on GSEA			
	TDC	Persistent	Incident	Remittent
***Nervous System processes***				
Neurogenesis	−0.3781	NS	−0.3549	−0.5699
Neuron Differentiation	−0.3894	NS	−0.3735	−0.6113
Generation of Neurons	−0.3638	NS	−0.3689	−0.5911
Nervous System Development	−0.3807	0.3156	−0.3859	−0.4942
Central Nervous System Development	−0.5184	0.4273	NS	NS
Neuron development	NS	NS	0.3988	−0.5688
Neurite development	NS	NS	−0.3712	NS
***Signalization processes***				
Signal transduction	−0.3332	−0.3146	NS	−0.355
Cell surface receptor linked signal transduction GO:0007166	−0.3373	NS	NS	NS
Intracellular signaling cascade	−0.3335	NS	NS	NS
***Regulation processes***				
Regulation of molecular function	NS	−0.4451	NS	NS
Regulation of catalytic activity	NS	−0.466	NS	NS
Negative regulation of catalytic activity	NS	−0.4222	NS	NS
Regulation of signal transduction	−0.3928	NS	NS	NS
Negative regulation of cellular process	−0.2951	NS	NS	−0.34
Regulation of developmental process	0.2634	−0.373	−0.4652	NS
Regulation of cellular metabolic process	−0.3065	−0.345	NS	NS
Regulation of gene expression	−0.2935	−0.3588	NS	NS
Regulation of transcription DNA dependent	−0.2624	−0.36	NS	NS
Positive regulation of biological process	−0.2886	NS	NS	NS
Regulation of metabolic process	−0.3041	NS	NS	NS
Regulation of nucleobase nucleoside nucleotide and nucleic acid metabolic process	−0.2911	−0.3436	NS	NS
Regulation of metabolic process	−0.3041	−0.3465	NS	NS
Regulation of RNA metabolic process	−0.2658	−0.3497		NS
Negative regulation of developmental process	0.305	NS	NS	NS
Positive regulation of cellular process	−0.2929	NS	NS	NS
Negative regulation of biological process	−0.2887	NS	NS	−0.3493
Regulation of transcription	−0.2844	−0.3604	NS	NS
Regulation of cell proliferation	−0.402	NS	NS	NS
Positive regulation of multicellular organismal process	0.3788	NS	NS	NS
Regulation of multicellular organismal process	−0.2907	NS	NS	NS
***Development processes***				
System development	NS	−0.2963	−0.387	−0.3826
Muscle development	−0.317	NS	NS	NS
Cell development	−0.2801	−0.3328	−0.4431	NS
Tissue development	−0.4514	NS	NS	NS
Multicellular organismal development	−0.4169	−0.287	−0.3736	NS
Anatomical structure development	−0.315	−0.2841	−0.3904	−0.3719
Organ development	−0.3261	NS	−0.3861	NS
Ectoderm development	−0.5677	NS	NS	NS
***Processes involved with protein and protein synthesis***				
Transcription	−0.2622	−0.381	NS	NS
Transcription DNA dependent	−0.2476	−0.3896	NS	NS
Post translational protein modification	−0.3913	−0.3351	NS	NS
Cellular protein metabolic process	−0.3728	NS	NS	NS
Protein kinase cascade	−0.2912	NS	NS	NS
Protein metabolic process	−0.3696	NS	NS	NS
Protein modification process	−0.3636	−0.3603	NS	NS
Protein aminoacid phosphorylation	−0.3672	NS	NS	NS
RNA biosynthetic process	−0.2474	−0.3907	NS	NS
***Other processes***			NS	NS
Biopolymer metabolic process	−0.318	−0.3353	NS	NS
Biopolymer modification	−0.3622	−0.2878	NS	NS
Anatomical structure morphogenesis	−0.282	−0.2791	−0.3262	−0.3735
Defense response	0.3405	NS	NS	NS
Cell proliferation GO:0008283	−0.275	NS	NS	NS
Cellular macromolecule metabolic process	−0.3712	NS	NS	NS
Phosphorylation	−0.3768	NS	NS	NS
Leukocyte activation	0.3234	NS	NS	NS
Locomotory behavior	NS	0.1618	NS	NS

Note: GSEA, Gene set enrichment analysis; TDC, Typically Developing Controls; NS, No significant (p > 0.001). All biological pathways significant at false discovery rate (FDR) correction. Signal negative represents DNA hypomethylated biological pathways.

Of note, several biological pathways related to nervous systems were differently methylated according to the different trajectory of anxiety disorders. There are two main findings that should be considered worth noting. *First*, we could see a pattern of DNA hypomethylation in biological pathways associated with neurogenesis, neuron differentiation and generation of neurons for TDC, incident and remittent groups that were not significant in the persistent group. In addition, the same pattern of DNA hypomethylation could be seen in nervous system development process in TDC, incident and remittent groups, whereas a pattern of DNA hypermethylation was observed in the persistent group. Furthermore, DNA hypermethylation was also observed in biological pathways related to the development of the central nervous system in individuals with chronic anxiety whereas in the TDC group we found DNA hypomethylation in this pathway (Table 2). *Second*, neuron development pathway was hypermethylated in the incident group and hypomethylated in the remittent group, but no significant results were found for TDC and persistent groups (Fig. 1 and Table 2). The genes that comprised each biological pathway in all four groups can be observed in supplementary information (Supplementary Appendix 1).

### Gene Signatures in cross-sectional approach: “Case - Control contrasts”

**Figure 2:**
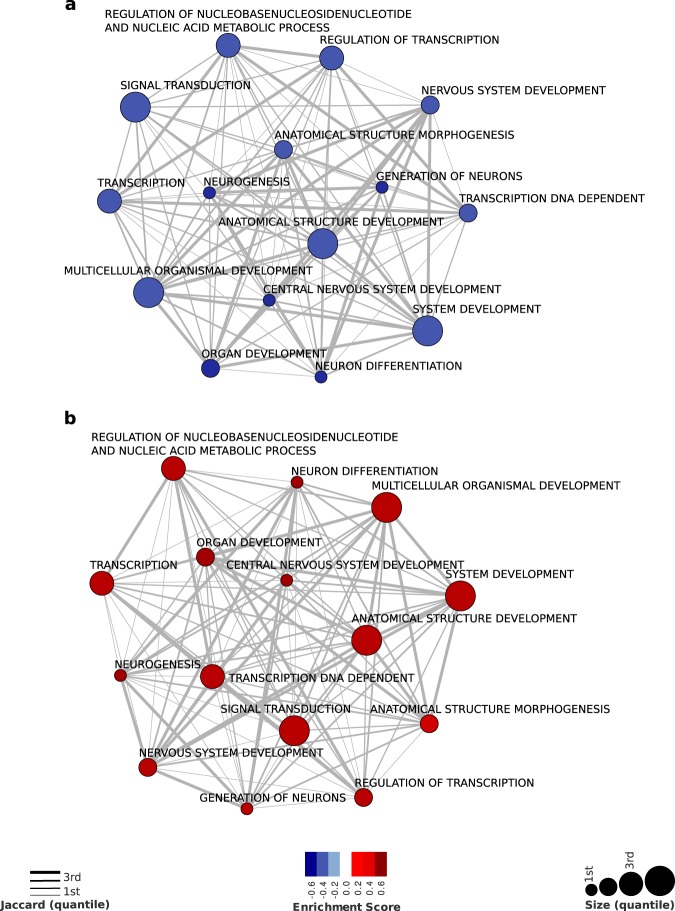
Association maps between gene sets enriched with DNA methylation signatures from the cross-sectional contrasts. The blue-red scale shows the DNA methylation enrichment scores computed by GSEA analysis for case-control groups at the 1^st^ (**a**) and 2^nd^ (**b**) year of evaluations. Node size represents the number of genes annotated in a given Gene Ontology (GO) biological pathways, and edge width represents the overlap between GO terms as measured by the Jaccard coefficient (JC).

Considering the cross-sectional approach in which the contrast is between cases as compared to control groups, we found 54 biological pathways significant at false discovery rate (FDR) correction in 2008 and 21 biological pathways in 2013 (Table 3). We observed DNA methylation in opposite directions in several biological pathways comparing both evaluations (2008 and 2013), as can be seen in Fig. 2.

**Table 3:** GSEA of Biological pathways in groups defined by “case-control” contrasts.

	Significant enrichment scores based on GSEA	
	2008	2013
***Nervous System processes***		
Nervous system development	−0.3724	0.3875
Central nervous system development	−0.4466	0.4619
Generation of neurons	−0.4522	0.3904
Neuron differentiation	−0.4678	0.4064
Neurogenesis	−0.4704	0.426
Brain development	−0.5241	NS
Neurite development	NS	0.4524
Neuron development	NS	0.4475
***Regulation processes***		
Negative regulation of nucleobase nucleoside nucleotide and nucleic acid metabolic process	−0.3366	NS
Negative regulation of cellular metabolic process	−0.3661	NS
Positive regulation of nucleobase nucleoside nucleotide and nucleic acid metabolic process	−0.5359	NS
Positive regulation of transcription DNA dependent	−0.5537	NS
Positive regulation of RNA metabolic process	−0.5589	NS
Negative regulation of transcription DNA dependent	−0.3836	NS
Negative regulation of cellular process	−0.3618	NS
Regulation of developmental process	−0.3511	NS
Regulation of transcription from RNA polymerase II promoter	−0.3982	NS
Regulation of cellular metabolic process	−0.36	NS
Regulation of gene expression.	−0.3292	NS
Positive regulation of developmental process	−0.3546	NS
Positive regulation of metabolic process	−0.5421	NS
Regulation of transcription DNA dependent	−0.4831	NS
Negative regulation of transcription	−0.3385	NS
Negative regulation of RNA metabolic process	−0.3754	NS
Positive regulation of cellular metabolic process	−0.4082	NS
Positive regulation of biological process	−0.4049	NS
Negative regulation of metabolic process	−0.3613	NS
Regulation of metabolic process	−0.3607	NS
Regulation of RNA metabolic process	−0.3869	NS
Positive regulation of cellular process	−0.4088	NS
Regulation of nucleobase nucleoside nucleotide and nucleic acid metabolic process	−0.3319	0.3038
Negative regulation of biological process	−0.3715	NS
Regulation of transcription	−0.3252	0.3122
Positive regulation of transcription	−0.5666	NS
***Development processes***		NS
System development	−0.3819	0.3671
Cell development	−0.3795	NS
Multicellular organismal development	−0.3767	0.368
Anatomical structure development	−0.3944	0.3279
Organ development	−0.42	0.4024
Tissue development	NS	0.4646
***Processes involved with protein and protein synthesis***		
RNA metabolic process	−0.373	NS
Nucleobase nucleoside nucleotide and nucleic acid metabolic process	−0.3783	NS
Transcription DNA dependente	−0.3912	0.3193
RNA biosynthetic process	−0.3928	NS
Post translational protein modification	−0.417	NS
Transcription	−0.391	0.3193
Protein modification process	−0.4134	NS
Protein aminoacid phosphorylation	−0.3827	NS
Transcription from RNA polymerase II promoter	−0.4092	NS
Protein amino acid dephosphorylation	−0.4928	NS
Protein metabolic process	NS	0.3637
***Other processes***		
Cell migration	NS	0.4125
Signal transduction	−0.3471	0.317
Cell surface receptor linked signal transduction GO: 0007166	NS	0.2819
Anatomical structure morphogenesis	−0.3608	0.2994
Locomotory behavior	−0.1731	NS
Biopolymer metabolic process	−0.3539	NS
Biopolymer modification	−0.4122	NS
Dephosphorylation	−0.5187	NS
Phosphorylation	−0.3469	NS

Note: GSEA, Gene set enrichment analysis; NS, No significant (p > 0.001). All biological pathways significant at false discovery rate (FDR) correction. Signal negative represents DNA hypomethylated biological pathways.

When we considered the time of the evaluation, we found that many biological pathways involved with nervous system (e.g, nervous system development, central nervous system development and generation of neurons) were DNA hypomethylated in 2008 and hypermethylated in 2013. It is noteworthy that other biological pathways (e.g, regulation of development process, regulation of gene expression and regulation of metabolic process) showed a pattern of DNA hypomethylation in 2008, which did not reach significance in 2013 (Fig. 2 and Table 3). The genes that compose each biological pathway in control and case groups can be observed in supplementary information (Supplementary Appendix 1).

## Discussion

We were able to show different DNA methylation patterns in many biological pathways depending on the longitudinal trajectory of anxiety disorders. Furthermore, we explore cross-sectional data in two important periods of life. Our results demonstrated that anxiety disorder trajectories in adolescence might be reflected on different DNA methylation patterns evaluated from saliva samples.

Even though some biological pathways showed DNA hypomethylation patterns either in the TDC (controls) or in the persistent (cases) groups (e.g, signal transduction, regulation of cellular metabolic process and regulation of metabolic process), when we considered only nervous system development and central nervous system development processes, we found opposite directions of DNA methylation (hypomethylation in controls as opposed to hypermethylation in cases) within the longitudinal contrast. Moreover, we were able to observe that the neuron development process was associated to DNA hypermethylation in the incident group while this same biological pathway was hypomethylated in the remittent group, but without significance in TDC or persistent groups. Up to now, we have no way to infer the consequence of DNA methylation in transcriptional levels but maybe the severity of anxiety symptoms over time could act silencing genes involved within nervous system.

Surprisingly, when we investigated cases and controls to anxiety disorders in cross-sectional approach, we found DNA methylation patterns with opposite directions with the “cases - controls” contrast. Moreover, the beginning of adolescence was characterized by DNA hypomethylation patterns whereas young adults presented DNA hypermethylation patterns. Although the majority of biological pathways were observed at both evaluations, some of them could be observed only in one or in another period. These results suggest that the time factor could be more important than anxiety diagnosis per se in changing epigenetic development biological patterns.

There is no consensus in the literature regarding the increase or decrease of DNA methylation in a longitudinal lifetime course. There are studies in healthy populations that suggested DNA hypermethylation patterns as people get older^47,48^, as well as to DNA hypomethylation over the time^49^. Talens *et al*. (2012) suggested small or even no differences in mean DNA methylation between the young versus the old age healthy twins, while increases in variation were generally more substantial in older individuals. It is suggested that epigenetic changes might either accumulate with age being generally nondirectional or they are the outcome of many smaller changes with opposite directions^50^.

According to our knowledge this is the first study that addresses the direct association between anxiety disorders and genome-wide DNA methylation, with a longitudinal approach in adolescents. There are very few data evaluating anxiety (trait, state or disorder) and genome-wide DNA methylation in humans^21–24,51^. Shimada *et al*.^23^ studied genome-wide DNA methylation in patients with Panic Disorder (PD) and healthy control subjects. They depicted forty CpG sites significant associated with PD at 5% FDR correction, but with small different rates of the DNA methylation. A study that compared medication-free PD patients with healthy controls^25^ suggested a possible sex-specific methylation process (hypermethylation in female patients) in the HECA gene, but no global changes in DNA methylation. Schartner *et al*.^52^ suggested that patients with PD presented significantly DNA hypomethylation in promotor region of CRHR1 gene when compared with a sample of healthy controls. Unfortunately, we cannot add information on these specific genes or any other gene because our data is limited to genome-wide DNA methylation.

Recent studies that explored the genome-wide DNA methylation in order to understand the etiology of psychiatric disorders did not consider anxiety disorders^53–58^. Most studies evaluated the contribution of environmental adversity in DNA methylation differences associated to depression^53^; post-traumatic stress disorder^16,59–62^, schizophrenia^63^; borderline personality disorder, attention deficit hyperactivity disorder and bipolar disorders^64,65^. Also, some cross-sectional studies have investigated DNA methylation in specific genes (e,g; BDNF and MB-COMT) in individuals with different psychiatric disorders^17,18,66^ and their data suggested higher DNA methylation in individuals with internalizing disorders and decreased DNA methylation in patients diagnosed with schizophrenia and bipolar in comparison to subjects without psychiatric disorders.

The few studies that explored a longitudinal approach involving the genome-wide DNA methylation in early development suggested that adversities in childhood are associated to differences in DNA methylation and risk to mental health^67,68^. In parallel to the influence of early adversities, DNA methylation changes may be observed as a result of therapeutic treatment using the same concept of environmental interference in DNA regulation^69^. An interesting study compared changes in *SLC6A4* methylation after Cognitive Behavior Treatment (CBT) in anxious children, considering those that remitted as compared to those that did not remit from their primary anxiety diagnoses. While responders showed an increase percentage of DNA methylation in *SLC6A4*, non-responders had decreased *SLC6A4* DNA methylation percentage^70^. These results demonstrated the complexity of the epigenetic mechanisms underlying anxiety as well as psychiatric disorders in general.

Our study has some limitations, such as the small sample size, which meant that we did not have sufficient power to explore individual genes associated with anxiety. In addition, we did not have information regarding the anti-anxiety medication use over time, or data on childhood trauma. We exclude participants with drug abuse or dependence, but we did not evaluate eventual smoking. The use of drugs or smoking can influence genome-wide DNA methylation^71–73^ and the presence of trauma or early life stressors is known to increase the risk to psychopathology during lifetime and are able to reprogram the epigenetic mechanisms^74^. Moreover, we did not evaluate the effects of genotype on the DNA methylation differences because we did not consider specific genes. In addition, even though we know that saliva samples contain a heterogeneous mixture of different cell-types: epithelial cells and leukocytes^75^, we were not able to estimate the proportion of epithelial cells in comparison to leukocytes in our sample. Although this issue could be a limitation of our study, the study of Smith *et al*.^75^ verified that comparisons of DNA methylation between saliva and four brain regions (cerebellum, frontal cortex, entorhinal cortex, and superior temporal gyrus) seems to be more similar than comparisons between blood and these same brain regions validating our measure. There are also some limitations in relation to gene-set analyses. Gene-gene correlations and the contributions of some genes to multiple Gene Ontology terms mean that some biological pathways are spuriously represented, when the association with the phenotype of interest could be explained by the overlap between genes within the biological pathways.

However, our study has important strengths that should be acknowledged. We used DNA from buccal cells present in saliva sample in our genome-wide DNA methylation study that were collected in two different evaluations in long term. This approach is considered a gold pattern design to evaluate epigenetics mechanisms. The DNA extracted from saliva sample is more representative than blood DNA due its consistence with typical methylation patterns in the brain regions once it is originated from the same ectodermal layer during development of brain tissue^57,75,76^. Although there are tissue-specific epigenetic variation across brain and blood^57,77^, the use of peripheral tissue is the only feasible method concerning biological investigation of the central nervous system^76^. We also used the GSEA method, which is the most appropriate way to generate hypotheses in a large-scale experiment in order to identifying biological pathways, avoiding the challenge of indicating single genes. In this way, we used our rich dataset to study the genome-wide DNA methylation in a more exploratory approach considering the different longitudinal trajectories of anxiety disorders. Our data may help to further focus on specific sets of genes in order to delineate more a priori hypothesis studies^45^.

## Conclusions

We used a rich dataset to study the genome-wide DNA methylation in a more exploratory approach considering the presence of either hypo or hyper DNA methylation in the distinct biological pathways as well as the participants’age along the different longitudinal trajectories of anxiety disorders. In the longitudinal analysis we observed that the incident and remittent youth cases of anxiety presented biological pathways associated to DNA hypomethylation patterns. A pattern of DNA hypermethylation within the nervous system development was observed in the persistent group over the five years. On the other hand, individuals presented mainly DNA hypomethylation when they were younger, and DNA hypermethylation patterns with increased age. Further studies are needed to deepen the understanding of the biological pathways involved with anxiety disorders, their longitudinal course and epigenetic changes. Our data may further help looking for more specific genes associated to this complex disorder in the future.

## Electronic supplementary material

Dataset 1

## References

[1] Salum GA, De Sousa DA, do Rosário MC, Pine DS, Manfro GG (2013). Pediatric anxiety disorders: From neuroscience to evidence-based clinical practice. Rev. Bras. Psiquiatr..

[2] Kendler KS, Myers JM, Maes HH, Keyes CLM (2011). The relationship between the genetic and environmental influences on common internalizing psychiatric disorders and mental well-being. Behav. Genet..

[3] Ask, H., Waaktaar, T., Seglem, K. B. & Torgersen, S. Common Etiological Sources of Anxiety, Depression, and Somatic Complaints in Adolescents: A Multiple Rater twin Study. *J*. *Abnorm*. *Child Psychol*. **44** (2015). 10.1007/s10802-015-9977-y 25619928

[4] Schiele MA, Domschke K (2017). Epigenetics at the crossroads between genes, environment and resilience in anxiety disorders. Genes, Brain Behav..

[5] Simon E, Bögels SM (2009). Screening for anxiety disorders in children. Eur. Child Adolesc. Psychiatry.

[6] Costello EJ, Egger HL, Angold A (2005). The developmental epidemiology of anxiety disorders: Phenomenology, prevalence, and comorbidity. Child Adolesc. Psychiatr. Clin. N. Am..

[7] Creswell C, Waite P, Cooper PJ (2014). Assessment and management of anxiety disorders in children and adolescents. Arch. Dis. Child..

[8] Bernstein BE, Meissner A, Lander ES (2007). The Mammalian Epigenome. Cell.

[9] Boyce WT, Kobor MS (2015). Development and the epigenome: the ‘synapse’ of gene-environment interplay. Dev. Sci..

[10] Archer T, Oscar-Berman M, Blum K, Gold M, Blum K (2013). Epigenetic Modulation of Mood Disorders. J. Genet. Syndr. Gene Ther..

[11] Hing B, Gardner C, Potash JB (2014). Effects of negative stressors on DNA methylation in the brain: Implications for mood and anxiety disorders. Am. J. Med. Genet. Part B Neuropsychiatr. Genet..

[12] Franklin TB (2010). Epigenetic Transmission of the Impact of Early Stress Across Generations. Biol. Psychiatry.

[13] Holmes A (2005). Early life genetic, epigenetic and environmental factors shaping emotionality in rodents. Neurosci. Biobehav. Rev..

[14] Naumova OY (2016). Epigenetic Patterns Modulate the Connection Between Developmental Dynamics of Parenting and Offspring Psychosocial Adjustment. Child Dev..

[15] Klengel T, Pape J, Binder EB, Mehta D (2014). The role of DNA methylation in stress-related psychiatric disorders. Neuropharmacology.

[16] Koenen KC (2011). SLC6A4 methylation modifies the effect of the number of traumatic events on risk for posttraumatic stress disorder. Depress. Anxiety.

[17] Nohesara S (2011). DNA hypomethylation of MB-COMT promoter in the DNA derived from saliva in schizophrenia and bipolar disorder. J. Psychiatr. Res..

[18] Chagnon YC, Potvin O, Hudon C, Préville M (2015). DNA methylation and single nucleotide variants in the brain-derived neurotrophic factor (BDNF) and oxytocin receptor (OXTR) genes are associated with anxiety/depression in older women. Front. Genet..

[19] Sharma S, Powers A, Bradley B, Ressler KJ (2016). Gene × Environment Determinants of Stress- and Anxiety-Related Disorders. Annu. Rev. Psychol..

[20] Otowa, T. *et al*. Meta-analysis of genome-wide association studies of anxiety disorders. *Mol*. *Psychiatry* 1–9, 10.1038/mp.2015.197 (2016). 10.1038/mp.2016.11 26857599

[21] Coleman JRI (2016). Genome-wide association study of response to cognitive-behavioural therapy in children with anxiety disorders. Br. J. Psychiatry.

[22] Chen L (2015). Brain-derived neurotrophic factor (BDNF) Val66Met polymorphism influences the association of the methylome with maternal anxiety and neonatal brain volumes. Dev. Psychopathol..

[23] Shimada-sugimoto M (2017). Epigenome-wide association study of DNA methylation in panic disorder. Clin. Epigenetics.

[24] Emeny RT (2018). Anxiety Associated Increased CpG Methylation in the Promoter of Asb1: A Translational Approach Evidenced by Epidemiological and Clinical Studies and a Murine Model. Neuropsychopharmacology.

[25] Iurato S (2017). “DNA Methylation signatures in panic disorder”. Transl. Psychiatry.

[26] Alisch RS (2014). Differentially Methylated Plasticity Genes in the Amygdala of Young Primates Are Linked to Anxious Temperament, an at Risk Phenotype for Anxiety and Depressive Disorders. J. Neurosci..

[27] Ashburner M (2000). The Gene Ontology Consortium. Gene ontology: tool for the unification of biology. Nat. Genet..

[28] Isolan L, Salum GA, Osowski AT, Amaro E, Manfro GG (2011). Psychometric properties of the Screen for Child Anxiety Related Emotional Disorders (SCARED) in Brazilian children and adolescents. J. Anxiety Disord..

[29] Brasil HH, Bordin IA (2010). Convergent validity of K-SADS-PL by comparison with CBCL in a Portuguese speaking outpatient population. BMC Psychiatry.

[30] Kaufman J (1997). Schedule for Affective Disorders and Schizophrenia for School- Aged Children - Present and Lifetime Version (K-SADS-PL): Initial Reliability and Validity Data. J. Am. Acad. Child Adolesc. Psychiatry.

[31] Salum GA (2011). The multidimensional evaluation and treatment of anxiety in children and adolescents: rationale, design, methods and preliminary findings Avaliação multidimensional e tratamento da ansiedade em crianças e adolescentes: marco teórico, desenho, método. Rev. Bras. Psiquiatr..

[32] Amorim P (2000). Mini International Neuropsychiatric Interview (MINI): validação de entrevista breve para diagnóstico de transtornos mentais. Rev. Bras. Psiquiatr..

[33] DeSousa DA, Salum GA, Isolan LR, Manfro GG (2013). Sensitivity and Specificity of the Screen for Child Anxiety Related Emotional Disorders (SCARED): A Community-Based Study. Child Psychiatry Hum. Dev..

[34] de Azevedo Marques JM, Zuardi AW (2008). Validity and applicability of the Mini International Neuropsychiatric Interview administered by family medicine residents in primary health care in Brazil. Gen. Hosp. Psychiatry.

[35] Illumina. Infinium ® HumanMethylation450 BeadChip. *Biotechnology* 2–3 (2010).

[36] Wilhelm-Benartzi CS (2013). Review of processing and analysis methods for DNA methylation array data. Br. J. Cancer.

[37] Sandoval J (2011). Validation of a DNA methylation microarray for 450,000 CpG sites in the human genome. Epigenetics.

[38] Bibikova M (2011). High density DNA methylation array with single CpG site resolution. Genomics.

[39] Du P, Kibbe WA, Lin S (2008). M. lumi: A pipeline for processing Illumina microarray. Bioinformatics.

[40] Du P, Bourgon R (2016). methyAnalysis: an R package for DNA methylation data analysis and visualization. R package version 1.14.0..

[41] Leek, J. T. *et al*. sva: Surrogate VariableAnalysis. *R package version 3.20.0.* 1–13 (2016).

[42] Phipson B, Lee S, Majewski IJ, Alexander WS, Smyth GK (2016). Robust hyperparameter estimation protects against hypervariable genes and improves power to detect differential expression. Ann. Appl. Stat..

[43] Wang X, Terfve C, Rose JC, Markowetz F (2011). HTSanalyzeR: an R/Bioconductor package for integrated network analysis of high-throughput screens. Bioinformatics.

[44] Collaborators (2015). Gene Ontology Consortium: going forward. Nucleic Acids Res..

[45] Subramanian A (2005). Gene set enrichment analysis: a knowledge-based approach for interpreting genome-wide expression profiles. Proc. Natl. Acad. Sci. USA.

[46] Benjamini Y, Hochberg Y (1995). Controlling the false discovery rate: a practical and powerful approach to multiple testing. Journal of the Royal Statistical Society.

[47] Bell JT (2012). Epigenome-Wide Scans Identify Differentially Methylated Regions for Age and Age-Related Phenotypes in a Healthy Ageing Population. PLoS Genet..

[48] Martino D (2013). Longitudinal, genome-scale analysis of DNA methylation in twins from birth to 18 months of age reveals rapid epigenetic change in early life and pair-specific effects of discordance. Genome Biol..

[49] Heyn H (2012). Distinct DNA methylomes of newborns and centenarians. Proc. Natl. Acad. Sci. USA.

[50] Talens RP (2012). Epigenetic variation during the adult lifespan: Cross-sectional and longitudinal data on monozygotic twin pairs. Aging Cell.

[51] Deckert, J. *et al*. GLRB allelic variation associated with agoraphobic cognitions, increased startle response and fear network activation: a potential neurogenetic pathway to panic disorder. *Nat*. *Publ*. *Gr*. 1–9, 10.1038/mp.2017.2 (2017). 10.1038/mp.2017.2 28167838

[52] Schartner C (2017). CRHR1 promoter hypomethylation: An epigenetic readout of panic disorder?. Eur. Neuropsychopharmacol..

[53] Weder N (2014). Child abuse, depression, and methylation in genes involved with stress, neural plasticity, and brain circuitry. J. Am. Acad. Child Adolesc. Psychiatry.

[54] Song Y (2014). Altered DNA methylation status of human brain derived neurotrophis factor gene could be useful as biomarker of depression. Am. J. Med. Genet. Part B Neuropsychiatr. Genet..

[55] Liu J (2014). Methylation Patterns in Whole Blood Correlate With Symptoms in Schizophrenia Patients. Schizophr. Bull..

[56] Kinoshita M (2013). DNA Methylation Signatures of Peripheral Leukocytes in Schizophrenia. NeuroMolecular Med..

[57] Oh G (2015). DNA Modification Study of Major Depressive Disorder: Beyond Locus-by-Locus Comparisons. Biol. Psychiatry.

[58] Dempster EL (2014). Genome-wide Methylomic Analysis of Monozygotic Twins Discordant for Adolescent Depression. Biol. Psychiatry.

[59] Uddin M (2010). Epigenetic and immune function profiles associated with posttraumatic stress disorder. Proc. Natl. Acad. Sci..

[60] Yehuda R (2014). Lower Methylation of Glucocorticoid Receptor Gene Promoter 1F in Peripheral Blood of Veterans with Posttraumatic Stress Disorder. Biol. Psychiatry.

[61] Malan-Müller S, Seedat S, Hemmings SMJ (2014). Understanding posttraumatic stress disorder: insights from the methylome. Genes, Brain Behav..

[62] Rutten BPF (2017). Longitudinal analyses of the DNA methylome in deployed military servicemen identify susceptibility loci for post-traumatic stress disorder. Mol. Psychiatr.

[63] Misiak B (2015). Lower LINE-1 methylation in first-episode schizophrenia patients with the history of childhood trauma. Epigenomics.

[64] Perroud N (2016). Methylation of serotonin receptor 3A in adhd, borderline personality, and bipolar disorders: Link with severity of the disorders and childhood maltreatment. Depress. Anxiety.

[65] Barker ED (2017). A Methylome-Wide Association Study of Trajectories of Oppositional Defiant Behaviors and Biological Overlap With Attention Deficit Hyperactivity Disorder. Child Dev..

[66] Fuchikami M (2011). DNA methylation profiles of the brain-derived neurotrophic factor (BDNF) gene as a potent diagnostic biomarker in major depression. PLoS One.

[67] Essex MJ, Boyce WT, Hertzman C, Lam LL, Armstrong JM (2013). Epigenetic Vestiges of Early Developmental Adversity: Childhood Stress Exposure and DNA Methylation in Adolescence. Child Dev..

[68] Yang B-Z (2013). Child abuse and epigenetic mechanisms of disease risk. Am. J. Prev. Med..

[69] Roberts S (2015). HPA axis related genes and response to psychological therapies: Genetics and Epigenetics. Depress. Anxiety.

[70] Roberts S (2014). Serotonin tranporter methylation and response to cognitive behaviour therapy in children with anxiety disorders. Transl. Psychiatry.

[71] Marzi, S. J. *et al*. Analysis of DNA Methylation in Young People: Limited Evidence for an Association Between Victimization Stress and Epigenetic Variation in Blood. *Am J Psychiatry* **Jan**, 1–13 (2018). 10.1176/appi.ajp.2017.17060693 PMC5988939 29325449

[72] Ajonijebu DC, Abboussi O, Russell VA, Mabandla MV, Daniels WMU (2017). Epigenetics: a link between addiction and social environment. Cell. Mol. Life Sci..

[73] Palmisano, M. & Pandey, S. C. Epigenetic Mechanisms Of Alcoholism And Stress- Related Disorders. *Alcohol* **May**, 7–18 (2017). 10.1016/j.alcohol.2017.01.001 PMC5464725 28477725

[74] Kundakovic M (2015). DNA methylation of BDNF as a biomarker of early-life adversity. Proc. Natl. Acad. Sci. USA.

[75] Smith AK (2015). DNA Extracted From Saliva for Methylation Studies of Psychiatric Traits: Evidence Tissue Specificity and Relatedness to Brain. Am J Med Genet B Neuropsychiatr Genet..

[76] Lowe R (2013). Buccals are likely to be a more informative surrogate tissue than blood for epigenome-wide association studies. Epigenetics.

[77] Davies MN (2012). Functional annotation of the human brain methylome identifies tissue-specific epigenetic variation across brain and blood. Genome Biol..

