# Supplementary material for: DNA methylation in adolescents with anxiety disorder: a longitudinal study
Source: Sci Rep. 2018 Sep 14;8:13800. doi: 10.1038/s41598-018-32090-1 (PMC6138655; doi:10.1038/s41598-018-32090-1)
Supplement: Supplementary file 1 — Dataset 1 [file 41598_2018_32090_MOESM1_ESM.pdf]

DNA methylation in adolescents with anxiety disorder: a longitudinal study

Authors: Andressa Bortoluzzi; Giovanni Abrahão Salum; Eduarda Dias da Rosa; Vinícius de Saraiva Chagas; Mauro Antônio Alves Castro and Gisele Gus Manfro

Supplementary Information

Appendix 1: 1

Table S1 - Gene collection of Control group........ 1

Table S2 - Gene collection of Incident group........ 42

Table S3 - Gene collection of Persistent (cases) group........ 62

Table S4 – Gene collection of Remittent group 112

Table S5. Descriptive data in the groups defined by trajectories of anxiety disorders 145

Table S6. Descriptive data about adolescents evaluated in 2008 (n= 234) 146

Appendix 1:

Table S1 - Gene collection of Control group

**Table S1** - Gene collection of Control group	
**Biological processes**	
**Tissue development**	
**Gene Symbol**	**Name Gene**
ACVR1	activin A receptor type 1
ALOX12B	arachidonate 12-lipoxygenase, 12R type
ATP2A2	ATPase sarcoplasmic/endoplasmic reticulum Ca2+ transporting 2
BTD	biotinidase
CASR	calcium sensing receptor
RUNX2	runt related transcription factor 2
CDK6	cyclin-dependent kinase 6
COL1A1	collagen type I alpha 1
COL5A2	collagen type V alpha 2
COL7A1	collagen type VII alpha 1
COL11A1	collagen type XI alpha 1
COL13A1	collagen type XIII alpha 1
CTGF	connective tissue growth fator
DHCR24	24-dehydrocholesterol reductase
DSP	Desmoplakin
EMP1	epithelial membrane protein 1
STX2	syntaxin 2
ERCC3	excision repair cross-complementation group 3
EVPL	Envoplakin
GJB5	gap junction protein beta 5

GLI2	GLI family zinc finger 2
JAK2	Janus kinase 2
KRT1	keratin 1
KRT5	keratin 5
KRT6B	keratin 6B
KRT13	keratin 13
KRT15	keratin 15
KRT32	keratin 32
LAMA3	laminin subunit alpha 3
LAMB3	laminin subunit beta 3
LAMC1	laminin subunit gamma 1
LAMC2	laminin subunit gamma 2
MATK	megakaryocyte-associated tyrosine kinase
MEST	mesoderm specific transcript
NF1	neurofibromin 1
SRGN	Serglycin
PROX1	prospero homeobox 1
SECTM1	secreted and transmembrane 1
SNAI2	snail family transcriptional repressor 2
SPRR1A	small proline rich protein 1A
TBX3	T-box 3
TFAP2A	transcription factor AP-2 alpha
TGFB2	transforming growth factor beta 2
TGM3	transglutaminase 3
TIE1	tyrosine kinase with immunoglobulin like and EGF like domains 1
UGCG	UDP-glucose ceramide glucosyltransferase
SCEL	sciellin
KLF4	Kruppel-like factor 4 (gut)
TGM5	transglutaminase 5
KL	Klotho
IKZF1	IKAROS family zinc finger 1
FST	Follistatin
IKZF3	IKAROS family zinc finger 3
VAX2	ventral anterior homeobox 2
KLK5	kallikrein related peptidase 5
ZBTB7B	zinc finger and BTB domain containing 7B
CALML5	calmodulin like 5
ANKH	ANKH inorganic pyrophosphate transport regulator
SMURF1	SMAD specific E3 ubiquitin protein ligase 1
TWIST2	twist family bHLH transcription factor 2
RTN4RL1	reticulon 4 receptor like 1
RTN4RL2	reticulon 4 receptor-like 2
**Regulation of signal transduction**	
**Gene Symbol**	**Name Gene**
ACVR1	activin A receptor type 1
AMBP	alpha-1-microglobulin/bikunin precursor
FASLG	Fas ligand
RHOH	ras homolog family member H
BST2	bone marrow stromal cell antigen 2
CASP8	caspase 8
CDH13	cadherin 13
DMPK	dystrophia myotonica protein kinase
DTX1	deltex 1
ECM1	extracellular matrix protein 1

ENG	Endoglin
F2R	coagulation factor II thrombin receptor
GJA1	gap junction protein alpha 1
GNG7	G protein subunit gamma 7
GRK4	G protein-coupled receptor kinase 4
GRK5	G protein-coupled receptor kinase 5
GRK6	G protein-coupled receptor kinase 6
GRM4	glutamate metabotropic receptor 4
HCLS1	hematopoietic cell-specific Lyn substrate 1
IGF1	insulin like growth factor 1
IL12A	interleukin 12A
LGALS1	galectin 1
LGALS9	galectin 9
LTBR	lymphotoxin beta receptor
LYN	LYN proto-oncogene, Src family tyrosine kinase
SMAD3	SMAD family member 3
MDFI	MyoD family inhibitor
MAP3K3	mitogen-activated protein kinase kinase kinase 3
NF1	neurofibromin 1
NF2	neurofibromin 2 (merlin)
PPM1A	protein phosphatase, Mg2+/Mn2+ dependent 1A
PTEN	phosphatase and tensin homolog
PTPRC	protein tyrosine phosphatase, receptor type C
RAC1	ras-related C3 botulinum toxin substrate 1 (rho family, small GTP binding protein Rac1)
RELA	RELA proto-oncogene, NF-kB subunit
REN	renin
RGS2	regulator of G-protein signaling 2
RGS3	regulator of G-protein signaling 3
RGS12	regulator of G-protein signaling 12
SECTM1	secreted and transmembrane 1
TPD52L1	tumor protein D52-like 1
TRAF5	TNF receptor associated factor 5
TRAF6	TNF receptor associated factor 6
UBE2N	ubiquitin conjugating enzyme E2 N
ZIC1	Zic family member 1
BRAP	BRCA1 associated protein
NCK2	NCK adaptor protein 2
RGS20	regulator of G-protein signaling 20
TP63	tumor protein p63
TRADD	TNFRSF1A associated via death domain
RIPK1	receptor interacting serine/threonine kinase 1
RIPK2	receptor interacting serine/threonine kinase 2
RGS9	regulator of G-protein signaling 9
CFLAR	CASP8 and FADD like apoptosis regulator
HGS	hepatocyte growth factor-regulated tyrosine kinase substrate
LITAF	lipopolysaccharide induced TNF factor
CDC42BPB	CDC42 binding protein kinase beta
IKBKE	inhibitor of kappa light polypeptide gene enhancer in B-cells, kinase epsilon
GIT2	GIT ArfGAP 2
RAMP1	receptor activity modifying protein 1
TLR6	toll like receptor 6
NOD1	nucleotide binding oligomerization domain containing 1
TRIM38	tripartite motif containing 38
RGS14	regulator of G-protein signaling 14
PLK2	polo like kinase 2
NEK6	NIMA related kinase 6
RALBP1	ralA binding protein 1

CARD8	caspase recruitment domain family member 8
TAB2	TGF-beta activated kinase 1/MAP3K7 binding protein 2
MAPK8IP3	mitogen-activated protein kinase 8 interacting protein 3
ZDHHC17	zinc finger DHHC-type containing 17
CBLC	Cbl proto-oncogene C
CBY1	chibby homolog 1 (Drosophila)
ATP2C1	ATPase secretory pathway Ca2+ transporting 1
GIT1	GIT ArfGAP 1
HIPK2	homeodomain interacting protein kinase 2
MDFIC	MyoD family inhibitor domain containing
IL20	interleukin 20
TAOK3	TAO kinase 3
CXXC5	CXXC finger protein 5
NLK	nemo like kinase
ZDHHC13	zinc finger DHHC-type containing 13
NDFIP2	Nedd4 family interacting protein 2
CDC42BPG	CDC42 binding protein kinase gamma
CDKN2AIP	CDKN2A interacting protein
FGD6	FYVE, RhoGEF and PH domain containing 6
OTUD7B	OTU deubiquitinase 7B
SLC44A2	solute carrier family 44 member 2
SMURF1	SMAD specific E3 ubiquitin protein ligase 1
PLEKHG5	pleckstrin homology and RhoGEF domain containing G5
SIGIRR	single immunoglobulin and toll-interleukin 1 receptor (TIR) domain
CARD9	caspase recruitment domain family member 9
ARAP3	ArfGAP with RhoGAP domain, ankyrin repeat and PH domain 3
VOPP1	vesicular, overexpressed in cancer, prosurvival protein 1
SLA2	Src-like-adaptor 2
TRAF7	TNF receptor associated factor 7
AFAP1L2	actin filament associated protein 1 like 2
NLRP12	NLR family, pyrin domain containing 12
IL22RA2	interleukin 22 receptor subunit alpha 2
ARAP1	ArfGAP with RhoGAP domain, ankyrin repeat and PH domain 1
FGD4	FYVE, RhoGEF and PH domain containing 4
IL31RA	interleukin 31 receptor A
ABRA	actin binding Rho activating protein
MIB2	mindbomb E3 ubiquitin protein ligase 2
NFAM1	NFAT activating protein with ITAM motif 1
FGD5	FYVE, RhoGEF and PH domain containing 5
ARHGAP27	Rho GTPase activating protein 27
FGD2	FYVE, RhoGEF and PH domain containing 2
EPGN	epithelial mitogen
VWC2	von Willebrand factor C domain containing 2
**Cellular protein metabolic process**	
**Gene Symbol**	**Name Gene**
AARS	alanyl-tRNA synthetase
ABCF1	ATP binding cassette subfamily F member 1
ABL1	ABL proto-oncogene 1, non-receptor tyrosine kinase
ABL2	ABL proto-oncogene 2, non-receptor tyrosine kinase
ACVR1	activin A receptor type 1
ACVRL1	activin A receptor like type 1
PARP1	poly(ADP-ribose) polymerase 1
PARP4	poly(ADP-ribose) polymerase family member 4
AGA	aspartylglucosaminidase
AKT2	AKT serine/threonine kinase 2
ALK	anaplastic lymphoma receptor tyrosine kinase
APBB1	amyloid beta precursor protein binding family B member 1
APC	adenomatous polyposis coli

APOA4	apolipoprotein A4
ART3	ADP-ribosyltransferase 3
AZU1	azurocidin 1
CCND1	cyclin D1
BMPR1A	bone morphogenetic protein receptor type 1A
BMPR1B	bone morphogenetic protein receptor type 1B
BRCA1	breast cancer 1
BRAF	B-Raf proto-oncogene, serine/threonine kinase
C2	complement component 2
CAMK2B	calcium/calmodulin dependent protein kinase II beta
CAPN3	calpain 3
CASP8	caspase 8
CCND2	cyclin D2
CD3E	CD3e molecule
CD28	CD28 molecule
CD80	CD80 molecule
TNFRSF8	tumor necrosis factor receptor superfamily member 8
CD37	CD37 molecule
CD81	CD81 molecule
CDK11B	cyclin-dependent kinase 11B
CETP	cholesteryl ester transfer protein
CHRM1	cholinergic receptor muscarinic 1
CHRM3	cholinergic receptor muscarinic 3
ERCC8	excision repair cross-complementation group 8
PLK3	polo like kinase 3
MAP3K8	mitogen-activated protein kinase kinase kinase 8
COX10	COX10 heme A:farnesyltransferase cytochrome c oxidase assembly factor
CPA3	carboxypeptidase A3
CPB2	carboxypeptidase B2
CREBBP	CREB binding protein
CSNK1A1	casein kinase 1 alpha 1
CSNK1D	casein kinase 1 delta
CSNK1G2	casein kinase 1 gamma 2
CSNK1G3	casein kinase 1 gamma 3
CTBP1	C-terminal binding protein 1
CTSH	cathepsin H
CTSO	cathepsin O
CTSZ	cathepsin Z
DAPK1	death associated protein kinase 1
DAPK3	death-associated protein kinase 3
DARS	aspartyl-tRNA synthetase
ACE	angiotensin I converting enzyme
CFD	complement factor D
DMPK	dystrophia myotonica protein kinase
DSP	desmoplakin
DUSP2	dual specificity phosphatase 2
DUSP3	dual specificity phosphatase 3
DUSP4	dual specificity phosphatase 4
DUSP5	dual specificity phosphatase 5
DUSP6	dual specificity phosphatase 6
DYRK1A	dual specificity tyrosine phosphorylation regulated kinase 1A
EGFR	epidermal growth factor receptor
EIF4A2	eukaryotic translation initiation factor 4A2
EIF5	eukaryotic translation initiation factor 5
ELANE	elastase, neutrophil expressed
MARK2	microtubule affinity regulating kinase 2
EP300	E1A binding protein p300
EPHA8	EPH receptor A8

EPHB2	EPH receptor B2
ERBB2	erb-b2 receptor tyrosine kinase 2
ERBB3	erb-b2 receptor tyrosine kinase 3
ERG	v-ets avian erythroblastosis virus E26 oncogene homolog
ERN1	endoplasmic reticulum to nucleus signaling 1
EVPL	envoplakin
EXT1	exostosin glycosyltransferase 1
F2R	coagulation factor II thrombin receptor
FGR	FGR proto-oncogene, Src family tyrosine kinase
FMOD	fibromodulin
FRK	fyn related Src family tyrosine kinase
FUT5	fucosyltransferase 5
FUT6	fucosyltransferase 6
FYB	FYN binding protein
GAD1	glutamate decarboxylase 1
GALNT1	polypeptide N-acetylgalactosaminyltransferase 1
GALNT2	polypeptide N-acetylgalactosaminyltransferase 2
GGCX	gamma-glutamyl carboxylase
MKNK2	MAP kinase interacting serine/threonine kinase 2
GSN	gelsolin
GYPC	glycophorin C (Gerbich blood group)
HCLS1	hematopoietic cell-specific Lyn substrate 1
HGFAC	HGF activator
NRG1	neuregulin 1
HMGA1	high mobility group AT-hook 1
PRMT1	protein arginine methyltransferase 1
HSPB1	heat shock protein family B (small) member 1
NDST1	N-deacetylase/N-sulfotransferase 1
IDE	insulin degrading enzyme
IGF1R	insulin like growth factor 1 receptor
IL6	interleukin 6
IL10	interleukin 10
IL12A	interleukin 12A
IL18	interleukin 18
INHBA	inhibin beta A
INHBB	inhibin beta B
INSR	insulin receptor
IRAK2	interleukin 1 receptor associated kinase 2
IRF4	interferon regulatory factor 4
ITGB2	integrin subunit beta 2
JAK2	Janus kinase 2
JAK3	Janus kinase 3
KRT1	keratin 1
KRT7	keratin 7
STMN1	stathmin 1
LCK	LCK proto-oncogene, Src family tyrosine kinase
LDLR	low density lipoprotein receptor
LIMK1	LIM domain kinase 1
LIPA	lipase A, lysosomal acid type
LIPC	lipase C, hepatic type
LMAN1	lectin, mannose binding 1
LOX	lysyl oxidase
LOXL1	lysyl oxidase like 1
LOXL2	lysyl oxidase like 2
LRPAP1	LDL receptor related protein associated protein 1
LTB	lymphotoxin beta
LYN	LYN proto-oncogene, Src family tyrosine kinase
SMAD3	SMAD family member 3

MAPT	microtubule associated protein tau
MATK	megakaryocyte-associated tyrosine kinase
MBL2	mannose binding lectin 2
MDM2	MDM2 proto-oncogene
MDM4	MDM4, p53 regulator
MAP3K3	mitogen-activated protein kinase kinase kinase 3
MIPEP	mitochondrial intermediate peptidase
MAP3K11	mitogen-activated protein kinase kinase kinase 11
MMP2	matrix metallopeptidase 2
MMP3	matrix metallopeptidase 3
MMP10	matrix metallopeptidase 10
MMP11	matrix metallopeptidase 11
MMP15	matrix metallopeptidase 15
MSRA	methionine sulfoxide reductase A
MTIF2	mitochondrial translational initiation factor 2
MYH9	myosin, heavy chain 9, non-muscle
MYLK	myosin light chain kinase
NUBP1	nucleotide binding protein 1
NF2	neurofibromin 2 (merlin)
OLR1	oxidized low density lipoprotein receptor 1
P4HB	prolyl 4-hydroxylase subunit beta
FURIN	furin, paired basic amino acid cleaving enzyme
PCSK6	proprotein convertase subtilisin/kexin type 6
PAK1	p21 (RAC1) activated kinase 1
PAK2	p21 (RAC1) activated kinase 2
PAM	peptidylglycine alpha-amidating monooxygenase
PARK2	parkin RBR E3 ubiquitin protein ligase
PCSK1	proprotein convertase subtilisin/kexin type 1
PCSK5	proprotein convertase subtilisin/kexin type 5
CDK17	cyclin-dependent kinase 17
PDPK1	3-phosphoinositide dependent protein kinase 1
PEPD	peptidase D
PIGH	phosphatidylinositol glycan anchor biosynthesis class H
PIM1	Pim-1 proto-oncogene, serine/threonine kinase
PLAT	plasminogen activator, tissue type
PLOD2	procollagen-lysine,2-oxoglutarate 5-dioxygenase 2
PPM1B	protein phosphatase, Mg2+/Mn2+ dependent 1B
PPM1G	protein phosphatase, Mg2+/Mn2+ dependent 1G
PPP1CA	protein phosphatase 1 catalytic subunit alpha
PPP2R2A	protein phosphatase 2 regulatory subunit Balpha
PPP2R3A	protein phosphatase 2 regulatory subunit B''alpha
PPP2R5C	protein phosphatase 2 regulatory subunit B'gamma
PPT1	palmitoyl-protein thioesterase 1
PREP	prolyl endopeptidase
SRGN	serglycin
PRKAA1	protein kinase AMP-activated catalytic subunit alpha 1
PRKCB	protein kinase C beta
PRKCD	protein kinase C delta
PRKCE	protein kinase C epsilon
PRKCH	protein kinase C eta
PKN1	protein kinase N1
PKN2	protein kinase N2
PRKCZ	protein kinase C zeta
PRKDC	protein kinase, DNA-activated, catalytic polypeptide
PRKG1	protein kinase, cGMP-dependent, type I
PRKG2	protein kinase, cGMP-dependent, type II
MAPK8	mitogen-activated protein kinase 8
MAP2K6	mitogen-activated protein kinase kinase 6

PRLR	prolactin receptor
PTEN	phosphatase and tensin homolog
PTK6	protein tyrosine kinase 6
QSOX1	quiescin sulfhydryl oxidase 1
PTPN3	protein tyrosine phosphatase, non-receptor type 3
PTPN4	protein tyrosine phosphatase, non-receptor type 4
PTPN6	protein tyrosine phosphatase, non-receptor type 6
PTPN7	protein tyrosine phosphatase, non-receptor type 7
PTPN12	protein tyrosine phosphatase, non-receptor type 12
PTPN14	protein tyrosine phosphatase, non-receptor type 14
PTPRB	protein tyrosine phosphatase, receptor type B
PTPRC	protein tyrosine phosphatase, receptor type C
PTPRD	protein tyrosine phosphatase, receptor type D
PTPRE	protein tyrosine phosphatase, receptor type E
PTPRH	protein tyrosine phosphatase, receptor type H
PTPRJ	protein tyrosine phosphatase, receptor type J
PTPRN2	protein tyrosine phosphatase, receptor type N2
PTPRO	protein tyrosine phosphatase, receptor type O
PTPRR	protein tyrosine phosphatase, receptor type R
PXN	paxillin
RAC1	ras-related C3 botulinum toxin substrate 1 (rho family, small GTP binding protein Rac1)
REN	renin
RPL3	ribosomal protein L3
RPL3L	ribosomal protein L3 like
RPL7	ribosomal protein L7
RPL7A	ribosomal protein L7a
RPL18A	ribosomal protein L18a
RPL23A	ribosomal protein L23a
MRPL23	mitochondrial ribosomal protein L23
RPL24	ribosomal protein L24
RPL26	ribosomal protein L26
RPN1	ribophorin I
RPS2	ribosomal protein S2
RPS6KB2	ribosomal protein S6 kinase B2
RPS11	ribosomal protein S11
RRBP1	ribosome binding protein 1
CCL2	C-C motif chemokine ligand 2
CCL11	C-C motif chemokine ligand 11
SFTPD	surfactant protein D
SGK1	serum/glucocorticoid regulated kinase 1
SCG5	secretogranin V
SGSH	N-sulfoglucosamine sulfohydrolase
SHH	sonic hedgehog
SIAH2	siah E3 ubiquitin protein ligase 2
SPG7	SPG7, paraplegin matrix AAA peptidase subunit
SPRR1A	small proline rich protein 1A
SRC	SRC proto-oncogene, non-receptor tyrosine kinase
SRP72	signal recognition particle 72kDa
SRPK1	SRSF protein kinase 1
SRPK2	SRSF protein kinase 2
ST14	suppression of tumorigenicity 14
NEK4	NIMA related kinase 4
STK10	serine/threonine kinase 10
TBCA	tubulin folding cofactor A
TBCD	tubulin folding cofactor D
TBCE	tubulin folding cofactor E
TGFBR1	transforming growth factor beta receptor 1

TGM3	transglutaminase 3
TLR1	toll like receptor 1
TLR3	toll like receptor 3
TLR4	toll like receptor 4
TM4SF4	transmembrane 4 L six family member 4
TNP1	transition protein 1
TPP2	tripeptidyl peptidase 2
TRAF6	TNF receptor associated factor 6
TTC1	tetratricopeptide repeat domain 1
TTN	titin
UBB	ubiquitin B
UBE2D1	ubiquitin conjugating enzyme E2 D1
UBE2D3	ubiquitin conjugating enzyme E2 D3
UBE2E1	ubiquitin conjugating enzyme E2 E1
UBE2E2	ubiquitin conjugating enzyme E2 E2
UBE2G1	ubiquitin conjugating enzyme E2 G1
UBE2G2	ubiquitin conjugating enzyme E2 G2
UBE2H	ubiquitin conjugating enzyme E2 H
UBE2I	ubiquitin conjugating enzyme E2 I
UBE2L3	ubiquitin conjugating enzyme E2 L3
UBE2N	ubiquitin conjugating enzyme E2 N
UBE2V2	ubiquitin conjugating enzyme E2 V2
UBE3A	ubiquitin protein ligase E3A
VHL	von Hippel-Lindau tumor suppressor
VRK2	vaccinia related kinase 2
EIF4H	eukaryotic translation initiation factor 4H
XPNPEP1	X-prolyl aminopeptidase (aminopeptidase P) 1, soluble
USP7	ubiquitin specific peptidase 7 (herpes virus-associated)
ST8SIA4	ST8 alpha-N-acetyl-neuraminide alpha-2,8-sialyltransferase 4
EPM2A	epilepsy, progressive myoclonus type 2A, Lafora disease (laforin)
KAT6A	lysine acetyltransferase 6A
NR4A3	nuclear receptor subfamily 4 group A member 3
CDK2AP1	cyclin-dependent kinase 2 associated protein 1
ST8SIA2	ST8 alpha-N-acetyl-neuraminide alpha-2,8-sialyltransferase 2
MKKS	McKusick-Kaufman syndrome
ULK1	unc-51 like autophagy activating kinase 1
LTBP4	latent transforming growth factor beta binding protein 4
NCK2	NCK adaptor protein 2
DYRK3	dual specificity tyrosine phosphorylation regulated kinase 3
TTF2	transcription termination factor, RNA polymerase II
TPST1	tyrosylprotein sulfotransferase 1
FKBP6	FK506 binding protein 6
CDC42BPA	CDC42 binding protein kinase alpha
CPZ	carboxypeptidase Z
MKNK1	MAP kinase interacting serine/threonine kinase 1
SCARF1	scavenger receptor class F member 1
TNKS	tankyrase
EIF3A	eukaryotic translation initiation factor 3 subunit A
EIF3C	eukaryotic translation initiation factor 3 subunit C
EIF3F	eukaryotic translation initiation factor 3 subunit F
EIF3G	eukaryotic translation initiation factor 3 subunit G
EIF3H	eukaryotic translation initiation factor 3 subunit H
EIF4G3	eukaryotic translation initiation factor 4 gamma 3
B3GALT4	beta-1,3-galactosyltransferase 4
B3GALNT1	beta-1,3-N-acetylgalactosaminyltransferase 1 (globoside blood group)
MBTPS1	membrane bound transcription factor peptidase, site 1
CDKL1	cyclin dependent kinase like 1
DPM2	dolichyl-phosphate mannosyltransferase polypeptide 2, regulatory subunit

EIF2B3	eukaryotic translation initiation factor 2B subunit gamma
PRPF4B	pre-mRNA processing factor 4B
ENDOU	endonuclease, poly(U) specific
BTRC	beta-transducin repeat containing E3 ubiquitin protein ligase
PLOD3	procollagen-lysine,2-oxoglutarate 5-dioxygenase 3
RPS6KA4	ribosomal protein S6 kinase A4
KALRN	kalirin, RhoGEF kinase
BRSK2	BR serine/threonine kinase 2
TM4SF5	transmembrane 4 L six family member 5
UBA3	ubiquitin like modifier activating enzyme 3
MAP3K6	mitogen-activated protein kinase kinase kinase 6
MTMR7	myotubularin related protein 7
DYRK1B	dual specificity tyrosine phosphorylation regulated kinase 1B
CTDP1	CTD phosphatase subunit 1
MAP3K13	mitogen-activated protein kinase kinase kinase 13
DCLK1	doublecortin like kinase 1
GCNT3	glucosaminyl (N-acetyl) transferase 3, mucin type
RPS6KA5	ribosomal protein S6 kinase A5
STK17B	serine/threonine kinase 17b
STK17A	serine/threonine kinase 17a
TGM5	transglutaminase 5
HS6ST1	heparan sulfate 6-O-sulfotransferase 1
NMT2	N-myristoyltransferase 2
MAP4K4	mitogen-activated protein kinase kinase kinase kinase 4
ROCK2	Rho associated coiled-coil containing protein kinase 2
ADAMTS4	ADAM metallopeptidase with thrombospondin type 1 motif 4
MPDU1	mannose-P-dolichol utilization defect 1
GMFG	glia maturation factor gamma
CDC42BPB	CDC42 binding protein kinase beta
RNF14	ring finger protein 14
ISG15	ISG15 ubiquitin-like modifier
IKBKE	inhibitor of kappa light polypeptide gene enhancer in B-cells, kinase epsilon
KDM4A	lysine demethylase 4A
UBE3C	ubiquitin protein ligase E3C
EDEM1	ER degradation enhancing alpha-mannosidase like protein 1
ECE2	endothelin converting enzyme 2
TLK1	tousled like kinase 1
OXSR1	oxidative stress responsive 1
HS3ST3B1	heparan sulfate-glucosamine 3-sulfotransferase 3B1
AKT3	AKT serine/threonine kinase 3
DNAJB6	DnaJ heat shock protein family (Hsp40) member B6
PTPRU	protein tyrosine phosphatase, receptor type U
UST	uronyl 2-sulfotransferase
HIPK3	homeodomain interacting protein kinase 3
CHST4	carbohydrate sulfotransferase 4
TNK2	tyrosine kinase non receptor 2
TRIB1	tribbles pseudokinase 1
RIDA	reactive intermediate imine deaminase A homolog
TLR6	toll like receptor 6
NOD1	nucleotide binding oligomerization domain containing 1
ST3GAL6	ST3 beta-galactoside alpha-2,3-sialyltransferase 6
TESK2	testis-specific kinase 2
PPIH	peptidylprolyl isomerase H
CARM1	coactivator associated arginine methyltransferase 1
PITRM1	pitrilysin metallopeptidase 1
ATG7	autophagy related 7
IGF2BP2	insulin like growth factor 2 mRNA binding protein 2
CAMKK2	calcium/calmodulin-dependent protein kinase kinase 2

FARS2	phenylalanyl-tRNA synthetase 2, mitochondrial
CORIN	corin, serine peptidase
MAP3K2	mitogen-activated protein kinase kinase kinase 2
NEK6	NIMA related kinase 6
WASF3	WAS protein family member 3
CLPX	caseinolytic mitochondrial matrix peptidase chaperone subunit
HPSE	heparanase
PPARGC1A	PPARG coactivator 1 alpha
MAN1A2	mannosidase alpha class 1A member 2
PDIA5	protein disulfide isomerase family A member 5
METAP2	methionyl aminopeptidase 2
ADRM1	adhesion regulating molecule 1
WWP2	WW domain containing E3 ubiquitin protein ligase 2
LECT1	leukocyte cell derived chemotaxin 1
UBE2C	ubiquitin conjugating enzyme E2 C
TPPP	tubulin polymerization promoting protein
PTPRT	protein tyrosine phosphatase, receptor type T
MAP4K5	mitogen-activated protein kinase kinase kinase kinase 5
MAP4K1	mitogen-activated protein kinase kinase kinase kinase 1
IRAK3	interleukin 1 receptor associated kinase 3
DUSP10	dual specificity phosphatase 10
MGAT4B	mannosyl (alpha-1,3-)-glycoprotein beta-1,4-N-acetylglucosaminyltransferase, isozyme B
MGAT4A	mannosyl (alpha-1,3-)-glycoprotein beta-1,4-N-acetylglucosaminyltransferase, isozyme A
STK38	serine/threonine kinase 38
LMTK2	lemur tyrosine kinase 2
TRAK1	trafficking kinesin protein 1
MAPRE1	microtubule associated protein RP/EB family member 1
ATF6	activating transcription factor 6
MAST1	microtubule associated serine/threonine kinase 1
STK38L	serine/threonine kinase 38 like
SAMD4A	sterile alpha motif domain containing 4A
SMG1	SMG1, nonsense mediated mRNA decay associated PI3K related kinase
ERC1	ELKS/RAB6-interacting/CAST family member 1
ATG4B	autophagy related 4B cysteine peptidase
CLASP1	cytoplasmic linker associated protein 1
SIK3	SIK family kinase 3
ICMT	isoprenylcysteine carboxyl methyltransferase
KAT6B	lysine acetyltransferase 6B
BACE1	beta-secretase 1
CBLC	Cbl proto-oncogene C
PRKD3	protein kinase D3
AIPL1	aryl hydrocarbon receptor interacting protein like 1
FBXO7	F-box protein 7
QPCT	glutaminyl-peptide cyclotransferase
GLCE	glucuronic acid epimerase
FBXW2	F-box and WD repeat domain containing 2
FBXL6	F-box and leucine-rich repeat protein 6
FBXO22	F-box protein 22
FBXO3	F-box protein 3
LATS2	large tumor suppressor kinase 2
EIF2AK1	eukaryotic translation initiation factor 2 alpha kinase 1
SERP1	stress-associated endoplasmic reticulum protein 1
TRIB2	tribbles pseudokinase 2
MYLIP	myosin regulatory light chain interacting protein
NPC1L1	NPC1 like intracellular cholesterol transporter 1
EEF2K	eukaryotic elongation factor 2 kinase

MINK1	misshapen like kinase 1
CHST11	carbohydrate (chondroitin 4) sulfotransferase 11
IL20	interleukin 20
NDUFA13	NADH:ubiquinone oxidoreductase subunit A13
YBX2	Y-box binding protein 2
SEPSECS	Sep (O-phosphoserine) tRNA:Sec (selenocysteine) tRNA synthase
A4GNT	alpha-1,4-N-acetylglucosaminyltransferase
ING4	inhibitor of growth family member 4
ABI3	ABI family member 3
CDKL3	cyclin dependent kinase like 3
GCNT4	glucosaminyl (N-acetyl) transferase 4, core 2
FZR1	fizzy/cell division cycle 20 related 1
TAOK3	TAO kinase 3
UBR5	ubiquitin protein ligase E3 component n-recognin 5
LIMA1	LIM domain and actin binding 1
PIGT	phosphatidylinositol glycan anchor biosynthesis class T
SUFU	SUFU negative regulator of hedgehog signaling
ERAP1	endoplasmic reticulum aminopeptidase 1
ZAK	sterile alpha motif and leucine zipper containing kinase AZK
GALNT7	polypeptide N-acetylgalactosaminyltransferase 7
RIPK4	receptor interacting serine/threonine kinase 4
TLR9	toll like receptor 9
SSH1	slingshot protein phosphatase 1
PRMT7	protein arginine methyltransferase 7
SNRK	SNF related kinase
PIGG	phosphatidylinositol glycan anchor biosynthesis class G
CLN6	ceroid-lipofuscinosis, neuronal 6, late infantile, variant
FBXW7	F-box and WD repeat domain containing 7
CHST12	carbohydrate (chondroitin 4) sulfotransferase 12
CDC42BPG	CDC42 binding protein kinase gamma
CDKN2AIP	CDKN2A interacting protein
PIGV	phosphatidylinositol glycan anchor biosynthesis class V
CSGALNACT1	chondroitin sulfate N-acetylgalactosaminyltransferase 1
CAND1	cullin associated and neddylation dissociated 1
CENPJ	centromere protein J
ALG1	ALG1, chitobiosyldiphosphodolichol beta-mannosyltransferase
DUSP22	dual specificity phosphatase 22
ADAMTS9	ADAM metallopeptidase with thrombospondin type 1 motif 9
MAN1C1	mannosidase alpha class 1C member 1
SMURF1	SMAD specific E3 ubiquitin protein ligase 1
DOLPP1	dolichyldiphosphatase 1
PREX1	phosphatidylinositol-3,4,5-trisphosphate dependent Rac exchange factor 1
MARK4	microtubule affinity regulating kinase 4
SIGIRR	single immunoglobulin and toll-interleukin 1 receptor (TIR) domain
NECAB3	N-terminal EF-hand calcium binding protein 3
DNAJC1	DnaJ heat shock protein family (Hsp40) member C1
NSD1	nuclear receptor binding SET domain protein 1
CHST8	carbohydrate sulfotransferase 8
SMURF2	SMAD specific E3 ubiquitin protein ligase 2
PINK1	PTEN induced putative kinase 1
WNK1	WNK lysine deficient protein kinase 1
BOLL	boule homolog, RNA binding protein
MIS12	MIS12, kinetochore complex component
CARD14	caspase recruitment domain family member 14
ISOC2	isochorismatase domain containing 2
EHMT1	euchromatic histone lysine methyltransferase 1
NEK11	NIMA related kinase 11
HDAC11	histone deacetylase 11

NAA15	N(alpha)-acetyltransferase 15, NatA auxiliary subunit
NAA50	N(alpha)-acetyltransferase 50, NatE catalytic subunit
CD276	CD276 molecule
MEX3B	mex-3 RNA binding family member B
TRAF7	TNF receptor associated factor 7
ING5	inhibitor of growth family member 5
BRSK1	BR serine/threonine kinase 1
KIAA1804	mixed lineage kinase 4
C9orf3	chromosome 9 open reading frame 3
ATG4C	autophagy related 4C cysteine peptidase
ATG4D	autophagy related 4D cysteine peptidase
SSH2	slingshot protein phosphatase 2
HPS4	HPS4, biogenesis of lysosomal organelles complex 3 subunit 2
NLRP12	NLR family, pyrin domain containing 12
TP53RK	TP53 regulating kinase
IL22RA2	interleukin 22 receptor subunit alpha 2
UHMK1	U2AF homology motif (UHM) kinase 1
IL31RA	interleukin 31 receptor A
SIK1	salt inducible kinase 1
PPP4R2	protein phosphatase 4 regulatory subunit 2
TMPRSS6	transmembrane protease, serine 6
STT3B	STT3B, catalytic subunit of the oligosaccharyltransferase complex
HS3ST5	heparan sulfate-glucosamine 3-sulfotransferase 5
MAPK15	mitogen-activated protein kinase 15
MSRB3	methionine sulfoxide reductase B3
METAP1D	methionyl aminopeptidase type 1D (mitochondrial)
RNF144B	ring finger protein 144B
COX18	COX18 cytochrome c oxidase assembly factor
NDUFS7	NADH:ubiquinone oxidoreductase core subunit S7
AGRN	agrin
CCDC88C	coiled-coil domain containing 88C
EIF2AK4	eukaryotic translation initiation factor 2 alpha kinase 4
D2HGDH	D-2-hydroxyglutarate dehydrogenase
**Protein metabolic process**	
**Gene Symbol**	**Name Gene**
AARS	alanyl-tRNA synthetase
ABCF1	ATP binding cassette subfamily F member 1
ABL1	ABL proto-oncogene 1, non-receptor tyrosine kinase
ABL2	ABL proto-oncogene 2, non-receptor tyrosine kinase
ACVR1	activin A receptor type 1
ACVRL1	activin A receptor like type 1
PARP1	poly(ADP-ribose) polymerase 1
PARP4	poly(ADP-ribose) polymerase family member 4
AGA	aspartylglucosaminidase
AKT2	AKT serine/threonine kinase 2
ALK	anaplastic lymphoma receptor tyrosine kinase
APBA1	amyloid beta precursor protein binding family A member 1
APBB1	amyloid beta precursor protein binding family B member 1
APC	adenomatous polyposis coli
APOA4	apolipoprotein A4
ART3	ADP-ribosyltransferase 3
AZU1	azurocidin 1
CCND1	cyclin D1
BMPR1A	bone morphogenetic protein receptor type 1A
BMPR1B	bone morphogenetic protein receptor type 1B
BRCA1	breast cancer 1
BRAF	B-Raf proto-oncogene, serine/threonine kinase
C2	complement component 2

CAMK2B	calcium/calmodulin dependent protein kinase II beta
CAPN3	calpain 3
CASP8	caspase 8
CCND2	cyclin D2
CD3E	CD3e molecule
CD9	CD9 molecule
CD28	CD28 molecule
CD80	CD80 molecule
TNFRSF8	tumor necrosis factor receptor superfamily member 8
CD37	CD37 molecule
CD81	CD81 molecule
CDK11B	cyclin-dependent kinase 11B
CETP	cholesteryl ester transfer protein
CHRM1	cholinergic receptor muscarinic 1
CHRM3	cholinergic receptor muscarinic 3
ERCC8	excision repair cross-complementation group 8
PLK3	polo like kinase 3
MAP3K8	mitogen-activated protein kinase kinase kinase 8
COX10	COX10 heme A:farnesyltransferase cytochrome c oxidase assembly factor
CPA3	carboxypeptidase A3
CPB2	carboxypeptidase B2
CREBBP	CREB binding protein
CSNK1A1	casein kinase 1 alpha 1
CSNK1D	casein kinase 1 delta
CSNK1G2	casein kinase 1 gamma 2
CSNK1G3	casein kinase 1 gamma 3
CTBP1	C-terminal binding protein 1
CTSH	cathepsin H
CTSO	cathepsin O
CTSZ	cathepsin Z
DAPK1	death associated protein kinase 1
DAPK3	death-associated protein kinase 3
DARS	aspartyl-tRNA synthetase
ACE	angiotensin I converting enzyme
CFD	complement factor D
DLG4	discs large homolog 4
DMPK	dystrophia myotonica protein kinase
DSP	desmoplakin
DUSP2	dual specificity phosphatase 2
DUSP3	dual specificity phosphatase 3
DUSP4	dual specificity phosphatase 4
DUSP5	dual specificity phosphatase 5
DUSP6	dual specificity phosphatase 6
DYRK1A	dual specificity tyrosine phosphorylation regulated kinase 1A
EGFR	epidermal growth factor receptor
EIF4A2	eukaryotic translation initiation factor 4A2
EIF5	eukaryotic translation initiation factor 5
ELANE	elastase, neutrophil expressed
MARK2	microtubule affinity regulating kinase 2
EP300	E1A binding protein p300
EPHA8	EPH receptor A8
EPHB2	EPH receptor B2
ERBB2	erb-b2 receptor tyrosine kinase 2
ERBB3	erb-b2 receptor tyrosine kinase 3
ERG	v-ets avian erythroblastosis virus E26 oncogene homolog
ERN1	endoplasmic reticulum to nucleus signaling 1
EVPL	envoplakin
EXT1	exostosin glycosyltransferase 1

F2R	coagulation factor II thrombin receptor
FANCC	Fanconi anemia complementation group C
FGR	FGR proto-oncogene, Src family tyrosine kinase
FMOD	fibromodulin
FRK	fyn related Src family tyrosine kinase
MTOR	mechanistic target of rapamycin
FUT5	fucosyltransferase 5
FUT6	fucosyltransferase 6
FYB	FYN binding protein
GAD1	glutamate decarboxylase 1
GALNT1	polypeptide N-acetylgalactosaminyltransferase 1
GALNT2	polypeptide N-acetylgalactosaminyltransferase 2
GGCX	gamma-glutamyl carboxylase
GJA1	gap junction protein alpha 1
MKNK2	MAP kinase interacting serine/threonine kinase 2
GSN	gelsolin
GYPC	glycophorin C (Gerbich blood group)
HCLS1	hematopoietic cell-specific Lyn substrate 1
HGFAC	HGF activator
NRG1	neuregulin 1
HMGA1	high mobility group AT-hook 1
PRMT1	protein arginine methyltransferase 1
HSPB1	heat shock protein family B (small) member 1
NDST1	N-deacetylase/N-sulfotransferase 1
IDE	insulin degrading enzyme
IGF1R	insulin like growth factor 1 receptor
IL2RB	interleukin 2 receptor subunit beta
IL6	interleukin 6
IL10	interleukin 10
IL12A	interleukin 12A
IL18	interleukin 18
INHBA	inhibin beta A
INHBB	inhibin beta B
INSR	insulin receptor
IRAK2	interleukin 1 receptor associated kinase 2
IRF4	interferon regulatory factor 4
ITGB2	integrin subunit beta 2
JAK2	Janus kinase 2
JAK3	Janus kinase 3
KPNA3	karyopherin subunit alpha 3
KRT1	keratin 1
KRT7	keratin 7
LAMC1	laminin subunit gamma 1
STMN1	stathmin 1
LCK	LCK proto-oncogene, Src family tyrosine kinase
LDLR	low density lipoprotein receptor
LIMK1	LIM domain kinase 1
LIPA	lipase A, lysosomal acid type
LIPC	lipase C, hepatic type
LLGL1	LLGL1, scribble cell polarity complex component
LMAN1	lectin, mannose binding 1
LOX	lysyl oxidase
LOXL1	lysyl oxidase like 1
LOXL2	lysyl oxidase like 2
LRPAP1	LDL receptor related protein associated protein 1
LTB	lymphotoxin beta
LYN	LYN proto-oncogene, Src family tyrosine kinase
SMAD3	SMAD family member 3

MAPT	microtubule associated protein tau
MATK	megakaryocyte-associated tyrosine kinase
MATN1	matrilin 1, cartilage matrix protein
MBL2	mannose binding lectin 2
MDM2	MDM2 proto-oncogene
MDM4	MDM4, p53 regulator
MAP3K3	mitogen-activated protein kinase kinase kinase 3
MIPEP	mitochondrial intermediate peptidase
MAP3K11	mitogen-activated protein kinase kinase kinase 11
MMP2	matrix metallopeptidase 2
MMP3	matrix metallopeptidase 3
MMP10	matrix metallopeptidase 10
MMP11	matrix metallopeptidase 11
MMP15	matrix metallopeptidase 15
MNAT1	MNAT1, CDK activating kinase assembly factor
MSRA	methionine sulfoxide reductase A
MTIF2	mitochondrial translational initiation factor 2
MYH9	myosin, heavy chain 9, non-muscle
MYH11	myosin, heavy chain 11, smooth muscle
MYLK	myosin light chain kinase
NUBP1	nucleotide binding protein 1
NF2	neurofibromin 2 (merlin)
OLR1	oxidized low density lipoprotein receptor 1
P4HB	prolyl 4-hydroxylase subunit beta
FURIN	furin, paired basic amino acid cleaving enzyme
PCSK6	proprotein convertase subtilisin/kexin type 6
PAK1	p21 (RAC1) activated kinase 1
PAK2	p21 (RAC1) activated kinase 2
PAM	peptidylglycine alpha-amidating monooxygenase
PARK2	parkin RBR E3 ubiquitin protein ligase
PCSK1	proprotein convertase subtilisin/kexin type 1
PCSK5	proprotein convertase subtilisin/kexin type 5
CDK17	cyclin-dependent kinase 17
PDPK1	3-phosphoinositide dependent protein kinase 1
PEPD	peptidase D
PIGH	phosphatidylinositol glycan anchor biosynthesis class H
PIM1	Pim-1 proto-oncogene, serine/threonine kinase
PLAT	plasminogen activator, tissue type
PLOD2	procollagen-lysine,2-oxoglutarate 5-dioxygenase 2
PML	promyelocytic leukemia
PPM1B	protein phosphatase, Mg2+/Mn2+ dependent 1B
PPM1G	protein phosphatase, Mg2+/Mn2+ dependent 1G
PPP1CA	protein phosphatase 1 catalytic subunit alpha
PPP2R2A	protein phosphatase 2 regulatory subunit Balpha
PPP2R3A	protein phosphatase 2 regulatory subunit B''alpha
PPP2R5C	protein phosphatase 2 regulatory subunit B'gamma
PPT1	palmitoyl-protein thioesterase 1
PREP	prolyl endopeptidase
SRGN	serglycin
PRKAA1	protein kinase AMP-activated catalytic subunit alpha 1
PRKCB	protein kinase C beta
PRKCD	protein kinase C delta
PRKCE	protein kinase C epsilon
PRKCH	protein kinase C eta
PKN1	protein kinase N1
PKN2	protein kinase N2
PRKCZ	protein kinase C zeta
PRKDC	protein kinase, DNA-activated, catalytic polypeptide

PRKG1	protein kinase, cGMP-dependent, type I
PRKG2	protein kinase, cGMP-dependent, type II
MAPK8	mitogen-activated protein kinase 8
MAP2K6	mitogen-activated protein kinase kinase 6
PRLR	prolactin receptor
PTEN	phosphatase and tensin homolog
PTK6	protein tyrosine kinase 6
QSOX1	quiescin sulfhydryl oxidase 1
PTPN3	protein tyrosine phosphatase, non-receptor type 3
PTPN4	protein tyrosine phosphatase, non-receptor type 4
PTPN6	protein tyrosine phosphatase, non-receptor type 6
PTPN7	protein tyrosine phosphatase, non-receptor type 7
PTPN12	protein tyrosine phosphatase, non-receptor type 12
PTPN14	protein tyrosine phosphatase, non-receptor type 14
PTPRB	protein tyrosine phosphatase, receptor type B
PTPRC	protein tyrosine phosphatase, receptor type C
PTPRD	protein tyrosine phosphatase, receptor type D
PTPRE	protein tyrosine phosphatase, receptor type E
PTPRH	protein tyrosine phosphatase, receptor type H
PTPRJ	protein tyrosine phosphatase, receptor type J
PTPRN2	protein tyrosine phosphatase, receptor type N2
PTPRO	protein tyrosine phosphatase, receptor type O
PTPRR	protein tyrosine phosphatase, receptor type R
PXN	paxillin
RAC1	ras-related C3 botulinum toxin substrate 1 (rho family, small GTP binding protein Rac1)
REN	renin
RPL3	ribosomal protein L3
RPL3L	ribosomal protein L3 like
RPL7	ribosomal protein L7
RPL7A	ribosomal protein L7a
RPL18A	ribosomal protein L18a
RPL23A	ribosomal protein L23a
MRPL23	mitochondrial ribosomal protein L23
RPL24	ribosomal protein L24
RPL26	ribosomal protein L26
RPN1	ribophorin I
RPS2	ribosomal protein S2
RPS6KB2	ribosomal protein S6 kinase B2
RPS11	ribosomal protein S11
RRBP1	ribosome binding protein 1
CCL2	C-C motif chemokine ligand 2
CCL11	C-C motif chemokine ligand 11
SFTPD	surfactant protein D
SGK1	serum/glucocorticoid regulated kinase 1
SCG5	secretogranin V
SGSH	N-sulfoglucosamine sulfohydrolase
SHH	sonic hedgehog
SIAH2	siah E3 ubiquitin protein ligase 2
SPG7	SPG7, paraplegin matrix AAA peptidase subunit
SPRR1A	small proline rich protein 1A
SRC	SRC proto-oncogene, non-receptor tyrosine kinase
SRP72	signal recognition particle 72kDa
SRPK1	SRSF protein kinase 1
SRPK2	SRSF protein kinase 2
ST14	suppression of tumorigenicity 14
NEK4	NIMA related kinase 4
STK10	serine/threonine kinase 10

SYT1	synaptotagmin 1
TAPBP	TAP binding protein (tapasin)
TBCA	tubulin folding cofactor A
TBCD	tubulin folding cofactor D
TBCE	tubulin folding cofactor E
TGFBR1	transforming growth factor beta receptor 1
TGM3	transglutaminase 3
TJP1	tight junction protein 1
TLR1	toll like receptor 1
TLR3	toll like receptor 3
TLR4	toll like receptor 4
TM4SF4	transmembrane 4 L six family member 4
TSPAN4	tetraspanin 4
TNP1	transition protein 1
TNXB	tenascin XB
TP53	tumor protein p53
TPP2	tripeptidyl peptidase 2
TRAF1	TNF receptor associated factor 1
TRAF6	TNF receptor associated factor 6
TTC1	tetratricopeptide repeat domain 1
TTN	titin
UBB	ubiquitin B
UBE2D1	ubiquitin conjugating enzyme E2 D1
UBE2D3	ubiquitin conjugating enzyme E2 D3
UBE2E1	ubiquitin conjugating enzyme E2 E1
UBE2E2	ubiquitin conjugating enzyme E2 E2
UBE2G1	ubiquitin conjugating enzyme E2 G1
UBE2G2	ubiquitin conjugating enzyme E2 G2
UBE2H	ubiquitin conjugating enzyme E2 H
UBE2I	ubiquitin conjugating enzyme E2 I
UBE2L3	ubiquitin conjugating enzyme E2 L3
UBE2N	ubiquitin conjugating enzyme E2 N
UBE2V2	ubiquitin conjugating enzyme E2 V2
UBE3A	ubiquitin protein ligase E3A
VCL	vinculin
VHL	von Hippel-Lindau tumor suppressor
VRK2	vaccinia related kinase 2
VWF	von Willebrand factor
WIPF1	WAS/WASL interacting protein family member 1
EIF4H	eukaryotic translation initiation factor 4H
XPNPEP1	X-prolyl aminopeptidase (aminopeptidase P) 1, soluble
USP7	ubiquitin specific peptidase 7 (herpes virus-associated)
ST8SIA4	ST8 alpha-N-acetyl-neuraminide alpha-2,8-sialyltransferase 4
EPM2A	epilepsy, progressive myoclonus type 2A, Lafora disease (laforin)
KAT6A	lysine acetyltransferase 6A
NR4A3	nuclear receptor subfamily 4 group A member 3
CDK2AP1	cyclin-dependent kinase 2 associated protein 1
ST8SIA2	ST8 alpha-N-acetyl-neuraminide alpha-2,8-sialyltransferase 2
MKKS	McKusick-Kaufman syndrome
CHAF1B	chromatin assembly factor 1 subunit B
PICALM	phosphatidylinositol binding clathrin assembly protein
ULK1	unc-51 like autophagy activating kinase 1
LTBP4	latent transforming growth factor beta binding protein 4
NCK2	NCK adaptor protein 2
DYRK3	dual specificity tyrosine phosphorylation regulated kinase 3
TTF2	transcription termination factor, RNA polymerase II
TPST1	tyrosylprotein sulfotransferase 1
FKBP6	FK506 binding protein 6

CDC42BPA	CDC42 binding protein kinase alpha
DGKD	diacylglycerol kinase delta
CPZ	carboxypeptidase Z
TCAP	titin-cap
MKNK1	MAP kinase interacting serine/threonine kinase 1
SCARF1	scavenger receptor class F member 1
TP63	tumor protein p63
TNKS	tankyrase
EIF3A	eukaryotic translation initiation factor 3 subunit A
EIF3C	eukaryotic translation initiation factor 3 subunit C
EIF3F	eukaryotic translation initiation factor 3 subunit F
EIF3G	eukaryotic translation initiation factor 3 subunit G
EIF3H	eukaryotic translation initiation factor 3 subunit H
EIF4G3	eukaryotic translation initiation factor 4 gamma 3
B3GALT4	beta-1,3-galactosyltransferase 4
B3GALNT1	beta-1,3-N-acetylgalactosaminyltransferase 1 (globoside blood group)
MBTPS1	membrane bound transcription factor peptidase, site 1
CDKL1	cyclin dependent kinase like 1
DPM2	dolichyl-phosphate mannosyltransferase polypeptide 2, regulatory subunit
EIF2B3	eukaryotic translation initiation factor 2B subunit gamma
PRPF4B	pre-mRNA processing factor 4B
ENDOU	endonuclease, poly(U) specific
SKAP2	src kinase associated phosphoprotein 2
BTRC	beta-transducin repeat containing E3 ubiquitin protein ligase
WASL	Wiskott-Aldrich syndrome like
PLOD3	procollagen-lysine,2-oxoglutarate 5-dioxygenase 3
RPS6KA4	ribosomal protein S6 kinase A4
KALRN	kalirin, RhoGEF kinase
BRSK2	BR serine/threonine kinase 2
TM4SF5	transmembrane 4 L six family member 5
UBA3	ubiquitin like modifier activating enzyme 3
SLC7A7	solute carrier family 7 member 7
SLC7A6	solute carrier family 7 member 6
MAP3K6	mitogen-activated protein kinase kinase kinase 6
MTMR7	myotubularin related protein 7
HGS	hepatocyte growth factor-regulated tyrosine kinase substrate
DYRK1B	dual specificity tyrosine phosphorylation regulated kinase 1B
CTDP1	CTD phosphatase subunit 1
MAP3K13	mitogen-activated protein kinase kinase kinase 13
ZW10	zw10 kinetochore protein
DCLK1	doublecortin like kinase 1
GCNT3	glucosaminyl (N-acetyl) transferase 3, mucin type
RPS6KA5	ribosomal protein S6 kinase A5
STK17B	serine/threonine kinase 17b
STK17A	serine/threonine kinase 17a
TGM5	transglutaminase 5
VAMP3	vesicle associated membrane protein 3
SLC9A3R2	SLC9A3 regulator 2
SLC9A3R1	SLC9A3 regulator 1
HS6ST1	heparan sulfate 6-O-sulfotransferase 1
NMT2	N-myristoyltransferase 2
MAP4K4	mitogen-activated protein kinase kinase kinase kinase 4
ROCK2	Rho associated coiled-coil containing protein kinase 2
ADAMTS4	ADAM metallopeptidase with thrombospondin type 1 motif 4
MPDU1	mannose-P-dolichol utilization defect 1
GMFG	glia maturation factor gamma
CDC42BPB	CDC42 binding protein kinase beta
RNF14	ring finger protein 14

ISG15	ISG15 ubiquitin-like modifier
IKBKE	inhibitor of kappa light polypeptide gene enhancer in B-cells, kinase epsilon
KDM4A	lysine demethylase 4A
UBE3C	ubiquitin protein ligase E3C
EDEM1	ER degradation enhancing alpha-mannosidase like protein 1
ECE2	endothelin converting enzyme 2
TLK1	tousled like kinase 1
OXSR1	oxidative stress responsive 1
HS3ST3B1	heparan sulfate-glucosamine 3-sulfotransferase 3B1
AKT3	AKT serine/threonine kinase 3
RANBP9	RAN binding protein 9
DNAJB6	DnaJ heat shock protein family (Hsp40) member B6
PTPRU	protein tyrosine phosphatase, receptor type U
UST	uronyl 2-sulfotransferase
HIPK3	homeodomain interacting protein kinase 3
AASS	aminoadipate-semialdehyde synthase
CHST4	carbohydrate sulfotransferase 4
TNK2	tyrosine kinase non receptor 2
TRIB1	tribbles pseudokinase 1
RIDA	reactive intermediate imine deaminase A homolog
APC2	adenomatosis polyposis coli 2
TLR6	toll like receptor 6
NOD1	nucleotide binding oligomerization domain containing 1
ST3GAL6	ST3 beta-galactoside alpha-2,3-sialyltransferase 6
TESK2	testis-specific kinase 2
PPIH	peptidylprolyl isomerase H
CARM1	coactivator associated arginine methyltransferase 1
PITRM1	pitrilysin metallopeptidase 1
ATG7	autophagy related 7
IGF2BP2	insulin like growth factor 2 mRNA binding protein 2
CAMKK2	calcium/calmodulin-dependent protein kinase kinase 2
FARS2	phenylalanyl-tRNA synthetase 2, mitochondrial
CORIN	corin, serine peptidase
MAP3K2	mitogen-activated protein kinase kinase kinase 2
NEK6	NIMA related kinase 6
SEPT9	septin 9
WASF3	WAS protein family member 3
TUBGCP2	tubulin gamma complex associated protein 2
CLPX	caseinolytic mitochondrial matrix peptidase chaperone subunit
HPSE	heparanase
PPARGC1A	PPARG coactivator 1 alpha
MAN1A2	mannosidase alpha class 1A member 2
PDIA5	protein disulfide isomerase family A member 5
METAP2	methionyl aminopeptidase 2
ADRM1	adhesion regulating molecule 1
WWP2	WW domain containing E3 ubiquitin protein ligase 2
LECT1	leukocyte cell derived chemotaxin 1
UBE2C	ubiquitin conjugating enzyme E2 C
TPPP	tubulin polymerization promoting protein
PTPRT	protein tyrosine phosphatase, receptor type T
FAF1	Fas associated factor 1
MAP4K5	mitogen-activated protein kinase kinase kinase kinase 5
MAP4K1	mitogen-activated protein kinase kinase kinase kinase 1
IRAK3	interleukin 1 receptor associated kinase 3
DUSP10	dual specificity phosphatase 10
MGAT4B	mannosyl (alpha-1,3-)-glycoprotein beta-1,4-N-acetylglucosaminyltransferase, isozyme B
MGAT4A	mannosyl (alpha-1,3-)-glycoprotein beta-1,4-N-

	acetylglucosaminyltransferase, isozyme A
STK38	serine/threonine kinase 38
LMTK2	lemur tyrosine kinase 2
TRAK1	trafficking kinesin protein 1
MAPRE1	microtubule associated protein RP/EB family member 1
KIFAP3	kinesin associated protein 3
ATF6	activating transcription factor 6
MAST1	microtubule associated serine/threonine kinase 1
STK38L	serine/threonine kinase 38 like
SAMD4A	sterile alpha motif domain containing 4A
SMG1	SMG1, nonsense mediated mRNA decay associated PI3K related kinase
ERC1	ELKS/RAB6-interacting/CAST family member 1
ATG4B	autophagy related 4B cysteine peptidase
CLASP1	cytoplasmic linker associated protein 1
SIK3	SIK family kinase 3
SF3B3	splicing factor 3b subunit 3
ICMT	isoprenylcysteine carboxyl methyltransferase
KAT6B	lysine acetyltransferase 6B
CLDN14	claudin 14
CD2AP	CD2-associated protein
BACE1	beta-secretase 1
CBLC	Cbl proto-oncogene C
PRKD3	protein kinase D3
AIPL1	aryl hydrocarbon receptor interacting protein like 1
FBXO7	F-box protein 7
QPCT	glutaminyl-peptide cyclotransferase
GLCE	glucuronic acid epimerase
FBXW2	F-box and WD repeat domain containing 2
FBXL6	F-box and leucine-rich repeat protein 6
FBXO22	F-box protein 22
FBXO3	F-box protein 3
LATS2	large tumor suppressor kinase 2
EIF2AK1	eukaryotic translation initiation factor 2 alpha kinase 1
SERP1	stress-associated endoplasmic reticulum protein 1
TRIB2	tribbles pseudokinase 2
MYLIP	myosin regulatory light chain interacting protein
NPC1L1	NPC1 like intracellular cholesterol transporter 1
EEF2K	eukaryotic elongation factor 2 kinase
MINK1	misshapen like kinase 1
CHST11	carbohydrate (chondroitin 4) sulfotransferase 11
IL20	interleukin 20
NDUFA13	NADH:ubiquinone oxidoreductase subunit A13
YBX2	Y-box binding protein 2
SEPSECS	Sep (O-phosphoserine) tRNA:Sec (selenocysteine) tRNA synthase
A4GNT	alpha-1,4-N-acetylglucosaminyltransferase
ING4	inhibitor of growth family member 4
ABI3	ABI family member 3
CDKL3	cyclin dependent kinase like 3
GCNT4	glucosaminyl (N-acetyl) transferase 4, core 2
FZR1	fizzy/cell division cycle 20 related 1
TAOK3	TAO kinase 3
UBR5	ubiquitin protein ligase E3 component n-recognin 5
LIMA1	LIM domain and actin binding 1
PIGT	phosphatidylinositol glycan anchor biosynthesis class T
SUFU	SUFU negative regulator of hedgehog signaling
ERAP1	endoplasmic reticulum aminopeptidase 1
ZAK	sterile alpha motif and leucine zipper containing kinase AZK
GALNT7	polypeptide N-acetylgalactosaminyltransferase 7

RIPK4	receptor interacting serine/threonine kinase 4
TLR9	toll like receptor 9
SSH1	slingshot protein phosphatase 1
PRMT7	protein arginine methyltransferase 7
SNRK	SNF related kinase
PIGG	phosphatidylinositol glycan anchor biosynthesis class G
CLN6	ceroid-lipofuscinosis, neuronal 6, late infantile, variant
FBXW7	F-box and WD repeat domain containing 7
CHST12	carbohydrate (chondroitin 4) sulfotransferase 12
CDC42BPG	CDC42 binding protein kinase gamma
CDKN2AIP	CDKN2A interacting protein
PIGV	phosphatidylinositol glycan anchor biosynthesis class V
SEPT11	septin 11
CSGALNACT1	chondroitin sulfate N-acetylgalactosaminyltransferase 1
CAND1	cullin associated and neddylation dissociated 1
CENPJ	centromere protein J
ALG1	ALG1, chitobiosyldiphosphodolichol beta-mannosyltransferase
PARD3	par-3 family cell polarity regulator
DUSP22	dual specificity phosphatase 22
ADAMTS9	ADAM metallopeptidase with thrombospondin type 1 motif 9
GOPC	golgi-associated PDZ and coiled-coil motif containing
MAN1C1	mannosidase alpha class 1C member 1
SMURF1	SMAD specific E3 ubiquitin protein ligase 1
DOLPP1	dolichyldiphosphatase 1
PREX1	phosphatidylinositol-3,4,5-trisphosphate dependent Rac exchange factor 1
MARK4	microtubule affinity regulating kinase 4
SIGIRR	single immunoglobulin and toll-interleukin 1 receptor (TIR) domain
NECAB3	N-terminal EF-hand calcium binding protein 3
NOD2	nucleotide binding oligomerization domain containing 2
DNAJC1	DnaJ heat shock protein family (Hsp40) member C1
NSD1	nuclear receptor binding SET domain protein 1
CHST8	carbohydrate sulfotransferase 8
SMURF2	SMAD specific E3 ubiquitin protein ligase 2
PINK1	PTEN induced putative kinase 1
WNK1	WNK lysine deficient protein kinase 1
BOLL	boule homolog, RNA binding protein
MIS12	MIS12, kinetochore complex component
CARD14	caspase recruitment domain family member 14
ISOC2	isochorismatase domain containing 2
EHMT1	euchromatic histone lysine methyltransferase 1
NEK11	NIMA related kinase 11
HDAC11	histone deacetylase 11
NAA15	N(alpha)-acetyltransferase 15, NatA auxiliary subunit
NAA50	N(alpha)-acetyltransferase 50, NatE catalytic subunit
SCUBE1	signal peptide, CUB domain and EGF like domain containing 1
CD276	CD276 molecule
TLN2	talin 2
MEX3B	mex-3 RNA binding family member B
TRAF7	TNF receptor associated factor 7
ING5	inhibitor of growth family member 5
BRSK1	BR serine/threonine kinase 1
KIAA1804	mixed lineage kinase 4
PARD6B	par-6 family cell polarity regulator beta
C9orf3	chromosome 9 open reading frame 3
ATG4C	autophagy related 4C cysteine peptidase
ATG4D	autophagy related 4D cysteine peptidase
SSH2	slingshot protein phosphatase 2
HPS4	HPS4, biogenesis of lysosomal organelles complex 3 subunit 2

NLRP12	NLR family, pyrin domain containing 12
TP53RK	TP53 regulating kinase
NLRP3	NLR family, pyrin domain containing 3
IL22RA2	interleukin 22 receptor subunit alpha 2
UHMK1	U2AF homology motif (UHM) kinase 1
IL31RA	interleukin 31 receptor A
SIK1	salt inducible kinase 1
PPP4R2	protein phosphatase 4 regulatory subunit 2
TMPRSS6	transmembrane protease, serine 6
MUC20	mucin 20, cell surface associated
STT3B	STT3B, catalytic subunit of the oligosaccharyltransferase complex
HS3ST5	heparan sulfate-glucosamine 3-sulfotransferase 5
SCUBE3	signal peptide, CUB domain and EGF like domain containing 3
MAPK15	mitogen-activated protein kinase 15
MSRB3	methionine sulfoxide reductase B3
METAP1D	methionyl aminopeptidase type 1D (mitochondrial)
RNF144B	ring finger protein 144B
COX18	COX18 cytochrome c oxidase assembly factor
NDUFS7	NADH:ubiquinone oxidoreductase core subunit S7
AGRN	agrin
CCDC88C	coiled-coil domain containing 88C
EIF2AK4	eukaryotic translation initiation factor 2 alpha kinase 4
D2HGDH	D-2-hydroxyglutarate dehydrogenase
**Phosphorylation**	
**Gene Symbol**	**Name Gene**
ABL1	ABL proto-oncogene 1, non-receptor tyrosine kinase
ABL2	ABL proto-oncogene 2, non-receptor tyrosine kinase
ACVR1	activin A receptor type 1
ACVRL1	activin A receptor like type 1
AKT1	AKT serine/threonine kinase 1
BARD1	BRCA1 associated RING domain 1
CCND1	cyclin D1
BCR	BCR, RhoGEF and GTPase activating protein
BMP4	bone morphogenetic protein 4
BMPR1A	bone morphogenetic protein receptor type 1A
BMPR1B	bone morphogenetic protein receptor type 1B
BRAF	B-Raf proto-oncogene, serine/threonine kinase
CAMK2A	calcium/calmodulin dependent protein kinase II alpha
CAMK2B	calcium/calmodulin dependent protein kinase II beta
CCND2	cyclin D2
CCND3	cyclin D3
CD80	CD80 molecule
CD81	CD81 molecule
CDK11B	cyclin-dependent kinase 11B
CDKN1A	cyclin-dependent kinase inhibitor 1A
CDKN2C	cyclin-dependent kinase inhibitor 2C
PLK3	polo like kinase 3
MAP3K8	mitogen-activated protein kinase kinase kinase 8
CSNK1A1	casein kinase 1 alpha 1
CSNK1D	casein kinase 1 delta
CSNK1G2	casein kinase 1 gamma 2
CTBP1	C-terminal binding protein 1
DAPK1	death associated protein kinase 1
DAPK3	death-associated protein kinase 3
DMPK	dystrophia myotonica protein kinase
DYRK1A	dual specificity tyrosine phosphorylation regulated kinase 1A
EGFR	epidermal growth factor receptor
MARK2	microtubule affinity regulating kinase 2

EPHA8	EPH receptor A8
EPHB2	EPH receptor B2
ERBB2	erb-b2 receptor tyrosine kinase 2
ERBB3	erb-b2 receptor tyrosine kinase 3
ERG	v-ets avian erythroblastosis virus E26 oncogene homolog
ERN1	endoplasmic reticulum to nucleus signaling 1
F2	coagulation factor II, thrombin
F2R	coagulation factor II thrombin receptor
FGR	FGR proto-oncogene, Src family tyrosine kinase
FRK	fyn related Src family tyrosine kinase
MTOR	mechanistic target of rapamycin
FYB	FYN binding protein
MKNK2	MAP kinase interacting serine/threonine kinase 2
GSK3B	glycogen synthase kinase 3 beta
HCLS1	hematopoietic cell-specific Lyn substrate 1
IGF1R	insulin like growth factor 1 receptor
IL12A	interleukin 12A
INHBA	inhibin beta A
INSR	insulin receptor
IRAK2	interleukin 1 receptor associated kinase 2
ITGB2	integrin subunit beta 2
JAK2	Janus kinase 2
JAK3	Janus kinase 3
LCK	LCK proto-oncogene, Src family tyrosine kinase
LIMK1	LIM domain kinase 1
LYN	LYN proto-oncogene, Src family tyrosine kinase
MATK	megakaryocyte-associated tyrosine kinase
MCM7	minichromosome maintenance complex component 7
MAP3K3	mitogen-activated protein kinase kinase kinase 3
MAP3K9	mitogen-activated protein kinase kinase kinase 9
MAP3K11	mitogen-activated protein kinase kinase kinase 11
MYLK	myosin light chain kinase
NF2	neurofibromin 2 (merlin)
PAK1	p21 (RAC1) activated kinase 1
PAK2	p21 (RAC1) activated kinase 2
CDK17	cyclin-dependent kinase 17
PDPK1	3-phosphoinositide dependent protein kinase 1
PIM1	Pim-1 proto-oncogene, serine/threonine kinase
PIK3R1	phosphoinositide-3-kinase regulatory subunit 1
PRKAA1	protein kinase AMP-activated catalytic subunit alpha 1
PRKCB	protein kinase C beta
PRKCD	protein kinase C delta
PRKCE	protein kinase C epsilon
PRKCH	protein kinase C eta
PKN1	protein kinase N1
PKN2	protein kinase N2
PRKCZ	protein kinase C zeta
PRKG1	protein kinase, cGMP-dependent, type I
PRKG2	protein kinase, cGMP-dependent, type II
MAPK8	mitogen-activated protein kinase 8
MAP2K6	mitogen-activated protein kinase kinase 6
PTK6	protein tyrosine kinase 6
RAF1	Raf-1 proto-oncogene, serine/threonine kinase
RET	ret proto-oncogene
CCL2	C-C motif chemokine ligand 2
CCL11	C-C motif chemokine ligand 11
SGK1	serum/glucocorticoid regulated kinase 1
SRP72	signal recognition particle 72kDa

SRPK1	SRSF protein kinase 1
SRPK2	SRSF protein kinase 2
NEK4	NIMA related kinase 4
STK10	serine/threonine kinase 10
TGFBR1	transforming growth factor beta receptor 1
TTN	titin
TXK	TXK tyrosine kinase
VRK2	vaccinia related kinase 2
NR4A3	nuclear receptor subfamily 4 group A member 3
CDK2AP1	cyclin-dependent kinase 2 associated protein 1
ULK1	unc-51 like autophagy activating kinase 1
DYRK3	dual specificity tyrosine phosphorylation regulated kinase 3
DYRK2	dual specificity tyrosine phosphorylation regulated kinase 2
CDC42BPA	CDC42 binding protein kinase alpha
MKNK1	MAP kinase interacting serine/threonine kinase 1
TNKS	tankyrase
CDKL1	cyclin dependent kinase like 1
PRPF4B	pre-mRNA processing factor 4B
RPS6KA4	ribosomal protein S6 kinase A4
KALRN	kalirin, RhoGEF kinase
BRSK2	BR serine/threonine kinase 2
MAP3K6	mitogen-activated protein kinase kinase kinase 6
DYRK1B	dual specificity tyrosine phosphorylation regulated kinase 1B
MAP3K13	mitogen-activated protein kinase kinase kinase 13
DCLK1	doublecortin like kinase 1
RPS6KA5	ribosomal protein S6 kinase A5
STK17B	serine/threonine kinase 17b
STK17A	serine/threonine kinase 17a
MAP4K4	mitogen-activated protein kinase kinase kinase kinase 4
ROCK2	Rho associated coiled-coil containing protein kinase 2
GMFG	glia maturation factor gamma
CDC42BPB	CDC42 binding protein kinase beta
IKBKE	inhibitor of kappa light polypeptide gene enhancer in B-cells, kinase epsilon
IP6K1	inositol hexakisphosphate kinase 1
TLK1	tousled like kinase 1
OXSR1	oxidative stress responsive 1
AKT3	AKT serine/threonine kinase 3
HIPK3	homeodomain interacting protein kinase 3
TNK2	tyrosine kinase non receptor 2
TRIB1	tribbles pseudokinase 1
BCKDK	branched chain ketoacid dehydrogenase kinase
TESK2	testis-specific kinase 2
MERTK	MER proto-oncogene, tyrosine kinase
CAMKK2	calcium/calmodulin-dependent protein kinase kinase 2
MAP3K2	mitogen-activated protein kinase kinase kinase 2
NEK6	NIMA related kinase 6
GLMN	glomulin, FKBP associated protein
MAP4K5	mitogen-activated protein kinase kinase kinase kinase 5
MAP4K1	mitogen-activated protein kinase kinase kinase kinase 1
IRAK3	interleukin 1 receptor associated kinase 3
PNKP	polynucleotide kinase 3'-phosphatase
STK38	serine/threonine kinase 38
LMTK2	lemur tyrosine kinase 2
MAST1	microtubule associated serine/threonine kinase 1
STK38L	serine/threonine kinase 38 like
SMG1	SMG1, nonsense mediated mRNA decay associated PI3K related kinase
ERC1	ELKS/RAB6-interacting/CAST family member 1
MAST2	microtubule associated serine/threonine kinase 2

SIK3	SIK family kinase 3
DAPK2	death-associated protein kinase 2
CBLC	Cbl proto-oncogene C
PRKD3	protein kinase D3
LATS2	large tumor suppressor kinase 2
EIF2AK1	eukaryotic translation initiation factor 2 alpha kinase 1
STK36	serine/threonine kinase 36
TRIB2	tribbles pseudokinase 2
MINK1	misshapen like kinase 1
IL20	interleukin 20
ABI3	ABI family member 3
TAOK3	TAO kinase 3
IP6K2	inositol hexakisphosphate kinase 2
ZAK	sterile alpha motif and leucine zipper containing kinase AZK
RIPK4	receptor interacting serine/threonine kinase 4
TOLLIP	toll interacting protein
SNRK	SNF related kinase
PXK	PX domain containing serine/threonine kinase like
CDC42BPG	CDC42 binding protein kinase gamma
MARK4	microtubule affinity regulating kinase 4
PINK1	PTEN induced putative kinase 1
WNK1	WNK lysine deficient protein kinase 1
WNK2	WNK lysine deficient protein kinase 2
CARD14	caspase recruitment domain family member 14
NEK11	NIMA related kinase 11
MEX3B	mex-3 RNA binding family member B
BRSK1	BR serine/threonine kinase 1
KIAA1804	mixed lineage kinase 4
NLRP12	NLR family, pyrin domain containing 12
TP53RK	TP53 regulating kinase
IL22RA2	interleukin 22 receptor subunit alpha 2
UHMK1	U2AF homology motif (UHM) kinase 1
IL31RA	interleukin 31 receptor A
SIK1	salt inducible kinase 1
MAPK15	mitogen-activated protein kinase 15
CCDC88C	coiled-coil domain containing 88C
EIF2AK4	eukaryotic translation initiation factor 2 alpha kinase 4
**Intracellular Signaling cascade**	
**Gene Symbol**	**Name Gene**
ABCA1	ATP binding cassette subfamily A member 1
ABL1	ABL proto-oncogene 1, non-receptor tyrosine kinase
ABR	active BCR-related
ADCY7	adenylate cyclase 7
ADORA2B	adenosine A2b receptor
ADORA3	adenosine A3 receptor
ADRA1B	adrenoceptor alpha 1B
ADRA2C	adrenoceptor alpha 2C
AGTR1	angiotensin II receptor type 1
AKT1	AKT serine/threonine kinase 1
AMBP	alpha-1-microglobulin/bikunin precursor
FASLG	Fas ligand
RHOB	ras homolog family member B
ARHGDIA	Rho GDP dissociation inhibitor alpha
ARHGDIB	Rho GDP dissociation inhibitor beta
RHOH	ras homolog family member H
AVPR1A	arginine vasopressin receptor 1A
AZU1	azurocidin 1
BAD	BCL2 associated agonist of cell death

BID	BH3 interacting domain death agonist
BLK	BLK proto-oncogene, Src family tyrosine kinase
BNIP3	BCL2/adenovirus E1B 19kDa interacting protein 3
BRCA1	breast cancer 1
BST2	bone marrow stromal cell antigen 2
CAPS	calcyphosine
CASP3	caspase 3
CASP8	caspase 8
CASP9	caspase 9
CCKAR	cholecystokinin A receptor
CCKBR	cholecystokinin B receptor
CD81	CD81 molecule
CDH13	cadherin 13
FOXN3	forkhead box N3
CHRM1	cholinergic receptor muscarinic 1
CHRM2	cholinergic receptor muscarinic 2
CHRM5	cholinergic receptor muscarinic 5
CIDEA	cell death-inducing DFFA-like effector a
CCR3	C-C motif chemokine receptor 3
LTB4R	leukotriene B4 receptor
CNR1	cannabinoid receptor 1 (brain)
CNR2	cannabinoid receptor 2
COL4A3	collagen type IV alpha 3
CRHR1	corticotropin releasing hormone receptor 1
CRHR2	corticotropin releasing hormone receptor 2
DGKA	diacylglycerol kinase alpha
DAPK1	death associated protein kinase 1
DAPK3	death-associated protein kinase 3
DAXX	death-domain associated protein
DMPK	dystrophia myotonica protein kinase
DOCK1	dedicator of cytokinesis 1
DRD1	dopamine receptor D1
DRD5	dopamine receptor D5
DUSP2	dual specificity phosphatase 2
DUSP4	dual specificity phosphatase 4
DUSP6	dual specificity phosphatase 6
ECM1	extracellular matrix protein 1
EDN2	endothelin 2
EDNRA	endothelin receptor type A
EDNRB	endothelin receptor type B
EGFR	epidermal growth factor receptor
MARK2	microtubule affinity regulating kinase 2
ERN1	endoplasmic reticulum to nucleus signaling 1
F2	coagulation factor II, thrombin
F2R	coagulation factor II thrombin receptor
FGFR1	fibroblast growth factor receptor 1
FGFR3	fibroblast growth factor receptor 3
FYB	FYN binding protein
FYN	FYN proto-oncogene, Src family tyrosine kinase
IFI6	interferon alpha inducible protein 6
GABBR1	gamma-aminobutyric acid type B receptor subunit 1
GAP43	growth associated protein 43
GHRH	growth hormone releasing hormone
GIPR	gastric inhibitory polypeptide receptor
GJA1	gap junction protein alpha 1
GLP1R	glucagon like peptide 1 receptor
GML	glycosylphosphatidylinositol anchored molecule like
GNA15	G protein subunit alpha 15

GNAS	GNAS complex locus
GNB1	G protein subunit beta 1
SFN	stratifin
XCR1	X-C motif chemokine receptor 1
MCHR1	melanin concentrating hormone receptor 1
GRK5	G protein-coupled receptor kinase 5
MKNK2	MAP kinase interacting serine/threonine kinase 2
GPS1	G protein pathway suppressor 1
GRB2	growth factor receptor bound protein 2
GRM2	glutamate metabotropic receptor 2
GRM3	glutamate metabotropic receptor 3
GRM4	glutamate metabotropic receptor 4
GRM8	glutamate metabotropic receptor 8
GSK3B	glycogen synthase kinase 3 beta
GZMA	granzyme A
GZMB	granzyme B
HCLS1	hematopoietic cell-specific Lyn substrate 1
HIP1	huntingtin interacting protein 1
HRH1	histamine receptor H1
HRH2	histamine receptor H2
HTR1B	5-hydroxytryptamine receptor 1B
HTR1F	5-hydroxytryptamine receptor 1F
HTR6	5-hydroxytryptamine receptor 6
IFNAR2	interferon alpha and beta receptor subunit 2
IGF1	insulin like growth factor 1
CXCR2	C-X-C motif chemokine receptor 2
IL12A	interleukin 12A
IRAK2	interleukin 1 receptor associated kinase 2
JAK2	Janus kinase 2
STMN1	stathmin 1
LCK	LCK proto-oncogene, Src family tyrosine kinase
LGALS1	galectin 1
LGALS9	galectin 9
LIMK1	LIM domain kinase 1
LTBR	lymphotoxin beta receptor
LYN	LYN proto-oncogene, Src family tyrosine kinase
SMAD3	SMAD family member 3
MC1R	melanocortin 1 receptor
MC4R	melanocortin 4 receptor
MCL1	myeloid cell leukemia 1
MDFI	MyoD family inhibitor
MAP3K3	mitogen-activated protein kinase kinase kinase 3
MAP3K4	mitogen-activated protein kinase kinase kinase 4
MAP3K5	mitogen-activated protein kinase kinase kinase 5
MAP3K9	mitogen-activated protein kinase kinase kinase 9
MAP3K11	mitogen-activated protein kinase kinase kinase 11
MTNR1A	melatonin receptor 1A
GADD45B	growth arrest and DNA damage inducible beta
MYO9B	myosin IXB
NF1	neurofibromin 1
NF2	neurofibromin 2 (merlin)
NFATC1	nuclear factor of activated T-cells 1
NPY	neuropeptide Y
OPRD1	opioid receptor delta 1
OPRL1	opioid related nociceptin receptor 1
OPRM1	opioid receptor mu 1
P2RY1	purinergic receptor P2Y1
P2RY2	purinergic receptor P2Y2

PAK1	p21 (RAC1) activated kinase 1
PDK1	pyruvate dehydrogenase kinase 1
PDPK1	3-phosphoinositide dependent protein kinase 1
PLCB2	phospholipase C beta 2
PLD1	phospholipase D1
PML	promyelocytic leukemia
PPM1A	protein phosphatase, Mg2+/Mn2+ dependent 1A
PPT1	palmitoyl-protein thioesterase 1
PRKAR2A	protein kinase cAMP-dependent type II regulatory subunit alpha
PKN1	protein kinase N1
MAPK8	mitogen-activated protein kinase 8
MAPK11	mitogen-activated protein kinase 11
MAPK10	mitogen-activated protein kinase 10
PTAFR	platelet activating factor receptor
PTEN	phosphatase and tensin homolog
PTGER4	prostaglandin E receptor 4
RAC1	ras-related C3 botulinum toxin substrate 1 (rho family, small GTP binding protein Rac1)
RAD9A	RAD9 checkpoint clamp component A
RELA	RELA proto-oncogene, NF-kB subunit
REN	renin
RGS3	regulator of G-protein signaling 3
RPS6KA2	ribosomal protein S6 kinase A2
RRAD	RRAD, Ras related glycolysis inhibitor and calcium channel regulator
RREB1	ras responsive element binding protein 1
RTKN	rhotekin
CCL2	C-C motif chemokine ligand 2
SECTM1	secreted and transmembrane 1
MAP2K4	mitogen-activated protein kinase kinase 4
SIAH2	siah E3 ubiquitin protein ligase 2
SOD1	superoxide dismutase 1, soluble
SOS1	SOS Ras/Rac guanine nucleotide exchange factor 1
SRC	SRC proto-oncogene, non-receptor tyrosine kinase
SRPK1	SRSF protein kinase 1
SRPK2	SRSF protein kinase 2
SSTR4	somatostatin receptor 4
SSTR5	somatostatin receptor 5
STAT3	signal transducer and activator of transcription 3
STAT5A	signal transducer and activator of transcription 5A
TACR1	tachykinin receptor 1
MAP3K7	mitogen-activated protein kinase kinase kinase 7
TNFRSF1A	tumor necrosis factor receptor superfamily member 1A
TP53	tumor protein p53
TP73	tumor protein p73
TPD52L1	tumor protein D52-like 1
TRAF5	TNF receptor associated factor 5
TRAF6	TNF receptor associated factor 6
TRIP6	thyroid hormone receptor interactor 6
TSHR	thyroid stimulating hormone receptor
UBE2N	ubiquitin conjugating enzyme E2 N
VDAC1	voltage dependent anion channel 1
VIPR1	vasoactive intestinal peptide receptor 1
YWHAE	tyrosine 3-monooxygenase/tryptophan 5-monooxygenase activation protein epsilon
SCG2	secretogranin II
NR4A3	nuclear receptor subfamily 4 group A member 3
ARID1A	AT-rich interaction domain 1A
DYRK2	dual specificity tyrosine phosphorylation regulated kinase 2

CDC42BPA	CDC42 binding protein kinase alpha
MKNK1	MAP kinase interacting serine/threonine kinase 1
S1PR4	sphingosine-1-phosphate receptor 4
TRADD	TNFRSF1A associated via death domain
RIPK1	receptor interacting serine/threonine kinase 1
RIPK2	receptor interacting serine/threonine kinase 2
SOCS2	suppressor of cytokine signaling 2
CFLAR	CASP8 and FADD like apoptosis regulator
RPS6KA4	ribosomal protein S6 kinase A4
KALRN	kalirin, RhoGEF kinase
F2RL3	F2R like thrombin/trypsin receptor 3
MAP3K6	mitogen-activated protein kinase kinase kinase 6
HGS	hepatocyte growth factor-regulated tyrosine kinase substrate
LPAR2	lysophosphatidic acid receptor 2
MAP3K13	mitogen-activated protein kinase kinase kinase 13
VAPA	VAMP associated protein A
RPS6KA5	ribosomal protein S6 kinase A5
STK17B	serine/threonine kinase 17b
STK17A	serine/threonine kinase 17a
S1PR2	sphingosine-1-phosphate receptor 2
GRAP2	GRB2-related adaptor protein 2
ARHGAP29	Rho GTPase activating protein 29
MAP4K4	mitogen-activated protein kinase kinase kinase kinase 4
AKAP7	A-kinase anchoring protein 7
SH3BP5	SH3 domain binding protein 5
LITAF	lipopolysaccharide induced TNF factor
CDC42BPB	CDC42 binding protein kinase beta
CARTPT	CART prepropeptide
IKBKE	inhibitor of kappa light polypeptide gene enhancer in B-cells, kinase epsilon
ELMO1	engulfment and cell motility 1
FARP2	FERM, ARH/RhoGEF and pleckstrin domain protein 2
MED24	mediator complex subunit 24
TLK1	tousled like kinase 1
MFN2	mitofusin 2
OXSR1	oxidative stress responsive 1
MED16	mediator complex subunit 16
SH2D3C	SH2 domain containing 3C
DNM1L	dynamin 1-like
WASF2	WAS protein family member 2
TNK2	tyrosine kinase non receptor 2
CNKSR1	connector enhancer of kinase suppressor of Ras 1
EFS	embryonal Fyn-associated substrate
TLR6	toll like receptor 6
TFG	TRK-fused gene
NOD1	nucleotide binding oligomerization domain containing 1
VAV3	vav guanine nucleotide exchange factor 3
TAB1	TGF-beta activated kinase 1/MAP3K7 binding protein 1
TRIM38	tripartite motif containing 38
CAP2	CAP, adenylate cyclase-associated protein, 2 (yeast)
CAP1	CAP, adenylate cyclase-associated protein 1 (yeast)
ARFGEF2	ADP ribosylation factor guanine nucleotide exchange factor 2
SH2B2	SH2B adaptor protein 2
CAMKK2	calcium/calmodulin-dependent protein kinase kinase 2
MAP3K2	mitogen-activated protein kinase kinase kinase 2
GRAP	GRB2-related adaptor protein
TRAF3IP2	TRAF3 interacting protein 2
PLK2	polo like kinase 2
NEK6	NIMA related kinase 6

RALBP1	ralA binding protein 1
FAF1	Fas associated factor 1
MAP4K5	mitogen-activated protein kinase kinase kinase kinase 5
MAP4K1	mitogen-activated protein kinase kinase kinase kinase 1
RASSF1	Ras association domain family member 1
AKAP11	A-kinase anchoring protein 11
DUSP10	dual specificity phosphatase 10
HRH3	histamine receptor H3
STK38	serine/threonine kinase 38
MRAS	muscle RAS oncogene homolog
CARD8	caspase recruitment domain family member 8
ATF6	activating transcription factor 6
SCAP	SREBF chaperone
MAST1	microtubule associated serine/threonine kinase 1
ACIN1	apoptotic chromatin condensation inducer 1
PLCH1	phospholipase C eta 1
STK38L	serine/threonine kinase 38 like
TNIK	TRAF2 and NCK interacting kinase
ERC1	ELKS/RAB6-interacting/CAST family member 1
TAB2	TGF-beta activated kinase 1/MAP3K7 binding protein 2
MAPK8IP3	mitogen-activated protein kinase 8 interacting protein 3
ZDHHC17	zinc finger DHHC-type containing 17
RBFOX2	RNA binding protein, fox-1 homolog 2
DDAH2	dimethylarginine dimethylaminohydrolase 2
LPAR3	lysophosphatidic acid receptor 3
DDAH1	dimethylarginine dimethylaminohydrolase 1
CARHSP1	calcium regulated heat stable protein 1
DAPK2	death-associated protein kinase 2
PRKD3	protein kinase D3
LATS2	large tumor suppressor kinase 2
ATP2C1	ATPase secretory pathway Ca2+ transporting 1
CECR2	CECR2, histone acetyl-lysine reader
DBNL	drebrin like
HIPK2	homeodomain interacting protein kinase 2
CARD10	caspase recruitment domain family member 10
MDFIC	MyoD family inhibitor domain containing
RHOD	ras homolog family member D
MINK1	misshapen like kinase 1
IL20	interleukin 20
NDUFA13	NADH:ubiquinone oxidoreductase subunit A13
SHISA5	shisa family member 5
GMIP	GEM interacting protein
TAOK3	TAO kinase 3
UBR5	ubiquitin protein ligase E3 component n-recognin 5
CXXC5	CXXC finger protein 5
MBIP	MAP3K12 binding inhibitory protein 1
STYXL1	serine/threonine/tyrosine interacting-like 1
NLK	nemo like kinase
ZAK	sterile alpha motif and leucine zipper containing kinase AZK
CYCS	cytochrome c, somatic
TREM1	triggering receptor expressed on myeloid cells 1
ZDHHC13	zinc finger DHHC-type containing 13
NDFIP2	Nedd4 family interacting protein 2
ARHGEF10L	Rho guanine nucleotide exchange factor 10 like
CDC42BPG	CDC42 binding protein kinase gamma
FGD6	FYVE, RhoGEF and PH domain containing 6
PAG1	phosphoprotein membrane anchor with glycosphingolipid microdomains 1
PARD3	par-3 family cell polarity regulator

LTB4R2	leukotriene B4 receptor 2
DUSP22	dual specificity phosphatase 22
OTUD7B	OTU deubiquitinase 7B
SLC44A2	solute carrier family 44 member 2
PLEKHG5	pleckstrin homology and RhoGEF domain containing G5
CALCOCO1	calcium binding and coiled-coil domain 1
NLRC4	NLR family, CARD domain containing 4
RRAGC	Ras related GTP binding C
NOD2	nucleotide binding oligomerization domain containing 2
CARD9	caspase recruitment domain family member 9
ARAP3	ArfGAP with RhoGAP domain, ankyrin repeat and PH domain 3
CLEC7A	C-type lectin domain family 7 member A
PINK1	PTEN induced putative kinase 1
WNK1	WNK lysine deficient protein kinase 1
WNK2	WNK lysine deficient protein kinase 2
DDX54	DEAD-box helicase 54
ADIPOR2	adiponectin receptor 2
NEK11	NIMA related kinase 11
VOPP1	vesicular, overexpressed in cancer, prosurvival protein 1
CCM2	CCM2 scaffolding protein
SLA2	Src-like-adaptor 2
TRAF7	TNF receptor associated factor 7
BRSK1	BR serine/threonine kinase 1
KIAA1804	mixed lineage kinase 4
DERL3	derlin 3
NLRP12	NLR family, pyrin domain containing 12
IL22RA2	interleukin 22 receptor subunit alpha 2
CLNK	cytokine dependent hematopoietic cell linker
ARAP1	ArfGAP with RhoGAP domain, ankyrin repeat and PH domain 1
FGD4	FYVE, RhoGEF and PH domain containing 4
EDARADD	EDAR-associated death domain
IL31RA	interleukin 31 receptor A
IRAK1BP1	interleukin 1 receptor associated kinase 1 binding protein 1
ABRA	actin binding Rho activating protein
MIB2	mindbomb E3 ubiquitin protein ligase 2
SIK1	salt inducible kinase 1
NFAM1	NFAT activating protein with ITAM motif 1
ROPN1B	rhophilin associated tail protein 1B
FGD5	FYVE, RhoGEF and PH domain containing 5
SPRED1	sprouty related EVH1 domain containing 1
NLRC3	NLR family, CARD domain containing 3
PIKFYVE	phosphoinositide kinase, FYVE-type zinc finger containing
SPRED2	sprouty related EVH1 domain containing 2
ARHGAP27	Rho GTPase activating protein 27
FGD2	FYVE, RhoGEF and PH domain containing 2
EPGN	epithelial mitogen
CRIPAK	cysteine rich PAK1 inhibitor
**Regulation of cell proliferation**	
**Gene Symbol**	**Name Gene**
ACVRL1	activin A receptor like type 1
AIF1	allograft inflammatory factor 1
ALOX15B	arachidonate 15-lipoxygenase, type B
BRCA1	breast cancer 1
BRCA2	breast cancer 2
BTC	betacellulin
CAPN1	calpain 1
CAPNS1	calpain small subunit 1
RUNX3	runt related transcription factor 3

CCKBR	cholecystokinin B receptor
CD3E	CD3e molecule
CD28	CD28 molecule
TNFRSF8	tumor necrosis factor receptor superfamily member 8
CD47	CD47 molecule
CD81	CD81 molecule
CDC25B	cell division cycle 25B
CDK6	cyclin-dependent kinase 6
CDKN2C	cyclin-dependent kinase inhibitor 2C
CDKN3	cyclin-dependent kinase inhibitor 3
CHRM1	cholinergic receptor muscarinic 1
CTBP1	C-terminal binding protein 1
CTBP2	C-terminal binding protein 2
CTF1	cardiotrophin 1
DDX11	DEAD/H-box helicase 11
EGFR	epidermal growth factor receptor
ERBB2	erb-b2 receptor tyrosine kinase 2
ETS1	ETS proto-oncogene 1, transcription factor
FABP6	fatty acid binding protein 6
FLT1	fms related tyrosine kinase 1
FLT4	fms related tyrosine kinase 4
FRK	fyn related Src family tyrosine kinase
GAS8	growth arrest specific 8
GML	glycosylphosphatidylinositol anchored molecule like
HCLS1	hematopoietic cell-specific Lyn substrate 1
HOXC10	homeobox C10
IGF1R	insulin like growth factor 1 receptor
IL1B	interleukin 1 beta
IL6	interleukin 6
CXCR2	C-X-C motif chemokine receptor 2
IL12RB2	interleukin 12 receptor subunit beta 2
IL18	interleukin 18
JAG2	jagged 2
KRT4	keratin 4
LAMC1	laminin subunit gamma 1
LRP5	LDL receptor related protein 5
LYN	LYN proto-oncogene, Src family tyrosine kinase
MATK	megakaryocyte-associated tyrosine kinase
MDM2	MDM2 proto-oncogene
MDM4	MDM4, p53 regulator
MNT	MAX network transcriptional repressor
MST1R	macrophage stimulating 1 receptor
NF1	neurofibromin 1
NF2	neurofibromin 2 (merlin)
NPM1	nucleophosmin (nucleolar phosphoprotein B23, numatrin)
OPRM1	opioid receptor mu 1
PAWR	pro-apoptotic WT1 regulator
PDGFA	platelet derived growth factor subunit A
PLG	plasminogen
POU3F2	POU class 3 homeobox 2
PTEN	phosphatase and tensin homolog
QSOX1	quiescin sulfhydryl oxidase 1
RARRES1	retinoic acid receptor responder 1
S100A11	S100 calcium binding protein A11
TSPAN31	tetraspanin 31
SFTPD	surfactant protein D
SLAMF1	signaling lymphocytic activation molecule family member 1
SSR1	signal sequence receptor subunit 1

SSTR4	somatostatin receptor 4
SSTR5	somatostatin receptor 5
TBX2	T-box 2
TBX3	T-box 3
TGFB1I1	transforming growth factor beta 1 induced transcript 1
TGFB2	transforming growth factor beta 2
TGFBR2	transforming growth factor beta receptor 2
KLF10	Kruppel-like factor 10
TM4SF4	transmembrane 4 L six family member 4
TNFSF4	tumor necrosis factor superfamily member 4
VEGFA	vascular endothelial growth factor A
VHL	von Hippel-Lindau tumor suppressor
VIPR1	vasoactive intestinal peptide receptor 1
BTG2	BTG family member 2
SCG2	secretogranin II
LST1	leukocyte specific transcript 1
NCK2	NCK adaptor protein 2
CUL4A	cullin 4A
CUL3	cullin 3
CUL2	cullin 2
CUL1	cullin 1
IFITM1	interferon induced transmembrane protein 1
FGF18	fibroblast growth factor 18
NRP1	neuropilin 1
HGS	hepatocyte growth factor-regulated tyrosine kinase substrate
DLG5	discs large homolog 5
S1PR2	sphingosine-1-phosphate receptor 2
KLF4	Kruppel-like factor 4 (gut)
TP53I11	tumor protein p53 inducible protein 11
FGFBP1	fibroblast growth factor binding protein 1
NAMPT	nicotinamide phosphoribosyltransferase
TOB1	transducer of ERBB2, 1
TCIRG1	T-cell immune regulator 1, ATPase H+ transporting V0 subunit a3
GPNMB	glycoprotein nmb
MXD4	MAX dimerization protein 4
ADAMTS8	ADAM metallopeptidase with thrombospondin type 1 motif 8
MYO16	myosin XVI
ICOSLG	inducible T-cell co-stimulator ligand
RBFOX2	RNA binding protein, fox-1 homolog 2
GNL3	G protein nucleolar 3
UTP20	UTP20, small subunit processome component
ING4	inhibitor of growth family member 4
ATP8A2	ATPase phospholipid transporting 8A2
AGGF1	angiogenic factor with G-patch and FHA domains 1
MARK4	microtubule affinity regulating kinase 4
IL21	interleukin 21
CD276	CD276 molecule
COL18A1	collagen type XVIII alpha 1
ING5	inhibitor of growth family member 5
SCIN	scinderin
IL31RA	interleukin 31 receptor A
EPGN	epithelial mitogen
LAMA1	laminin subunit alpha 1
ENPP7	ectonucleotide pyrophosphatase/phosphodiesterase 7
**Ectoderm development**	
**Gene Symbol**	**Name Gene**
ALOX12B	arachidonate 12-lipoxygenase, 12R type
ATP2A2	ATPase sarcoplasmic/endoplasmic reticulum Ca2+ transporting 2

BTD	biotinidase
COL1A1	collagen type I alpha 1
COL5A2	collagen type V alpha 2
COL7A1	collagen type VII alpha 1
CTGF	connective tissue growth factor
DHCR24	24-dehydrocholesterol reductase
DSP	desmoplakin
EMP1	epithelial membrane protein 1
STX2	syntaxin 2
ERCC3	excision repair cross-complementation group 3
EVPL	envoplakin
GJB5	gap junction protein beta 5
KRT1	keratin 1
KRT5	keratin 5
KRT6B	keratin 6B
KRT13	keratin 13
KRT15	keratin 15
KRT32	keratin 32
LAMA3	laminin subunit alpha 3
LAMB3	laminin subunit beta 3
LAMC2	laminin subunit gamma 2
PROX1	prospero homeobox 1
SPRR1A	small proline rich protein 1A
TFAP2A	transcription factor AP-2 alpha
TGFB2	transforming growth factor beta 2
TGM3	transglutaminase 3
UGCG	UDP-glucose ceramide glucosyltransferase
FOXN1	forkhead box N1
SCEL	sciellin
TGM5	transglutaminase 5
FST	follistatin
VAX2	ventral anterior homeobox 2
KLK5	kallikrein related peptidase 5
POU2F3	POU class 2 homeobox 3
ATP2C1	ATPase secretory pathway Ca2+ transporting 1
IL20	interleukin 20
ZBTB7B	zinc finger and BTB domain containing 7B
CALML5	calmodulin like 5
SMURF1	SMAD specific E3 ubiquitin protein ligase 1
**Neuron Differentiation**	
**Gene Symbol**	**Name Gene**
ATP2B2	ATPase plasma membrane Ca2+ transporting 2
DTX1	deltex 1
GDNF	glial cell derived neurotrophic factor
GLI2	GLI family zinc finger 2
LAMB1	laminin subunit beta 1
LMX1B	LIM homeobox transcription factor 1 beta
MAPT	microtubule associated protein tau
NRCAM	neuronal cell adhesion molecule
PAX2	paired box 2
PPT1	palmitoyl-protein thioesterase 1
ROBO1	roundabout guidance receptor 1
ROBO2	roundabout guidance receptor 2
RTN1	reticulon 1
S100B	S100 calcium binding protein B
SHH	sonic hedgehog
TGFB2	transforming growth factor beta 2
UBB	ubiquitin B

YWHAG	tyrosine 3-monooxygenase/tryptophan 5-monooxygenase activation protein gamma
SEMA3B	semaphorin 3B
LST1	leukocyte specific transcript 1
NRP2	neuropilin 2
NRP1	neuropilin 1
BRSK2	BR serine/threonine kinase 2
SLIT2	slit guidance ligand 2
NRXN3	neurexin 3
FEZ2	fasciculation and elongation protein zeta 2
SPON2	spondin 2
BAIAP2	BAI1 associated protein 2
SEMA4F	ssemaphorin 4F
POU6F2	POU class 6 homeobox 2
NTNG1	netrin G1
NLGN1	neuroligin 1
CYFIP1	cytoplasmic FMR1 interacting protein 1
PARD3	par-3 family cell polarity regulator
RTN4	reticulon 4
PARD6B	par-6 family cell polarity regulator beta
RTN4RL1	reticulon 4 receptor like 1
MDGA2	MAM domain containing glycosylphosphatidylinositol anchor 2
MDGA1	MAM domain containing glycosylphosphatidylinositol anchor 1
RTN4RL2	reticulon 4 receptor-like 2
VWC2	von Willebrand factor C domain containing 2
AGRN	agrin
**Neurogenesis**	
**Gene Symbol**	**Name Gene**
ATP2B2	ATPase plasma membrane Ca2+ transporting 2
AZU1	azurocidin 1
CDK6	cyclin-dependent kinase 6
CLN5	ceroid-lipofuscinosis, neuronal 5
DTX1	deltex 1
GDNF	glial cell derived neurotrophic factor
GLI2	GLI family zinc finger 2
LAMB1	laminin subunit beta 1
LMX1B	LIM homeobox transcription factor 1 beta
MAPT	microtubule associated protein tau
NF1	neurofibromin 1
NF2	neurofibromin 2 (merlin)
NRCAM	neuronal cell adhesion molecule
PAX2	paired box 2
PPT1	palmitoyl-protein thioesterase 1
ROBO1	roundabout guidance receptor 1
ROBO2	roundabout guidance receptor 2
RTN1	reticulon 1
S100B	S100 calcium binding protein B
SHH	sonic hedgehog
SOD1	superoxide dismutase 1, soluble
TGFB2	transforming growth factor beta 2
UBB	ubiquitin B
YWHAG	tyrosine 3-monooxygenase/tryptophan 5-monooxygenase activation protein gamma
SEMA3B	semaphorin 3B
LST1	leukocyte specific transcript 1
NRP2	neuropilin 2
NRP1	neuropilin 1
EIF2B3	eukaryotic translation initiation factor 2B subunit gamma

BRSK2	BR serine/threonine kinase 2
SLIT2	slit guidance ligand 2
NRXN3	neurexin 3
FEZ2	fasciculation and elongation protein zeta 2
SPON2	spondin 2
BAIAP2	BAI1 associated protein 2
SEMA4F	ssemaphorin 4F
CIT	citron rho-interacting serine/threonine kinase
POU6F2	POU class 6 homeobox 2
NTNG1	netrin G1
NLGN1	neuroligin 1
CYFIP1	cytoplasmic FMR1 interacting protein 1
RACGAP1	Rac GTPase activating protein 1
PARD3	par-3 family cell polarity regulator
RTN4	reticulon 4
PARD6B	par-6 family cell polarity regulator beta
RTN4RL1	reticulon 4 receptor like 1
MDGA2	MAM domain containing glycosylphosphatidylinositol anchor 2
MDGA1	MAM domain containing glycosylphosphatidylinositol anchor 1
RTN4RL2	reticulon 4 receptor-like 2
VWC2	von Willebrand factor C domain containing 2
AGRN	agrin
**Generation of neurons**	
**Gene Symbol**	**Name Gene**
ATP2B2	ATPase plasma membrane Ca2+ transporting 2
DTX1	deltex 1
GDNF	glial cell derived neurotrophic factor
GLI2	GLI family zinc finger 2
LAMB1	laminin subunit beta 1
LMX1B	LIM homeobox transcription factor 1 beta
MAPT	microtubule associated protein tau
NF1	neurofibromin 1
NRCAM	neuronal cell adhesion molecule
PAX2	paired box 2
PPT1	palmitoyl-protein thioesterase 1
ROBO1	roundabout guidance receptor 1
ROBO2	roundabout guidance receptor 2
RTN1	reticulon 1
S100B	S100 calcium binding protein B
SHH	sonic hedgehog
TGFB2	transforming growth factor beta 2
UBB	ubiquitin B
YWHAG	tyrosine 3-monooxygenase/tryptophan 5-monooxygenase activation protein gamma
SEMA3B	semaphorin 3B
LST1	leukocyte specific transcript 1
NRP2	neuropilin 2
NRP1	neuropilin 1
BRSK2	BR serine/threonine kinase 2
SLIT2	slit guidance ligand 2
NRXN3	neurexin 3
FEZ2	fasciculation and elongation protein zeta 2
SPON2	spondin 2
BAIAP2	BAI1 associated protein 2
SEMA4F	ssemaphorin 4F
CIT	citron rho-interacting serine/threonine kinase
POU6F2	POU class 6 homeobox 2
NTNG1	netrin G1

NLGN1	neuroligin 1
CYFIP1	cytoplasmic FMR1 interacting protein 1
RACGAP1	Rac GTPase activating protein 1
PARD3	par-3 family cell polarity regulator
RTN4	reticulon 4
PARD6B	par-6 family cell polarity regulator beta
RTN4RL1	reticulon 4 receptor like 1
MDGA2	MAM domain containing glycosylphosphatidylinositol anchor 2
MDGA1	MAM domain containing glycosylphosphatidylinositol anchor 1
RTN4RL2	reticulon 4 receptor-like 2
VWC2	von Willebrand factor C domain containing 2
AGRN	agrin
**Nervous system development**	
**Gene Symbol**	**Name Gene**
ALK	anaplastic lymphoma receptor tyrosine kinase
APBA1	amyloid beta precursor protein binding family A member 1
APBA2	amyloid beta precursor protein binding family A member 2
ATP2B2	ATPase plasma membrane Ca2+ transporting 2
AZU1	azurocidin 1
BTD	biotinidase
MPPED2	metallophosphoesterase domain containing 2
CBLN1	cerebellin 1 precursor
CD9	CD9 molecule
CDK6	cyclin-dependent kinase 6
CHRM1	cholinergic receptor muscarinic 1
CHRM2	cholinergic receptor muscarinic 2
CHRM3	cholinergic receptor muscarinic 3
CHRNA4	cholinergic receptor nicotinic alpha 4 subunit
CLN5	ceroid-lipofuscinosis, neuronal 5
CNTFR	ciliary neurotrophic factor receptor
CRMP1	collapsin response mediator protein 1
CTF1	cardiotrophin 1
CTNS	cystinosin, lysosomal cystine transporter
DLG4	discs large homolog 4
DLX2	distal-less homeobox 2
DLX5	distal-less homeobox 5
DPYSL2	dihydropyrimidinase like 2
ATN1	atrophin 1
DSCAM	DS cell adhesion molecule
RCAN1	regulator of calcineurin 1
DTX1	deltex 1
DYRK1A	dual specificity tyrosine phosphorylation regulated kinase 1A
EFNA5	ephrin A5
EP300	E1A binding protein p300
EPHB2	EPH receptor B2
FGF5	fibroblast growth factor 5
FGF12	fibroblast growth factor 12
FOXG1	forkhead box G1
GDNF	glial cell derived neurotrophic factor
GFRA3	GDNF family receptor alpha 3
GLI2	GLI family zinc finger 2
GSS	glutathione synthetase
GSTP1	glutathione S-transferase pi 1
HES1	hes family bHLH transcription factor 1
JARID2	jumonji and AT-rich interaction domain containing 2
KCNN3	potassium calcium-activated channel subfamily N member 3
KCNQ2	potassium voltage-gated channel subfamily Q member 2
LAMB1	laminin subunit beta 1

LMX1B	LIM homeobox transcription factor 1 beta
LY6H	lymphocyte antigen 6 complex, locus H
MAPT	microtubule associated protein tau
MBNL1	muscleblind like splicing regulator 1
MBP	myelin basic protein
MEF2C	myocyte enhancer factor 2C
MOG	myelin oligodendrocyte glycoprotein
NEUROG1	neurogenin 1
NF1	neurofibromin 1
NF2	neurofibromin 2 (merlin)
NHLH2	nescient helix-loop-helix 2
NINJ1	ninjurin 1
NPAS2	neuronal PAS domain protein 2
NPTX1	neuronal pentraxin 1
NRCAM	neuronal cell adhesion molecule
NRGN	neurogranin
NTF3	neurotrophin 3
PARK2	parkin RBR E3 ubiquitin protein ligase
PAX2	paired box 2
PBX1	PBX homeobox 1
PCDH1	protocadherin 1
PPT1	palmitoyl-protein thioesterase 1
PSPN	persephin
PTEN	phosphatase and tensin homolog
PTS	6-pyruvoyltetrahydropterin synthase
ROBO1	roundabout guidance receptor 1
ROBO2	roundabout guidance receptor 2
RTN1	reticulon 1
S100B	S100 calcium binding protein B
SCN8A	sodium voltage-gated channel alpha subunit 8
SH3GL3	SH3 domain containing GRB2 like endophilin A3
SHH	sonic hedgehog
SHOX2	short stature homeobox 2
SIM1	single-minded family bHLH transcription factor 1
SIM2	single-minded family bHLH transcription factor 2
SIX3	SIX homeobox 3
SLIT3	slit guidance ligand 3
SNCA	synuclein alpha
SOD1	superoxide dismutase 1, soluble
SOX11	SRY-box 11
SPG7	SPG7, paraplegin matrix AAA peptidase subunit
SPOCK1	sparc/osteonectin, cwcv and kazal-like domains proteoglycan (testican) 1
STAT3	signal transducer and activator of transcription 3
TFAP2B	transcription factor AP-2 beta
TGFB2	transforming growth factor beta 2
NR2E1	nuclear receptor subfamily 2 group E member 1
NR2C2	nuclear receptor subfamily 2 group C member 2
UBB	ubiquitin B
UBE3A	ubiquitin protein ligase E3A
UGT8	UDP glycosyltransferase 8
VEGFA	vascular endothelial growth factor A
YWHAG	tyrosine 3-monooxygenase/tryptophan 5-monooxygenase activation protein gamma
ZIC1	Zic family member 1
ZBTB16	zinc finger and BTB domain containing 16
SEMA3B	semaphorin 3B
LST1	leukocyte specific transcript 1
ST8SIA2	ST8 alpha-N-acetyl-neuraminide alpha-2,8-sialyltransferase 2

DGCR14	DiGeorge syndrome critical region gene 14
FZD9	frizzled class receptor 9
SOX14	SRY-box 14
ENC1	ectodermal-neural cortex 1
ADAM23	ADAM metallopeptidase domain 23
FGF17	fibroblast growth factor 17
NRP2	neuropilin 2
NRP1	neuropilin 1
EIF2B3	eukaryotic translation initiation factor 2B subunit gamma
KALRN	kalirin, RhoGEF kinase
BRSK2	BR serine/threonine kinase 2
DCLK1	doublecortin like kinase 1
LGI1	leucine-rich, glioma inactivated 1
NUMBL	NUMB like, endocytic adaptor protein
SLIT2	slit guidance ligand 2
CPNE6	copine 6
NRXN3	neurexin 3
CELSR1	cadherin EGF LAG seven-pass G-type receptor 1
FEZ2	fasciculation and elongation protein zeta 2
ECE2	endothelin converting enzyme 2
HDAC4	histone deacetylase 4
FGF19	fibroblast growth factor 19
SPON2	spondin 2
OLFM1	olfactomedin 1
BAIAP2	BAI1 associated protein 2
SEMA4F	ssemaphorin 4F
AVIL	advillin
TBR1	T-box, brain 1
CYP46A1	cytochrome P450 family 46 subfamily A member 1
CIT	citron rho-interacting serine/threonine kinase
POU6F2	POU class 6 homeobox 2
NTNG1	netrin G1
NLGN1	neuroligin 1
MYO16	myosin XVI
CYFIP1	cytoplasmic FMR1 interacting protein 1
ATXN10	ataxin 10
PHGDH	phosphoglycerate dehydrogenase
LHX6	LIM homeobox 6
HEYL	hes related family bHLH transcription factor with YRPW motif-like
MYLIP	myosin regulatory light chain interacting protein
RACGAP1	Rac GTPase activating protein 1
TMOD2	tropomodulin 2
SOX8	SRY-box 8
STMN3	stathmin 3
UTP11	UTP11, small subunit processome component homolog (S. cerevisiae)
CRIM1	cysteine rich transmembrane BMP regulator 1 (chordin-like)
HPCAL4	hippocalcin like 4
HDAC7	histone deacetylase 7
SNTG2	syntrophin gamma 2
PCDH18	protocadherin 18
MAML3	mastermind like transcriptional coactivator 3
PCDHA1	protocadherin alpha 1
PARD3	par-3 family cell polarity regulator
RTN4	reticulon 4
VANGL2	VANGL planar cell polarity protein 2
DSCAML1	DS cell adhesion molecule like 1
MARK4	microtubule affinity regulating kinase 4
PARD6B	par-6 family cell polarity regulator beta

FOXP2	forkhead box P2
NRSN1	neurensin 1
RTN4RL1	reticulon 4 receptor like 1
MDGA2	MAM domain containing glycosylphosphatidylinositol anchor 2
MDGA1	MAM domain containing glycosylphosphatidylinositol anchor 1
RTN4RL2	reticulon 4 receptor-like 2
VWC2	von Willebrand factor C domain containing 2
AGRN	agrin
**Central nervous system development**	
**Gene Symbol**	**Name Gene**
ALK	anaplastic lymphoma receptor tyrosine kinase
BTD	biotinidase
CLN5	ceroid-lipofuscinosis, neuronal 5
CTNS	cystinosin, lysosomal cystine transporter
DLX2	distal-less homeobox 2
ATN1	atrophin 1
RCAN1	regulator of calcineurin 1
FOXG1	forkhead box G1
GLI2	GLI family zinc finger 2
GSTP1	glutathione S-transferase pi 1
JARID2	jumonji and AT-rich interaction domain containing 2
MBP	myelin basic protein
MOG	myelin oligodendrocyte glycoprotein
NF1	neurofibromin 1
NHLH2	nescient helix-loop-helix 2
NPAS2	neuronal PAS domain protein 2
NPTX1	neuronal pentraxin 1
PARK2	parkin RBR E3 ubiquitin protein ligase
PBX1	PBX homeobox 1
PPT1	palmitoyl-protein thioesterase 1
PSPN	persephin
PTEN	phosphatase and tensin homolog
PTS	6-pyruvoyltetrahydropterin synthase
SH3GL3	SH3 domain containing GRB2 like endophilin A3
SHH	sonic hedgehog
SIX3	SIX homeobox 3
SLIT3	slit guidance ligand 3
SNCA	synuclein alpha
UBE3A	ubiquitin protein ligase E3A
ZBTB16	zinc finger and BTB domain containing 16
ADAM23	ADAM metallopeptidase domain 23
EIF2B3	eukaryotic translation initiation factor 2B subunit gamma
DCLK1	doublecortin like kinase 1
CELSR1	cadherin EGF LAG seven-pass G-type receptor 1
ECE2	endothelin converting enzyme 2
TBR1	T-box, brain 1
POU6F2	POU class 6 homeobox 2
MYO16	myosin XVI
PHGDH	phosphoglycerate dehydrogenase
LHX6	LIM homeobox 6
SOX8	SRY-box 8
HPCAL4	hippocalcin like 4
SNTG2	syntrophin gamma 2
PCDH18	protocadherin 18
DSCAML1	DS cell adhesion molecule like 1
MDGA2	MAM domain containing glycosylphosphatidylinositol anchor 2
MDGA1	MAM domain containing glycosylphosphatidylinositol anchor 1

Table S2 - Gene collection of Incident group

**Table S2** - Gene collection of Incident group	
**Biological processes**	
**System development**	
**Gene Symbol**	**Name Gene**
ACTA1	actin, alpha 1, skeletal muscle
ACVRL1	activin A receptor like type 1
AEBP1	AE binding protein 1
ALDH3A2	aldehyde dehydrogenase 3 family member A2
ALK	anaplastic lymphoma receptor tyrosine kinase
ALOX12B	arachidonate 12-lipoxygenase, 12R type
ANG	angiogenin
APAF1	apoptotic peptidase activating factor 1
APLP1	amyloid beta precursor like protein 1
ATP2B2	ATPase plasma membrane Ca2+ transporting 2
ATP6V1B1	ATPase H+ transporting V1 subunit B1
AZU1	azurocidin 1
BDNF	brain-derived neurotrophic factor
BNC1	basonuclin 1
BMP1	bone morphogenetic protein 1
BMP2	bone morphogenetic protein 2
FOXL2	forkhead box L2
BTD	biotinidase
MPPED2	metallophosphoesterase domain containing 2
CACNB2	calcium voltage-gated channel auxiliary subunit beta 2
CALCA	calcitonin related polypeptide alpha
CANX	calnexin
CASR	calcium sensing receptor
RUNX2	runt related transcription factor 2
RUNX1	runt related transcription factor 1
CBLN1	cerebellin 1 precursor
CDH13	cadherin 13
CDK6	cyclin-dependent kinase 6
CDX1	caudal type homeobox 1
CHRM1	cholinergic receptor muscarinic 1
CHRM2	cholinergic receptor muscarinic 2
CHRM3	cholinergic receptor muscarinic 3
CHRNA1	cholinergic receptor nicotinic alpha 1 subunit
CHRNA4	cholinergic receptor nicotinic alpha 4 subunit
CMKLR1	chemerin chemokine-like receptor 1
CNTFR	ciliary neurotrophic factor receptor
COL1A1	collagen type I alpha 1
COL1A2	collagen type I alpha 2
COL4A2	collagen type IV alpha 2
COL5A2	collagen type V alpha 2
COL6A3	collagen type VI alpha 3
COL7A1	collagen type VII alpha 1
COL9A1	collagen type IX alpha 1
COL9A2	collagen type IX alpha 2
COL12A1	collagen type XII alpha 1
COL13A1	collagen type XIII alpha 1
CTNS	cystinosin, lysosomal cystine transporter
DCN	decorin
DLX2	distal-less homeobox 2
DLX5	distal-less homeobox 5
DPYSL2	dihydropyrimidinase like 2

DPYSL3	dihydropyrimidinase like 3
DSCAM	DS cell adhesion molecule
DSP	desmoplakin
DTX1	deltex 1
DVL3	dishevelled segment polarity protein 3
DYRK1A	dual specificity tyrosine phosphorylation regulated kinase 1A
EFNA5	ephrin A5
EGF	epidermal growth factor
EGR2	early growth response 2
EGR3	early growth response 3
ELN	elastin
EMP1	epithelial membrane protein 1
EN1	engrailed homeobox 1
EPHB1	EPH receptor B1
EPHB2	EPH receptor B2
STX2	syntaxin 2
CLN8	ceroid-lipofuscinosis, neuronal 8
ERBB2	erb-b2 receptor tyrosine kinase 2
ERCC2	excision repair cross-complementation group 2
ERCC3	excision repair cross-complementation group 3
ETS1	ETS proto-oncogene 1, transcription factor
EVC	EvC ciliary complex subunit 1
EVPL	envoplakin
EXT2	exostosin glycosyltransferase 2
FBN1	fibrillin 1
FGF12	fibroblast growth factor 12
FGFR3	fibroblast growth factor receptor 3
FHL3	four and a half LIM domains 3
FOXG1	forkhead box G1
FOXC1	forkhead box C1
FOXC2	forkhead box C2
FOXE1	forkhead box E1
FOXO3	forkhead box O3
FLI1	Fli-1 proto-oncogene, ETS transcription factor
FSHR	follicle stimulating hormone receptor
NR5A1	nuclear receptor subfamily 5 group A member 1
GATA4	GATA binding protein 4
GATA6	GATA binding protein 6
GDNF	glial cell derived neurotrophic factor
GFRA3	GDNF family receptor alpha 3
GJB5	gap junction protein beta 5
GLI2	GLI family zinc finger 2
CXCL1	C-X-C motif chemokine ligand 1
HCLS1	hematopoietic cell-specific Lyn substrate 1
HTT	huntingtin
HOXA13	homeobox A13
HOXD13	homeobox D13
HRAS	Harvey rat sarcoma viral oncogene homolog
HES1	hes family bHLH transcription factor 1
IGFBP3	insulin like growth factor binding protein 3
IGFBP4	insulin like growth factor binding protein 4
IL7	interleukin 7
PDX1	pancreatic and duodenal homeobox 1
JAG2	jagged 2
JAK2	Janus kinase 2
JARID2	jumonji and AT-rich interaction domain containing 2
KCNQ2	potassium voltage-gated channel subfamily Q member 2
KRT1	keratin 1

KRT6A	keratin 6A
KRT10	keratin 10
KRT15	keratin 15
KRT32	keratin 32
LAMA3	laminin subunit alpha 3
LAMB3	laminin subunit beta 3
LAMC1	laminin subunit gamma 1
LAMC2	laminin subunit gamma 2
LCK	LCK proto-oncogene, Src family tyrosine kinase
LHX1	LIM homeobox 1
LIG1	DNA ligase 1
ABLIM1	actin binding LIM protein 1
LMX1B	LIM homeobox transcription factor 1 beta
LRMP	lymphoid restricted membrane protein
LRCH4	leucine-rich repeats and calponin homology (CH) domain containing 4
LSAMP	limbic system-associated membrane protein
LY6H	lymphocyte antigen 6 complex, locus H
MAL	mal, T-cell differentiation protein
MAPT	microtubule associated protein tau
MBNL1	muscleblind like splicing regulator 1
MBP	myelin basic protein
MEA1	male-enhanced antigen 1
MEF2C	myocyte enhancer factor 2C
MEF2D	myocyte enhancer factor 2D
MEST	mesoderm specific transcript
MLF1	myeloid leukemia factor 1
MOG	myelin oligodendrocyte glycoprotein
MSX1	msh homeobox 1
MSX2	msh homeobox 2
MYBPC3	myosin binding protein C, cardiac
MYH7	myosin, heavy chain 7, cardiac muscle, beta
MYH9	myosin, heavy chain 9, non-muscle
MYH11	myosin, heavy chain 11, smooth muscle
MYOD1	myogenic differentiation 1
NAB2	NGFI-A binding protein 2
NCL	nucleolin
NDUFV2	NADH:ubiquinone oxidoreductase core subunit V2
NELL1	neural EGFL like 1
NEUROG1	neurogenin 1
NF1	neurofibromin 1
NF2	neurofibromin 2 (merlin)
NHLH2	nescient helix-loop-helix 2
NINJ2	ninjurin 2
NKX2-2	NK2 homeobox 2
NKX6-1	NK6 homeobox 1
NOTCH1	notch 1
NOTCH4	notch 4
NPAS2	neuronal PAS domain protein 2
NPR1	natriuretic peptide receptor 1
NPR3	natriuretic peptide receptor 3
NPTX1	neuronal pentraxin 1
NRCAM	neuronal cell adhesion molecule
NRDC	nardilysin convertase
NTF3	neurotrophin 3
TNFRSF11B	tumor necrosis factor receptor superfamily member 11b
PARK2	parkin RBR E3 ubiquitin protein ligase
PAX2	paired box 2

PAX5	paired box 5
PAX6	paired box 6
PBX1	PBX homeobox 1
PBX3	PBX homeobox 3
PCDH1	protocadherin 1
PCP4	Purkinje cell protein 4
PF4	platelet factor 4
SERPINI1	serpin family I member 1
PITX2	paired like homeodomain 2
PLG	plasminogen
POU3F3	POU class 3 homeobox 3
POU4F2	POU class 4 homeobox 2
POU6F1	POU class 6 homeobox 1
PPARD	peroxisome proliferator activated receptor delta
PRELP	proline/arginine-rich end leucine-rich repeat protein
SRGN	serglycin
PRKACG	protein kinase cAMP-activated catalytic subunit gamma
PRL	prolactin
PROP1	PROP paired-like homeobox 1
PROX1	prospero homeobox 1
PTCH1	patched 1
PTH1R	parathyroid hormone 1 receptor
RASA1	RAS p21 protein activator 1
RBP2	retinol binding protein 2
RNH1	ribonuclease/angiogenin inhibitor 1
ROBO1	roundabout guidance receptor 1
ROBO2	roundabout guidance receptor 2
RPS19	ribosomal protein S19
RTN1	reticulon 1
S100A7	S100 calcium binding protein A7
S100B	S100 calcium binding protein B
MAPK12	mitogen-activated protein kinase 12
SCN8A	sodium voltage-gated channel alpha subunit 8
SECTM1	secreted and transmembrane 1
SGCA	sarcoglycan alpha
SGCD	sarcoglycan delta
SH3GL2	SH3 domain containing GRB2 like 2, endophilin A1
SHH	sonic hedgehog
SIAH1	siah E3 ubiquitin protein ligase 1
SIM1	single-minded family bHLH transcription factor 1
SIM2	single-minded family bHLH transcription factor 2
SIX3	SIX homeobox 3
SLIT1	slit guidance ligand 1
SLIT3	slit guidance ligand 3
SNAI2	snail family transcriptional repressor 2
SMPD1	sphingomyelin phosphodiesterase 1
SNCA	synuclein alpha
SOX11	SRY-box 11
SPARC	secreted protein acidic and cysteine rich
SPG7	SPG7, paraplegin matrix AAA peptidase subunit
SPI1	Spi-1 proto-oncogene
SPP2	secreted phosphoprotein 2
SPOCK1	sparc/osteonectin, cwcv and kazal-like domains proteoglycan (testican) 1
STAT3	signal transducer and activator of transcription 3
SVIL	supervillin
TBX5	T-box 5
TBX3	T-box 3

TCF12	transcription factor 12
TCOF1	treacle ribosome biogenesis factor 1
TFAP2A	transcription factor AP-2 alpha
TFAP2B	transcription factor AP-2 beta
TGFB1	transforming growth factor beta 1
KLF10	Kruppel-like factor 10
TIE1	tyrosine kinase with immunoglobulin like and EGF like domains 1
TLE2	transducin like enhancer of split 2
TLE3	transducin like enhancer of split 3
TLL1	tolloid like 1
NR2E1	nuclear receptor subfamily 2 group E member 1
TPD52	tumor protein D52
NR2C2	nuclear receptor subfamily 2 group C member 2
TRPS1	transcriptional repressor GATA binding 1
UBB	ubiquitin B
UTRN	utrophin
WNT8B	Wnt family member 8B
YWHAG	tyrosine 3-monooxygenase/tryptophan 5-monooxygenase activation protein gamma
ZAP70	zeta chain of T cell receptor associated protein kinase 70kDa
ZIC2	Zic family member 2
ZBTB16	zinc finger and BTB domain containing 16
CSDE1	cold shock domain containing E1
LST1	leukocyte specific transcript 1
LHX3	LIM homeobox 3
ADAM12	ADAM metallopeptidase domain 12
ALX1	ALX homeobox 1
ST8SIA2	ST8 alpha-N-acetyl-neuraminide alpha-2,8-sialyltransferase 2
MKKS	McKusick-Kaufman syndrome
DGCR6	DiGeorge syndrome critical region gene 6
DGCR14	DiGeorge syndrome critical region gene 14
DYRK3	dual specificity tyrosine phosphorylation regulated kinase 3
LMO4	LIM domain only 4
ADAM23	ADAM metallopeptidase domain 23
SCEL	sciellin
NRP2	neuropilin 2
NRP1	neuropilin 1
EIF2B3	eukaryotic translation initiation factor 2B subunit gamma
EIF2B2	eukaryotic translation initiation factor 2B subunit beta
CACNA1H	calcium voltage-gated channel subunit alpha1 H
KALRN	kalirin, RhoGEF kinase
BRSK2	BR serine/threonine kinase 2
SEMA5A	semaphorin 5A
PAPSS1	3'-phosphoadenosine 5'-phosphosulfate synthase 1
SART1	squamous cell carcinoma antigen recognized by T-cells 1
LARGE	like-glycosyltransferase
KLF4	Kruppel-like factor 4 (gut)
TGM5	transglutaminase 5
SLIT2	slit guidance ligand 2
KL	klotho
NRXN3	neurexin 3
NRXN1	neurexin 1
HAND2	heart and neural crest derivatives expressed 2
TBX4	T-box 4
CELSR1	cadherin EGF LAG seven-pass G-type receptor 1
HDAC9	histone deacetylase 9
HDAC4	histone deacetylase 4
ARNT2	aryl hydrocarbon receptor nuclear translocator 2

FGF19	fibroblast growth factor 19
HDAC5	histone deacetylase 5
CLEC3A	C-type lectin domain family 3 member A
SPRY2	sprouty RTK signaling antagonist 2
SPEG	SPEG complex locus
IKZF1	IKAROS family zinc finger 1
SPON2	spondin 2
OLFM1	olfactomedin 1
BAIAP2	BAI1 associated protein 2
HOXB13	homeobox B13
SEMA4F	ssemaphorin 4F
DPYSL4	dihydropyrimidinase like 4
PDPN	podoplanin
DMRT2	doublesex and mab-3 related transcription factor 2
AVIL	advillin
DLL3	delta like canonical Notch ligand 3
TBR1	T-box, brain 1
VAMP5	vesicle associated membrane protein 5
CYP46A1	cytochrome P450 family 46 subfamily A member 1
GPR45	G protein-coupled receptor 45
POU6F2	POU class 6 homeobox 2
NTNG1	netrin G1
NLGN1	neuroligin 1
MYO16	myosin XVI
STAB1	stabilin 1
CYFIP1	cytoplasmic FMR1 interacting protein 1
VAX2	ventral anterior homeobox 2
ATXN10	ataxin 10
KLK5	kallikrein related peptidase 5
POU2F3	POU class 2 homeobox 3
PHGDH	phosphoglycerate dehydrogenase
LHX6	LIM homeobox 6
NPTN	neuroplastin
ATP2C1	ATPase secretory pathway Ca2+ transporting 1
RND1	Rho family GTPase 1
MYLIP	myosin regulatory light chain interacting protein
TMOD2	tropomodulin 2
SOX8	SRY-box 8
IL20	interleukin 20
IRX4	iroquois homeobox 4
STMN3	stathmin 3
UTP11	UTP11, small subunit processome component homolog (S. cerevisiae)
EGFL7	EGF like domain multiple 7
PLCE1	phospholipase C epsilon 1
CRIM1	cysteine rich transmembrane BMP regulator 1 (chordin-like)
TRAPPC4	trafficking protein particle complex 4
HPCAL4	hippocalcin like 4
SUFU	SUFU negative regulator of hedgehog signaling
EMCN	endomucin
SNTG2	syntrophin gamma 2
PCDH18	protocadherin 18
ROBO4	roundabout guidance receptor 4
AGGF1	angiogenic factor with G-patch and FHA domains 1
MAP1S	microtubule associated protein 1S
MAML3	mastermind like transcriptional coactivator 3
PCDHB13	protocadherin beta 13
PCDHB12	protocadherin beta 12

PCDHB4	protocadherin beta 4
PCDHA1	protocadherin alpha 1
KIAA1217	KIAA1217
PARD3	par-3 family cell polarity regulator
ANKRD7	ankyrin repeat domain 7
C1GALT1	core 1 synthase, glycoprotein-N-acetylgalactosamine 3-beta-galactosyltransferase 1
RTN4	reticulon 4
SMURF1	SMAD specific E3 ubiquitin protein ligase 1
DSCAML1	DS cell adhesion molecule like 1
AMIGO1	adhesion molecule with Ig-like domain 1
ALPK3	alpha kinase 3
LRRC4C	leucine rich repeat containing 4C
PCDHB16	protocadherin beta 16
MARK4	microtubule affinity regulating kinase 4
NHEJ1	non-homologous end joining factor 1
COL18A1	collagen type XVIII alpha 1
CCM2	CCM2 scaffolding protein
PARD6B	par-6 family cell polarity regulator beta
KIRREL3	kin of IRRE like 3 (Drosophila)
NTNG2	netrin G2
BOC	BOC cell adhesion associated, oncogene regulated
ATPIF1	ATPase inhibitory factor 1
FOXP2	forkhead box P2
APOA5	apolipoprotein A5
GYLTL1B	glycosyltransferase-like 1B
DMBX1	diencephalon/mesencephalon homeobox 1
IL31RA	interleukin 31 receptor A
NKX2-6	NK2 homeobox 6
RTN4RL1	reticulon 4 receptor like 1
NFAM1	NFAT activating protein with ITAM motif 1
CNTN4	contactin 4
MDGA2	MAM domain containing glycosylphosphatidylinositol anchor 2
ZNF384	zinc finger protein 384
IL27	interleukin 27
MDGA1	MAM domain containing glycosylphosphatidylinositol anchor 1
RTN4RL2	reticulon 4 receptor-like 2
AGRN	agrin
**Nervous system development**	
**Gene Symbol**	**Name Gene**
ALDH3A2	aldehyde dehydrogenase 3 family member A2
ALK	anaplastic lymphoma receptor tyrosine kinase
APAF1	apoptotic peptidase activating factor 1
APLP1	amyloid beta precursor like protein 1
ATP2B2	ATPase plasma membrane Ca2+ transporting 2
AZU1	azurocidin 1
BDNF	brain-derived neurotrophic factor
BTD	biotinidase
MPPED2	metallophosphoesterase domain containing 2
CBLN1	cerebellin 1 precursor
CDK6	cyclin-dependent kinase 6
CHRM1	cholinergic receptor muscarinic 1
CHRM2	cholinergic receptor muscarinic 2
CHRM3	cholinergic receptor muscarinic 3
CHRNA4	cholinergic receptor nicotinic alpha 4 subunit
CNTFR	ciliary neurotrophic factor receptor
CTF1	cardiotrophin 1
CTNS	cystinosin, lysosomal cystine transporter

DLX2	distal-less homeobox 2
DLX5	distal-less homeobox 5
DPYSL2	dihydropyrimidinase like 2
DPYSL3	dihydropyrimidinase like 3
DSCAM	DS cell adhesion molecule
DTX1	deltex 1
DVL3	dishevelled segment polarity protein 3
DYRK1A	dual specificity tyrosine phosphorylation regulated kinase 1A
EFNA5	ephrin A5
EGR2	early growth response 2
EPHB1	EPH receptor B1
EPHB2	EPH receptor B2
CLN8	ceroid-lipofuscinosis, neuronal 8
FGF12	fibroblast growth factor 12
FOXG1	forkhead box G1
GDNF	glial cell derived neurotrophic factor
GFRA3	GDNF family receptor alpha 3
GLI2	GLI family zinc finger 2
CXCL1	C-X-C motif chemokine ligand 1
HES1	hes family bHLH transcription factor 1
JARID2	jumonji and AT-rich interaction domain containing 2
KCNQ2	potassium voltage-gated channel subfamily Q member 2
LHX1	LIM homeobox 1
LMX1B	LIM homeobox transcription factor 1 beta
LRCH4	leucine-rich repeats and calponin homology (CH) domain containing 4
LSAMP	limbic system-associated membrane protein
LY6H	lymphocyte antigen 6 complex, locus H
MAL	mal, T-cell differentiation protein
MAPT	microtubule associated protein tau
MBNL1	muscleblind like splicing regulator 1
MBP	myelin basic protein
MEF2C	myocyte enhancer factor 2C
MOG	myelin oligodendrocyte glycoprotein
NAB2	NGFI-A binding protein 2
NDUFV2	NADH:ubiquinone oxidoreductase core subunit V2
NELL1	neural EGFL like 1
NEUROD2	neuronal differentiation 2
NEUROG1	neurogenin 1
NF1	neurofibromin 1
NF2	neurofibromin 2 (merlin)
NHLH2	nescient helix-loop-helix 2
NINJ2	ninjurin 2
NKX2-2	NK2 homeobox 2
NPAS2	neuronal PAS domain protein 2
NPTX1	neuronal pentraxin 1
NRCAM	neuronal cell adhesion molecule
NRTN	neurturin
NTF3	neurotrophin 3
PAFAH1B3	platelet activating factor acetylhydrolase 1b catalytic subunit 3
PARK2	parkin RBR E3 ubiquitin protein ligase
PAX2	paired box 2
PAX6	paired box 6
PBX1	PBX homeobox 1
PBX3	PBX homeobox 3
PCDH1	protocadherin 1
PCP4	Purkinje cell protein 4
SERPINI1	serpin family I member 1

POU3F3	POU class 3 homeobox 3
POU4F2	POU class 4 homeobox 2
POU6F1	POU class 6 homeobox 1
PPARD	peroxisome proliferator activated receptor delta
PSPN	persephin
PROP1	PROP paired-like homeobox 1
PTS	6-pyruvoyltetrahydropterin synthase
ROBO1	roundabout guidance receptor 1
ROBO2	roundabout guidance receptor 2
RTN1	reticulon 1
S100B	S100 calcium binding protein B
SCN8A	sodium voltage-gated channel alpha subunit 8
SH3GL2	SH3 domain containing GRB2 like 2, endophilin A1
SH3GL3	SH3 domain containing GRB2 like endophilin A3
SHH	sonic hedgehog
SIAH1	siah E3 ubiquitin protein ligase 1
SIM1	single-minded family bHLH transcription factor 1
SIM2	single-minded family bHLH transcription factor 2
SIX3	SIX homeobox 3
SLIT1	slit guidance ligand 1
SLIT3	slit guidance ligand 3
SMPD1	sphingomyelin phosphodiesterase 1
SNCA	synuclein alpha
SOX11	SRY-box 11
SPG7	SPG7, paraplegin matrix AAA peptidase subunit
SPOCK1	sparc/osteonectin, cwcv and kazal-like domains proteoglycan (testican) 1
STAT3	signal transducer and activator of transcription 3
TFAP2B	transcription factor AP-2 beta
NR2E1	nuclear receptor subfamily 2 group E member 1
NR2C2	nuclear receptor subfamily 2 group C member 2
UBB	ubiquitin B
WNT8B	Wnt family member 8B
YWHAG	tyrosine 3-monooxygenase/tryptophan 5-monooxygenase activation protein gamma
ZIC2	Zic family member 2
ZBTB16	zinc finger and BTB domain containing 16
LST1	leukocyte specific transcript 1
ALX1	ALX homeobox 1
ST8SIA2	ST8 alpha-N-acetyl-neuraminide alpha-2,8-sialyltransferase 2
DGCR14	DiGeorge syndrome critical region gene 14
LMO4	LIM domain only 4
ADAM23	ADAM metallopeptidase domain 23
NRP2	neuropilin 2
NRP1	neuropilin 1
EIF2B3	eukaryotic translation initiation factor 2B subunit gamma
EIF2B2	eukaryotic translation initiation factor 2B subunit beta
KALRN	kalirin, RhoGEF kinase
BRSK2	BR serine/threonine kinase 2
SEMA5A	semaphorin 5A
LGI1	leucine-rich, glioma inactivated 1
SLIT2	slit guidance ligand 2
NRXN3	neurexin 3
NRXN1	neurexin 1
CELSR1	cadherin EGF LAG seven-pass G-type receptor 1
HDAC4	histone deacetylase 4
ARNT2	aryl hydrocarbon receptor nuclear translocator 2
FGF19	fibroblast growth factor 19

SPON2	spondin 2
OLFM1	olfactomedin 1
BAIAP2	BAI1 associated protein 2
SEMA4F	ssemaphorin 4F
DPYSL4	dihydropyrimidinase like 4
AVIL	advillin
TBR1	T-box, brain 1
CYP46A1	cytochrome P450 family 46 subfamily A member 1
GPR45	G protein-coupled receptor 45
POU6F2	POU class 6 homeobox 2
NTNG1	netrin G1
NLGN1	neuroligin 1
MYO16	myosin XVI
CYFIP1	cytoplasmic FMR1 interacting protein 1
ATXN10	ataxin 10
PHGDH	phosphoglycerate dehydrogenase
LHX6	LIM homeobox 6
NPTN	neuroplastin
RND1	Rho family GTPase 1
MYLIP	myosin regulatory light chain interacting protein
TMOD2	tropomodulin 2
SOX8	SRY-box 8
STMN3	stathmin 3
UTP11	UTP11, small subunit processome component homolog (S. cerevisiae)
CRIM1	cysteine rich transmembrane BMP regulator 1 (chordin-like)
TRAPPC4	trafficking protein particle complex 4
HPCAL4	hippocalcin like 4
SNTG2	syntrophin gamma 2
PCDH18	protocadherin 18
MAP1S	microtubule associated protein 1S
MAML3	mastermind like transcriptional coactivator 3
PCDHB13	protocadherin beta 13
PCDHB12	protocadherin beta 12
PCDHB4	protocadherin beta 4
PCDHB3	protocadherin beta 3
PCDHA1	protocadherin alpha 1
PARD3	par-3 family cell polarity regulator
RTN4	reticulon 4
DSCAML1	DS cell adhesion molecule like 1
AMIGO1	adhesion molecule with Ig-like domain 1
LRRC4C	leucine rich repeat containing 4C
PCDHB16	protocadherin beta 16
MARK4	microtubule affinity regulating kinase 4
PARD6B	par-6 family cell polarity regulator beta
NTNG2	netrin G2
FOXP2	forkhead box P2
DMBX1	diencephalon/mesencephalon homeobox 1
RTN4RL1	reticulon 4 receptor like 1
CNTN4	contactin 4
MDGA2	MAM domain containing glycosylphosphatidylinositol anchor 2
ZNF384	zinc finger protein 384
MDGA1	MAM domain containing glycosylphosphatidylinositol anchor 1
RTN4RL2	reticulon 4 receptor-like 2
AGRN	agrin
**Cell development**	
**Gene Symbol**	**Name Gene**
ACTA1	actin, alpha 1, skeletal muscle

ADRA1A	adrenoceptor alpha 1A
ANXA1	annexin A1
APAF1	apoptotic peptidase activating factor 1
BIRC3	baculoviral IAP repeat containing 3
BIRC5	baculoviral IAP repeat containing 5
FASLG	Fas ligand
ARHGDIA	Rho GDP dissociation inhibitor alpha
ATP2B2	ATPase plasma membrane Ca2+ transporting 2
AZU1	azurocidin 1
BARD1	BRCA1 associated RING domain 1
BCL2	B-cell CLL/lymphoma 2
BCL6	B-cell CLL/lymphoma 6
BMP2	bone morphogenetic protein 2
BMP7	bone morphogenetic protein 7
BNIP2	BCL2/adenovirus E1B 19kDa interacting protein 2
BNIP3	BCL2/adenovirus E1B 19kDa interacting protein 3
BOK	BCL2-related ovarian killer
FOXL2	forkhead box L2
BRCA1	breast cancer 1
CACNA1A	calcium voltage-gated channel subunit alpha1 A
CACNB2	calcium voltage-gated channel auxiliary subunit beta 2
CASP3	caspase 3
RUNX3	runt related transcription factor 3
CD2	CD2 molecule
CD28	CD28 molecule
TNFRSF8	tumor necrosis factor receptor superfamily member 8
TNFSF8	tumor necrosis factor superfamily member 8
CD38	CD38 molecule
CD70	CD70 molecule
CD74	CD74 molecule
CDH13	cadherin 13
CDK6	cyclin-dependent kinase 6
CDKN1B	cyclin-dependent kinase inhibitor 1B
CHRNA1	cholinergic receptor nicotinic alpha 1 subunit
CRYAA	crystallin alpha A
DAD1	defender against cell death 1
DAP	death-associated protein
DAPK3	death-associated protein kinase 3
DAXX	death-domain associated protein
GADD45A	growth arrest and DNA damage inducible alpha
DOCK1	dedicator of cytokinesis 1
DTX1	deltex 1
ERCC2	excision repair cross-complementation group 2
ERCC3	excision repair cross-complementation group 3
F2R	coagulation factor II thrombin receptor
PTK2B	protein tyrosine kinase 2 beta
FOXO1	forkhead box O1
FOXO3	forkhead box O3
IFI6	interferon alpha inducible protein 6
GDNF	glial cell derived neurotrophic factor
GLI2	GLI family zinc finger 2
GRIK2	glutamate ionotropic receptor kainate type subunit 2
GRM4	glutamate metabotropic receptor 4
HDAC1	histone deacetylase 1
HGF	hepatocyte growth factor
HIP1	huntingtin interacting protein 1
HMGB1	high mobility group box 1
HNRNPAB	heterogeneous nuclear ribonucleoprotein A/B

HSPA2	heat shock protein family A (Hsp70) member 2
IGF1R	insulin like growth factor 1 receptor
IGFBP3	insulin like growth factor binding protein 3
IL2RA	interleukin 2 receptor subunit alpha
IL6	interleukin 6
IL7	interleukin 7
LCK	LCK proto-oncogene, Src family tyrosine kinase
LMX1B	LIM homeobox transcription factor 1 beta
LRMP	lymphoid restricted membrane protein
SMAD3	SMAD family member 3
MAL	mal, T-cell differentiation protein
MAPT	microtubule associated protein tau
MBNL1	muscleblind like splicing regulator 1
MDM4	MDM4, p53 regulator
MPO	myeloperoxidase
MX1	MX dynamin like GTPase 1
MYH11	myosin, heavy chain 11, smooth muscle
NF1	neurofibromin 1
NF2	neurofibromin 2 (merlin)
NFKBIA	NFKB inhibitor alpha
NOTCH1	notch 1
NRCAM	neuronal cell adhesion molecule
NRL	neural retina leucine zipper
OPA1	OPA1, mitochondrial dynamin like GTPase
SERPINB2	serpin family B member 2
PAWR	pro-apoptotic WT1 regulator
PAX2	paired box 2
PAX7	paired box 7
PDCD1	programmed cell death 1
PIM1	Pim-1 proto-oncogene, serine/threonine kinase
PIK3R2	phosphoinositide-3-kinase regulatory subunit 2
PLAGL1	PLAG1 like zinc finger 1
PLG	plasminogen
PMAIP1	phorbol-12-myristate-13-acetate-induced protein 1
SRGN	serglycin
PRKCA	protein kinase C alpha
PRKCE	protein kinase C epsilon
PRKCZ	protein kinase C zeta
PRL	prolactin
PROC	protein C, inactivator of coagulation factors Va and VIIIa
PSEN2	presenilin 2
PTGER3	prostaglandin E receptor 3
PTPN6	protein tyrosine phosphatase, non-receptor type 6
RASA1	RAS p21 protein activator 1
DPF2	double PHD fingers 2
ROBO1	roundabout guidance receptor 1
ROBO2	roundabout guidance receptor 2
RTKN	rhotekin
S100B	S100 calcium binding protein B
MAPK12	mitogen-activated protein kinase 12
SFRP1	secreted frizzled related protein 1
SHH	sonic hedgehog
SIAH1	siah E3 ubiquitin protein ligase 1
SLIT1	slit guidance ligand 1
SNCA	synuclein alpha
SSTR3	somatostatin receptor 3
STAT1	signal transducer and activator of transcription 1
STK3	serine/threonine kinase 3

TBX5	T-box 5
TBX3	T-box 3
TGFB1	transforming growth factor beta 1
TIAL1	TIA1 cytotoxic granule-associated RNA binding protein-like 1
TNP1	transition protein 1
TOP2A	topoisomerase (DNA) II alpha
TP53BP2	tumor protein p53 binding protein 2
TP73	tumor protein p73
TPD52L1	tumor protein D52-like 1
TRAF3	TNF receptor associated factor 3
UBB	ubiquitin B
VDAC1	voltage dependent anion channel 1
YWHAG	tyrosine 3-monooxygenase/tryptophan 5-monooxygenase activation protein gamma
YWHAZ	tyrosine 3-monooxygenase/tryptophan 5-monooxygenase activation protein zeta
LST1	leukocyte specific transcript 1
ADAM12	ADAM metallopeptidase domain 12
AXIN1	axin 1
TNFRSF25	tumor necrosis factor receptor superfamily member 25
CRADD	CASP2 and RIPK1 domain containing adaptor with death domain
HRK	harakiri, BCL2 interacting protein
TNFSF14	tumor necrosis factor superfamily member 14
TNFSF9	tumor necrosis factor superfamily member 9
TNFRSF18	tumor necrosis factor receptor superfamily member 18
TNFRSF10D	tumor necrosis factor receptor superfamily member 10d
NRP2	neuropilin 2
NRP1	neuropilin 1
SOCS2	suppressor of cytokine signaling 2
CFLAR	CASP8 and FADD like apoptosis regulator
IER3	immediate early response 3
NAE1	NEDD8 activating enzyme E1 subunit 1
EIF2B3	eukaryotic translation initiation factor 2B subunit gamma
EIF2B2	eukaryotic translation initiation factor 2B subunit beta
CACNA1H	calcium voltage-gated channel subunit alpha1 H
TNFSF18	tumor necrosis factor superfamily member 18
SOCS3	suppressor of cytokine signaling 3
BRSK2	BR serine/threonine kinase 2
DEDD	death effector domain containing
STK17A	serine/threonine kinase 17a
SLIT2	slit guidance ligand 2
NRXN3	neurexin 3
NRXN1	neurexin 1
NCR1	natural cytotoxicity triggering receptor 1
BRE	brain and reproductive organ-expressed (TNFRSF1A modulator)
RNF7	ring finger protein 7
CEP57	centrosomal protein 57
BCL2L11	BCL2 like 11
DNAJB6	DnaJ heat shock protein family (Hsp40) member B6
HIPK3	homeodomain interacting protein kinase 3
MAEA	macrophage erythroblast attacher
SPON2	spondin 2
BAIAP2	BAI1 associated protein 2
CIB1	calcium and integrin binding 1
DEAF1	DEAF1, transcription factor
SIVA1	SIVA1 apoptosis inducing factor
RRAGA	Ras related GTP binding A
CHEK2	checkpoint kinase 2

POU6F2	POU class 6 homeobox 2
NTNG1	netrin G1
NLGN1	neuroligin 1
CYFIP1	cytoplasmic FMR1 interacting protein 1
DDAH2	dimethylarginine dimethylaminohydrolase 2
ZNF346	zinc finger protein 346
PPP1R15A	protein phosphatase 1 regulatory subunit 15A
CADM1	cell adhesion molecule 1
TNFAIP8	TNF alpha induced protein 8
AATF	apoptosis antagonizing transcription factor
CYFIP2	cytoplasmic FMR1 interacting protein 2
NPTN	neuroplastin
CIDEB	cell death-inducing DFFA-like effector b
CECR2	CECR2, histone acetyl-lysine reader
SH3GLB1	SH3 domain containing GRB2 like endophilin B1
UTP11	UTP11, small subunit processome component homolog (S. cerevisiae)
BFAR	bifunctional apoptosis regulator
TRAPPC4	trafficking protein particle complex 4
DDX41	DEAD-box helicase 41
GULP1	GULP, engulfment adaptor PTB domain containing 1
ZAK	sterile alpha motif and leucine zipper containing kinase AZK
MOV10L1	Mov10 RISC complex RNA helicase like 1
ADAMTSL4	ADAMTS like 4
MAP1S	microtubule associated protein 1S
PARD3	par-3 family cell polarity regulator
RTN4	reticulon 4
AMIGO1	adhesion molecule with Ig-like domain 1
LRRC4C	leucine rich repeat containing 4C
NLRC4	NLR family, CARD domain containing 4
CIDEC	cell death inducing DFFA like effector c
TP53AIP1	tumor protein p53 regulated apoptosis inducing protein 1
PARD6B	par-6 family cell polarity regulator beta
AIFM2	apoptosis inducing factor, mitochondria associated 2
BMF	Bcl2 modifying factor
BOC	BOC cell adhesion associated, oncogene regulated
NLRP3	NLR family, pyrin domain containing 3
IKBIP	IKBKB interacting protein
JMY	junction mediating and regulatory protein, p53 cofactor
RTN4RL1	reticulon 4 receptor like 1
CNTN4	contactin 4
PRUNE2	prune homolog 2 (Drosophila)
MDGA2	MAM domain containing glycosylphosphatidylinositol anchor 2
MDGA1	MAM domain containing glycosylphosphatidylinositol anchor 1
RTN4RL2	reticulon 4 receptor-like 2
DNAJB13	DnaJ heat shock protein family (Hsp40) member B13
AGRN	agrin
MYO18A	myosin XVIIIA
**Regulation developmental process**	
**Gene Symbol**	**Name Gene**
ANXA1	annexin A1
APAF1	apoptotic peptidase activating factor 1
BIRC3	baculoviral IAP repeat containing 3
BIRC5	baculoviral IAP repeat containing 5
FASLG	Fas ligand
ARHGDIA	Rho GDP dissociation inhibitor alpha
BCL2	B-cell CLL/lymphoma 2
BCL6	B-cell CLL/lymphoma 6

BNIP2	BCL2/adenovirus E1B 19kDa interacting protein 2
BNIP3	BCL2/adenovirus E1B 19kDa interacting protein 3
BOK	BCL2-related ovarian killer
FOXL2	forkhead box L2
BRCA1	breast cancer 1
CALCA	calcitonin related polypeptide alpha
CASP3	caspase 3
RUNX1	runt related transcription factor 1
RUNX3	runt related transcription factor 3
CD2	CD2 molecule
CD28	CD28 molecule
TNFRSF8	tumor necrosis factor receptor superfamily member 8
TNFSF8	tumor necrosis factor superfamily member 8
CD38	CD38 molecule
CD70	CD70 molecule
CD74	CD74 molecule
CDH13	cadherin 13
CDK6	cyclin-dependent kinase 6
CDKN1B	cyclin-dependent kinase inhibitor 1B
COL4A2	collagen type IV alpha 2
CRYAA	crystallin alpha A
DAD1	defender against cell death 1
DAP	death-associated protein
DAPK3	death-associated protein kinase 3
DAXX	death-domain associated protein
DTX1	deltex 1
ERCC3	excision repair cross-complementation group 3
ETS1	ETS proto-oncogene 1, transcription factor
F2R	coagulation factor II thrombin receptor
FOXO1	forkhead box O1
FOXO3	forkhead box O3
IFI6	interferon alpha inducible protein 6
GDNF	glial cell derived neurotrophic factor
GRM4	glutamate metabotropic receptor 4
HDAC1	histone deacetylase 1
HIP1	huntingtin interacting protein 1
HMGB1	high mobility group box 1
IGF1R	insulin like growth factor 1 receptor
IGFBP3	insulin like growth factor binding protein 3
IL6	interleukin 6
IL7	interleukin 7
LCK	LCK proto-oncogene, Src family tyrosine kinase
SMAD3	SMAD family member 3
MAPT	microtubule associated protein tau
MPO	myeloperoxidase
MX1	MX dynamin like GTPase 1
MYH9	myosin, heavy chain 9, non-muscle
NF1	neurofibromin 1
NOTCH1	notch 1
NOTCH4	notch 4
NPR1	natriuretic peptide receptor 1
OPA1	OPA1, mitochondrial dynamin like GTPase
PAX7	paired box 7
PF4	platelet factor 4
PIM1	Pim-1 proto-oncogene, serine/threonine kinase
PIK3R2	phosphoinositide-3-kinase regulatory subunit 2
PLAGL1	PLAG1 like zinc finger 1
PLG	plasminogen

PMAIP1	phorbol-12-myristate-13-acetate-induced protein 1
PPARG	peroxisome proliferator activated receptor gamma
SRGN	serglycin
PRKCA	protein kinase C alpha
PRKCE	protein kinase C epsilon
PRKCZ	protein kinase C zeta
PROC	protein C, inactivator of coagulation factors Va and VIIIa
RASA1	RAS p21 protein activator 1
DPF2	double PHD fingers 2
RNH1	ribonuclease/angiogenin inhibitor 1
ROBO1	roundabout guidance receptor 1
ROBO2	roundabout guidance receptor 2
RTKN	rhotekin
SFRP1	secreted frizzled related protein 1
SHH	sonic hedgehog
SNCA	synuclein alpha
SPI1	Spi-1 proto-oncogene
SSTR3	somatostatin receptor 3
STAT1	signal transducer and activator of transcription 1
STK3	serine/threonine kinase 3
TBX5	T-box 5
TBX3	T-box 3
TGFB1	transforming growth factor beta 1
TIAL1	TIA1 cytotoxic granule-associated RNA binding protein-like 1
TOP2A	topoisomerase (DNA) II alpha
TP53BP2	tumor protein p53 binding protein 2
TP73	tumor protein p73
TPD52L1	tumor protein D52-like 1
TRAF3	TNF receptor associated factor 3
YWHAG	tyrosine 3-monooxygenase/tryptophan 5-monooxygenase activation protein gamma
YWHAZ	tyrosine 3-monooxygenase/tryptophan 5-monooxygenase activation protein zeta
ZAP70	zeta chain of T cell receptor associated protein kinase 70kDa
ZBTB16	zinc finger and BTB domain containing 16
TNFRSF25	tumor necrosis factor receptor superfamily member 25
CRADD	CASP2 and RIPK1 domain containing adaptor with death domain
HRK	harakiri, BCL2 interacting protein
TNFSF14	tumor necrosis factor superfamily member 14
TNFRSF18	tumor necrosis factor receptor superfamily member 18
TNFRSF10D	tumor necrosis factor receptor superfamily member 10d
CFLAR	CASP8 and FADD like apoptosis regulator
IER3	immediate early response 3
NAE1	NEDD8 activating enzyme E1 subunit 1
TNFSF18	tumor necrosis factor superfamily member 18
SOCS3	suppressor of cytokine signaling 3
SART1	squamous cell carcinoma antigen recognized by T-cells 1
DEDD	death effector domain containing
SLIT2	slit guidance ligand 2
KL	klotho
NCR1	natural cytotoxicity triggering receptor 1
BRE	brain and reproductive organ-expressed (TNFRSF1A modulator)
RNF7	ring finger protein 7
HDAC4	histone deacetylase 4
HDAC5	histone deacetylase 5
BCL2L11	BCL2 like 11
DNAJB6	DnaJ heat shock protein family (Hsp40) member B6
HIPK3	homeodomain interacting protein kinase 3

CDC42EP2	CDC42 effector protein 2
SIVA1	SIVA1 apoptosis inducing factor
RRAGA	Ras related GTP binding A
CHEK2	checkpoint kinase 2
NLGN1	neuroligin 1
STAB1	stabilin 1
DDAH2	dimethylarginine dimethylaminohydrolase 2
CADM1	cell adhesion molecule 1
TNFAIP8	TNF alpha induced protein 8
AATF	apoptosis antagonizing transcription factor
NPTN	neuroplastin
CIDEB	cell death-inducing DFFA-like effector b
IL20	interleukin 20
SH3GLB1	SH3 domain containing GRB2 like endophilin B1
UTP11	UTP11, small subunit processome component homolog (S. cerevisiae)
BFAR	bifunctional apoptosis regulator
ZAK	sterile alpha motif and leucine zipper containing kinase AZK
ADAMTSL4	ADAMTS like 4
AGGF1	angiogenic factor with G-patch and FHA domains 1
FGD6	FYVE, RhoGEF and PH domain containing 6
RTN4	reticulon 4
AMIGO1	adhesion molecule with Ig-like domain 1
LRRC4C	leucine rich repeat containing 4C
NLRC4	NLR family, CARD domain containing 4
CIDEC	cell death inducing DFFA like effector c
ARAP3	ArfGAP with RhoGAP domain, ankyrin repeat and PH domain 3
AIFM2	apoptosis inducing factor, mitochondria associated 2
BOC	BOC cell adhesion associated, oncogene regulated
NLRP3	NLR family, pyrin domain containing 3
ARAP1	ArfGAP with RhoGAP domain, ankyrin repeat and PH domain 1
IKBIP	IKBKB interacting protein
JMY	junction mediating and regulatory protein, p53 cofactor
SIK1	salt inducible kinase 1
FGD5	FYVE, RhoGEF and PH domain containing 5
CNTN4	contactin 4
PRUNE2	prune homolog 2 (Drosophila)
FGD2	FYVE, RhoGEF and PH domain containing 2
IL27	interleukin 27
MYO18A	myosin XVIIIA
**Generation of neurons**	
**Gene Symbol**	**Name Gene**
ATP2B2	ATPase plasma membrane Ca2+ transporting 2
DTX1	deltex 1
GDNF	glial cell derived neurotrophic factor
GLI2	GLI family zinc finger 2
LMX1B	LIM homeobox transcription factor 1 beta
MAPT	microtubule associated protein tau
NF1	neurofibromin 1
NRCAM	neuronal cell adhesion molecule
PAX2	paired box 2
ROBO1	roundabout guidance receptor 1
ROBO2	roundabout guidance receptor 2
S100B	S100 calcium binding protein B
SHH	sonic hedgehog
SIAH1	siah E3 ubiquitin protein ligase 1
SLIT1	slit guidance ligand 1
UBB	ubiquitin B

YWHAG	tyrosine 3-monooxygenase/tryptophan 5-monooxygenase activation protein gamma
LST1	leukocyte specific transcript 1
NRP2	neuropilin 2
NRP1	neuropilin 1
BRSK2	BR serine/threonine kinase 2
SLIT2	slit guidance ligand 2
NRXN3	neurexin 3
NRXN1	neurexin 1
SPON2	spondin 2
BAIAP2	BAI1 associated protein 2
POU6F2	POU class 6 homeobox 2
NTNG1	netrin G1
NLGN1	neuroligin 1
CYFIP1	cytoplasmic FMR1 interacting protein 1
NPTN	neuroplastin
TRAPPC4	trafficking protein particle complex 4
MAP1S	microtubule associated protein 1S
PARD3	par-3 family cell polarity regulator
RTN4	reticulon 4
AMIGO1	adhesion molecule with Ig-like domain 1
LRRC4C	leucine rich repeat containing 4C
PARD6B	par-6 family cell polarity regulator beta
RTN4RL1	reticulon 4 receptor like 1
CNTN4	contactin 4
MDGA2	MAM domain containing glycosylphosphatidylinositol anchor 2
MDGA1	MAM domain containing glycosylphosphatidylinositol anchor 1
RTN4RL2	reticulon 4 receptor-like 2
AGRN	agrin
**Neuron differentiation**	
**Gene Symbol**	**Name Gene**
ATP2B2	ATPase plasma membrane Ca2+ transporting 2
DTX1	deltex 1
GDNF	glial cell derived neurotrophic factor
GLI2	GLI family zinc finger 2
LMX1B	LIM homeobox transcription factor 1 beta
MAPT	microtubule associated protein tau
NRCAM	neuronal cell adhesion molecule
PAX2	paired box 2
ROBO1	roundabout guidance receptor 1
ROBO2	roundabout guidance receptor 2
S100B	S100 calcium binding protein B
SHH	sonic hedgehog
SIAH1	siah E3 ubiquitin protein ligase 1
SLIT1	slit guidance ligand 1
UBB	ubiquitin B
YWHAG	tyrosine 3-monooxygenase/tryptophan 5-monooxygenase activation protein gamma
LST1	leukocyte specific transcript 1
NRP2	neuropilin 2
NRP1	neuropilin 1
BRSK2	BR serine/threonine kinase 2
SLIT2	slit guidance ligand 2
NRXN3	neurexin 3
NRXN1	neurexin 1
SPON2	spondin 2
BAIAP2	BAI1 associated protein 2
POU6F2	POU class 6 homeobox 2

NTNG1	netrin G1
NLGN1	neuroligin 1
CYFIP1	cytoplasmic FMR1 interacting protein 1
TRAPPC4	trafficking protein particle complex 4
MAP1S	microtubule associated protein 1S
PARD3	par-3 family cell polarity regulator
RTN4	reticulon 4
AMIGO1	adhesion molecule with Ig-like domain 1
LRRC4C	leucine rich repeat containing 4C
PARD6B	par-6 family cell polarity regulator beta
RTN4RL1	reticulon 4 receptor like 1
CNTN4	contactin 4
MDGA2	MAM domain containing glycosylphosphatidylinositol anchor 2
MDGA1	MAM domain containing glycosylphosphatidylinositol anchor 1
RTN4RL2	reticulon 4 receptor-like 2
AGRN	agrin
**Neurogenesis**	
**Gene Symbol**	**Name Gene**
ATP2B2	ATPase plasma membrane Ca2+ transporting 2
AZU1	azurocidin 1
CDK6	cyclin-dependent kinase 6
DTX1	deltex 1
GDNF	glial cell derived neurotrophic factor
GLI2	GLI family zinc finger 2
LMX1B	LIM homeobox transcription factor 1 beta
MAPT	microtubule associated protein tau
NF1	neurofibromin 1
NF2	neurofibromin 2 (merlin)
NRCAM	neuronal cell adhesion molecule
PAX2	paired box 2
ROBO1	roundabout guidance receptor 1
ROBO2	roundabout guidance receptor 2
S100B	S100 calcium binding protein B
SHH	sonic hedgehog
SIAH1	siah E3 ubiquitin protein ligase 1
SLIT1	slit guidance ligand 1
UBB	ubiquitin B
YWHAG	tyrosine 3-monooxygenase/tryptophan 5-monooxygenase activation protein gamma
LST1	leukocyte specific transcript 1
NRP2	neuropilin 2
NRP1	neuropilin 1
EIF2B3	eukaryotic translation initiation factor 2B subunit gamma
EIF2B2	eukaryotic translation initiation factor 2B subunit beta
BRSK2	BR serine/threonine kinase 2
SLIT2	slit guidance ligand 2
NRXN3	neurexin 3
NRXN1	neurexin 1
SPON2	spondin 2
BAIAP2	BAI1 associated protein 2
POU6F2	POU class 6 homeobox 2
NTNG1	netrin G1
NLGN1	neuroligin 1
CYFIP1	cytoplasmic FMR1 interacting protein 1
NPTN	neuroplastin
TRAPPC4	trafficking protein particle complex 4
MAP1S	microtubule associated protein 1S
PARD3	par-3 family cell polarity regulator

RTN4	reticulon 4
AMIGO1	adhesion molecule with Ig-like domain 1
LRRC4C	leucine rich repeat containing 4C
PARD6B	par-6 family cell polarity regulator beta
RTN4RL1	reticulon 4 receptor like 1
CNTN4	contactin 4
MDGA2	MAM domain containing glycosylphosphatidylinositol anchor 2
MDGA1	MAM domain containing glycosylphosphatidylinositol anchor 1
RTN4RL2	reticulon 4 receptor-like 2
AGRN	agrin
**Neuron development**	
**Gene Symbol**	**Name Gene**
GDNF	glial cell derived neurotrophic factor
GLI2	GLI family zinc finger 2
MAPT	microtubule associated protein tau
NRCAM	neuronal cell adhesion molecule
PAX2	paired box 2
ROBO1	roundabout guidance receptor 1
ROBO2	roundabout guidance receptor 2
S100B	S100 calcium binding protein B
SHH	sonic hedgehog
SIAH1	siah E3 ubiquitin protein ligase 1
SLIT1	slit guidance ligand 1
LST1	leukocyte specific transcript 1
NRP2	neuropilin 2
NRP1	neuropilin 1
SLIT2	slit guidance ligand 2
NRXN3	neurexin 3
NRXN1	neurexin 1
SPON2	spondin 2
BAIAP2	BAI1 associated protein 2
NTNG1	netrin G1
CYFIP1	cytoplasmic FMR1 interacting protein 1
TRAPPC4	trafficking protein particle complex 4
MAP1S	microtubule associated protein 1S
PARD3	par-3 family cell polarity regulator
RTN4	reticulon 4
AMIGO1	adhesion molecule with Ig-like domain 1
LRRC4C	leucine rich repeat containing 4C
PARD6B	par-6 family cell polarity regulator beta
RTN4RL1	reticulon 4 receptor like 1
CNTN4	contactin 4
RTN4RL2	reticulon 4 receptor-like 2
AGRN	agrin

Table S3 - Gene collection of Persistent (cases) group

**Table S3** - Gene collection of Persistent group	
**Biological Processes**	
**Regulation molecular function**	
**Gene Symbol**	**Name Gene**
ABL2	ABL proto-oncogene 2, non-receptor tyrosine kinase
ADRA2A	adrenoceptor alpha 2A
ADRB1	adrenoceptor beta 1
ANG	angiogenin
APAF1	apoptotic peptidase activating factor 1
APC	adenomatous polyposis coli
BIRC5	baculoviral IAP repeat containing 5
BAK1	BCL2 antagonist/killer 1
CCND1	cyclin D1
BCL2	B-cell CLL/lymphoma 2
BCL3	B-cell CLL/lymphoma 3
FOXL2	forkhead box L2
CALCA	calcitonin related polypeptide alpha
CALCR	calcitonin receptor
CASP9	caspase 9
CCKBR	cholecystokinin B receptor
CCND2	cyclin D2
CCNG1	cyclin G1
CD81	CD81 molecule
CDK7	cyclin-dependent kinase 7
CDKN1C	cyclin-dependent kinase inhibitor 1C
CDKN3	cyclin-dependent kinase inhibitor 3
CHRM1	cholinergic receptor muscarinic 1
CKS1B	CDC28 protein kinase regulatory subunit 1B
CRHR1	corticotropin releasing hormone receptor 1
DAXX	death-domain associated protein
GADD45A	growth arrest and DNA damage inducible alpha
DHCR24	24-dehydrocholesterol reductase
DUSP2	dual specificity phosphatase 2
DUSP6	dual specificity phosphatase 6
EDNRB	endothelin receptor type B
EGF	epidermal growth factor
EGFR	epidermal growth factor receptor
EP300	E1A binding protein p300
GABBR1	gamma-aminobutyric acid type B receptor subunit 1
GALR1	galanin receptor 1
GHRHR	growth hormone releasing hormone receptor
GIPR	gastric inhibitory polypeptide receptor
GNA15	G protein subunit alpha 15
GNAI2	G protein subunit alpha i2
GNAS	GNAS complex locus
GPS1	G protein pathway suppressor 1
GRM2	glutamate metabotropic receptor 2
GRM3	glutamate metabotropic receptor 3
GRM4	glutamate metabotropic receptor 4
GTF2H1	general transcription factor IIH subunit 1
HIP1	huntingtin interacting protein 1
ID2	inhibitor of DNA binding 2, HLH protein
ID3	inhibitor of DNA binding 3, HLH protein
IKBKB	inhibitor of kappa light polypeptide gene enhancer in B-cells, kinase beta

LCK	LCK proto-oncogene, Src family tyrosine kinase
SMAD3	SMAD family member 3
MAP3K5	mitogen-activated protein kinase kinase kinase 5
MAP3K10	mitogen-activated protein kinase kinase kinase 10
NF1	neurofibromin 1
PAK1	p21 (RAC1) activated kinase 1
PAK2	p21 (RAC1) activated kinase 2
PEX14	peroxisomal biogenesis factor 14
PIK3CB	phosphatidylinositol-4,5-bisphosphate 3-kinase catalytic subunit beta
PMAIP1	phorbol-12-myristate-13-acetate-induced protein 1
PRLR	prolactin receptor
PTEN	phosphatase and tensin homolog
PTPRC	protein tyrosine phosphatase, receptor type C
RB1	retinoblastoma 1
RGS3	regulator of G-protein signaling 3
SHC1	SHC adaptor protein 1
TARBP2	TARBP2, RISC loading complex RNA binding subunit
TGFA	transforming growth factor alpha
TGFB1	transforming growth factor beta 1
TNF	tumor necrosis factor
TNNT2	troponin T2, cardiac type
TPD52L1	tumor protein D52-like 1
TRAF6	TNF receptor associated factor 6
TSC1	tuberous sclerosis 1
MADD	MAP kinase activating death domain
PKMYT1	protein kinase, membrane associated tyrosine/threonine 1
CCNE2	cyclin E2
LPAR2	lysophosphatidic acid receptor 2
MAP3K13	mitogen-activated protein kinase kinase kinase 13
PICK1	protein interacting with PRKCA 1
GABBR2	gamma-aminobutyric acid type B receptor subunit 2
CASP8AP2	caspase 8 associated protein 2
DNAJB6	DnaJ heat shock protein family (Hsp40) member B6
HIPK3	homeodomain interacting protein kinase 3
TRIB1	tribbles pseudokinase 1
NMUR1	neuromedin U receptor 1
TAB1	TGF-beta activated kinase 1/MAP3K7 binding protein 1
CAP1	CAP, adenylate cyclase-associated protein 1 (yeast)
CAMKK2	calcium/calmodulin-dependent protein kinase kinase 2
GADD45G	growth arrest and DNA damage inducible gamma
RALBP1	ralA binding protein 1
ADAP1	ArfGAP with dual PH domains 1
CDC37	cell division cycle 37
ERC1	ELKS/RAB6-interacting/CAST family member 1
CBLC	Cbl proto-oncogene C
FBXO5	F-box protein 5
LATS2	large tumor suppressor kinase 2
SERTAD1	SERTA domain containing 1
MDFIC	MyoD family inhibitor domain containing
MBIP	MAP3K12 binding inhibitory protein 1
ZAK	sterile alpha motif and leucine zipper containing kinase AZK
EGLN1	egl-9 family hypoxia inducible factor 1
IFT57	intraflagellar transport 57
CAND1	cullin associated and neddylation dissociated 1
PSENEN	presenilin enhancer gamma-secretase subunit
PARD3	par-3 family cell polarity regulator
PRMT8	protein arginine methyltransferase 8
LTB4R2	leukotriene B4 receptor 2

BCCIP	BRCA2 and CDKN1A interacting protein
DUSP22	dual specificity phosphatase 22
ALS2	ALS2, alsin Rho guanine nucleotide exchange factor
SIGIRR	single immunoglobulin and toll-interleukin 1 receptor (TIR) domain
NARFL	nuclear prelamin A recognition factor like
PIF1	PIF1 5'-to-3' DNA helicase
CDK5RAP3	CDK5 regulatory subunit associated protein 3
CARD11	caspase recruitment domain family member 11
ATPIF1	ATPase inhibitory factor 1
NLRP3	NLR family, pyrin domain containing 3
ARAP1	ArfGAP with RhoGAP domain, ankyrin repeat and PH domain 1
HEXIM2	hexamethylene bisacetamide inducible 2
COMMD7	COMM domain containing 7
AIFM3	apoptosis inducing factor, mitochondria associated 3
NFAM1	NFAT activating protein with ITAM motif 1
FGD5	FYVE, RhoGEF and PH domain containing 5
SPRED2	sprouty related EVH1 domain containing 2
ARHGAP27	Rho GTPase activating protein 27
FGD2	FYVE, RhoGEF and PH domain containing 2
CRIPAK	cysteine rich PAK1 inhibitor
SERINC2	serine incorporator 2
**Signal transduction**	
**Gene Symbol**	**Name Gene**
ABL2	ABL proto-oncogene 2, non-receptor tyrosine kinase
ABR	active BCR-related
ACTL6A	actin like 6A
ACVR1	activin A receptor type 1
ACVR2A	activin A receptor type 2A
ACVR2B	activin A receptor type 2B
ACVRL1	activin A receptor like type 1
ADCYAP1	adenylate cyclase activating polypeptide 1
ADM	adrenomedullin
ADORA1	adenosine A1 receptor
ADORA3	adenosine A3 receptor
ADRA1A	adrenoceptor alpha 1A
ADRA2A	adrenoceptor alpha 2A
ADRA2C	adrenoceptor alpha 2C
ADRB1	adrenoceptor beta 1
GRK2	G protein-coupled receptor kinase 2
GRK3	G protein-coupled receptor kinase 3
AKT1	AKT serine/threonine kinase 1
ALCAM	activated leukocyte cell adhesion molecule
AMHR2	anti-Mullerian hormone receptor type 2
ANG	angiogenin
ANXA1	annexin A1
ANXA3	annexin A3
ANXA4	annexin A4
ANXA5	annexin A5
APAF1	apoptotic peptidase activating factor 1
BIRC3	baculoviral IAP repeat containing 3
FASLG	Fas ligand
RHOA	ras homolog family member A
RHOB	ras homolog family member B
RHOG	ras homolog family member G
ARHGAP1	Rho GTPase activating protein 1
ARHGAP5	Rho GTPase activating protein 5
ARHGDIA	Rho GDP dissociation inhibitor alpha

ARHGDIB	Rho GDP dissociation inhibitor beta
RHOH	ras homolog family member H
ARRB1	arrestin beta 1
ASIP	agouti signaling protein
ATR	ATR serine/threonine kinase
AXL	AXL receptor tyrosine kinase
BAK1	BCL2 antagonist/killer 1
BCL3	B-cell CLL/lymphoma 3
BID	BH3 interacting domain death agonist
BIK	BCL2 interacting killer
BLK	BLK proto-oncogene, Src family tyrosine kinase
BMPR1A	bone morphogenetic protein receptor type 1A
BMPR2	bone morphogenetic protein receptor type 2
BNIP3	BCL2/adenovirus E1B 19kDa interacting protein 3
FOXL2	forkhead box L2
BRCA1	breast cancer 1
BSG	basigin (Ok blood group)
BST2	bone marrow stromal cell antigen 2
C3	complement component 3
CAPN5	calpain 5
CALCA	calcitonin related polypeptide alpha
CALCR	calcitonin receptor
CALM3	calmodulin 3 (phosphorylase kinase, delta)
CAMK2B	calcium/calmodulin dependent protein kinase II beta
CASP8	caspase 8
CASP9	caspase 9
CASR	calcium sensing receptor
CBL	Cbl proto-oncogene
CCKBR	cholecystokinin B receptor
CD3E	CD3e molecule
TNFRSF8	tumor necrosis factor receptor superfamily member 8
CD47	CD47 molecule
CD59	CD59 molecule
CD74	CD74 molecule
CD81	CD81 molecule
CDH13	cadherin 13
CDKN1C	cyclin-dependent kinase inhibitor 1C
CFL1	cofilin 1
CGA	glycoprotein hormones, alpha polypeptide
FOXN3	forkhead box N3
CHRM1	cholinergic receptor muscarinic 1
CHRM2	cholinergic receptor muscarinic 2
LYST	lysosomal trafficking regulator
CHRM3	cholinergic receptor muscarinic 3
CHRM4	cholinergic receptor muscarinic 4
CHRM5	cholinergic receptor muscarinic 5
CHRNA1	cholinergic receptor nicotinic alpha 1 subunit
CHRNA4	cholinergic receptor nicotinic alpha 4 subunit
CHRNB1	cholinergic receptor nicotinic beta 1 subunit
CHRNE	cholinergic receptor nicotinic epsilon subunit
CHUK	conserved helix-loop-helix ubiquitous kinase
CIDEA	cell death-inducing DFFA-like effector a
CLIC1	chloride intracellular channel 1
CCR6	C-C motif chemokine receptor 6
CNGA3	cyclic nucleotide gated channel alpha 3
CNR1	cannabinoid receptor 1 (brain)
CRABP1	cellular retinoic acid binding protein 1
CREM	cAMP responsive element modulator

CRH	corticotropin releasing hormone
CRHBP	corticotropin releasing hormone binding protein
CRHR1	corticotropin releasing hormone receptor 1
CRHR2	corticotropin releasing hormone receptor 2
CRKL	v-crk avian sarcoma virus CT10 oncogene homolog-like
MAPK14	mitogen-activated protein kinase 14
CSNK1D	casein kinase 1 delta
CSNK1E	casein kinase 1 epsilon
CSNK1G2	casein kinase 1 gamma 2
CSNK2A2	casein kinase 2 alpha 2
CSNK2B	casein kinase 2 beta
CTNND2	catenin delta 2
CX3CR1	C-X3-C motif chemokine receptor 1
DGKA	diacylglycerol kinase alpha
DAPK1	death associated protein kinase 1
DAPK3	death-associated protein kinase 3
DAXX	death-domain associated protein
DFFA	DNA fragmentation factor subunit alpha
DFFB	DNA fragmentation factor subunit beta
DLG4	discs large homolog 4
DOCK1	dedicator of cytokinesis 1
DPYSL2	dihydropyrimidinase like 2
DRD1	dopamine receptor D1
DRD2	dopamine receptor D2
DRD4	dopamine receptor D4
DRD5	dopamine receptor D5
RCAN1	regulator of calcineurin 1
DTNA	dystrobrevin alpha
DTX1	deltex 1
DUSP2	dual specificity phosphatase 2
DUSP4	dual specificity phosphatase 4
DUSP6	dual specificity phosphatase 6
ECT2	epithelial cell transforming 2
S1PR1	sphingosine-1-phosphate receptor 1
EDN3	endothelin 3
EDNRB	endothelin receptor type B
EEF1D	eukaryotic translation elongation factor 1 delta
EGF	epidermal growth factor
EGFR	epidermal growth factor receptor
EPHA2	EPH receptor A2
ELK3	ELK3, ETS transcription factor
MARK2	microtubule affinity regulating kinase 2
EPAS1	endothelial PAS domain protein 1
EPHA1	EPH receptor A1
EPHA4	EPH receptor A4
EPHB1	EPH receptor B1
EPHB3	EPH receptor B3
STX2	syntaxin 2
EPS8	epidermal growth factor receptor pathway substrate 8
EPS15	epidermal growth factor receptor pathway substrate 15
NR2F6	nuclear receptor subfamily 2 group F member 6
ERBB2	erb-b2 receptor tyrosine kinase 2
ERBB4	erb-b2 receptor tyrosine kinase 4
ESR1	estrogen receptor 1
EXT1	exostosin glycosyltransferase 1
EXT2	exostosin glycosyltransferase 2
F2RL1	F2R like trypsin receptor 1
PTK2B	protein tyrosine kinase 2 beta

FGF5	fibroblast growth factor 5
FGF9	fibroblast growth factor 9
FGF11	fibroblast growth factor 11
FGF12	fibroblast growth factor 12
FGF14	fibroblast growth factor 14
FGFR1	fibroblast growth factor receptor 1
FGFR3	fibroblast growth factor receptor 3
FGFR4	fibroblast growth factor receptor 4
FOXC2	forkhead box C2
FLT3LG	fms related tyrosine kinase 3 ligand
FLT4	fms related tyrosine kinase 4
FPR2	formyl peptide receptor 2
FPR3	formyl peptide receptor 3
FYB	FYN binding protein
FYN	FYN proto-oncogene, Src family tyrosine kinase
IFI6	interferon alpha inducible protein 6
GABBR1	gamma-aminobutyric acid type B receptor subunit 1
GABRA1	gamma-aminobutyric acid type A receptor alpha1 subunit
GABRB1	gamma-aminobutyric acid type A receptor beta1 subunit
GABRB3	gamma-aminobutyric acid type A receptor beta3 subunit
GABRR1	gamma-aminobutyric acid type A receptor rho1 subunit
GABRR2	gamma-aminobutyric acid type A receptor rho2 subunit
GALR1	galanin receptor 1
GDI2	GDP dissociation inhibitor 2
GDNF	glial cell derived neurotrophic factor
GHRHR	growth hormone releasing hormone receptor
GHSR	growth hormone secretagogue receptor
GIPR	gastric inhibitory polypeptide receptor
GLI2	GLI family zinc finger 2
GLI3	GLI family zinc finger 3
GLRA1	glycine receptor alpha 1
GNA11	G protein subunit alpha 11
GNA12	G protein subunit alpha 12
GNA15	G protein subunit alpha 15
GNAI2	G protein subunit alpha i2
GNAI3	G protein subunit alpha i3
GNAL	G protein subunit alpha L
GNAS	GNAS complex locus
GNB1	G protein subunit beta 1
GNG4	G protein subunit gamma 4
GNG7	G protein subunit gamma 7
GNL1	G protein nucleolar 1 (putative)
SFN	stratifin
GP1BA	glycoprotein Ib platelet alpha subunit
GPR1	G protein-coupled receptor 1
CCR10	C-C motif chemokine receptor 10
GPR19	G protein-coupled receptor 19
GPR20	G protein-coupled receptor 20
GPR27	G protein-coupled receptor 27
GRK5	G protein-coupled receptor kinase 5
MKNK2	MAP kinase interacting serine/threonine kinase 2
GPS1	G protein pathway suppressor 1
GRB2	growth factor receptor bound protein 2
GRB7	growth factor receptor bound protein 7
GRB10	growth factor receptor bound protein 10
RAPGEF1	Rap guanine nucleotide exchange factor 1
GRIA2	glutamate ionotropic receptor AMPA type subunit 2
GRIA4	glutamate ionotropic receptor AMPA type subunit 4

GRID2	glutamate ionotropic receptor delta type subunit 2
GRIK2	glutamate ionotropic receptor kainate type subunit 2
GRIK4	glutamate ionotropic receptor kainate type subunit 4
GRIN2A	glutamate ionotropic receptor NMDA type subunit 2A
GRIN2B	glutamate ionotropic receptor NMDA type subunit 2B
NR3C1	nuclear receptor subfamily 3 group C member 1
GRM2	glutamate metabotropic receptor 2
GRM3	glutamate metabotropic receptor 3
GRM4	glutamate metabotropic receptor 4
GRM6	glutamate metabotropic receptor 6
GRM8	glutamate metabotropic receptor 8
CXCL1	C-X-C motif chemokine ligand 1
GTF2I	general transcription factor IIi
GUCY1A3	guanylate cyclase 1, soluble, alpha 3
GUCY1B3	guanylate cyclase 1, soluble, beta 3
GUCY2D	guanylate cyclase 2D, retinal
HCRTR2	hypocretin receptor 2
HIP1	huntingtin interacting protein 1
HINT1	histidine triad nucleotide binding protein 1
HMGB1	high mobility group box 1
NR4A1	nuclear receptor subfamily 4 group A member 1
HNRNPK	heterogeneous nuclear ribonucleoprotein K
HPGD	hydroxyprostaglandin dehydrogenase 15-(NAD)
PRMT2	protein arginine methyltransferase 2
PRMT1	protein arginine methyltransferase 1
HTR1B	5-hydroxytryptamine receptor 1B
HTR2A	5-hydroxytryptamine receptor 2A
HTR5A	5-hydroxytryptamine receptor 5A
HTR6	5-hydroxytryptamine receptor 6
HTR7	5-hydroxytryptamine receptor 7
HUS1	HUS1 checkpoint clamp component
IFNA2	interferon, alpha 2
IFNAR2	interferon alpha and beta receptor subunit 2
IFNG	interferon, gamma
IFNGR1	interferon gamma receptor 1
IGF1R	insulin like growth factor 1 receptor
IGF2R	insulin like growth factor 2 receptor
IGFALS	insulin like growth factor binding protein acid labile subunit
IGFBP1	insulin like growth factor binding protein 1
IGFBP4	insulin like growth factor binding protein 4
IL1B	interleukin 1 beta
IL5RA	interleukin 5 receptor subunit alpha
IL12A	interleukin 12A
IL12RB1	interleukin 12 receptor subunit beta 1
IL15	interleukin 15
IL15RA	interleukin 15 receptor subunit alpha
IMPA1	inositol monophosphatase 1
ING2	inhibitor of growth family member 2
INHBA	inhibin beta A
INPP4A	inositol polyphosphate-4-phosphatase type I A
INPP5D	inositol polyphosphate-5-phosphatase D
ITPKA	inositol-trisphosphate 3-kinase A
ITPR2	inositol 1,4,5-trisphosphate receptor type 2
ITPR3	inositol 1,4,5-trisphosphate receptor type 3
JAK2	Janus kinase 2
KIT	KIT proto-oncogene receptor tyrosine kinase
STMN1	stathmin 1
LCK	LCK proto-oncogene, Src family tyrosine kinase

LCP2	lymphocyte cytosolic protein 2
LETM1	leucine zipper and EF-hand containing transmembrane protein 1
LGALS1	galectin 1
LIFR	leukemia inhibitory factor receptor alpha
LTA	lymphotoxin alpha
LTB	lymphotoxin beta
LTBR	lymphotoxin beta receptor
SMAD1	SMAD family member 1
SMAD2	SMAD family member 2
SMAD3	SMAD family member 3
SMAD7	SMAD family member 7
MAL	mal, T-cell differentiation protein
MARK1	microtubule affinity regulating kinase 1
MAS1	MAS1 proto-oncogene, G protein-coupled receptor
MC1R	melanocortin 1 receptor
MC2R	melanocortin 2 receptor
MC4R	melanocortin 4 receptor
MCC	mutated in colorectal cancers
ADAM11	ADAM metallopeptidase domain 11
MAP3K5	mitogen-activated protein kinase kinase kinase 5
MGST2	microsomal glutathione S-transferase 2
MAP3K10	mitogen-activated protein kinase kinase kinase 10
NR3C2	nuclear receptor subfamily 3 group C member 2
MPL	MPL proto-oncogene, thrombopoietin receptor
MPP3	membrane palmitoylated protein 3
MST1R	macrophage stimulating 1 receptor
MTNR1A	melatonin receptor 1A
MYO6	myosin VI
PPP1R12B	protein phosphatase 1 regulatory subunit 12B
NBN	nibrin
NEDD9	neural precursor cell expressed, developmentally down-regulated 9
NF1	neurofibromin 1
NF2	neurofibromin 2 (merlin)
NFATC1	nuclear factor of activated T-cells 1
NFKBIB	NFKB inhibitor beta
NMB	neuromedin B
NOTCH1	notch 1
NPR2	natriuretic peptide receptor 2
NPY1R	neuropeptide Y receptor Y1
NRGN	neurogranin
NTF3	neurotrophin 3
NTRK1	neurotrophic receptor tyrosine kinase 1
NTRK2	neurotrophic receptor tyrosine kinase 2
NTRK3	neurotrophic receptor tyrosine kinase 3
ROR2	receptor tyrosine kinase like orphan receptor 2
NTSR1	neurotensin receptor 1 (high affinity)
TNFRSF11B	tumor necrosis factor receptor superfamily member 11b
OPRK1	opioid receptor kappa 1
OPRL1	opioid related nociceptin receptor 1
OPRM1	opioid receptor mu 1
OTX2	orthodenticle homeobox 2
OXT	oxytocin/neurophysin I prepropeptide
P2RY1	purinergic receptor P2Y1
P2RY2	purinergic receptor P2Y2
P2RY6	pyrimidinergic receptor P2Y6
PAFAH1B1	platelet activating factor acetylhydrolase 1b regulatory subunit 1
PAK1	p21 (RAC1) activated kinase 1
PAK2	p21 (RAC1) activated kinase 2

PDE9A	phosphodiesterase 9A
PDGFA	platelet derived growth factor subunit A
PDGFRA	platelet derived growth factor receptor alpha
PDGFRB	platelet derived growth factor receptor beta
PDK1	pyruvate dehydrogenase kinase 1
ENPP2	ectonucleotide pyrophosphatase/phosphodiesterase 2
PECAM1	platelet and endothelial cell adhesion molecule 1
PIK3CB	phosphatidylinositol-4,5-bisphosphate 3-kinase catalytic subunit beta
PIK3CG	phosphatidylinositol-4,5-bisphosphate 3-kinase catalytic subunit gamma
PIK3R1	phosphoinositide-3-kinase regulatory subunit 1
PLA2G1B	phospholipase A2 group IB
PLD1	phospholipase D1
PMAIP1	phorbol-12-myristate-13-acetate-induced protein 1
POMC	proopiomelanocortin
PPARG	peroxisome proliferator activated receptor gamma
PPP5C	protein phosphatase 5 catalytic subunit
PRKAA2	protein kinase AMP-activated catalytic subunit alpha 2
PRKAB2	protein kinase AMP-activated non-catalytic subunit beta 2
PRKACB	protein kinase cAMP-activated catalytic subunit beta
PRKAG1	protein kinase AMP-activated non-catalytic subunit gamma 1
PRKAR2B	protein kinase cAMP-dependent type II regulatory subunit beta
PKN2	protein kinase N2
PRKG1	protein kinase, cGMP-dependent, type I
MAPK1	mitogen-activated protein kinase 1
MAPK10	mitogen-activated protein kinase 10
MAP2K5	mitogen-activated protein kinase kinase 5
THAP12	THAP domain containing 12
PRL	prolactin
PRLR	prolactin receptor
PSEN1	presenilin 1
PSEN2	presenilin 2
PTCH1	patched 1
PTEN	phosphatase and tensin homolog
PTGER3	prostaglandin E receptor 3
PTGER4	prostaglandin E receptor 4
PTH	parathyroid hormone
PTH1R	parathyroid hormone 1 receptor
PTK7	protein tyrosine kinase 7 (inactive)
PTN	pleiotrophin
PTPN11	protein tyrosine phosphatase, non-receptor type 11
PTPRC	protein tyrosine phosphatase, receptor type C
PTPRD	protein tyrosine phosphatase, receptor type D
PTPRF	protein tyrosine phosphatase, receptor type F
PTPRG	protein tyrosine phosphatase, receptor type G
PTPRJ	protein tyrosine phosphatase, receptor type J
PWP2	PWP2 periodic tryptophan protein homolog (yeast)
MAP4K2	mitogen-activated protein kinase kinase kinase kinase 2
RAC1	ras-related C3 botulinum toxin substrate 1 (rho family, small GTP binding protein Rac1)
RALB	RALB Ras like proto-oncogene B
RAN	RAN, member RAS oncogene family
RANBP1	RAN binding protein 1
RAP1A	RAP1A, member of RAS oncogene family
RAP1GAP	RAP1 GTPase activating protein
RAP2A	RAP2A, member of RAS oncogene family
RAP2B	RAP2B, member of RAS oncogene family
RCVRN	recoverin
RELA	RELA proto-oncogene, NF-kB subunit

RET	ret proto-oncogene
RGS2	regulator of G-protein signaling 2
RGS3	regulator of G-protein signaling 3
RGS4	regulator of G-protein signaling 4
RGS12	regulator of G-protein signaling 12
RIT1	Ras like without CAAX 1
RPS6KA1	ribosomal protein S6 kinase A1
RPS6KA2	ribosomal protein S6 kinase A2
RPS6KB2	ribosomal protein S6 kinase B2
RREB1	ras responsive element binding protein 1
RTKN	rhotekin
RSU1	Ras suppressor protein 1
S100A11	S100 calcium binding protein A11
CXCL5	C-X-C motif chemokine ligand 5
SECTM1	secreted and transmembrane 1
MAP2K4	mitogen-activated protein kinase kinase 4
SFRP1	secreted frizzled related protein 1
SFRP4	secreted frizzled related protein 4
SFRP5	secreted frizzled related protein 5
SH3BP2	SH3 domain binding protein 2
SH3GL2	SH3 domain containing GRB2 like 2, endophilin A1
SH3GL3	SH3 domain containing GRB2 like endophilin A3
SHB	SH2 domain containing adaptor protein B
SHC1	SHC adaptor protein 1
SHH	sonic hedgehog
SIAH2	siah E3 ubiquitin protein ligase 2
SLC20A1	solute carrier family 20 member 1
SMPD1	sphingomyelin phosphodiesterase 1
SPG7	SPG7, paraplegin matrix AAA peptidase subunit
SRC	SRC proto-oncogene, non-receptor tyrosine kinase
SRI	sorcin
SRF	serum response factor
SRP72	signal recognition particle 72kDa
SRPK1	SRSF protein kinase 1
SST	somatostatin
SSTR1	somatostatin receptor 1
STC1	stanniocalcin 1
STK4	serine/threonine kinase 4
XCL2	X-C motif chemokine ligand 2
TACR2	tachykinin receptor 2
TACR1	tachykinin receptor 1
TCF7L2	transcription factor 7 like 2
NR2F2	nuclear receptor subfamily 2 group F member 2
TGFA	transforming growth factor alpha
TGFB1	transforming growth factor beta 1
TGFB3	transforming growth factor beta 3
TGFBR3	transforming growth factor beta receptor 3
KLF10	Kruppel-like factor 10
TIE1	tyrosine kinase with immunoglobulin like and EGF like domains 1
TLE2	transducin like enhancer of split 2
TLE3	transducin like enhancer of split 3
TLR3	toll like receptor 3
TNF	tumor necrosis factor
TNFAIP6	TNF alpha induced protein 6
TOP2A	topoisomerase (DNA) II alpha
TP53BP2	tumor protein p53 binding protein 2
TP73	tumor protein p73
TPD52L1	tumor protein D52-like 1

TRAF3	TNF receptor associated factor 3
TRAF5	TNF receptor associated factor 5
TRAF6	TNF receptor associated factor 6
TRIO	trio Rho guanine nucleotide exchange factor
TSC1	tuberous sclerosis 1
TYRO3	TYRO3 protein tyrosine kinase
VDAC1	voltage dependent anion channel 1
VEGFA	vascular endothelial growth factor A
VEGFB	vascular endothelial growth factor B
VIPR1	vasoactive intestinal peptide receptor 1
VIPR2	vasoactive intestinal peptide receptor 2
TRPV1	transient receptor potential cation channel subfamily V member 1
WNT5A	Wnt family member 5A
YWHAH	tyrosine 3-monooxygenase/tryptophan 5-monooxygenase activation protein eta
YWHAZ	tyrosine 3-monooxygenase/tryptophan 5-monooxygenase activation protein zeta
ZIC1	Zic family member 1
CXCR4	C-X-C motif chemokine receptor 4
MAPKAPK3	mitogen-activated protein kinase-activated protein kinase 3
NR4A3	nuclear receptor subfamily 4 group A member 3
GPR68	G protein-coupled receptor 68
ARID1A	AT-rich interaction domain 1A
TRRAP	transformation/transcription domain associated protein
AXIN2	axin 2
PIP5K1A	phosphatidylinositol-4-phosphate 5-kinase type 1 alpha
PIP4K2B	phosphatidylinositol-5-phosphate 4-kinase type 2 beta
ULK1	unc-51 like autophagy activating kinase 1
BCAR3	breast cancer anti-estrogen resistance 3
STK24	serine/threonine kinase 24
NSMAF	neutral sphingomyelinase activation associated factor
RGS5	regulator of G-protein signaling 5
MAP4K3	mitogen-activated protein kinase kinase kinase kinase 3
PPFIA1	PTPRF interacting protein alpha 1
PIK3R3	phosphoinositide-3-kinase regulatory subunit 3
CNTNAP1	contactin associated protein 1
LGR5	leucine-rich repeat containing G protein-coupled receptor 5
MADD	MAP kinase activating death domain
MKNK1	MAP kinase interacting serine/threonine kinase 1
RGS20	regulator of G-protein signaling 20
STC2	stanniocalcin 2
SKAP1	src kinase associated phosphoprotein 1
IRS2	insulin receptor substrate 2
TRADD	TNFRSF1A associated via death domain
TNFRSF25	tumor necrosis factor receptor superfamily member 25
RIPK1	receptor interacting serine/threonine kinase 1
TNFSF14	tumor necrosis factor superfamily member 14
TNFSF9	tumor necrosis factor superfamily member 9
RIPK2	receptor interacting serine/threonine kinase 2
FADD	Fas associated via death domain
TNFRSF18	tumor necrosis factor receptor superfamily member 18
RGS9	regulator of G-protein signaling 9
TNFRSF11A	tumor necrosis factor receptor superfamily member 11a
TNFRSF10D	tumor necrosis factor receptor superfamily member 10d
TNFRSF10C	tumor necrosis factor receptor superfamily member 10c
TNFRSF10B	tumor necrosis factor receptor superfamily member 10b
IL18RAP	interleukin 18 receptor accessory protein
GALR2	galanin receptor 2
FGF18	fibroblast growth factor 18
INPP4B	inositol polyphosphate-4-phosphatase type II B

NRP1	neuropilin 1
TMEM11	transmembrane protein 11
WISP1	WNT1 inducible signaling pathway protein 1
NR1I2	nuclear receptor subfamily 1 group I member 2
ARHGEF7	Rho guanine nucleotide exchange factor 7
SPHK1	sphingosine kinase 1
SQSTM1	sequestosome 1
BUD31	BUD31 homolog
CACNA1I	calcium voltage-gated channel subunit alpha1 I
FOXH1	forkhead box H1
SKAP2	src kinase associated phosphoprotein 2
BTRC	beta-transducin repeat containing E3 ubiquitin protein ligase
RPS6KA4	ribosomal protein S6 kinase A4
TNFSF18	tumor necrosis factor superfamily member 18
KALRN	kalirin, RhoGEF kinase
CDKL2	cyclin dependent kinase like 2
F2RL3	F2R like thrombin/trypsin receptor 3
SOCS3	suppressor of cytokine signaling 3
ARTN	artemin
PSTPIP1	proline-serine-threonine phosphatase interacting protein 1
NMI	N-myc and STAT interactor
MTA1	metastasis associated 1
SMC3	structural maintenance of chromosomes 3
HGS	hepatocyte growth factor-regulated tyrosine kinase substrate
LPAR2	lysophosphatidic acid receptor 2
MAP3K13	mitogen-activated protein kinase kinase kinase 13
VAPB	VAMP (vesicle-associated membrane protein)-associated protein B and C
VAPA	VAMP associated protein A
MAGI1	membrane associated guanylate kinase, WW and PDZ domain containing 1
CD83	CD83 molecule
TRIP10	thyroid hormone receptor interactor 10
GLP2R	glucagon like peptide 2 receptor
CD101	CD101 molecule
ECEL1	endothelin converting enzyme-like 1
NCR2	natural cytotoxicity triggering receptor 2
MED17	mediator complex subunit 17
MAP4K4	mitogen-activated protein kinase kinase kinase kinase 4
HOMER3	homer scaffolding protein 3
HOMER2	homer scaffolding protein 2
RASAL2	RAS protein activator like 2
PICK1	protein interacting with PRKCA 1
IL27RA	interleukin 27 receptor subunit alpha
MAPK8IP1	mitogen-activated protein kinase 8 interacting protein 1
ADAMTS1	ADAM metallopeptidase with thrombospondin type 1 motif 1
BCAR1	BCAR1, Cas family scaffolding protein
GABBR2	gamma-aminobutyric acid type B receptor subunit 2
CDC42BPB	CDC42 binding protein kinase beta
AKAP12	A-kinase anchoring protein 12
SMAD5-AS1	SMAD5 antisense RNA 1
RNF14	ring finger protein 14
RIN1	Ras and Rab interactor 1
CEP57	centrosomal protein 57
MAML1	mastermind like transcriptional coactivator 1
GIT2	GIT ArfGAP 2
ELMO1	engulfment and cell motility 1
MED24	mediator complex subunit 24
TLK1	tousled like kinase 1
MFN2	mitofusin 2

OXSR1	oxidative stress responsive 1
MED13	mediator complex subunit 13
NR1H4	nuclear receptor subfamily 1 group H member 4
CASP8AP2	caspase 8 associated protein 2
AKT3	AKT serine/threonine kinase 3
ABI1	abl interactor 1
TANK	TRAF family member associated NFKB activator
BCL2L10	BCL2 like 10
MED16	mediator complex subunit 16
SH2D3C	SH2 domain containing 3C
DNM1L	dynamin 1-like
PTPRU	protein tyrosine phosphatase, receptor type U
SGK2	SGK2, serine/threonine kinase 2
NAMPT	nicotinamide phosphoribosyltransferase
WASF2	WAS protein family member 2
TNK2	tyrosine kinase non receptor 2
CNKSR1	connector enhancer of kinase suppressor of Ras 1
RAMP1	receptor activity modifying protein 1
AKAP8	A-kinase anchoring protein 8
NET1	neuroepithelial cell transforming 1
APC2	adenomatosis polyposis coli 2
PAK4	p21 (RAC1) activated kinase 4
NMUR1	neuromedin U receptor 1
CITED2	Cbp/p300 interacting transactivator with Glu/Asp rich carboxy-terminal domain 2
NOD1	nucleotide binding oligomerization domain containing 1
DLC1	DLC1 Rho GTPase activating protein
TAB1	TGF-beta activated kinase 1/MAP3K7 binding protein 1
BAIAP2	BAI1 associated protein 2
MERTK	MER proto-oncogene, tyrosine kinase
TRIM38	tripartite motif containing 38
CAP1	CAP, adenylate cyclase-associated protein 1 (yeast)
STK25	serine/threonine kinase 25
IPO8	importin 8
SORBS1	sorbin and SH3 domain containing 1
CDC42EP3	CDC42 effector protein 3
STAMBP	STAM binding protein
RASL10A	RAS like family 10 member A
RGS14	regulator of G-protein signaling 14
CAMKK2	calcium/calmodulin-dependent protein kinase kinase 2
KHDRBS1	KH domain containing, RNA binding, signal transduction associated 1
RRAGA	Ras related GTP binding A
TNFSF13B	tumor necrosis factor superfamily member 13b
PTGES3	prostaglandin E synthase 3
GRAP	GRB2-related adaptor protein
CHL1	cell adhesion molecule L1 like
IQGAP2	IQ motif containing GTPase activating protein 2
FRS2	fibroblast growth factor receptor substrate 2
PPP1R17	protein phosphatase 1 regulatory subunit 17
CD3EAP	CD3e molecule associated protein
NMU	neuromedin U
RUNDC3A	RUN domain containing 3A
BRD8	bromodomain containing 8
GADD45G	growth arrest and DNA damage inducible gamma
RALBP1	ralA binding protein 1
MAPRE2	microtubule associated protein RP/EB family member 2
TLK2	tousled like kinase 2
TMED1	transmembrane p24 trafficking protein 1
ADAP1	ArfGAP with dual PH domains 1

RIPK3	receptor interacting serine/threonine kinase 3
RAPGEF4	Rap guanine nucleotide exchange factor 4
PRDM4	PR domain 4
PTPRT	protein tyrosine phosphatase, receptor type T
FAF1	Fas associated factor 1
RASSF1	Ras association domain family member 1
CHEK2	checkpoint kinase 2
AKAP10	A-kinase anchoring protein 10
GPR176	G protein-coupled receptor 176
GPR45	G protein-coupled receptor 45
PARK7	Parkinsonism associated deglycase
ICK	intestinal cell (MAK-like) kinase
ATF6	activating transcription factor 6
SCAP	SREBF chaperone
MAST1	microtubule associated serine/threonine kinase 1
ACIN1	apoptotic chromatin condensation inducer 1
PLCH1	phospholipase C eta 1
STK38L	serine/threonine kinase 38 like
TNIK	TRAF2 and NCK interacting kinase
ERC1	ELKS/RAB6-interacting/CAST family member 1
PEG10	paternally expressed 10
TAB2	TGF-beta activated kinase 1/MAP3K7 binding protein 2
MAPK8IP3	mitogen-activated protein kinase 8 interacting protein 3
ZDHHC17	zinc finger DHHC-type containing 17
ITGB3BP	integrin subunit beta 3 binding protein
TNFRSF13B	tumor necrosis factor receptor superfamily member 13B
RBFOX2	RNA binding protein, fox-1 homolog 2
DDAH2	dimethylarginine dimethylaminohydrolase 2
LPAR3	lysophosphatidic acid receptor 3
DDAH1	dimethylarginine dimethylaminohydrolase 1
OPN3	opsin 3
CLEC5A	C-type lectin domain family 5 member A
CORO1C	coronin 1C
DAPK2	death-associated protein kinase 2
CBLC	Cbl proto-oncogene C
ARFIP2	ADP ribosylation factor interacting protein 2
APPL1	adaptor protein, phosphotyrosine interacting with PH domain and leucine zipper 1
FGF20	fibroblast growth factor 20
SERGEF	secretion regulating guanine nucleotide exchange factor
LATS2	large tumor suppressor kinase 2
CIDEB	cell death-inducing DFFA-like effector b
SGSM3	small G protein signaling modulator 3
CECR2	CECR2, histone acetyl-lysine reader
GIT1	GIT ArfGAP 1
HIPK2	homeodomain interacting protein kinase 2
MED4	mediator complex subunit 4
CNOT7	CCR4-NOT transcription complex subunit 7
TRHDE	thyrotropin releasing hormone degrading enzyme
MDFIC	MyoD family inhibitor domain containing
RHOD	ras homolog family member D
HUNK	hormonally up-regulated Neu-associated kinase
KCNIP3	potassium voltage-gated channel interacting protein 3
KCNIP2	potassium voltage-gated channel interacting protein 2
KCNIP1	potassium voltage-gated channel interacting protein 1
DUOX2	dual oxidase 2
IL20	interleukin 20
TAS2R1	taste 2 receptor member 1
PDE11A	phosphodiesterase 11A

SOST	sclerostin
NDUFA13	NADH:ubiquinone oxidoreductase subunit A13
UTP11	UTP11, small subunit processome component homolog (S. cerevisiae)
PLCE1	phospholipase C epsilon 1
GMIP	GEM interacting protein
TAOK3	TAO kinase 3
UBR5	ubiquitin protein ligase E3 component n-recognin 5
TRIAP1	TP53 regulated inhibitor of apoptosis 1
CXXC5	CXXC finger protein 5
MBIP	MAP3K12 binding inhibitory protein 1
SUFU	SUFU negative regulator of hedgehog signaling
NLK	nemo like kinase
ZAK	sterile alpha motif and leucine zipper containing kinase AZK
TREM1	triggering receptor expressed on myeloid cells 1
DLL4	delta like canonical Notch ligand 4
IFT57	intraflagellar transport 57
ARHGEF10L	Rho guanine nucleotide exchange factor 10 like
RIC8B	RIC8 guanine nucleotide exchange factor B
APPL2	adaptor protein, phosphotyrosine interacting with PH domain and leucine zipper 2
MAML3	mastermind like transcriptional coactivator 3
CDC42BPG	CDC42 binding protein kinase gamma
CDKN2AIP	CDKN2A interacting protein
PSENEN	presenilin enhancer gamma-secretase subunit
GPRC5C	G protein-coupled receptor class C group 5 member C
ERBIN	erbb2 interacting protein
GNG12	G protein subunit gamma 12
PARD3	par-3 family cell polarity regulator
LTB4R2	leukotriene B4 receptor 2
CDC42SE1	CDC42 small effector 1
DUSP22	dual specificity phosphatase 22
OTUD7B	OTU deubiquitinase 7B
CDC42SE2	CDC42 small effector 2
CTNNBIP1	catenin beta interacting protein 1
SLC44A2	solute carrier family 44 member 2
SMURF1	SMAD specific E3 ubiquitin protein ligase 1
PLEKHG5	pleckstrin homology and RhoGEF domain containing G5
ALS2	ALS2, alsin Rho guanine nucleotide exchange factor
MIER1	MIER1 transcriptional regulator
MARK4	microtubule affinity regulating kinase 4
SIGIRR	single immunoglobulin and toll-interleukin 1 receptor (TIR) domain
RINT1	RAD50 interactor 1
MOAP1	modulator of apoptosis 1
RRAGC	Ras related GTP binding C
NOD2	nucleotide binding oligomerization domain containing 2
ARAP3	ArfGAP with RhoGAP domain, ankyrin repeat and PH domain 3
PINK1	PTEN induced putative kinase 1
DDX54	DEAD-box helicase 54
MUL1	mitochondrial E3 ubiquitin protein ligase 1
ADIPOR2	adiponectin receptor 2
WLS	wntless Wnt ligand secretion mediator
PREX2	phosphatidylinositol-3,4,5-trisphosphate dependent Rac exchange factor 2
APOL3	apolipoprotein L3
VOPP1	vesicular, overexpressed in cancer, prosurvival protein 1
PPP1R1B	protein phosphatase 1 regulatory inhibitor subunit 1B
SLA2	Src-like-adaptor 2
TMEM101	transmembrane protein 101
MAML2	mastermind like transcriptional coactivator 2
KIAA1804	mixed lineage kinase 4

AFAP1L2	actin filament associated protein 1 like 2
DERL3	derlin 3
CLNK	cytokine dependent hematopoietic cell linker
ARAP1	ArfGAP with RhoGAP domain, ankyrin repeat and PH domain 1
AGAP2	ArfGAP with GTPase domain, ankyrin repeat and PH domain 2
PIGU	phosphatidylinositol glycan anchor biosynthesis class U
IL31RA	interleukin 31 receptor A
IRAK1BP1	interleukin 1 receptor associated kinase 1 binding protein 1
MIB2	mindbomb E3 ubiquitin protein ligase 2
COMMD7	COMM domain containing 7
SIK1	salt inducible kinase 1
AIFM3	apoptosis inducing factor, mitochondria associated 3
NFAM1	NFAT activating protein with ITAM motif 1
ROPN1B	rhophilin associated tail protein 1B
FGD5	FYVE, RhoGEF and PH domain containing 5
EID2	EP300 interacting inhibitor of differentiation 2
NLRC3	NLR family, CARD domain containing 3
SPRED2	sprouty related EVH1 domain containing 2
ARHGAP27	Rho GTPase activating protein 27
FGD2	FYVE, RhoGEF and PH domain containing 2
RICTOR	RPTOR independent companion of MTOR complex 2
CRIPAK	cysteine rich PAK1 inhibitor
RGMB	repulsive guidance molecule family member b
AGRN	agrin
**Transcription DNA dependent**	
**Gene Symbol**	**Name Gene**
ABCA2	ATP binding cassette subfamily A member 2
PARP1	poly(ADP-ribose) polymerase 1
AHR	aryl hydrocarbon receptor
ANG	angiogenin
ARNTL	aryl hydrocarbon receptor nuclear translocator like
ZFHX3	zinc finger homeobox 3
NKX3-2	NK3 homeobox 2
BCL6	B-cell CLL/lymphoma 6
PRDM1	PR domain 1
BMP2	bone morphogenetic protein 2
BMP6	bone morphogenetic protein 6
BRCA1	breast cancer 1
BTF3	basic transcription factor 3
CALR	calreticulin
RUNX1	runt related transcription factor 1
RUNX3	runt related transcription factor 3
CBFB	core-binding factor, beta subunit
CDK7	cyclin-dependent kinase 7
CEBPA	CCAAT/enhancer binding protein alpha
CEBPB	CCAAT/enhancer binding protein beta
CHD3	chromodomain helicase DNA binding protein 3
FOXN3	forkhead box N3
CREBBP	CREB binding protein
CREM	cAMP responsive element modulator
NKX2-5	NK2 homeobox 5
CUX1	cut like homeobox 1
DAXX	death-domain associated protein
DBP	D-box binding PAR bZIP transcription factor
DNMT1	DNA (cytosine-5-)-methyltransferase 1
DTX1	deltex 1
E2F2	E2F transcription factor 2

E2F3	E2F transcription factor 3
ELAVL2	ELAV like neuron-specific RNA binding protein 2
ELF2	E74 like ETS transcription factor 2
EPAS1	endothelial PAS domain protein 1
ERCC3	excision repair cross-complementation group 3
ERCC6	excision repair cross-complementation group 6
ERF	ETS2 repressor factor
ESRRG	estrogen related receptor gamma
EZH2	enhancer of zeste 2 polycomb repressive complex 2 subunit
BPTF	bromodomain PHD finger transcription factor
FOXF1	forkhead box F1
FOXF2	forkhead box F2
FOXE3	forkhead box E3
FOXO1	forkhead box O1
FOSL2	FOS like 2, AP-1 trancription factor subunit
NR5A2	nuclear receptor subfamily 5 group A member 2
NR5A1	nuclear receptor subfamily 5 group A member 1
XRCC6	X-ray repair complementing defective repair in Chinese hamster cells 6
GATA2	GATA binding protein 2
GATA3	GATA binding protein 3
GATA4	GATA binding protein 4
GATA6	GATA binding protein 6
KAT2A	lysine acetyltransferase 2A
NR6A1	nuclear receptor subfamily 6 group A member 1
GFI1	growth factor independent 1 transcriptional repressor
GLI2	GLI family zinc finger 2
NR3C1	nuclear receptor subfamily 3 group C member 1
GSC2	goosecoid homeobox 2
GTF2B	general transcription factor IIB
GTF2H1	general transcription factor IIH subunit 1
GTF2I	general transcription factor IIi
BRF1	BRF1, RNA polymerase III transcription initiation factor 90 kDa subunit
HDAC2	histone deacetylase 2
HIC1	hypermethylated in cancer 1
MNX1	motor neuron and pancreas homeobox 1
HMGB1	high mobility group box 1
HNF4A	hepatocyte nuclear factor 4 alpha
HNF4G	hepatocyte nuclear factor 4 gamma
HNRNPAB	heterogeneous nuclear ribonucleoprotein A/B
HOXC6	homeobox C6
IRF8	interferon regulatory factor 8
ID4	inhibitor of DNA binding 4, HLH protein
FOXK2	forkhead box K2
ILF2	interleukin enhancer binding factor 2
ILF3	interleukin enhancer binding factor 3
ING2	inhibitor of growth family member 2
INHBA	inhibin beta A
IRF1	interferon regulatory factor 1
IRF2	interferon regulatory factor 2
IRF7	interferon regulatory factor 7
LMX1B	LIM homeobox transcription factor 1 beta
SMAD2	SMAD family member 2
SMAD3	SMAD family member 3
MAX	MYC associated factor X
MEIS2	Meis homeobox 2
MEN1	menin 1
MLLT6	myeloid/lymphoid or mixed-lineage leukemia; translocated to, 6
MYC	v-myc avian myelocytomatosis viral oncogene homolog

MYCN	v-myc avian myelocytomatosis viral oncogene neuroblastoma derived homolog
MYO6	myosin VI
MYOD1	myogenic differentiation 1
NEUROD2	neuronal differentiation 2
NEUROG1	neurogenin 1
NFATC1	nuclear factor of activated T-cells 1
NFATC2	nuclear factor of activated T-cells 2
NFATC4	nuclear factor of activated T-cells 4
NFIC	nuclear factor I C
NFIX	nuclear factor I X
NFKB1	nuclear factor kappa B subunit 1
NFRKB	nuclear factor related to kappaB binding protein
NFYA	nuclear transcription factor Y subunit alpha
NFYC	nuclear transcription factor Y subunit gamma
NOTCH4	notch 4
NPAS2	neuronal PAS domain protein 2
NRF1	nuclear respiratory factor 1
NRL	neural retina leucine zipper
PAWR	pro-apoptotic WT1 regulator
PAX1	paired box 1
PAX2	paired box 2
PBX1	PBX homeobox 1
PBX3	PBX homeobox 3
PEX14	peroxisomal biogenesis factor 14
PFDN5	prefoldin subunit 5
PITX2	paired like homeodomain 2
PLAGL1	PLAG1 like zinc finger 1
POLR2D	polymerase (RNA) II subunit D
POLR2F	polymerase (RNA) II subunit F
POLR2G	polymerase (RNA) II subunit G
POLR2J	polymerase (RNA) II subunit J
POLRMT	polymerase (RNA) mitochondrial
POU2AF1	POU class 2 associating factor 1
POU5F1	POU class 5 homeobox 1
PPARA	peroxisome proliferator activated receptor alpha
PPARD	peroxisome proliferator activated receptor delta
PPARG	peroxisome proliferator activated receptor gamma
PSMC5	proteasome 26S subunit, ATPase 5
PTGER3	prostaglandin E receptor 3
RB1	retinoblastoma 1
RBBP8	retinoblastoma binding protein 8
REST	RE1 silencing transcription factor
TRIM27	tripartite motif containing 27
RNF4	ring finger protein 4
RORB	RAR related orphan receptor B
RREB1	ras responsive element binding protein 1
RXRA	retinoid X receptor alpha
SNAI2	snail family transcriptional repressor 2
SMARCA2	SWI/SNF related, matrix associated, actin dependent regulator of chromatin, subfamily a, member 2
SMARCD2	SWI/SNF related, matrix associated, actin dependent regulator of chromatin, subfamily d, member 2
SMARCE1	SWI/SNF related, matrix associated, actin dependent regulator of chromatin, subfamily e, member 1
SNAPC2	small nuclear RNA activating complex polypeptide 2
SNAPC3	small nuclear RNA activating complex polypeptide 3
SOD2	superoxide dismutase 2, mitochondrial
SP1	Sp1 transcription factor

SP2	Sp2 transcription factor
SP3	Sp3 transcription factor
SP100	SP100 nuclear antigen
SPI1	Spi-1 proto-oncogene
SPIB	Spi-B transcription factor
SREBF1	sterol regulatory element binding transcription factor 1
SREBF2	sterol regulatory element binding transcription factor 2
SUPT5H	SPT5 homolog, DSIF elongation factor subunit
TAF5	TATA-box binding protein associated factor 5
TARBP1	TAR (HIV-1) RNA binding protein 1
TARBP2	TARBP2, RISC loading complex RNA binding subunit
TBX5	T-box 5
TCEB1	transcription elongation factor B subunit 1
TCEB2	transcription elongation factor B subunit 2
TBX3	T-box 3
HNF1A	HNF1 homeobox A
HNF1B	HNF1 homeobox B
TCF3	transcription factor 3
TCF7L2	transcription factor 7 like 2
ZEB1	zinc finger E-box binding homeobox 1
TCF12	transcription factor 12
ZNF354A	zinc finger protein 354A
TCF19	transcription factor 19
VPS72	vacuolar protein sorting 72 homolog
TFAP2A	transcription factor AP-2 alpha
TFAP2B	transcription factor AP-2 beta
NR2F2	nuclear receptor subfamily 2 group F member 2
TFDP1	transcription factor Dp-1
TGFB1	transforming growth factor beta 1
TGIF1	TGFB induced factor homeobox 1
KLF10	Kruppel-like factor 10
TIAL1	TIA1 cytotoxic granule-associated RNA binding protein-like 1
TNF	tumor necrosis factor
TP73	tumor protein p73
TRPS1	transcriptional repressor GATA binding 1
HIRA	histone cell cycle regulator
TWIST1	twist family bHLH transcription factor 1
UBTF	upstream binding transcription factor, RNA polymerase I
USF1	upstream transcription factor 1
YY1	YY1 transcription factor
ZNF45	zinc finger protein 45
ZNF74	zinc finger protein 74
ZBTB16	zinc finger and BTB domain containing 16
ZNF146	zinc finger protein 146
ZNF148	zinc finger protein 148
ZNF174	zinc finger protein 174
PAX8	paired box 8
ALX1	ALX homeobox 1
ELL	elongation factor for RNA polymerase II
ARID1A	AT-rich interaction domain 1A
KLF11	Kruppel-like factor 11
SUPT3H	SPT3 homolog, SAGA and STAGA complex component
IKBKAP	inhibitor of kappa light polypeptide gene enhancer in B-cells, kinase complex-associated protein
RUVBL1	RuvB like AAA ATPase 1
CREG1	cellular repressor of E1A stimulated genes 1
TRIM24	tripartite motif containing 24
TSC22D1	TSC22 domain family member 1

LDB1	LIM domain binding 1
SQSTM1	sequestosome 1
FUBP1	far upstream element binding protein 1
BUD31	BUD31 homolog
RPS6KA4	ribosomal protein S6 kinase A4
ASH2L	ASH2 like histone lysine methyltransferase complex subunit
TBX19	T-box 19
NMI	N-myc and STAT interactor
DYRK1B	dual specificity tyrosine phosphorylation regulated kinase 1B
LRRFIP1	leucine rich repeat (in FLII) interacting protein 1
PTTG1	pituitary tumor-transforming 1
MSC	musculin
KLF4	Kruppel-like factor 4 (gut)
TRIP13	thyroid hormone receptor interactor 13
TRIP11	thyroid hormone receptor interactor 11
ZNHIT3	zinc finger HIT-type containing 3
MED21	mediator complex subunit 21
MED17	mediator complex subunit 17
MED26	mediator complex subunit 26
HAND2	heart and neural crest derivatives expressed 2
VPS4B	vacuolar protein sorting 4 homolog B
POLR1C	polymerase (RNA) I subunit C
CIR1	corepressor interacting with RBPJ, 1
CREB5	cAMP responsive element binding protein 5
NFE2L3	nuclear factor, erythroid 2 like 3
RNF14	ring finger protein 14
HDAC4	histone deacetylase 4
MAML1	mastermind like transcriptional coactivator 1
MED24	mediator complex subunit 24
MED13	mediator complex subunit 13
HDAC5	histone deacetylase 5
TRIM28	tripartite motif containing 28
SAP18	Sin3A associated protein 18kDa
CITED2	Cbp/p300 interacting transactivator with Glu/Asp rich carboxy-terminal domain 2
FST	follistatin
TADA3	transcriptional adaptor 3
MYBBP1A	MYB binding protein 1a
DEAF1	DEAF1, transcription factor
KAT5	lysine acetyltransferase 5
IVNS1ABP	influenza virus NS1A binding protein
TCFL5	transcription factor like 5
ZMYND11	zinc finger MYND-type containing 11
ZNF274	zinc finger protein 274
CD3EAP	CD3e molecule associated protein
ARID5A	AT-rich interaction domain 5A
PPARGC1A	PPARG coactivator 1 alpha
BRD8	bromodomain containing 8
ADRM1	adhesion regulating molecule 1
SOX30	SRY-box 30
PRDM4	PR domain 4
KAT7	lysine acetyltransferase 7
SOX21	SRY-box 21
SNF8	SNF8, ESCRT-II complex subunit
KLF12	Kruppel-like factor 12
POU6F2	POU class 6 homeobox 2
ATF5	activating transcription factor 5
ZHX2	zinc fingers and homeoboxes 2
TRAK1	trafficking kinesin protein 1

ATF6	activating transcription factor 6
SCAP	SREBF chaperone
CRTC1	CREB regulated transcription coactivator 1
SIRT5	sirtuin 5
RYBP	RING1 and YY1 binding protein
TRIM29	tripartite motif containing 29
POU2F3	POU class 2 homeobox 3
SIN3A	SIN3 transcription regulator family member A
GMEB2	glucocorticoid modulatory element binding protein 2
EHF	ETS homologous factor
NUFIP1	NUFIP1, FMR1 interacting protein 1
FOXD3	forkhead box D3
MED4	mediator complex subunit 4
UHRF1	ubiquitin like with PHD and ring finger domains 1
ABT1	activator of basal transcription 1
UBN1	ubinuclein 1
MDFIC	MyoD family inhibitor domain containing
KCNIP3	potassium voltage-gated channel interacting protein 3
SMARCAL1	SWI/SNF related, matrix associated, actin dependent regulator of chromatin, subfamily a like 1
MYEF2	myelin expression factor 2
NDUFA13	NADH:ubiquinone oxidoreductase subunit A13
YBX2	Y-box binding protein 2
PHF21A	PHD finger protein 21A
KLF13	Kruppel-like factor 13
SUFU	SUFU negative regulator of hedgehog signaling
POLR3K	polymerase (RNA) III subunit K
SOX18	SRY-box 18
FEV	FEV, ETS transcription factor
ELP3	elongator acetyltransferase complex subunit 3
MAML3	mastermind like transcriptional coactivator 3
TRERF1	transcriptional regulating factor 1
ASH1L	ASH1 like histone lysine methyltransferase
SLC2A4RG	SLC2A4 regulator
CTNNBIP1	catenin beta interacting protein 1
NSD1	nuclear receptor binding SET domain protein 1
NAA15	N(alpha)-acetyltransferase 15, NatA auxiliary subunit
ELL3	elongation factor for RNA polymerase II 3
PBX4	PBX homeobox 4
SCRT1	scratch family transcriptional repressor 1
PCBD2	pterin-4 alpha-carbinolamine dehydratase 2
SLA2	Src-like-adaptor 2
MAML2	mastermind like transcriptional coactivator 2
AFAP1L2	actin filament associated protein 1 like 2
GTF3C6	general transcription factor IIIC subunit 6
TWIST2	twist family bHLH transcription factor 2
HEXIM2	hexamethylene bisacetamide inducible 2
GABPB2	GA binding protein transcription factor beta subunit 2
TAF8	TATA-box binding protein associated factor 8
GLIS3	GLIS family zinc finger 3
ZNF367	zinc finger protein 367
JMJD1C	jumonji domain containing 1C
JAZF1	JAZF zinc finger 1
ZBTB38	zinc finger and BTB domain containing 38
PTF1A	pancreas specific transcription factor, 1a
MAFA	MAF bZIP transcription factor A
**Regulation of developmental process**	
**Gene**	**Name Gene**

Symbol	
ACVR1	activin A receptor type 1
ADORA1	adenosine A1 receptor
ALB	albumin
ALOX15B	arachidonate 15-lipoxygenase, type B
ANXA1	annexin A1
ANXA4	annexin A4
ANXA5	annexin A5
APAF1	apoptotic peptidase activating factor 1
BIRC3	baculoviral IAP repeat containing 3
BIRC5	baculoviral IAP repeat containing 5
FASLG	Fas ligand
RHOB	ras homolog family member B
ARHGDIA	Rho GDP dissociation inhibitor alpha
ASNS	asparagine synthetase (glutamine-hydrolyzing)
BAK1	BCL2 antagonist/killer 1
BCL2	B-cell CLL/lymphoma 2
BCL2A1	BCL2 related protein A1
BCL2L2	BCL2 like 2
BCL3	B-cell CLL/lymphoma 3
BCL6	B-cell CLL/lymphoma 6
BID	BH3 interacting domain death agonist
BIK	BCL2 interacting killer
BMP4	bone morphogenetic protein 4
BNIP2	BCL2/adenovirus E1B 19kDa interacting protein 2
BNIP3	BCL2/adenovirus E1B 19kDa interacting protein 3
BNIP3L	BCL2/adenovirus E1B 19kDa interacting protein 3-like
FOXL2	forkhead box L2
BRCA1	breast cancer 1
BRAF	B-Raf proto-oncogene, serine/threonine kinase
BTG1	B-cell translocation gene 1, anti-proliferative
CALCA	calcitonin related polypeptide alpha
CALR	calreticulin
CASP9	caspase 9
CASP10	caspase 10
RUNX1	runt related transcription factor 1
RUNX3	runt related transcription factor 3
TNFRSF8	tumor necrosis factor receptor superfamily member 8
CD74	CD74 molecule
CDC42	cell division cycle 42
CDK6	cyclin-dependent kinase 6
CDKN1A	cyclin-dependent kinase inhibitor 1A
CDKN1B	cyclin-dependent kinase inhibitor 1B
CENPF	centromere protein F
CFL1	cofilin 1
CIDEA	cell death-inducing DFFA-like effector a
COL4A2	collagen type IV alpha 2
CRYAA	crystallin alpha A
DAD1	defender against cell death 1
DAP	death-associated protein
DAPK1	death associated protein kinase 1
DAPK3	death-associated protein kinase 3
DAXX	death-domain associated protein
DFFA	DNA fragmentation factor subunit alpha
DHCR24	24-dehydrocholesterol reductase
DTX1	deltex 1
EIF5A	eukaryotic translation initiation factor 5A
ERCC3	excision repair cross-complementation group 3

ETS1	ETS proto-oncogene 1, transcription factor
FOXO1	forkhead box O1
IFI6	interferon alpha inducible protein 6
GDNF	glial cell derived neurotrophic factor
GCLC	glutamate-cysteine ligase catalytic subunit
SFN	stratifin
GRM4	glutamate metabotropic receptor 4
GSTP1	glutathione S-transferase pi 1
HTT	huntingtin
HDAC1	histone deacetylase 1
HIP1	huntingtin interacting protein 1
HMGB1	high mobility group box 1
HSPA9	heat shock protein family A (Hsp70) member 9
HSPD1	heat shock protein family D (Hsp60) member 1
IFNA2	interferon, alpha 2
IGF1R	insulin like growth factor 1 receptor
IGFBP3	insulin like growth factor binding protein 3
IL12A	interleukin 12A
IL12B	interleukin 12B
INHBA	inhibin beta A
KRT18	keratin 18
LCK	LCK proto-oncogene, Src family tyrosine kinase
LTA	lymphotoxin alpha
SMAD3	SMAD family member 3
MAPT	microtubule associated protein tau
MAP3K5	mitogen-activated protein kinase kinase kinase 5
MAP3K10	mitogen-activated protein kinase kinase kinase 10
MPO	myeloperoxidase
MYH9	myosin, heavy chain 9, non-muscle
NF1	neurofibromin 1
NFKB1	nuclear factor kappa B subunit 1
NOTCH1	notch 1
NOTCH4	notch 4
NPR1	natriuretic peptide receptor 1
OPA1	OPA1, mitochondrial dynamin like GTPase
PAX7	paired box 7
PIM1	Pim-1 proto-oncogene, serine/threonine kinase
PIK3R2	phosphoinositide-3-kinase regulatory subunit 2
PLAGL1	PLAG1 like zinc finger 1
PLG	plasminogen
PMAIP1	phorbol-12-myristate-13-acetate-induced protein 1
PPARG	peroxisome proliferator activated receptor gamma
PPP2CA	protein phosphatase 2 catalytic subunit alpha
SRGN	serglycin
PRKCA	protein kinase C alpha
PRKCE	protein kinase C epsilon
PRKCZ	protein kinase C zeta
MAPK1	mitogen-activated protein kinase 1
PROC	protein C, inactivator of coagulation factors Va and VIIIa
PSEN1	presenilin 1
PTEN	phosphatase and tensin homolog
PTH	parathyroid hormone
PTPRC	protein tyrosine phosphatase, receptor type C
RELA	RELA proto-oncogene, NF-kB subunit
DPF2	double PHD fingers 2
ROBO1	roundabout guidance receptor 1
ROBO2	roundabout guidance receptor 2
RTKN	rhotekin

SFRP1	secreted frizzled related protein 1
SHH	sonic hedgehog
SON	SON DNA binding protein
SPI1	Spi-1 proto-oncogene
STK4	serine/threonine kinase 4
TBX5	T-box 5
TBX3	T-box 3
PRDX2	peroxiredoxin 2
TGFB1	transforming growth factor beta 1
TGFB2	transforming growth factor beta 2
TIA1	TIA1 cytotoxic granule-associated RNA binding protein
TIAL1	TIA1 cytotoxic granule-associated RNA binding protein-like 1
TNF	tumor necrosis factor
TNNI3	troponin I3, cardiac type
TOP2A	topoisomerase (DNA) II alpha
TP53BP2	tumor protein p53 binding protein 2
TP73	tumor protein p73
TPD52L1	tumor protein D52-like 1
TRAF3	TNF receptor associated factor 3
UBB	ubiquitin B
VEGFA	vascular endothelial growth factor A
YWHAG	tyrosine 3-monooxygenase/tryptophan 5-monooxygenase activation protein gamma
YWHAH	tyrosine 3-monooxygenase/tryptophan 5-monooxygenase activation protein eta
YWHAZ	tyrosine 3-monooxygenase/tryptophan 5-monooxygenase activation protein zeta
ZAP70	zeta chain of T cell receptor associated protein kinase 70kDa
ZBTB16	zinc finger and BTB domain containing 16
CUL5	cullin 5
DPF1	double PHD fingers 1
CUL4A	cullin 4A
CUL2	cullin 2
MADD	MAP kinase activating death domain
BECN1	beclin 1
TRADD	TNFRSF1A associated via death domain
TNFRSF25	tumor necrosis factor receptor superfamily member 25
HRK	harakiri, BCL2 interacting protein
TNFSF14	tumor necrosis factor superfamily member 14
FADD	Fas associated via death domain
TNFRSF18	tumor necrosis factor receptor superfamily member 18
TNFRSF10D	tumor necrosis factor receptor superfamily member 10d
LDB1	LIM domain binding 1
IER3	immediate early response 3
SPHK1	sphingosine kinase 1
TNFSF18	tumor necrosis factor superfamily member 18
SOCS3	suppressor of cytokine signaling 3
SART1	squamous cell carcinoma antigen recognized by T-cells 1
KL	klotho
EI24	EI24, autophagy associated transmembrane protein
BCAR1	BCAR1, Cas family scaffolding protein
RNF7	ring finger protein 7
HDAC9	histone deacetylase 9
HDAC4	histone deacetylase 4
CASP8AP2	caspase 8 associated protein 2
HDAC5	histone deacetylase 5
BCL2L10	BCL2 like 10
DNAJB6	DnaJ heat shock protein family (Hsp40) member B6
HIPK3	homeodomain interacting protein kinase 3
SMNDC1	survival motor neuron domain containing 1
CDC42EP2	CDC42 effector protein 2

FST	follistatin
SEMA4D	semaphorin 4D
RRAGA	Ras related GTP binding A
TCFL5	transcription factor like 5
RIPK3	receptor interacting serine/threonine kinase 3
CHEK2	checkpoint kinase 2
NLGN1	neuroligin 1
ACIN1	apoptotic chromatin condensation inducer 1
ERC1	ELKS/RAB6-interacting/CAST family member 1
STAB1	stabilin 1
PPP1R13B	protein phosphatase 1 regulatory subunit 13B
DDAH2	dimethylarginine dimethylaminohydrolase 2
DAPK2	death-associated protein kinase 2
CADM1	cell adhesion molecule 1
TNFAIP8	TNF alpha induced protein 8
CIDEB	cell death-inducing DFFA-like effector b
SAP30BP	SAP30 binding protein
IL20	interleukin 20
NDUFA13	NADH:ubiquinone oxidoreductase subunit A13
SH3GLB1	SH3 domain containing GRB2 like endophilin B1
TRIAP1	TP53 regulated inhibitor of apoptosis 1
HDAC7	histone deacetylase 7
ZAK	sterile alpha motif and leucine zipper containing kinase AZK
IFT57	intraflagellar transport 57
AGGF1	angiogenic factor with G-patch and FHA domains 1
ANKH	ANKH inorganic pyrophosphate transport regulator
MKL2	MKL1/myocardin like 2
LRRC4C	leucine rich repeat containing 4C
CIDEC	cell death inducing DFFA like effector c
MOAP1	modulator of apoptosis 1
ARAP3	ArfGAP with RhoGAP domain, ankyrin repeat and PH domain 3
AKT1S1	AKT1 substrate 1
AIFM2	apoptosis inducing factor, mitochondria associated 2
HPS4	HPS4, biogenesis of lysosomal organelles complex 3 subunit 2
NLRP3	NLR family, pyrin domain containing 3
ARAP1	ArfGAP with RhoGAP domain, ankyrin repeat and PH domain 1
TWIST2	twist family bHLH transcription factor 2
IKBIP	IKBKB interacting protein
TAF8	TATA-box binding protein associated factor 8
ACVR1C	activin A receptor type 1C
ADIG	adipogenin
SIK1	salt inducible kinase 1
AIFM3	apoptosis inducing factor, mitochondria associated 3
FGD5	FYVE, RhoGEF and PH domain containing 5
CNTN4	contactin 4
PRUNE2	prune homolog 2 (Drosophila)
FGD2	FYVE, RhoGEF and PH domain containing 2
PCSK9	proprotein convertase subtilisin/kexin type 9
MYO18A	myosin XVIIIA
**RNA biosynthetic process**	
**Gene Symbol**	**Name Gene**
ABCA2	ATP binding cassette subfamily A member 2
PARP1	poly(ADP-ribose) polymerase 1
AHR	aryl hydrocarbon receptor
ANG	angiogenin
ARNTL	aryl hydrocarbon receptor nuclear translocator like
ZFHX3	zinc finger homeobox 3

NKX3-2	NK3 homeobox 2
BCL6	B-cell CLL/lymphoma 6
PRDM1	PR domain 1
BMP2	bone morphogenetic protein 2
BMP6	bone morphogenetic protein 6
BRCA1	breast cancer 1
BTF3	basic transcription factor 3
CALR	calreticulin
RUNX1	runt related transcription factor 1
RUNX3	runt related transcription factor 3
CBFB	core-binding factor, beta subunit
CDK7	cyclin-dependent kinase 7
CEBPA	CCAAT/enhancer binding protein alpha
CEBPB	CCAAT/enhancer binding protein beta
CHD3	chromodomain helicase DNA binding protein 3
FOXN3	forkhead box N3
CREBBP	CREB binding protein
CREM	cAMP responsive element modulator
NKX2-5	NK2 homeobox 5
CUX1	cut like homeobox 1
DAXX	death-domain associated protein
DBP	D-box binding PAR bZIP transcription factor
DNMT1	DNA (cytosine-5-)-methyltransferase 1
DTX1	deltex 1
E2F2	E2F transcription factor 2
E2F3	E2F transcription factor 3
ELAVL2	ELAV like neuron-specific RNA binding protein 2
ELF2	E74 like ETS transcription factor 2
EPAS1	endothelial PAS domain protein 1
ERCC3	excision repair cross-complementation group 3
ERCC6	excision repair cross-complementation group 6
ERF	ETS2 repressor factor
ESRRG	estrogen related receptor gamma
EZH2	enhancer of zeste 2 polycomb repressive complex 2 subunit
BPTF	bromodomain PHD finger transcription factor
FOXF1	forkhead box F1
FOXF2	forkhead box F2
FOXE3	forkhead box E3
FOXO1	forkhead box O1
FOSL2	FOS like 2, AP-1 trancription factor subunit
NR5A2	nuclear receptor subfamily 5 group A member 2
NR5A1	nuclear receptor subfamily 5 group A member 1
XRCC6	X-ray repair complementing defective repair in Chinese hamster cells 6
GATA2	GATA binding protein 2
GATA3	GATA binding protein 3
GATA4	GATA binding protein 4
GATA6	GATA binding protein 6
KAT2A	lysine acetyltransferase 2A
NR6A1	nuclear receptor subfamily 6 group A member 1
GFI1	growth factor independent 1 transcriptional repressor
GLI2	GLI family zinc finger 2
NR3C1	nuclear receptor subfamily 3 group C member 1
GSC2	goosecoid homeobox 2
GTF2B	general transcription factor IIB
GTF2H1	general transcription factor IIH subunit 1
GTF2I	general transcription factor IIi
BRF1	BRF1, RNA polymerase III transcription initiation factor 90 kDa subunit
HDAC2	histone deacetylase 2

HIC1	hypermethylated in cancer 1
MNX1	motor neuron and pancreas homeobox 1
HMGB1	high mobility group box 1
HNF4A	hepatocyte nuclear factor 4 alpha
HNF4G	hepatocyte nuclear factor 4 gamma
HNRNPAB	heterogeneous nuclear ribonucleoprotein A/B
HOXC6	homeobox C6
IRF8	interferon regulatory factor 8
ID4	inhibitor of DNA binding 4, HLH protein
FOXK2	forkhead box K2
ILF2	interleukin enhancer binding factor 2
ILF3	interleukin enhancer binding factor 3
ING2	inhibitor of growth family member 2
INHBA	inhibin beta A
IRF1	interferon regulatory factor 1
IRF2	interferon regulatory factor 2
IRF7	interferon regulatory factor 7
LMX1B	LIM homeobox transcription factor 1 beta
SMAD2	SMAD family member 2
SMAD3	SMAD family member 3
MAX	MYC associated factor X
MEIS2	Meis homeobox 2
MEN1	menin 1
MLLT6	myeloid/lymphoid or mixed-lineage leukemia; translocated to, 6
MYC	v-myc avian myelocytomatosis viral oncogene homolog
MYCN	v-myc avian myelocytomatosis viral oncogene neuroblastoma derived homolog
MYO6	myosin VI
MYOD1	myogenic differentiation 1
NEUROD2	neuronal differentiation 2
NEUROG1	neurogenin 1
NFATC1	nuclear factor of activated T-cells 1
NFATC2	nuclear factor of activated T-cells 2
NFATC4	nuclear factor of activated T-cells 4
NFIC	nuclear factor I C
NFIX	nuclear factor I X
NFKB1	nuclear factor kappa B subunit 1
NFRKB	nuclear factor related to kappaB binding protein
NFYA	nuclear transcription factor Y subunit alpha
NFYC	nuclear transcription factor Y subunit gamma
NOTCH4	notch 4
NPAS2	neuronal PAS domain protein 2
NRF1	nuclear respiratory factor 1
NRL	neural retina leucine zipper
PAWR	pro-apoptotic WT1 regulator
PAX1	paired box 1
PAX2	paired box 2
PBX1	PBX homeobox 1
PBX3	PBX homeobox 3
PEX14	peroxisomal biogenesis factor 14
PFDN5	prefoldin subunit 5
PITX2	paired like homeodomain 2
PLAGL1	PLAG1 like zinc finger 1
POLR2D	polymerase (RNA) II subunit D
POLR2F	polymerase (RNA) II subunit F
POLR2G	polymerase (RNA) II subunit G
POLR2J	polymerase (RNA) II subunit J
POLRMT	polymerase (RNA) mitochondrial
POU2AF1	POU class 2 associating factor 1

POU5F1	POU class 5 homeobox 1
PPARA	peroxisome proliferator activated receptor alpha
PPARD	peroxisome proliferator activated receptor delta
PPARG	peroxisome proliferator activated receptor gamma
PRIM1	primase (DNA) subunit 1
PSMC5	proteasome 26S subunit, ATPase 5
PTGER3	prostaglandin E receptor 3
RB1	retinoblastoma 1
RBBP8	retinoblastoma binding protein 8
REST	RE1 silencing transcription factor
TRIM27	tripartite motif containing 27
RNF4	ring finger protein 4
RORB	RAR related orphan receptor B
RREB1	ras responsive element binding protein 1
RXRA	retinoid X receptor alpha
SNAI2	snail family transcriptional repressor 2
SMARCA2	SWI/SNF related, matrix associated, actin dependent regulator of chromatin, subfamily a, member 2
SMARCD2	SWI/SNF related, matrix associated, actin dependent regulator of chromatin, subfamily d, member 2
SMARCE1	SWI/SNF related, matrix associated, actin dependent regulator of chromatin, subfamily e, member 1
SNAPC2	small nuclear RNA activating complex polypeptide 2
SNAPC3	small nuclear RNA activating complex polypeptide 3
SOD2	superoxide dismutase 2, mitochondrial
SP1	Sp1 transcription factor
SP2	Sp2 transcription factor
SP3	Sp3 transcription factor
SP100	SP100 nuclear antigen
SPI1	Spi-1 proto-oncogene
SPIB	Spi-B transcription factor
SREBF1	sterol regulatory element binding transcription factor 1
SREBF2	sterol regulatory element binding transcription factor 2
SUPT5H	SPT5 homolog, DSIF elongation factor subunit
TAF5	TATA-box binding protein associated factor 5
TARBP1	TAR (HIV-1) RNA binding protein 1
TARBP2	TARBP2, RISC loading complex RNA binding subunit
TBX5	T-box 5
TCEB1	transcription elongation factor B subunit 1
TCEB2	transcription elongation factor B subunit 2
TBX3	T-box 3
HNF1A	HNF1 homeobox A
HNF1B	HNF1 homeobox B
TCF3	transcription factor 3
TCF7L2	transcription factor 7 like 2
ZEB1	zinc finger E-box binding homeobox 1
TCF12	transcription factor 12
ZNF354A	zinc finger protein 354A
TCF19	transcription factor 19
VPS72	vacuolar protein sorting 72 homolog
TFAP2A	transcription factor AP-2 alpha
TFAP2B	transcription factor AP-2 beta
NR2F2	nuclear receptor subfamily 2 group F member 2
TFDP1	transcription factor Dp-1
TGFB1	transforming growth factor beta 1
TGIF1	TGFB induced factor homeobox 1
KLF10	Kruppel-like factor 10
TIAL1	TIA1 cytotoxic granule-associated RNA binding protein-like 1

TNF	tumor necrosis factor
TP73	tumor protein p73
TRPS1	transcriptional repressor GATA binding 1
HIRA	histone cell cycle regulator
TWIST1	twist family bHLH transcription factor 1
UBTF	upstream binding transcription factor, RNA polymerase I
USF1	upstream transcription factor 1
YY1	YY1 transcription factor
ZNF45	zinc finger protein 45
ZNF74	zinc finger protein 74
ZBTB16	zinc finger and BTB domain containing 16
ZNF146	zinc finger protein 146
ZNF148	zinc finger protein 148
ZNF174	zinc finger protein 174
PAX8	paired box 8
ALX1	ALX homeobox 1
ELL	elongation factor for RNA polymerase II
ARID1A	AT-rich interaction domain 1A
KLF11	Kruppel-like factor 11
SUPT3H	SPT3 homolog, SAGA and STAGA complex component
IKBKAP	inhibitor of kappa light polypeptide gene enhancer in B-cells, kinase complex-associated protein
RUVBL1	RuvB like AAA ATPase 1
CREG1	cellular repressor of E1A stimulated genes 1
TRIM24	tripartite motif containing 24
TSC22D1	TSC22 domain family member 1
LDB1	LIM domain binding 1
SQSTM1	sequestosome 1
FUBP1	far upstream element binding protein 1
BUD31	BUD31 homolog
RPS6KA4	ribosomal protein S6 kinase A4
ASH2L	ASH2 like histone lysine methyltransferase complex subunit
TBX19	T-box 19
NMI	N-myc and STAT interactor
DYRK1B	dual specificity tyrosine phosphorylation regulated kinase 1B
LRRFIP1	leucine rich repeat (in FLII) interacting protein 1
PTTG1	pituitary tumor-transforming 1
MSC	musculin
KLF4	Kruppel-like factor 4 (gut)
TRIP13	thyroid hormone receptor interactor 13
TRIP11	thyroid hormone receptor interactor 11
ZNHIT3	zinc finger HIT-type containing 3
MED21	mediator complex subunit 21
MED17	mediator complex subunit 17
MED26	mediator complex subunit 26
HAND2	heart and neural crest derivatives expressed 2
VPS4B	vacuolar protein sorting 4 homolog B
POLR1C	polymerase (RNA) I subunit C
CIR1	corepressor interacting with RBPJ, 1
CREB5	cAMP responsive element binding protein 5
NFE2L3	nuclear factor, erythroid 2 like 3
RNF14	ring finger protein 14
HDAC4	histone deacetylase 4
MAML1	mastermind like transcriptional coactivator 1
MED24	mediator complex subunit 24
MED13	mediator complex subunit 13
HDAC5	histone deacetylase 5
TRIM28	tripartite motif containing 28

SAP18	Sin3A associated protein 18kDa
CITED2	Cbp/p300 interacting transactivator with Glu/Asp rich carboxy-terminal domain 2
FST	follistatin
TADA3	transcriptional adaptor 3
MYBBP1A	MYB binding protein 1a
DEAF1	DEAF1, transcription factor
KAT5	lysine acetyltransferase 5
IVNS1ABP	influenza virus NS1A binding protein
TCFL5	transcription factor like 5
ZMYND11	zinc finger MYND-type containing 11
ZNF274	zinc finger protein 274
CD3EAP	CD3e molecule associated protein
ARID5A	AT-rich interaction domain 5A
PPARGC1A	PPARG coactivator 1 alpha
BRD8	bromodomain containing 8
ADRM1	adhesion regulating molecule 1
SOX30	SRY-box 30
PRDM4	PR domain 4
KAT7	lysine acetyltransferase 7
SOX21	SRY-box 21
SNF8	SNF8, ESCRT-II complex subunit
KLF12	Kruppel-like factor 12
POU6F2	POU class 6 homeobox 2
ATF5	activating transcription factor 5
ZHX2	zinc fingers and homeoboxes 2
TRAK1	trafficking kinesin protein 1
ATF6	activating transcription factor 6
SCAP	SREBF chaperone
CRTC1	CREB regulated transcription coactivator 1
SIRT5	sirtuin 5
RYBP	RING1 and YY1 binding protein
TRIM29	tripartite motif containing 29
POU2F3	POU class 2 homeobox 3
SIN3A	SIN3 transcription regulator family member A
GMEB2	glucocorticoid modulatory element binding protein 2
EHF	ETS homologous factor
NUFIP1	NUFIP1, FMR1 interacting protein 1
FOXD3	forkhead box D3
MED4	mediator complex subunit 4
UHRF1	ubiquitin like with PHD and ring finger domains 1
ABT1	activator of basal transcription 1
UBN1	ubinuclein 1
MDFIC	MyoD family inhibitor domain containing
KCNIP3	potassium voltage-gated channel interacting protein 3
SMARCAL1	SWI/SNF related, matrix associated, actin dependent regulator of chromatin, subfamily a like 1
MYEF2	myelin expression factor 2
NDUFA13	NADH:ubiquinone oxidoreductase subunit A13
YBX2	Y-box binding protein 2
PHF21A	PHD finger protein 21A
KLF13	Kruppel-like factor 13
SUFU	SUFU negative regulator of hedgehog signaling
POLR3K	polymerase (RNA) III subunit K
SOX18	SRY-box 18
FEV	FEV, ETS transcription factor
ELP3	elongator acetyltransferase complex subunit 3
MAML3	mastermind like transcriptional coactivator 3
TRERF1	transcriptional regulating factor 1

ASH1L	ASH1 like histone lysine methyltransferase
SLC2A4RG	SLC2A4 regulator
CTNNBIP1	catenin beta interacting protein 1
NSD1	nuclear receptor binding SET domain protein 1
NAA15	N(alpha)-acetyltransferase 15, NatA auxiliary subunit
ELL3	elongation factor for RNA polymerase II 3
PBX4	PBX homeobox 4
SCRT1	scratch family transcriptional repressor 1
PCBD2	pterin-4 alpha-carbinolamine dehydratase 2
SLA2	Src-like-adaptor 2
MAML2	mastermind like transcriptional coactivator 2
AFAP1L2	actin filament associated protein 1 like 2
GTF3C6	general transcription factor IIIC subunit 6
TWIST2	twist family bHLH transcription factor 2
HEXIM2	hexamethylene bisacetamide inducible 2
GABPB2	GA binding protein transcription factor beta subunit 2
TAF8	TATA-box binding protein associated factor 8
GLIS3	GLIS family zinc finger 3
ZNF367	zinc finger protein 367
JMJD1C	jumonji domain containing 1C
JAZF1	JAZF zinc finger 1
ZBTB38	zinc finger and BTB domain containing 38
PTF1A	pancreas specific transcription factor, 1a
MAFA	MAF bZIP transcription factor A
**Regulation of gene expression**	
**Gene Symbol**	**Name Gene**
ABCA2	ATP binding cassette subfamily A member 2
ACVR1	activin A receptor type 1
ACVR2B	activin A receptor type 2B
ACVRL1	activin A receptor like type 1
AHR	aryl hydrocarbon receptor
APBB1	amyloid beta precursor protein binding family B member 1
APBB2	amyloid beta precursor protein binding family B member 2
ARNTL	aryl hydrocarbon receptor nuclear translocator like
ZFHX3	zinc finger homeobox 3
BCL3	B-cell CLL/lymphoma 3
BCL6	B-cell CLL/lymphoma 6
PRDM1	PR domain 1
BMP2	bone morphogenetic protein 2
BMP6	bone morphogenetic protein 6
BNIP3	BCL2/adenovirus E1B 19kDa interacting protein 3
BNIP3L	BCL2/adenovirus E1B 19kDa interacting protein 3-like
BRCA1	breast cancer 1
CALCA	calcitonin related polypeptide alpha
CALR	calreticulin
CAMK2A	calcium/calmodulin dependent protein kinase II alpha
RUNX2	runt related transcription factor 2
RUNX1	runt related transcription factor 1
TNFRSF8	tumor necrosis factor receptor superfamily member 8
CDK6	cyclin-dependent kinase 6
CDK7	cyclin-dependent kinase 7
CENPF	centromere protein F
CHD3	chromodomain helicase DNA binding protein 3
FOXN3	forkhead box N3
CREBBP	CREB binding protein
CREM	cAMP responsive element modulator
NKX2-5	NK2 homeobox 5

CUX1	cut like homeobox 1
DAXX	death-domain associated protein
DAZL	deleted in azoospermia like
DBP	D-box binding PAR bZIP transcription factor
DNMT1	DNA (cytosine-5-)-methyltransferase 1
DNMT3A	DNA (cytosine-5-)-methyltransferase 3 alpha
EIF4B	eukaryotic translation initiation factor 4B
EIF5	eukaryotic translation initiation factor 5
EIF5A	eukaryotic translation initiation factor 5A
ELAVL2	ELAV like neuron-specific RNA binding protein 2
ELF2	E74 like ETS transcription factor 2
ELK3	ELK3, ETS transcription factor
EP300	E1A binding protein p300
EPAS1	endothelial PAS domain protein 1
ERCC3	excision repair cross-complementation group 3
ERF	ETS2 repressor factor
ESRRG	estrogen related receptor gamma
EZH2	enhancer of zeste 2 polycomb repressive complex 2 subunit
BPTF	bromodomain PHD finger transcription factor
FOXF1	forkhead box F1
FOXF2	forkhead box F2
FOXO1	forkhead box O1
FOSL2	FOS like 2, AP-1 trancription factor subunit
NR5A1	nuclear receptor subfamily 5 group A member 1
XRCC6	X-ray repair complementing defective repair in Chinese hamster cells 6
GATA4	GATA binding protein 4
GATA6	GATA binding protein 6
KAT2A	lysine acetyltransferase 2A
NR6A1	nuclear receptor subfamily 6 group A member 1
GFI1	growth factor independent 1 transcriptional repressor
GHSR	growth hormone secretagogue receptor
GCLC	glutamate-cysteine ligase catalytic subunit
GLI2	GLI family zinc finger 2
GSC2	goosecoid homeobox 2
GTF2H1	general transcription factor IIH subunit 1
HDAC2	histone deacetylase 2
NRG1	neuregulin 1
HIC1	hypermethylated in cancer 1
MNX1	motor neuron and pancreas homeobox 1
HMGB1	high mobility group box 1
HMGB2	high mobility group box 2
HNF4A	hepatocyte nuclear factor 4 alpha
HNF4G	hepatocyte nuclear factor 4 gamma
HNRNPAB	heterogeneous nuclear ribonucleoprotein A/B
HOXC6	homeobox C6
IRF8	interferon regulatory factor 8
ID2	inhibitor of DNA binding 2, HLH protein
ID3	inhibitor of DNA binding 3, HLH protein
ID4	inhibitor of DNA binding 4, HLH protein
IKBKB	inhibitor of kappa light polypeptide gene enhancer in B-cells, kinase beta
IL12B	interleukin 12B
FOXK2	forkhead box K2
ILF2	interleukin enhancer binding factor 2
ILF3	interleukin enhancer binding factor 3
ING2	inhibitor of growth family member 2
INHBA	inhibin beta A
INHBB	inhibin beta B
EIF3E	eukaryotic translation initiation factor 3 subunit E

IRF2	interferon regulatory factor 2
IRF4	interferon regulatory factor 4
IRF7	interferon regulatory factor 7
LMX1B	LIM homeobox transcription factor 1 beta
LTB	lymphotoxin beta
SMAD2	SMAD family member 2
SMAD3	SMAD family member 3
MEIS2	Meis homeobox 2
MEN1	menin 1
MLLT6	myeloid/lymphoid or mixed-lineage leukemia; translocated to, 6
MYC	v-myc avian myelocytomatosis viral oncogene homolog
MYCN	v-myc avian myelocytomatosis viral oncogene neuroblastoma derived homolog
MYO6	myosin VI
MYOD1	myogenic differentiation 1
NEUROD2	neuronal differentiation 2
NEUROG1	neurogenin 1
NFATC1	nuclear factor of activated T-cells 1
NFATC2	nuclear factor of activated T-cells 2
NFYA	nuclear transcription factor Y subunit alpha
NFYC	nuclear transcription factor Y subunit gamma
NOTCH4	notch 4
NPAS2	neuronal PAS domain protein 2
NRF1	nuclear respiratory factor 1
NRL	neural retina leucine zipper
PAWR	pro-apoptotic WT1 regulator
PBX1	PBX homeobox 1
PBX3	PBX homeobox 3
PER1	period circadian clock 1
PEX14	peroxisomal biogenesis factor 14
PFDN5	prefoldin subunit 5
PITX2	paired like homeodomain 2
PLAGL1	PLAG1 like zinc finger 1
POU2F1	POU class 2 homeobox 1
POU5F1	POU class 5 homeobox 1
PPARD	peroxisome proliferator activated receptor delta
PPARG	peroxisome proliferator activated receptor gamma
RB1	retinoblastoma 1
RBBP8	retinoblastoma binding protein 8
RELA	RELA proto-oncogene, NF-kB subunit
UPF1	UPF1, RNA helicase and ATPase
REST	RE1 silencing transcription factor
TRIM27	tripartite motif containing 27
RNF4	ring finger protein 4
RORB	RAR related orphan receptor B
RREB1	ras responsive element binding protein 1
RXRA	retinoid X receptor alpha
ATXN1	ataxin 1
SNAI2	snail family transcriptional repressor 2
SMARCA2	SWI/SNF related, matrix associated, actin dependent regulator of chromatin, subfamily a, member 2
SMARCD2	SWI/SNF related, matrix associated, actin dependent regulator of chromatin, subfamily d, member 2
SMARCE1	SWI/SNF related, matrix associated, actin dependent regulator of chromatin, subfamily e, member 1
SOD2	superoxide dismutase 2, mitochondrial
SP1	Sp1 transcription factor
SP2	Sp2 transcription factor
SP3	Sp3 transcription factor

SP100	SP100 nuclear antigen
SPI1	Spi-1 proto-oncogene
SPIB	Spi-B transcription factor
SREBF1	sterol regulatory element binding transcription factor 1
SREBF2	sterol regulatory element binding transcription factor 2
SUPT5H	SPT5 homolog, DSIF elongation factor subunit
TARBP1	TAR (HIV-1) RNA binding protein 1
TARBP2	TARBP2, RISC loading complex RNA binding subunit
TBX5	T-box 5
TCEB1	transcription elongation factor B subunit 1
TBX3	T-box 3
HNF1A	HNF1 homeobox A
HNF1B	HNF1 homeobox B
TCF3	transcription factor 3
TCF7L2	transcription factor 7 like 2
ZEB1	zinc finger E-box binding homeobox 1
TCF12	transcription factor 12
ZNF354A	zinc finger protein 354A
TCF19	transcription factor 19
VPS72	vacuolar protein sorting 72 homolog
TFAP2A	transcription factor AP-2 alpha
TFAP2B	transcription factor AP-2 beta
NR2F2	nuclear receptor subfamily 2 group F member 2
TFDP1	transcription factor Dp-1
TGFB1	transforming growth factor beta 1
TGIF1	TGFB induced factor homeobox 1
KLF10	Kruppel-like factor 10
TIAL1	TIA1 cytotoxic granule-associated RNA binding protein-like 1
TLR3	toll like receptor 3
TNF	tumor necrosis factor
TP73	tumor protein p73
TSC1	tuberous sclerosis 1
HIRA	histone cell cycle regulator
TWIST1	twist family bHLH transcription factor 1
UBB	ubiquitin B
UBTF	upstream binding transcription factor, RNA polymerase I
NR1H2	nuclear receptor subfamily 1 group H member 2
YY1	YY1 transcription factor
YWHAH	tyrosine 3-monooxygenase/tryptophan 5-monooxygenase activation protein eta
ZNF45	zinc finger protein 45
ZNF74	zinc finger protein 74
ZBTB16	zinc finger and BTB domain containing 16
ZNF146	zinc finger protein 146
ZNF148	zinc finger protein 148
ZNF174	zinc finger protein 174
PAX8	paired box 8
KAT6A	lysine acetyltransferase 6A
ARID1A	AT-rich interaction domain 1A
KLF11	Kruppel-like factor 11
SUPT3H	SPT3 homolog, SAGA and STAGA complex component
IKBKAP	inhibitor of kappa light polypeptide gene enhancer in B-cells, kinase complex-associated protein
RUVBL1	RuvB like AAA ATPase 1
EIF3A	eukaryotic translation initiation factor 3 subunit A
EIF3G	eukaryotic translation initiation factor 3 subunit G
EIF3H	eukaryotic translation initiation factor 3 subunit H
EIF4G3	eukaryotic translation initiation factor 4 gamma 3
HRK	harakiri, BCL2 interacting protein

CREG1	cellular repressor of E1A stimulated genes 1
NR1I2	nuclear receptor subfamily 1 group I member 2
LDB1	LIM domain binding 1
SQSTM1	sequestosome 1
EIF2B2	eukaryotic translation initiation factor 2B subunit beta
BUD31	BUD31 homolog
RPS6KA4	ribosomal protein S6 kinase A4
TBX19	T-box 19
DYRK1B	dual specificity tyrosine phosphorylation regulated kinase 1B
MAP3K13	mitogen-activated protein kinase kinase kinase 13
LRRFIP1	leucine rich repeat (in FLII) interacting protein 1
KLF4	Kruppel-like factor 4 (gut)
ZNHIT3	zinc finger HIT-type containing 3
MED21	mediator complex subunit 21
MED17	mediator complex subunit 17
MED26	mediator complex subunit 26
PICK1	protein interacting with PRKCA 1
VPS4B	vacuolar protein sorting 4 homolog B
CIR1	corepressor interacting with RBPJ, 1
H2AFY	H2A histone family member Y
CREB5	cAMP responsive element binding protein 5
RNF14	ring finger protein 14
MTRF1	mitochondrial translational release factor 1
HDAC4	histone deacetylase 4
MAML1	mastermind like transcriptional coactivator 1
ARNT2	aryl hydrocarbon receptor nuclear translocator 2
MED13	mediator complex subunit 13
HDAC5	histone deacetylase 5
TRIM28	tripartite motif containing 28
RIDA	reactive intermediate imine deaminase A homolog
SAP18	Sin3A associated protein 18kDa
CITED2	Cbp/p300 interacting transactivator with Glu/Asp rich carboxy-terminal domain 2
FST	follistatin
TADA3	transcriptional adaptor 3
MYBBP1A	MYB binding protein 1a
IGF2BP2	insulin like growth factor 2 mRNA binding protein 2
CAMKK2	calcium/calmodulin-dependent protein kinase kinase 2
KHDRBS1	KH domain containing, RNA binding, signal transduction associated 1
TCFL5	transcription factor like 5
ZMYND11	zinc finger MYND-type containing 11
ZNF274	zinc finger protein 274
ARID5A	AT-rich interaction domain 5A
BRD8	bromodomain containing 8
SOX30	SRY-box 30
KAT7	lysine acetyltransferase 7
SOX21	SRY-box 21
SNF8	SNF8, ESCRT-II complex subunit
KLF12	Kruppel-like factor 12
POU6F2	POU class 6 homeobox 2
ATF5	activating transcription factor 5
ZHX2	zinc fingers and homeoboxes 2
TRAK1	trafficking kinesin protein 1
ATF6	activating transcription factor 6
SCAP	SREBF chaperone
SAMD4A	sterile alpha motif domain containing 4A
ERC1	ELKS/RAB6-interacting/CAST family member 1
ZNF423	zinc finger protein 423
CRTC1	CREB regulated transcription coactivator 1

DICER1	dicer 1, ribonuclease III
SIRT5	sirtuin 5
RYBP	RING1 and YY1 binding protein
KAT6B	lysine acetyltransferase 6B
RBFOX2	RNA binding protein, fox-1 homolog 2
POU2F3	POU class 2 homeobox 3
SIN3A	SIN3 transcription regulator family member A
EHF	ETS homologous factor
NUFIP1	NUFIP1, FMR1 interacting protein 1
FOXD3	forkhead box D3
STK36	serine/threonine kinase 36
UHRF1	ubiquitin like with PHD and ring finger domains 1
UBN1	ubinuclein 1
MDFIC	MyoD family inhibitor domain containing
KCNIP3	potassium voltage-gated channel interacting protein 3
SMARCAL1	SWI/SNF related, matrix associated, actin dependent regulator of chromatin, subfamily a like 1
NDUFA13	NADH:ubiquinone oxidoreductase subunit A13
YBX2	Y-box binding protein 2
SEPSECS	Sep (O-phosphoserine) tRNA:Sec (selenocysteine) tRNA synthase
PHF21A	PHD finger protein 21A
PIAS4	protein inhibitor of activated STAT 4
SUFU	SUFU negative regulator of hedgehog signaling
TLR9	toll like receptor 9
SOX18	SRY-box 18
DGCR8	DGCR8 microprocessor complex subunit
EGLN1	egl-9 family hypoxia inducible factor 1
TRNAU1AP	tRNA selenocysteine 1 associated protein 1
ELP3	elongator acetyltransferase complex subunit 3
H2AFY2	H2A histone family member Y2
MAML3	mastermind like transcriptional coactivator 3
TRERF1	transcriptional regulating factor 1
LANCL2	LanC like 2
SLC2A4RG	SLC2A4 regulator
CTNNBIP1	catenin beta interacting protein 1
HIVEP3	human immunodeficiency virus type I enhancer binding protein 3
SIGIRR	single immunoglobulin and toll-interleukin 1 receptor (TIR) domain
NECAB3	N-terminal EF-hand calcium binding protein 3
NSD1	nuclear receptor binding SET domain protein 1
SUDS3	SDS3 homolog, SIN3A corepressor complex component
NARFL	nuclear prelamin A recognition factor like
BOLL	boule homolog, RNA binding protein
NAA15	N(alpha)-acetyltransferase 15, NatA auxiliary subunit
ELL3	elongation factor for RNA polymerase II 3
PBX4	PBX homeobox 4
SCRT1	scratch family transcriptional repressor 1
PCBD2	pterin-4 alpha-carbinolamine dehydratase 2
SLA2	Src-like-adaptor 2
AKT1S1	AKT1 substrate 1
CARD11	caspase recruitment domain family member 11
MAML2	mastermind like transcriptional coactivator 2
AFAP1L2	actin filament associated protein 1 like 2
GLIS2	GLIS family zinc finger 2
NLRP3	NLR family, pyrin domain containing 3
TWIST2	twist family bHLH transcription factor 2
HEXIM2	hexamethylene bisacetamide inducible 2
GABPB2	GA binding protein transcription factor beta subunit 2
DMBX1	diencephalon/mesencephalon homeobox 1

TAF8	TATA-box binding protein associated factor 8
COMMD7	COMM domain containing 7
NFAM1	NFAT activating protein with ITAM motif 1
EID2	EP300 interacting inhibitor of differentiation 2
GLIS3	GLIS family zinc finger 3
ZNF367	zinc finger protein 367
NLRC3	NLR family, CARD domain containing 3
MTIF3	mitochondrial translational initiation factor 3
JMJD1C	jumonji domain containing 1C
JAZF1	JAZF zinc finger 1
ZBTB38	zinc finger and BTB domain containing 38
PTF1A	pancreas specific transcription factor, 1a
RGMB	repulsive guidance molecule family member b
MAFA	MAF bZIP transcription factor A
EIF2AK4	eukaryotic translation initiation factor 2 alpha kinase 4
**Regulation of catalytic activity**	
**Gene Symbol**	**Name Gene**
ABL2	ABL proto-oncogene 2, non-receptor tyrosine kinase
ADRA2A	adrenoceptor alpha 2A
ADRB1	adrenoceptor beta 1
ANG	angiogenin
APAF1	apoptotic peptidase activating factor 1
APC	adenomatous polyposis coli
BIRC5	baculoviral IAP repeat containing 5
CCND1	cyclin D1
FOXL2	forkhead box L2
CALCA	calcitonin related polypeptide alpha
CALCR	calcitonin receptor
CASP9	caspase 9
CCKBR	cholecystokinin B receptor
CCND2	cyclin D2
CCNG1	cyclin G1
CD81	CD81 molecule
CDK7	cyclin-dependent kinase 7
CDKN1C	cyclin-dependent kinase inhibitor 1C
CDKN3	cyclin-dependent kinase inhibitor 3
CHRM1	cholinergic receptor muscarinic 1
CKS1B	CDC28 protein kinase regulatory subunit 1B
CRHR1	corticotropin releasing hormone receptor 1
DAXX	death-domain associated protein
GADD45A	growth arrest and DNA damage inducible alpha
DHCR24	24-dehydrocholesterol reductase
DUSP2	dual specificity phosphatase 2
DUSP6	dual specificity phosphatase 6
EDNRB	endothelin receptor type B
EGF	epidermal growth factor
EGFR	epidermal growth factor receptor
GABBR1	gamma-aminobutyric acid type B receptor subunit 1
GALR1	galanin receptor 1
GHRHR	growth hormone releasing hormone receptor
GIPR	gastric inhibitory polypeptide receptor
GNA15	G protein subunit alpha 15
GNAI2	G protein subunit alpha i2
GNAS	GNAS complex locus
GPS1	G protein pathway suppressor 1
GRM2	glutamate metabotropic receptor 2
GRM3	glutamate metabotropic receptor 3

GRM4	glutamate metabotropic receptor 4
GTF2H1	general transcription factor IIH subunit 1
HIP1	huntingtin interacting protein 1
LCK	LCK proto-oncogene, Src family tyrosine kinase
SMAD3	SMAD family member 3
MAP3K5	mitogen-activated protein kinase kinase kinase 5
MAP3K10	mitogen-activated protein kinase kinase kinase 10
NF1	neurofibromin 1
PAK1	p21 (RAC1) activated kinase 1
PAK2	p21 (RAC1) activated kinase 2
PIK3CB	phosphatidylinositol-4,5-bisphosphate 3-kinase catalytic subunit beta
PMAIP1	phorbol-12-myristate-13-acetate-induced protein 1
PRLR	prolactin receptor
PTEN	phosphatase and tensin homolog
PTPRC	protein tyrosine phosphatase, receptor type C
RB1	retinoblastoma 1
RGS3	regulator of G-protein signaling 3
SHC1	SHC adaptor protein 1
TARBP2	TARBP2, RISC loading complex RNA binding subunit
TGFA	transforming growth factor alpha
TNNT2	troponin T2, cardiac type
TPD52L1	tumor protein D52-like 1
TRAF6	TNF receptor associated factor 6
TSC1	tuberous sclerosis 1
MADD	MAP kinase activating death domain
PKMYT1	protein kinase, membrane associated tyrosine/threonine 1
CCNE2	cyclin E2
LPAR2	lysophosphatidic acid receptor 2
MAP3K13	mitogen-activated protein kinase kinase kinase 13
PICK1	protein interacting with PRKCA 1
GABBR2	gamma-aminobutyric acid type B receptor subunit 2
CASP8AP2	caspase 8 associated protein 2
DNAJB6	DnaJ heat shock protein family (Hsp40) member B6
HIPK3	homeodomain interacting protein kinase 3
TRIB1	tribbles pseudokinase 1
NMUR1	neuromedin U receptor 1
TAB1	TGF-beta activated kinase 1/MAP3K7 binding protein 1
CAP1	CAP, adenylate cyclase-associated protein 1 (yeast)
CAMKK2	calcium/calmodulin-dependent protein kinase kinase 2
GADD45G	growth arrest and DNA damage inducible gamma
RALBP1	ralA binding protein 1
ADAP1	ArfGAP with dual PH domains 1
CDC37	cell division cycle 37
CBLC	Cbl proto-oncogene C
FBXO5	F-box protein 5
LATS2	large tumor suppressor kinase 2
SERTAD1	SERTA domain containing 1
MDFIC	MyoD family inhibitor domain containing
MBIP	MAP3K12 binding inhibitory protein 1
ZAK	sterile alpha motif and leucine zipper containing kinase AZK
IFT57	intraflagellar transport 57
CAND1	cullin associated and neddylation dissociated 1
PSENEN	presenilin enhancer gamma-secretase subunit
PARD3	par-3 family cell polarity regulator
LTB4R2	leukotriene B4 receptor 2
BCCIP	BRCA2 and CDKN1A interacting protein
DUSP22	dual specificity phosphatase 22
ALS2	ALS2, alsin Rho guanine nucleotide exchange factor

PIF1	PIF1 5'-to-3' DNA helicase
CDK5RAP3	CDK5 regulatory subunit associated protein 3
ATPIF1	ATPase inhibitory factor 1
NLRP3	NLR family, pyrin domain containing 3
ARAP1	ArfGAP with RhoGAP domain, ankyrin repeat and PH domain 1
HEXIM2	hexamethylene bisacetamide inducible 2
AIFM3	apoptosis inducing factor, mitochondria associated 3
FGD5	FYVE, RhoGEF and PH domain containing 5
SPRED2	sprouty related EVH1 domain containing 2
ARHGAP27	Rho GTPase activating protein 27
FGD2	FYVE, RhoGEF and PH domain containing 2
CRIPAK	cysteine rich PAK1 inhibitor
SERINC2	serine incorporator 2
**Transcription**	
**Gene Symbol**	**Name Gene**
ABCA2	ATP binding cassette subfamily A member 2
ACVR1	activin A receptor type 1
ACVR2B	activin A receptor type 2B
ACVRL1	activin A receptor like type 1
PARP1	poly(ADP-ribose) polymerase 1
AHR	aryl hydrocarbon receptor
ANG	angiogenin
APBB1	amyloid beta precursor protein binding family B member 1
APBB2	amyloid beta precursor protein binding family B member 2
APEX1	apurinic/apyrimidinic endodeoxyribonuclease 1
ARNTL	aryl hydrocarbon receptor nuclear translocator like
ZFHX3	zinc finger homeobox 3
ATF4	activating transcription factor 4
NKX3-2	NK3 homeobox 2
BCL3	B-cell CLL/lymphoma 3
BCL6	B-cell CLL/lymphoma 6
PRDM1	PR domain 1
BMP2	bone morphogenetic protein 2
BMP6	bone morphogenetic protein 6
BRCA1	breast cancer 1
BTF3	basic transcription factor 3
CALCA	calcitonin related polypeptide alpha
CALR	calreticulin
CAMK2A	calcium/calmodulin dependent protein kinase II alpha
RUNX2	runt related transcription factor 2
RUNX1	runt related transcription factor 1
RUNX3	runt related transcription factor 3
CBFB	core-binding factor, beta subunit
CDK7	cyclin-dependent kinase 7
CEBPA	CCAAT/enhancer binding protein alpha
CEBPB	CCAAT/enhancer binding protein beta
CENPF	centromere protein F
CHD3	chromodomain helicase DNA binding protein 3
FOXN3	forkhead box N3
CREBBP	CREB binding protein
CREM	cAMP responsive element modulator
NKX2-5	NK2 homeobox 5
CUX1	cut like homeobox 1
DAXX	death-domain associated protein
DBP	D-box binding PAR bZIP transcription factor
DNMT1	DNA (cytosine-5-)-methyltransferase 1
DTX1	deltex 1

E2F2	E2F transcription factor 2
E2F3	E2F transcription factor 3
ELAVL2	ELAV like neuron-specific RNA binding protein 2
ELF2	E74 like ETS transcription factor 2
ELK3	ELK3, ETS transcription factor
EP300	E1A binding protein p300
EPAS1	endothelial PAS domain protein 1
ERCC3	excision repair cross-complementation group 3
ERCC6	excision repair cross-complementation group 6
ERF	ETS2 repressor factor
ESRRG	estrogen related receptor gamma
EZH2	enhancer of zeste 2 polycomb repressive complex 2 subunit
BPTF	bromodomain PHD finger transcription factor
FOXF1	forkhead box F1
FOXF2	forkhead box F2
FOXE3	forkhead box E3
FOXO1	forkhead box O1
FOSL2	FOS like 2, AP-1 trancription factor subunit
NR5A2	nuclear receptor subfamily 5 group A member 2
NR5A1	nuclear receptor subfamily 5 group A member 1
XRCC6	X-ray repair complementing defective repair in Chinese hamster cells 6
GATA2	GATA binding protein 2
GATA3	GATA binding protein 3
GATA4	GATA binding protein 4
GATA6	GATA binding protein 6
KAT2A	lysine acetyltransferase 2A
NR6A1	nuclear receptor subfamily 6 group A member 1
GFI1	growth factor independent 1 transcriptional repressor
GCLC	glutamate-cysteine ligase catalytic subunit
GLI2	GLI family zinc finger 2
NR3C1	nuclear receptor subfamily 3 group C member 1
GSC2	goosecoid homeobox 2
GTF2B	general transcription factor IIB
GTF2H1	general transcription factor IIH subunit 1
GTF2I	general transcription factor IIi
BRF1	BRF1, RNA polymerase III transcription initiation factor 90 kDa subunit
HDAC2	histone deacetylase 2
NRG1	neuregulin 1
HIC1	hypermethylated in cancer 1
MNX1	motor neuron and pancreas homeobox 1
HMGB1	high mobility group box 1
HMGB2	high mobility group box 2
HMGA1	high mobility group AT-hook 1
HNF4A	hepatocyte nuclear factor 4 alpha
HNF4G	hepatocyte nuclear factor 4 gamma
HNRNPAB	heterogeneous nuclear ribonucleoprotein A/B
HOXC6	homeobox C6
IRF8	interferon regulatory factor 8
ID2	inhibitor of DNA binding 2, HLH protein
ID3	inhibitor of DNA binding 3, HLH protein
ID4	inhibitor of DNA binding 4, HLH protein
IKBKB	inhibitor of kappa light polypeptide gene enhancer in B-cells, kinase beta
FOXK2	forkhead box K2
ILF2	interleukin enhancer binding factor 2
ILF3	interleukin enhancer binding factor 3
ING2	inhibitor of growth family member 2
INHBA	inhibin beta A
IRF1	interferon regulatory factor 1

IRF2	interferon regulatory factor 2
IRF4	interferon regulatory factor 4
IRF7	interferon regulatory factor 7
LMX1B	LIM homeobox transcription factor 1 beta
SMAD2	SMAD family member 2
SMAD3	SMAD family member 3
MAX	MYC associated factor X
MEIS2	Meis homeobox 2
MEN1	menin 1
MLLT1	myeloid/lymphoid or mixed-lineage leukemia; translocated to, 1
MLLT6	myeloid/lymphoid or mixed-lineage leukemia; translocated to, 6
MYC	v-myc avian myelocytomatosis viral oncogene homolog
MYCN	v-myc avian myelocytomatosis viral oncogene neuroblastoma derived homolog
MYO6	myosin VI
MYOD1	myogenic differentiation 1
NEUROD2	neuronal differentiation 2
NEUROG1	neurogenin 1
NFATC1	nuclear factor of activated T-cells 1
NFATC2	nuclear factor of activated T-cells 2
NFATC4	nuclear factor of activated T-cells 4
NFIC	nuclear factor I C
NFIX	nuclear factor I X
NFKB1	nuclear factor kappa B subunit 1
NFKBIB	NFKB inhibitor beta
NFRKB	nuclear factor related to kappaB binding protein
NFYA	nuclear transcription factor Y subunit alpha
NFYC	nuclear transcription factor Y subunit gamma
NOTCH4	notch 4
NPAS2	neuronal PAS domain protein 2
NRF1	nuclear respiratory factor 1
NRL	neural retina leucine zipper
PAWR	pro-apoptotic WT1 regulator
PAX1	paired box 1
PAX2	paired box 2
PBX1	PBX homeobox 1
PBX3	PBX homeobox 3
PER1	period circadian clock 1
PEX14	peroxisomal biogenesis factor 14
PFDN5	prefoldin subunit 5
PITX2	paired like homeodomain 2
PLAGL1	PLAG1 like zinc finger 1
POLR2D	polymerase (RNA) II subunit D
POLR2F	polymerase (RNA) II subunit F
POLR2G	polymerase (RNA) II subunit G
POLR2H	polymerase (RNA) II subunit H
POLR2J	polymerase (RNA) II subunit J
POLRMT	polymerase (RNA) mitochondrial
POU2AF1	POU class 2 associating factor 1
POU2F1	POU class 2 homeobox 1
POU5F1	POU class 5 homeobox 1
PPARA	peroxisome proliferator activated receptor alpha
PPARD	peroxisome proliferator activated receptor delta
PPARG	peroxisome proliferator activated receptor gamma
PPP5C	protein phosphatase 5 catalytic subunit
PSMC5	proteasome 26S subunit, ATPase 5
PTGER3	prostaglandin E receptor 3
PTMA	prothymosin, alpha
RB1	retinoblastoma 1

RBBP8	retinoblastoma binding protein 8
RELA	RELA proto-oncogene, NF-kB subunit
REST	RE1 silencing transcription factor
TRIM27	tripartite motif containing 27
RNF4	ring finger protein 4
RORB	RAR related orphan receptor B
RREB1	ras responsive element binding protein 1
RXRA	retinoid X receptor alpha
ATXN1	ataxin 1
SNAI2	snail family transcriptional repressor 2
SMARCA2	SWI/SNF related, matrix associated, actin dependent regulator of chromatin, subfamily a, member 2
SMARCD2	SWI/SNF related, matrix associated, actin dependent regulator of chromatin, subfamily d, member 2
SMARCE1	SWI/SNF related, matrix associated, actin dependent regulator of chromatin, subfamily e, member 1
SNAPC2	small nuclear RNA activating complex polypeptide 2
SNAPC3	small nuclear RNA activating complex polypeptide 3
SOD2	superoxide dismutase 2, mitochondrial
SP1	Sp1 transcription factor
SP2	Sp2 transcription factor
SP3	Sp3 transcription factor
SP100	SP100 nuclear antigen
SPI1	Spi-1 proto-oncogene
SPIB	Spi-B transcription factor
SREBF1	sterol regulatory element binding transcription factor 1
SREBF2	sterol regulatory element binding transcription factor 2
SUPT5H	SPT5 homolog, DSIF elongation factor subunit
TAF5	TATA-box binding protein associated factor 5
TARBP1	TAR (HIV-1) RNA binding protein 1
TARBP2	TARBP2, RISC loading complex RNA binding subunit
TBP	TATA-box binding protein
TBX5	T-box 5
TCEB1	transcription elongation factor B subunit 1
TCEB2	transcription elongation factor B subunit 2
TBX3	T-box 3
HNF1A	HNF1 homeobox A
HNF1B	HNF1 homeobox B
TCF3	transcription factor 3
TCF7L2	transcription factor 7 like 2
ZEB1	zinc finger E-box binding homeobox 1
TCF12	transcription factor 12
ZNF354A	zinc finger protein 354A
TCF19	transcription factor 19
VPS72	vacuolar protein sorting 72 homolog
TFAP2A	transcription factor AP-2 alpha
TFAP2B	transcription factor AP-2 beta
NR2F2	nuclear receptor subfamily 2 group F member 2
TFDP1	transcription factor Dp-1
TGFB1	transforming growth factor beta 1
TGIF1	TGFB induced factor homeobox 1
KLF10	Kruppel-like factor 10
TIAL1	TIA1 cytotoxic granule-associated RNA binding protein-like 1
TNF	tumor necrosis factor
TP73	tumor protein p73
TRPS1	transcriptional repressor GATA binding 1
HIRA	histone cell cycle regulator
TWIST1	twist family bHLH transcription factor 1

UBB	ubiquitin B
UBTF	upstream binding transcription factor, RNA polymerase I
NR1H2	nuclear receptor subfamily 1 group H member 2
USF1	upstream transcription factor 1
YY1	YY1 transcription factor
YWHAH	tyrosine 3-monooxygenase/tryptophan 5-monooxygenase activation protein eta
ZNF45	zinc finger protein 45
ZNF74	zinc finger protein 74
ZBTB16	zinc finger and BTB domain containing 16
ZNF146	zinc finger protein 146
ZNF148	zinc finger protein 148
ZNF174	zinc finger protein 174
PAX8	paired box 8
KAT6A	lysine acetyltransferase 6A
ALX1	ALX homeobox 1
ELL	elongation factor for RNA polymerase II
ARID1A	AT-rich interaction domain 1A
ZNF282	zinc finger protein 282
KLF11	Kruppel-like factor 11
SUPT3H	SPT3 homolog, SAGA and STAGA complex component
IKBKAP	inhibitor of kappa light polypeptide gene enhancer in B-cells, kinase complex-associated protein
RUVBL1	RuvB like AAA ATPase 1
EDF1	endothelial differentiation related factor 1
CREG1	cellular repressor of E1A stimulated genes 1
TRIM24	tripartite motif containing 24
TSC22D1	TSC22 domain family member 1
NR1I2	nuclear receptor subfamily 1 group I member 2
LDB1	LIM domain binding 1
SQSTM1	sequestosome 1
FUBP1	far upstream element binding protein 1
BUD31	BUD31 homolog
RPS6KA4	ribosomal protein S6 kinase A4
ASH2L	ASH2 like histone lysine methyltransferase complex subunit
TBX19	T-box 19
NMI	N-myc and STAT interactor
DYRK1B	dual specificity tyrosine phosphorylation regulated kinase 1B
MAP3K13	mitogen-activated protein kinase kinase kinase 13
LRRFIP1	leucine rich repeat (in FLII) interacting protein 1
PTTG1	pituitary tumor-transforming 1
MSC	musculin
KLF4	Kruppel-like factor 4 (gut)
TRIP13	thyroid hormone receptor interactor 13
TRIP11	thyroid hormone receptor interactor 11
ZNHIT3	zinc finger HIT-type containing 3
MED21	mediator complex subunit 21
MED17	mediator complex subunit 17
MED26	mediator complex subunit 26
HAND2	heart and neural crest derivatives expressed 2
VPS4B	vacuolar protein sorting 4 homolog B
POLR1C	polymerase (RNA) I subunit C
CIR1	corepressor interacting with RBPJ, 1
CREB5	cAMP responsive element binding protein 5
NFE2L3	nuclear factor, erythroid 2 like 3
RNF14	ring finger protein 14
HDAC4	histone deacetylase 4
MAML1	mastermind like transcriptional coactivator 1
MED24	mediator complex subunit 24

ARNT2	aryl hydrocarbon receptor nuclear translocator 2
MED13	mediator complex subunit 13
HDAC5	histone deacetylase 5
MED16	mediator complex subunit 16
TRIM28	tripartite motif containing 28
SAP18	Sin3A associated protein 18kDa
CITED2	Cbp/p300 interacting transactivator with Glu/Asp rich carboxy-terminal domain 2
FST	follistatin
TADA3	transcriptional adaptor 3
MYBBP1A	MYB binding protein 1a
DEAF1	DEAF1, transcription factor
KAT5	lysine acetyltransferase 5
IVNS1ABP	influenza virus NS1A binding protein
CAMKK2	calcium/calmodulin-dependent protein kinase kinase 2
KHDRBS1	KH domain containing, RNA binding, signal transduction associated 1
TCFL5	transcription factor like 5
ZMYND11	zinc finger MYND-type containing 11
ZNF274	zinc finger protein 274
CD3EAP	CD3e molecule associated protein
ARID5A	AT-rich interaction domain 5A
PPARGC1A	PPARG coactivator 1 alpha
BRD8	bromodomain containing 8
RNPS1	RNA binding protein with serine rich domain 1
ADRM1	adhesion regulating molecule 1
SOX30	SRY-box 30
PRDM4	PR domain 4
KAT7	lysine acetyltransferase 7
SOX21	SRY-box 21
SNF8	SNF8, ESCRT-II complex subunit
KLF12	Kruppel-like factor 12
POU6F2	POU class 6 homeobox 2
ATF5	activating transcription factor 5
ZHX2	zinc fingers and homeoboxes 2
TRAK1	trafficking kinesin protein 1
ATF6	activating transcription factor 6
SCAP	SREBF chaperone
ERC1	ELKS/RAB6-interacting/CAST family member 1
ZNF423	zinc finger protein 423
CRTC1	CREB regulated transcription coactivator 1
SIRT5	sirtuin 5
RYBP	RING1 and YY1 binding protein
KAT6B	lysine acetyltransferase 6B
RBFOX2	RNA binding protein, fox-1 homolog 2
TRIM29	tripartite motif containing 29
POU2F3	POU class 2 homeobox 3
SIN3A	SIN3 transcription regulator family member A
GMEB2	glucocorticoid modulatory element binding protein 2
EHF	ETS homologous factor
NUFIP1	NUFIP1, FMR1 interacting protein 1
FOXD3	forkhead box D3
STK36	serine/threonine kinase 36
MED4	mediator complex subunit 4
UHRF1	ubiquitin like with PHD and ring finger domains 1
ABT1	activator of basal transcription 1
UBN1	ubinuclein 1
MDFIC	MyoD family inhibitor domain containing
KCNIP3	potassium voltage-gated channel interacting protein 3
SMARCAL1	SWI/SNF related, matrix associated, actin dependent regulator of chromatin,

	subfamily a like 1
MYEF2	myelin expression factor 2
NDUFA13	NADH:ubiquinone oxidoreductase subunit A13
YBX2	Y-box binding protein 2
PHF21A	PHD finger protein 21A
PIAS4	protein inhibitor of activated STAT 4
KLF13	Kruppel-like factor 13
SUFU	SUFU negative regulator of hedgehog signaling
POLR3K	polymerase (RNA) III subunit K
SOX18	SRY-box 18
EGLN1	egl-9 family hypoxia inducible factor 1
FEV	FEV, ETS transcription factor
ELP3	elongator acetyltransferase complex subunit 3
ARHGEF10L	Rho guanine nucleotide exchange factor 10 like
MAML3	mastermind like transcriptional coactivator 3
TRERF1	transcriptional regulating factor 1
ASH1L	ASH1 like histone lysine methyltransferase
LANCL2	LanC like 2
SLC2A4RG	SLC2A4 regulator
CTNNBIP1	catenin beta interacting protein 1
MKL2	MKL1/myocardin like 2
HIVEP3	human immunodeficiency virus type I enhancer binding protein 3
SIGIRR	single immunoglobulin and toll-interleukin 1 receptor (TIR) domain
APOBEC3G	apolipoprotein B mRNA editing enzyme catalytic subunit 3G
NOD2	nucleotide binding oligomerization domain containing 2
NSD1	nuclear receptor binding SET domain protein 1
SUDS3	SDS3 homolog, SIN3A corepressor complex component
NARFL	nuclear prelamin A recognition factor like
NAA15	N(alpha)-acetyltransferase 15, NatA auxiliary subunit
ELL3	elongation factor for RNA polymerase II 3
PBX4	PBX homeobox 4
SCRT1	scratch family transcriptional repressor 1
PCBD2	pterin-4 alpha-carbinolamine dehydratase 2
SLA2	Src-like-adaptor 2
CARD11	caspase recruitment domain family member 11
MAML2	mastermind like transcriptional coactivator 2
AFAP1L2	actin filament associated protein 1 like 2
GLIS2	GLIS family zinc finger 2
GTF3C6	general transcription factor IIIC subunit 6
NLRP3	NLR family, pyrin domain containing 3
TWIST2	twist family bHLH transcription factor 2
HEXIM2	hexamethylene bisacetamide inducible 2
GABPB2	GA binding protein transcription factor beta subunit 2
DMBX1	diencephalon/mesencephalon homeobox 1
TAF8	TATA-box binding protein associated factor 8
GLIS1	GLIS family zinc finger 1
COMMD7	COMM domain containing 7
NFAM1	NFAT activating protein with ITAM motif 1
EID2	EP300 interacting inhibitor of differentiation 2
GLIS3	GLIS family zinc finger 3
ZNF367	zinc finger protein 367
NLRC3	NLR family, CARD domain containing 3
JMJD1C	jumonji domain containing 1C
JAZF1	JAZF zinc finger 1
ZBTB38	zinc finger and BTB domain containing 38
PTF1A	pancreas specific transcription factor, 1a
RGMB	repulsive guidance molecule family member b
MAFA	MAF bZIP transcription factor A

Central Nervous system development	
**Gene Symbol**	**Name Gene**
ALDH3A2	aldehyde dehydrogenase 3 family member A2
CLN5	ceroid-lipofuscinosis, neuronal 5
DLX2	distal-less homeobox 2
RCAN1	regulator of calcineurin 1
EGR2	early growth response 2
FOXG1	forkhead box G1
GLI2	GLI family zinc finger 2
JARID2	jumonji and AT-rich interaction domain containing 2
MAL	mal, T-cell differentiation protein
MBP	myelin basic protein
MOG	myelin oligodendrocyte glycoprotein
NF1	neurofibromin 1
NHLH2	nescient helix-loop-helix 2
NPTX1	neuronal pentraxin 1
OTX2	orthodenticle homeobox 2
PAX6	paired box 6
PBX1	PBX homeobox 1
PBX3	PBX homeobox 3
POU3F3	POU class 3 homeobox 3
POU6F1	POU class 6 homeobox 1
PROP1	PROP paired-like homeobox 1
PTEN	phosphatase and tensin homolog
PTPRZ1	protein tyrosine phosphatase, receptor type Z1
PTS	6-pyruvoyltetrahydropterin synthase
ROBO2	roundabout guidance receptor 2
SH3GL2	SH3 domain containing GRB2 like 2, endophilin A1
SH3GL3	SH3 domain containing GRB2 like endophilin A3
SHH	sonic hedgehog
SIX3	SIX homeobox 3
SLIT1	slit guidance ligand 1
SLIT3	slit guidance ligand 3
ZIC1	Zic family member 1
ZIC2	Zic family member 2
ZBTB16	zinc finger and BTB domain containing 16
ALX1	ALX homeobox 1
EIF2B3	eukaryotic translation initiation factor 2B subunit gamma
EIF2B2	eukaryotic translation initiation factor 2B subunit beta
PITPNM1	phosphatidylinositol transfer protein membrane associated 1
CELSR1	cadherin EGF LAG seven-pass G-type receptor 1
ECE2	endothelin converting enzyme 2
ARNT2	aryl hydrocarbon receptor nuclear translocator 2
NCKAP1	NCK associated protein 1
PPP1R17	protein phosphatase 1 regulatory subunit 17
POU6F2	POU class 6 homeobox 2
LHX6	LIM homeobox 6
SOX8	SRY-box 8
SNTG2	syntrophin gamma 2
PCDH18	protocadherin 18
PDGFC	platelet derived growth factor C
DSCAML1	DS cell adhesion molecule like 1
PBX4	PBX homeobox 4
FOXP2	forkhead box P2
DMBX1	diencephalon/mesencephalon homeobox 1
CNTN4	contactin 4
MDGA1	MAM domain containing glycosylphosphatidylinositol anchor 1

Nervous system development	
**Gene Symbol**	**Name Gene**
ADORA1	adenosine A1 receptor
ALDH3A2	aldehyde dehydrogenase 3 family member A2
APAF1	apoptotic peptidase activating factor 1
APBA1	amyloid beta precursor protein binding family A member 1
APBA2	amyloid beta precursor protein binding family A member 2
ATP2B2	ATPase plasma membrane Ca2+ transporting 2
BDNF	brain-derived neurotrophic factor
CD9	CD9 molecule
CDK6	cyclin-dependent kinase 6
CHRM1	cholinergic receptor muscarinic 1
CHRM2	cholinergic receptor muscarinic 2
CHRM3	cholinergic receptor muscarinic 3
CHRNA4	cholinergic receptor nicotinic alpha 4 subunit
CLN5	ceroid-lipofuscinosis, neuronal 5
CNTFR	ciliary neurotrophic factor receptor
CRMP1	collapsin response mediator protein 1
DLG4	discs large homolog 4
DLX2	distal-less homeobox 2
DLX5	distal-less homeobox 5
DPYSL2	dihydropyrimidinase like 2
DRD2	dopamine receptor D2
ATN1	atrophin 1
RCAN1	regulator of calcineurin 1
DTX1	deltex 1
DVL3	dishevelled segment polarity protein 3
EFNA5	ephrin A5
EGR2	early growth response 2
EP300	E1A binding protein p300
EPHB1	EPH receptor B1
EPHB2	EPH receptor B2
CLN8	ceroid-lipofuscinosis, neuronal 8
BPTF	bromodomain PHD finger transcription factor
FGF5	fibroblast growth factor 5
FGF11	fibroblast growth factor 11
FGF12	fibroblast growth factor 12
FGF14	fibroblast growth factor 14
FOXG1	forkhead box G1
GDNF	glial cell derived neurotrophic factor
GLI2	GLI family zinc finger 2
CXCL1	C-X-C motif chemokine ligand 1
GSTP1	glutathione S-transferase pi 1
JARID2	jumonji and AT-rich interaction domain containing 2
KCNQ2	potassium voltage-gated channel subfamily Q member 2
LAMB1	laminin subunit beta 1
LHX1	LIM homeobox 1
LMX1B	LIM homeobox transcription factor 1 beta
LSAMP	limbic system-associated membrane protein
LY6H	lymphocyte antigen 6 complex, locus H
MAL	mal, T-cell differentiation protein
MAPT	microtubule associated protein tau
MBNL1	muscleblind like splicing regulator 1
MBP	myelin basic protein
MOG	myelin oligodendrocyte glycoprotein
NAB2	NGFI-A binding protein 2
NEUROD2	neuronal differentiation 2

NEUROG1	neurogenin 1
NF1	neurofibromin 1
NF2	neurofibromin 2 (merlin)
NHLH2	nescient helix-loop-helix 2
NPAS2	neuronal PAS domain protein 2
NPTX1	neuronal pentraxin 1
NRCAM	neuronal cell adhesion molecule
NRGN	neurogranin
NTF3	neurotrophin 3
OTX2	orthodenticle homeobox 2
PAFAH1B1	platelet activating factor acetylhydrolase 1b regulatory subunit 1
PAX2	paired box 2
PAX6	paired box 6
PBX1	PBX homeobox 1
PBX3	PBX homeobox 3
PHYH	phytanoyl-CoA 2-hydroxylase
POU3F3	POU class 3 homeobox 3
POU6F1	POU class 6 homeobox 1
PPARD	peroxisome proliferator activated receptor delta
PROP1	PROP paired-like homeobox 1
PTEN	phosphatase and tensin homolog
PTN	pleiotrophin
PTPRZ1	protein tyrosine phosphatase, receptor type Z1
PTS	6-pyruvoyltetrahydropterin synthase
ROBO1	roundabout guidance receptor 1
ROBO2	roundabout guidance receptor 2
SCN8A	sodium voltage-gated channel alpha subunit 8
SH3GL2	SH3 domain containing GRB2 like 2, endophilin A1
SH3GL3	SH3 domain containing GRB2 like endophilin A3
SHH	sonic hedgehog
SIAH1	siah E3 ubiquitin protein ligase 1
SIM1	single-minded family bHLH transcription factor 1
SIM2	single-minded family bHLH transcription factor 2
SIX3	SIX homeobox 3
SLIT1	slit guidance ligand 1
SLIT3	slit guidance ligand 3
SMPD1	sphingomyelin phosphodiesterase 1
SOX11	SRY-box 11
SPG7	SPG7, paraplegin matrix AAA peptidase subunit
SPOCK1	sparc/osteonectin, cwcv and kazal-like domains proteoglycan (testican) 1
TFAP2B	transcription factor AP-2 beta
TGFB2	transforming growth factor beta 2
NR2E1	nuclear receptor subfamily 2 group E member 1
UBB	ubiquitin B
VEGFA	vascular endothelial growth factor A
YWHAG	tyrosine 3-monooxygenase/tryptophan 5-monooxygenase activation protein gamma
YWHAH	tyrosine 3-monooxygenase/tryptophan 5-monooxygenase activation protein eta
ZIC1	Zic family member 1
ZIC2	Zic family member 2
ZBTB16	zinc finger and BTB domain containing 16
SEMA3B	semaphorin 3B
ALX1	ALX homeobox 1
ST8SIA2	ST8 alpha-N-acetyl-neuraminide alpha-2,8-sialyltransferase 2
SOX14	SRY-box 14
NRP2	neuropilin 2
NRP1	neuropilin 1
LDB1	LIM domain binding 1
EIF2B3	eukaryotic translation initiation factor 2B subunit gamma

EIF2B2	eukaryotic translation initiation factor 2B subunit beta
PHOX2B	paired like homeobox 2b
KALRN	kalirin, RhoGEF kinase
BRSK2	BR serine/threonine kinase 2
ARTN	artemin
LGI1	leucine-rich, glioma inactivated 1
NOG	noggin
NUMBL	NUMB like, endocytic adaptor protein
NRXN3	neurexin 3
NRXN1	neurexin 1
PICK1	protein interacting with PRKCA 1
PITPNM1	phosphatidylinositol transfer protein membrane associated 1
CELSR1	cadherin EGF LAG seven-pass G-type receptor 1
FEZ1	fasciculation and elongation protein zeta 1
ECE2	endothelin converting enzyme 2
HDAC4	histone deacetylase 4
ARNT2	aryl hydrocarbon receptor nuclear translocator 2
FGF19	fibroblast growth factor 19
SPON2	spondin 2
OLFM1	olfactomedin 1
BAIAP2	BAI1 associated protein 2
NCKAP1	NCK associated protein 1
PPP1R17	protein phosphatase 1 regulatory subunit 17
CYP46A1	cytochrome P450 family 46 subfamily A member 1
CIT	citron rho-interacting serine/threonine kinase
GPR45	G protein-coupled receptor 45
POU6F2	POU class 6 homeobox 2
NLGN1	neuroligin 1
MYO16	myosin XVI
ATXN10	ataxin 10
LHX6	LIM homeobox 6
RND1	Rho family GTPase 1
MYLIP	myosin regulatory light chain interacting protein
TMOD2	tropomodulin 2
SOX8	SRY-box 8
STMN3	stathmin 3
TRAPPC4	trafficking protein particle complex 4
HDAC7	histone deacetylase 7
ADAM22	ADAM metallopeptidase domain 22
SNTG2	syntrophin gamma 2
PCDH18	protocadherin 18
MAML3	mastermind like transcriptional coactivator 3
PDGFC	platelet derived growth factor C
PCDHB13	protocadherin beta 13
PCDHB3	protocadherin beta 3
PCDHB2	protocadherin beta 2
PCDHA1	protocadherin alpha 1
PARD3	par-3 family cell polarity regulator
VANGL2	VANGL planar cell polarity protein 2
DSCAML1	DS cell adhesion molecule like 1
ALS2	ALS2, alsin Rho guanine nucleotide exchange factor
LRRC4C	leucine rich repeat containing 4C
PCDHB16	protocadherin beta 16
MARK4	microtubule affinity regulating kinase 4
PBX4	PBX homeobox 4
FOXP2	forkhead box P2
DMBX1	diencephalon/mesencephalon homeobox 1
RBM45	RNA binding motif protein 45

RTN4RL1	reticulon 4 receptor like 1
CNTN4	contactin 4
MDGA2	MAM domain containing glycosylphosphatidylinositol anchor 2
ZNF384	zinc finger protein 384
PCSK9	proprotein convertase subtilisin/kexin type 9
MDGA1	MAM domain containing glycosylphosphatidylinositol anchor 1
RTN4RL2	reticulon 4 receptor-like 2
AGRN	agrin

Table S4 – Gene collection of Remittent group

**Table S4** - Gene collection of Remittent group	
**Biological processes**	
**System development**	
**Gene Symbol**	**Name Gene**
ACTA1	actin, alpha 1, skeletal muscle
ACVR1	activin A receptor type 1
ACVR2A	activin A receptor type 2A
ACVRL1	activin A receptor like type 1
AES	amino-terminal enhancer of split
ALK	anaplastic lymphoma receptor tyrosine kinase
ALOX12B	arachidonate 12-lipoxygenase, 12R type
ANG	angiogenin
APBA1	amyloid beta precursor protein binding family A member 1
APBA2	amyloid beta precursor protein binding family A member 2
APLP1	amyloid beta precursor like protein 1
RHOB	ras homolog family member B
ATP2A2	ATPase sarcoplasmic/endoplasmic reticulum Ca2+ transporting 2
ATP2B2	ATPase plasma membrane Ca2+ transporting 2
ATP6V1B1	ATPase H+ transporting V1 subunit B1
BDNF	brain-derived neurotrophic factor
BMPR1B	bone morphogenetic protein receptor type 1B
FOXL2	forkhead box L2
BRAF	B-Raf proto-oncogene, serine/threonine kinase
BTG1	B-cell translocation gene 1, anti-proliferative
MPPED2	metallophosphoesterase domain containing 2
CACNB2	calcium voltage-gated channel auxiliary subunit beta 2
CASQ2	calsequestrin 2
CASR	calcium sensing receptor
RUNX2	runt related transcription factor 2
RUNX1	runt related transcription factor 1
CD3D	CD3d molecule
CDH13	cadherin 13
CENPF	centromere protein F
CHRM1	cholinergic receptor muscarinic 1
CHRM3	cholinergic receptor muscarinic 3
CHRNA1	cholinergic receptor nicotinic alpha 1 subunit
CHRNA4	cholinergic receptor nicotinic alpha 4 subunit
CLN5	ceroid-lipofuscinosis, neuronal 5
CMKLR1	chemerin chemokine-like receptor 1
COL1A1	collagen type I alpha 1
COL1A2	collagen type I alpha 2
COL4A2	collagen type IV alpha 2
COL4A3	collagen type IV alpha 3
COL5A2	collagen type V alpha 2
COL6A3	collagen type VI alpha 3
COL7A1	collagen type VII alpha 1
COL9A1	collagen type IX alpha 1
COL9A2	collagen type IX alpha 2
COL11A1	collagen type XI alpha 1
COL13A1	collagen type XIII alpha 1
CRMP1	collapsin response mediator protein 1
CRX	cone-rod homeobox
CTF1	cardiotrophin 1
CTGF	connective tissue growth factor
DHCR24	24-dehydrocholesterol reductase

DLG4	discs large homolog 4
DLX2	distal-less homeobox 2
DLX5	distal-less homeobox 5
DPYSL3	dihydropyrimidinase like 3
DRD2	dopamine receptor D2
DSCAM	DS cell adhesion molecule
DSP	desmoplakin
DTX1	deltex 1
DVL2	dishevelled segment polarity protein 2
DVL3	dishevelled segment polarity protein 3
EFNA5	ephrin A5
EGR2	early growth response 2
EGR3	early growth response 3
ELN	elastin
EN1	engrailed homeobox 1
EPHB1	EPH receptor B1
EPHB2	EPH receptor B2
EPHB4	EPH receptor B4
STX2	syntaxin 2
CLN8	ceroid-lipofuscinosis, neuronal 8
ERCC2	excision repair cross-complementation group 2
ETS1	ETS proto-oncogene 1, transcription factor
EVC	EvC ciliary complex subunit 1
EVPL	envoplakin
EXT1	exostosin glycosyltransferase 1
EXT2	exostosin glycosyltransferase 2
EXTL1	exostosin-like glycosyltransferase 1
FBN1	fibrillin 1
FGF5	fibroblast growth factor 5
FGF12	fibroblast growth factor 12
FGFR1	fibroblast growth factor receptor 1
FGFR3	fibroblast growth factor receptor 3
FHL3	four and a half LIM domains 3
FOXG1	forkhead box G1
FOXC1	forkhead box C1
FOXO3	forkhead box O3
FLI1	Fli-1 proto-oncogene, ETS transcription factor
FSHR	follicle stimulating hormone receptor
GAA	glucosidase alpha, acid
GATA4	GATA binding protein 4
GDNF	glial cell derived neurotrophic factor
GHSR	growth hormone secretagogue receptor
GLI2	GLI family zinc finger 2
GSTM3	glutathione S-transferase mu 3 (brain)
GYPC	glycophorin C (Gerbich blood group)
HOXA13	homeobox A13
HOXD13	homeobox D13
HES1	hes family bHLH transcription factor 1
HSD17B3	hydroxysteroid (17-beta) dehydrogenase 3
IFI16	interferon gamma inducible protein 16
IFRD1	interferon related developmental regulator 1
IGFBP3	insulin like growth factor binding protein 3
IL10	interleukin 10
IL18	interleukin 18
INHBA	inhibin beta A
PDX1	pancreatic and duodenal homeobox 1
ITGA2	integrin subunit alpha 2
JAG2	jagged 2

JAK2	Janus kinase 2
JARID2	jumonji and AT-rich interaction domain containing 2
KCNQ2	potassium voltage-gated channel subfamily Q member 2
KRT6A	keratin 6A
KRT34	keratin 34
KRT85	keratin 85
LAMA2	laminin subunit alpha 2
LAMC1	laminin subunit gamma 1
LHX1	LIM homeobox 1
LIG1	DNA ligase 1
ABLIM1	actin binding LIM protein 1
LSAMP	limbic system-associated membrane protein
LY6H	lymphocyte antigen 6 complex, locus H
LYN	LYN proto-oncogene, Src family tyrosine kinase
MAL	mal, T-cell differentiation protein
MAPT	microtubule associated protein tau
MBP	myelin basic protein
MEA1	male-enhanced antigen 1
MEF2A	myocyte enhancer factor 2A
MEF2C	myocyte enhancer factor 2C
MEF2D	myocyte enhancer factor 2D
MEST	mesoderm specific transcript
MOBP	myelin-associated oligodendrocyte basic protein
MOG	myelin oligodendrocyte glycoprotein
MSI1	musashi RNA binding protein 1
MSX1	msh homeobox 1
MSX2	msh homeobox 2
MYBPC3	myosin binding protein C, cardiac
MYH7	myosin, heavy chain 7, cardiac muscle, beta
MYH11	myosin, heavy chain 11, smooth muscle
MYL6	myosin light chain 6
NAB2	NGFI-A binding protein 2
NAIP	NLR family, apoptosis inhibitory protein
NCL	nucleolin
NELL1	neural EGFL like 1
NEUROG1	neurogenin 1
NHLH1	nescient helix-loop-helix 1
NINJ1	ninjurin 1
NKX2-2	NK2 homeobox 2
NKX6-1	NK6 homeobox 1
NOTCH1	notch 1
NOTCH2	notch 2
NOTCH4	notch 4
NPAS2	neuronal PAS domain protein 2
NPPB	natriuretic peptide B
NPR1	natriuretic peptide receptor 1
NPR3	natriuretic peptide receptor 3
NPTX1	neuronal pentraxin 1
NRCAM	neuronal cell adhesion molecule
NRTN	neurturin
OTX2	orthodenticle homeobox 2
PAFAH1B3	platelet activating factor acetylhydrolase 1b catalytic subunit 3
PRDX1	peroxiredoxin 1
PARK2	parkin RBR E3 ubiquitin protein ligase
PAX1	paired box 1
PAX2	paired box 2
PAX3	paired box 3
PAX6	paired box 6

PBX1	PBX homeobox 1
PCDH1	protocadherin 1
PCP4	Purkinje cell protein 4
SERPINF1	serpin family F member 1
PF4	platelet factor 4
PITX2	paired like homeodomain 2
PKD2	polycystin 2, transient receptor potential cation channel
PLG	plasminogen
PML	promyelocytic leukemia
POU3F3	POU class 3 homeobox 3
POU6F1	POU class 6 homeobox 1
PPARD	peroxisome proliferator activated receptor delta
KLK7	kallikrein related peptidase 7
PTCH1	patched 1
PTH	parathyroid hormone
PTH1R	parathyroid hormone 1 receptor
PTN	pleiotrophin
PTS	6-pyruvoyltetrahydropterin synthase
RET	ret proto-oncogene
RNH1	ribonuclease/angiogenin inhibitor 1
ROBO1	roundabout guidance receptor 1
ROBO2	roundabout guidance receptor 2
RORB	RAR related orphan receptor B
RTN1	reticulon 1
SCN8A	sodium voltage-gated channel alpha subunit 8
CCL2	C-C motif chemokine ligand 2
SECTM1	secreted and transmembrane 1
SFTPD	surfactant protein D
SGCA	sarcoglycan alpha
SGCD	sarcoglycan delta
SHH	sonic hedgehog
SHOX2	short stature homeobox 2
SIM1	single-minded family bHLH transcription factor 1
SIM2	single-minded family bHLH transcription factor 2
SIX1	SIX homeobox 1
SMTN	smoothelin
SLIT3	slit guidance ligand 3
SNAI2	snail family transcriptional repressor 2
SNCA	synuclein alpha
SOD1	superoxide dismutase 1, soluble
SOX11	SRY-box 11
SPARC	secreted protein acidic and cysteine rich
SPG7	SPG7, paraplegin matrix AAA peptidase subunit
SRD5A2	steroid 5 alpha-reductase 2
STAT3	signal transducer and activator of transcription 3
STATH	statherin
SVIL	supervillin
TBX1	T-box 1
TBX5	T-box 5
TBX3	T-box 3
TCF12	transcription factor 12
TEAD4	TEA domain transcription factor 4
TFAP2A	transcription factor AP-2 alpha
TGM3	transglutaminase 3
KLF10	Kruppel-like factor 10
TIE1	tyrosine kinase with immunoglobulin like and EGF like domains 1
TLE1	transducin like enhancer of split 1
TLE3	transducin like enhancer of split 3

TLL1	tolloid like 1
NR2E1	nuclear receptor subfamily 2 group E member 1
TNNI3	troponin I3, cardiac type
TPD52	tumor protein D52
NR2C2	nuclear receptor subfamily 2 group C member 2
TRPS1	transcriptional repressor GATA binding 1
TWIST1	twist family bHLH transcription factor 1
UBB	ubiquitin B
UBE3A	ubiquitin protein ligase E3A
UGT8	UDP glycosyltransferase 8
UTRN	utrophin
WFS1	wolframin ER transmembrane glycoprotein
YWHAG	tyrosine 3-monooxygenase/tryptophan 5-monooxygenase activation protein gamma
YWHAH	tyrosine 3-monooxygenase/tryptophan 5-monooxygenase activation protein eta
ZAP70	zeta chain of T cell receptor associated protein kinase 70kDa
ZIC1	Zic family member 1
ZIC2	Zic family member 2
ZBTB16	zinc finger and BTB domain containing 16
SCG2	secretogranin II
SEMA3B	semaphorin 3B
ADAM12	ADAM metallopeptidase domain 12
ALX1	ALX homeobox 1
ST8SIA2	ST8 alpha-N-acetyl-neuraminide alpha-2,8-sialyltransferase 2
FZD9	frizzled class receptor 9
SOX14	SRY-box 14
FOXN1	forkhead box N1
ENC1	ectodermal-neural cortex 1
LMO4	LIM domain only 4
UNC5C	unc-5 netrin receptor C
JRKL	JRK-like
SCEL	sciellin
NRP2	neuropilin 2
NRP1	neuropilin 1
LDB1	LIM domain binding 1
SPHK1	sphingosine kinase 1
EIF2B3	eukaryotic translation initiation factor 2B subunit gamma
SGCE	sarcoglycan epsilon
CACNA1H	calcium voltage-gated channel subunit alpha1 H
KALRN	kalirin, RhoGEF kinase
BRSK2	BR serine/threonine kinase 2
SEMA5A	semaphorin 5A
ARTN	artemin
SART1	squamous cell carcinoma antigen recognized by T-cells 1
DCLK1	doublecortin like kinase 1
LGI1	leucine-rich, glioma inactivated 1
NOG	noggin
NUMBL	NUMB like, endocytic adaptor protein
KLF4	Kruppel-like factor 4 (gut)
KL	klotho
NRXN3	neurexin 3
NRXN1	neurexin 1
EIF2AK3	eukaryotic translation initiation factor 2 alpha kinase 3
TBX4	T-box 4
PITPNM1	phosphatidylinositol transfer protein membrane associated 1
CARTPT	CART prepropeptide
CELSR1	cadherin EGF LAG seven-pass G-type receptor 1

FEZ2	fasciculation and elongation protein zeta 2
SOCS5	suppressor of cytokine signaling 5
ECE2	endothelin converting enzyme 2
HDAC9	histone deacetylase 9
HDAC4	histone deacetylase 4
FARP2	FERM, ARH/RhoGEF and pleckstrin domain protein 2
HDAC5	histone deacetylase 5
CLEC3A	C-type lectin domain family 3 member A
GDF11	growth differentiation factor 11
SPRY2	sprouty RTK signaling antagonist 2
SPEG	SPEG complex locus
IKZF1	IKAROS family zinc finger 1
SPON2	spondin 2
OLFM1	olfactomedin 1
BAIAP2	BAI1 associated protein 2
FST	follistatin
SEMA4F	ssemaphorin 4F
DPYSL4	dihydropyrimidinase like 4
PDPN	podoplanin
POSTN	periostin
DMRT2	doublesex and mab-3 related transcription factor 2
DLL3	delta like canonical Notch ligand 3
NCKAP1	NCK associated protein 1
CYP46A1	cytochrome P450 family 46 subfamily A member 1
CIT	citron rho-interacting serine/threonine kinase
GPR45	G protein-coupled receptor 45
IKZF3	IKAROS family zinc finger 3
MRAS	muscle RAS oncogene homolog
NTNG1	netrin G1
NLGN1	neuroligin 1
ACIN1	apoptotic chromatin condensation inducer 1
MYO16	myosin XVI
CYFIP1	cytoplasmic FMR1 interacting protein 1
CBY1	chibby homolog 1 (Drosophila)
VAX2	ventral anterior homeobox 2
ATXN10	ataxin 10
KLK5	kallikrein related peptidase 5
POU2F3	POU class 2 homeobox 3
WHRN	whirlin
PCDHB5	protocadherin beta 5
LHX6	LIM homeobox 6
NPTN	neuroplastin
TINAG	tubulointerstitial nephritis antigen
RND1	Rho family GTPase 1
MYLIP	myosin regulatory light chain interacting protein
SOX8	SRY-box 8
COL5A3	collagen type V alpha 3
NEUROG3	neurogenin 3
MYEF2	myelin expression factor 2
IRX4	iroquois homeobox 4
STMN3	stathmin 3
ZBTB7B	zinc finger and BTB domain containing 7B
EGFL7	EGF like domain multiple 7
CRIM1	cysteine rich transmembrane BMP regulator 1 (chordin-like)
CALML5	calmodulin like 5
SNTG2	syntrophin gamma 2
PCDH18	protocadherin 18
ROBO4	roundabout guidance receptor 4

SNRK	SNF related kinase
AGGF1	angiogenic factor with G-patch and FHA domains 1
MAP1S	microtubule associated protein 1S
MAML3	mastermind like transcriptional coactivator 3
ADAP2	ArfGAP with dual PH domains 2
PCDHB15	protocadherin beta 15
PCDHB13	protocadherin beta 13
PCDHB2	protocadherin beta 2
PCDHA1	protocadherin alpha 1
ANKH	ANKH inorganic pyrophosphate transport regulator
KIAA1217	KIAA1217
PARD3	par-3 family cell polarity regulator
ANKRD7	ankyrin repeat domain 7
C1GALT1	core 1 synthase, glycoprotein-N-acetylgalactosamine 3-beta-galactosyltransferase 1
NMUR2	neuromedin U receptor 2
MEPE	matrix extracellular phosphoglycoprotein
UTP3	UTP3, small subunit processome component homolog (S. cerevisiae)
RTN4	reticulon 4
SMURF1	SMAD specific E3 ubiquitin protein ligase 1
DSCAML1	DS cell adhesion molecule like 1
MKL2	MKL1/myocardin like 2
LRRC4C	leucine rich repeat containing 4C
PROK2	prokineticin 2
COL18A1	collagen type XVIII alpha 1
CCM2	CCM2 scaffolding protein
KIRREL3	kin of IRRE like 3 (Drosophila)
NTNG2	netrin G2
SCIN	scinderin
TRIM15	tripartite motif containing 15
ATPIF1	ATPase inhibitory factor 1
FOXP2	forkhead box P2
APOA5	apolipoprotein A5
TWIST2	twist family bHLH transcription factor 2
GYLTL1B	glycosyltransferase-like 1B
DMBX1	diencephalon/mesencephalon homeobox 1
NRSN1	neurensin 1
RTN4RL1	reticulon 4 receptor like 1
NFAM1	NFAT activating protein with ITAM motif 1
CNTN4	contactin 4
ZNF384	zinc finger protein 384
EPGN	epithelial mitogen
PCSK9	proprotein convertase subtilisin/kexin type 9
PTF1A	pancreas specific transcription factor, 1a
MDGA1	MAM domain containing glycosylphosphatidylinositol anchor 1
VWC2	von Willebrand factor C domain containing 2
AGRN	agrin
**Nervous system development**	
**Gene Symbol**	**Name Gene**
ALK	anaplastic lymphoma receptor tyrosine kinase
APBA1	amyloid beta precursor protein binding family A member 1
APBA2	amyloid beta precursor protein binding family A member 2
APLP1	amyloid beta precursor like protein 1
ATP2B2	ATPase plasma membrane Ca2+ transporting 2
BDNF	brain-derived neurotrophic factor
CHRM3	cholinergic receptor muscarinic 3
CHRNA4	cholinergic receptor nicotinic alpha 4 subunit
CLN5	ceroid-lipofuscinosis, neuronal 5

CRMP1	collapsin response mediator protein 1
CTF1	cardiotrophin 1
DLG4	discs large homolog 4
DLX2	distal-less homeobox 2
DLX5	distal-less homeobox 5
DRD2	dopamine receptor D2
DSCAM	DS cell adhesion molecule
DTX1	deltex 1
DVL3	dishevelled segment polarity protein 3
EFNA5	ephrin A5
EPHB1	EPH receptor B1
EPHB2	EPH receptor B2
FGF12	fibroblast growth factor 12
FOXG1	forkhead box G1
GDNF	glial cell derived neurotrophic factor
GLI2	GLI family zinc finger 2
HES1	hes family bHLH transcription factor 1
JARID2	jumonji and AT-rich interaction domain containing 2
KCNQ2	potassium voltage-gated channel subfamily Q member 2
LHX1	LIM homeobox 1
LY6H	lymphocyte antigen 6 complex, locus H
MAL	mal, T-cell differentiation protein
MAPT	microtubule associated protein tau
MBP	myelin basic protein
MEF2C	myocyte enhancer factor 2C
MOBP	myelin-associated oligodendrocyte basic protein
MOG	myelin oligodendrocyte glycoprotein
MSI1	musashi RNA binding protein 1
NAB2	NGFI-A binding protein 2
NAIP	NLR family, apoptosis inhibitory protein
NELL1	neural EGFL like 1
NEUROG1	neurogenin 1
NHLH1	nescient helix-loop-helix 1
NINJ1	ninjurin 1
NPAS2	neuronal PAS domain protein 2
NPTX1	neuronal pentraxin 1
NRCAM	neuronal cell adhesion molecule
NRTN	neurturin
PAFAH1B3	platelet activating factor acetylhydrolase 1b catalytic subunit 3
PARK2	parkin RBR E3 ubiquitin protein ligase
PAX2	paired box 2
PAX6	paired box 6
PBX1	PBX homeobox 1
PCDH1	protocadherin 1
SERPINF1	serpin family F member 1
POU3F3	POU class 3 homeobox 3
POU6F1	POU class 6 homeobox 1
PPARD	peroxisome proliferator activated receptor delta
PTN	pleiotrophin
PTS	6-pyruvoyltetrahydropterin synthase
ROBO1	roundabout guidance receptor 1
ROBO2	roundabout guidance receptor 2
RTN1	reticulon 1
SCN8A	sodium voltage-gated channel alpha subunit 8
SHH	sonic hedgehog
SIM1	single-minded family bHLH transcription factor 1
SIM2	single-minded family bHLH transcription factor 2
SLIT3	slit guidance ligand 3

SNCA	synuclein alpha
SOD1	superoxide dismutase 1, soluble
SOX11	SRY-box 11
SPG7	SPG7, paraplegin matrix AAA peptidase subunit
STAT3	signal transducer and activator of transcription 3
NR2E1	nuclear receptor subfamily 2 group E member 1
UBB	ubiquitin B
UBE3A	ubiquitin protein ligase E3A
UGT8	UDP glycosyltransferase 8
WFS1	wolframin ER transmembrane glycoprotein
YWHAG	tyrosine 3-monooxygenase/tryptophan 5-monooxygenase activation protein gamma
ZIC2	Zic family member 2
ZBTB16	zinc finger and BTB domain containing 16
SEMA3B	semaphorin 3B
ALX1	ALX homeobox 1
ST8SIA2	ST8 alpha-N-acetyl-neuraminide alpha-2,8-sialyltransferase 2
SOX14	SRY-box 14
ENC1	ectodermal-neural cortex 1
UNC5C	unc-5 netrin receptor C
NRP2	neuropilin 2
NRP1	neuropilin 1
LDB1	LIM domain binding 1
EIF2B3	eukaryotic translation initiation factor 2B subunit gamma
KALRN	kalirin, RhoGEF kinase
BRSK2	BR serine/threonine kinase 2
ARTN	artemin
LGI1	leucine-rich, glioma inactivated 1
NOG	noggin
NUMBL	NUMB like, endocytic adaptor protein
NRXN3	neurexin 3
NRXN1	neurexin 1
PITPNM1	phosphatidylinositol transfer protein membrane associated 1
CELSR1	cadherin EGF LAG seven-pass G-type receptor 1
FEZ2	fasciculation and elongation protein zeta 2
HDAC4	histone deacetylase 4
FARP2	FERM, ARH/RhoGEF and pleckstrin domain protein 2
SPON2	spondin 2
OLFM1	olfactomedin 1
BAIAP2	BAI1 associated protein 2
SEMA4F	ssemaphorin 4F
CYP46A1	cytochrome P450 family 46 subfamily A member 1
CIT	citron rho-interacting serine/threonine kinase
GPR45	G protein-coupled receptor 45
NTNG1	netrin G1
NLGN1	neuroligin 1
MYO16	myosin XVI
CYFIP1	cytoplasmic FMR1 interacting protein 1
ATXN10	ataxin 10
PCDHB5	protocadherin beta 5
LHX6	LIM homeobox 6
NPTN	neuroplastin
RND1	Rho family GTPase 1
MYLIP	myosin regulatory light chain interacting protein
SOX8	SRY-box 8
STMN3	stathmin 3
CRIM1	cysteine rich transmembrane BMP regulator 1 (chordin-like)
SNTG2	syntrophin gamma 2

PCDH18	protocadherin 18
MAP1S	microtubule associated protein 1S
PCDHB13	protocadherin beta 13
PCDHA1	protocadherin alpha 1
PARD3	par-3 family cell polarity regulator
NMUR2	neuromedin U receptor 2
RTN4	reticulon 4
DSCAML1	DS cell adhesion molecule like 1
LRRC4C	leucine rich repeat containing 4C
NTNG2	netrin G2
FOXP2	forkhead box P2
DMBX1	diencephalon/mesencephalon homeobox 1
NRSN1	neurensin 1
RTN4RL1	reticulon 4 receptor like 1
CNTN4	contactin 4
ZNF384	zinc finger protein 384
MDGA1	MAM domain containing glycosylphosphatidylinositol anchor 1
AGRN	agrin
**Signal transduction**	
**Gene Symbol**	**Name Gene**
ABCA1	ATP binding cassette subfamily A member 1
ABR	active BCR-related
ACTL6A	actin like 6A
ACVR1	activin A receptor type 1
ACVR2A	activin A receptor type 2A
ACVRL1	activin A receptor like type 1
ADM	adrenomedullin
ADRA1A	adrenoceptor alpha 1A
ADRA2A	adrenoceptor alpha 2A
ADRA2B	adrenoceptor alpha 2B
ADRA2C	adrenoceptor alpha 2C
ADRB3	adrenoceptor beta 3
GRK3	G protein-coupled receptor kinase 3
ALCAM	activated leukocyte cell adhesion molecule
ANG	angiogenin
ANGPT1	angiopoietin 1
BIRC2	baculoviral IAP repeat containing 2
FAS	Fas cell surface death receptor
FASLG	Fas ligand
RHOB	ras homolog family member B
ARHGAP1	Rho GTPase activating protein 1
ARHGDIA	Rho GDP dissociation inhibitor alpha
ASGR2	asialoglycoprotein receptor 2
AVPR1A	arginine vasopressin receptor 1A
AXL	AXL receptor tyrosine kinase
BCL3	B-cell CLL/lymphoma 3
BDKRB2	bradykinin receptor B2
BIK	BCL2 interacting killer
BMPR1A	bone morphogenetic protein receptor type 1A
BMPR2	bone morphogenetic protein receptor type 2
BNIP3	BCL2/adenovirus E1B 19kDa interacting protein 3
FOXL2	forkhead box L2
BRCA1	breast cancer 1
BSG	basigin (Ok blood group)
BST2	bone marrow stromal cell antigen 2
C3	complement component 3
C3AR1	complement component 3a receptor 1
CAPN5	calpain 5

CALCB	calcitonin related polypeptide beta
CALCR	calcitonin receptor
CAMK2B	calcium/calmodulin dependent protein kinase II beta
CASP3	caspase 3
CASP9	caspase 9
CASR	calcium sensing receptor
CBL	Cbl proto-oncogene
CCNA2	cyclin A2
CD2	CD2 molecule
CD3E	CD3e molecule
CD3G	CD3g molecule
CD28	CD28 molecule
TNFRSF8	tumor necrosis factor receptor superfamily member 8
CD40	CD40 molecule
CD59	CD59 molecule
CD70	CD70 molecule
CD74	CD74 molecule
CD81	CD81 molecule
CDH13	cadherin 13
CDS1	CDP-diacylglycerol synthase 1
CGA	glycoprotein hormones, alpha polypeptide
CHEK1	checkpoint kinase 1
FOXN3	forkhead box N3
CHRM1	cholinergic receptor muscarinic 1
LYST	lysosomal trafficking regulator
CHRM3	cholinergic receptor muscarinic 3
CHRM4	cholinergic receptor muscarinic 4
CHRNA1	cholinergic receptor nicotinic alpha 1 subunit
CHRNA2	cholinergic receptor nicotinic alpha 2 subunit
CHRNA3	cholinergic receptor nicotinic alpha 3 subunit
CHRNA4	cholinergic receptor nicotinic alpha 4 subunit
CHRNB1	cholinergic receptor nicotinic beta 1 subunit
CHRNB2	cholinergic receptor nicotinic beta 2 subunit
CHRNB3	cholinergic receptor nicotinic beta 3 subunit
CHUK	conserved helix-loop-helix ubiquitous kinase
CIDEA	cell death-inducing DFFA-like effector a
CLIC1	chloride intracellular channel 1
CCR3	C-C motif chemokine receptor 3
CCR6	C-C motif chemokine receptor 6
LTB4R	leukotriene B4 receptor
CNGA3	cyclic nucleotide gated channel alpha 3
CNR1	cannabinoid receptor 1 (brain)
CNR2	cannabinoid receptor 2
COL4A3	collagen type IV alpha 3
CREB1	cAMP responsive element binding protein 1
ATF6B	activating transcription factor 6 beta
CREM	cAMP responsive element modulator
CRHBP	corticotropin releasing hormone binding protein
CRHR1	corticotropin releasing hormone receptor 1
CRHR2	corticotropin releasing hormone receptor 2
CSNK1D	casein kinase 1 delta
CSNK1G2	casein kinase 1 gamma 2
CSNK1G3	casein kinase 1 gamma 3
CSNK2A1	casein kinase 2 alpha 1
CTNND2	catenin delta 2
CX3CR1	C-X3-C motif chemokine receptor 1
DGKA	diacylglycerol kinase alpha
DAPK1	death associated protein kinase 1

DAPK3	death-associated protein kinase 3
DAXX	death-domain associated protein
DFFB	DNA fragmentation factor subunit beta
DLG4	discs large homolog 4
DMPK	dystrophia myotonica protein kinase
DOCK1	dedicator of cytokinesis 1
DPYSL3	dihydropyrimidinase like 3
DRD2	dopamine receptor D2
DRG2	developmentally regulated GTP binding protein 2
DTX1	deltex 1
DUSP4	dual specificity phosphatase 4
ECT2	epithelial cell transforming 2
S1PR3	sphingosine-1-phosphate receptor 3
EDN1	endothelin 1
EDN2	endothelin 2
EDNRB	endothelin receptor type B
EEF1D	eukaryotic translation elongation factor 1 delta
EGFR	epidermal growth factor receptor
EPHA2	EPH receptor A2
ELK3	ELK3, ETS transcription factor
MARK2	microtubule affinity regulating kinase 2
EPAS1	endothelial PAS domain protein 1
EPHA3	EPH receptor A3
EPHB1	EPH receptor B1
EPHB3	EPH receptor B3
STX2	syntaxin 2
EPS8	epidermal growth factor receptor pathway substrate 8
EPS15	epidermal growth factor receptor pathway substrate 15
ERBB3	erb-b2 receptor tyrosine kinase 3
ERBB4	erb-b2 receptor tyrosine kinase 4
ERN1	endoplasmic reticulum to nucleus signaling 1
ESR1	estrogen receptor 1
EXT1	exostosin glycosyltransferase 1
EXT2	exostosin glycosyltransferase 2
F2	coagulation factor II, thrombin
PTK2B	protein tyrosine kinase 2 beta
FGF3	fibroblast growth factor 3
FGF4	fibroblast growth factor 4
FGF5	fibroblast growth factor 5
FGF6	fibroblast growth factor 6
FGF9	fibroblast growth factor 9
FGF12	fibroblast growth factor 12
FGF14	fibroblast growth factor 14
FGFR1	fibroblast growth factor receptor 1
FGFR3	fibroblast growth factor receptor 3
FGFR4	fibroblast growth factor receptor 4
FLNB	filamin B
FLT3	fms related tyrosine kinase 3
FLT4	fms related tyrosine kinase 4
FMOD	fibromodulin
FPR1	formyl peptide receptor 1
FSHR	follicle stimulating hormone receptor
FYB	FYN binding protein
FYN	FYN proto-oncogene, Src family tyrosine kinase
IFI6	interferon alpha inducible protein 6
GAB1	GRB2 associated binding protein 1
GABBR1	gamma-aminobutyric acid type B receptor subunit 1
GABRA1	gamma-aminobutyric acid type A receptor alpha1 subunit

GABRA4	gamma-aminobutyric acid type A receptor alpha4 subunit
GABRB1	gamma-aminobutyric acid type A receptor beta1 subunit
GABRB2	gamma-aminobutyric acid type A receptor beta2 subunit
GABRB3	gamma-aminobutyric acid type A receptor beta3 subunit
GABRD	gamma-aminobutyric acid type A receptor delta subunit
GABRR2	gamma-aminobutyric acid type A receptor rho2 subunit
GALR1	galanin receptor 1
GAP43	growth associated protein 43
GDF9	growth differentiation factor 9
GDNF	glial cell derived neurotrophic factor
GEM	GTP binding protein overexpressed in skeletal muscle
GFRA2	GDNF family receptor alpha 2
GHRHR	growth hormone releasing hormone receptor
GHSR	growth hormone secretagogue receptor
GIPR	gastric inhibitory polypeptide receptor
GJA1	gap junction protein alpha 1
GLI2	GLI family zinc finger 2
GLI3	GLI family zinc finger 3
GLP1R	glucagon like peptide 1 receptor
GLRA1	glycine receptor alpha 1
GML	glycosylphosphatidylinositol anchored molecule like
GNA11	G protein subunit alpha 11
GNA12	G protein subunit alpha 12
GNAI2	G protein subunit alpha i2
GNAL	G protein subunit alpha L
GNAS	GNAS complex locus
GNB2	G protein subunit beta 2
GNB3	G protein subunit beta 3
GNG4	G protein subunit gamma 4
GNG7	G protein subunit gamma 7
GNL1	G protein nucleolar 1 (putative)
GPR3	G protein-coupled receptor 3
XCR1	X-C motif chemokine receptor 1
GPR6	G protein-coupled receptor 6
NPBWR1	neuropeptides B/W receptor 1
NPBWR2	neuropeptides B/W receptor 2
UTS2R	urotensin 2 receptor
GPR19	G protein-coupled receptor 19
GPR20	G protein-coupled receptor 20
GPR25	G protein-coupled receptor 25
GPR27	G protein-coupled receptor 27
GPR35	G protein-coupled receptor 35
GPR39	G protein-coupled receptor 39
FFAR1	free fatty acid receptor 1
FFAR2	free fatty acid receptor 2
GRK5	G protein-coupled receptor kinase 5
GRK6	G protein-coupled receptor kinase 6
MKNK2	MAP kinase interacting serine/threonine kinase 2
GPS1	G protein pathway suppressor 1
GRB2	growth factor receptor bound protein 2
GRB7	growth factor receptor bound protein 7
GRB10	growth factor receptor bound protein 10
GRB14	growth factor receptor bound protein 14
RAPGEF1	Rap guanine nucleotide exchange factor 1
GRIA1	glutamate ionotropic receptor AMPA type subunit 1
GRIA2	glutamate ionotropic receptor AMPA type subunit 2
GRIA4	glutamate ionotropic receptor AMPA type subunit 4
GRID2	glutamate ionotropic receptor delta type subunit 2

GRIK2	glutamate ionotropic receptor kainate type subunit 2
GRIK4	glutamate ionotropic receptor kainate type subunit 4
GRIN2A	glutamate ionotropic receptor NMDA type subunit 2A
GRIN2B	glutamate ionotropic receptor NMDA type subunit 2B
GRIN2C	glutamate ionotropic receptor NMDA type subunit 2C
NR3C1	nuclear receptor subfamily 3 group C member 1
GRM2	glutamate metabotropic receptor 2
GRM3	glutamate metabotropic receptor 3
GRM4	glutamate metabotropic receptor 4
GRM5	glutamate metabotropic receptor 5
GRM6	glutamate metabotropic receptor 6
GRM7	glutamate metabotropic receptor 7
GRM8	glutamate metabotropic receptor 8
GRP	gastrin releasing peptide
GTF2I	general transcription factor IIi
GUCY2D	guanylate cyclase 2D, retinal
HCRTR1	hypocretin receptor 1
HCRTR2	hypocretin receptor 2
HIP1	huntingtin interacting protein 1
HINT1	histidine triad nucleotide binding protein 1
HMGB1	high mobility group box 1
NR4A1	nuclear receptor subfamily 4 group A member 1
HRH1	histamine receptor H1
PRMT2	protein arginine methyltransferase 2
HTR1A	5-hydroxytryptamine receptor 1A
HTR1B	5-hydroxytryptamine receptor 1B
HTR1E	5-hydroxytryptamine receptor 1E
HTR1F	5-hydroxytryptamine receptor 1F
HTR2A	5-hydroxytryptamine receptor 2A
HTR5A	5-hydroxytryptamine receptor 5A
HTR6	5-hydroxytryptamine receptor 6
IFI16	interferon gamma inducible protein 16
IFNAR2	interferon alpha and beta receptor subunit 2
IFNGR1	interferon gamma receptor 1
IFNGR2	interferon gamma receptor 2 (interferon gamma transducer 1)
IGF1R	insulin like growth factor 1 receptor
IGF2R	insulin like growth factor 2 receptor
IGFALS	insulin like growth factor binding protein acid labile subunit
IL1B	interleukin 1 beta
IL2RA	interleukin 2 receptor subunit alpha
IL6	interleukin 6
IL6R	interleukin 6 receptor
IL12A	interleukin 12A
IL12RB1	interleukin 12 receptor subunit beta 1
IL15	interleukin 15
IL15RA	interleukin 15 receptor subunit alpha
ING2	inhibitor of growth family member 2
INHBA	inhibin beta A
INPP5D	inositol polyphosphate-5-phosphatase D
INSR	insulin receptor
ITPK1	inositol-tetrakisphosphate 1-kinase
ITPKA	inositol-trisphosphate 3-kinase A
ITPR3	inositol 1,4,5-trisphosphate receptor type 3
JAK2	Janus kinase 2
KIT	KIT proto-oncogene receptor tyrosine kinase
LALBA	lactalbumin alpha
STMN1	stathmin 1
LEPR	leptin receptor

LETM1	leucine zipper and EF-hand containing transmembrane protein 1
LGALS1	galectin 1
LGALS9	galectin 9
LIFR	leukemia inhibitory factor receptor alpha
LRP6	LDL receptor related protein 6
LTA	lymphotoxin alpha
LTB	lymphotoxin beta
LTBP2	latent transforming growth factor beta binding protein 2
BCAM	basal cell adhesion molecule (Lutheran blood group)
LY6E	lymphocyte antigen 6 complex, locus E
LYN	LYN proto-oncogene, Src family tyrosine kinase
TACSTD2	tumor-associated calcium signal transducer 2
SMAD3	SMAD family member 3
SMAD7	SMAD family member 7
MAL	mal, T-cell differentiation protein
MC3R	melanocortin 3 receptor
MCC	mutated in colorectal cancers
MCL1	myeloid cell leukemia 1
MDFI	MyoD family inhibitor
MAP3K3	mitogen-activated protein kinase kinase kinase 3
KITLG	KIT ligand
MAP3K9	mitogen-activated protein kinase kinase kinase 9
MAP3K10	mitogen-activated protein kinase kinase kinase 10
MAP3K11	mitogen-activated protein kinase kinase kinase 11
MLLT4	myeloid/lymphoid or mixed-lineage leukemia; translocated to, 4
MPP3	membrane palmitoylated protein 3
MST1R	macrophage stimulating 1 receptor
MTNR1A	melatonin receptor 1A
MTNR1B	melatonin receptor 1B
MX1	MX dynamin like GTPase 1
MYD88	myeloid differentiation primary response 88
GADD45B	growth arrest and DNA damage inducible beta
MYO9B	myosin IXB
PPP1R12B	protein phosphatase 1 regulatory subunit 12B
NEDD9	neural precursor cell expressed, developmentally down-regulated 9
NF2	neurofibromin 2 (merlin)
NFATC1	nuclear factor of activated T-cells 1
NFKBIB	NFKB inhibitor beta
NMB	neuromedin B
NMBR	neuromedin B receptor
NOTCH1	notch 1
NPY	neuropeptide Y
NOTCH2	notch 2
NPM1	nucleophosmin (nucleolar phosphoprotein B23, numatrin)
NPR2	natriuretic peptide receptor 2
NPY5R	neuropeptide Y receptor Y5
NRTN	neurturin
NTF3	neurotrophin 3
NTRK2	neurotrophic receptor tyrosine kinase 2
NTRK3	neurotrophic receptor tyrosine kinase 3
ROR1	receptor tyrosine kinase-like orphan receptor 1
ROR2	receptor tyrosine kinase like orphan receptor 2
NTSR1	neurotensin receptor 1 (high affinity)
OPRD1	opioid receptor delta 1
OPRM1	opioid receptor mu 1
OTX2	orthodenticle homeobox 2
OXT	oxytocin/neurophysin I prepropeptide
P2RX1	purinergic receptor P2X 1

P2RX4	purinergic receptor P2X 4
P2RX7	purinergic receptor P2X 7
PAK1	p21 (RAC1) activated kinase 1
PDE4A	phosphodiesterase 4A
PDE9A	phosphodiesterase 9A
PDGFA	platelet derived growth factor subunit A
PDGFRA	platelet derived growth factor receptor alpha
PDGFRB	platelet derived growth factor receptor beta
PDK1	pyruvate dehydrogenase kinase 1
PDPK1	3-phosphoinositide dependent protein kinase 1
PENK	proenkephalin
PF4	platelet factor 4
PGR	progesterone receptor
SERPINB9	serpin family B member 9
PIK3R1	phosphoinositide-3-kinase regulatory subunit 1
PLA2G1B	phospholipase A2 group IB
PLCB2	phospholipase C beta 2
PLD1	phospholipase D1
PLD2	phospholipase D2
PML	promyelocytic leukemia
PPARG	peroxisome proliferator activated receptor gamma
MED1	mediator complex subunit 1
PPM1A	protein phosphatase, Mg2+/Mn2+ dependent 1A
PPP5C	protein phosphatase 5 catalytic subunit
PRKAA2	protein kinase AMP-activated catalytic subunit alpha 2
PKN1	protein kinase N1
PKN2	protein kinase N2
PRKG1	protein kinase, cGMP-dependent, type I
PRKG2	protein kinase, cGMP-dependent, type II
MAPK11	mitogen-activated protein kinase 11
MAPK10	mitogen-activated protein kinase 10
MAP2K6	mitogen-activated protein kinase kinase 6
THAP12	THAP domain containing 12
PRLR	prolactin receptor
PSEN1	presenilin 1
PSEN2	presenilin 2
PYY	peptide YY
PTAFR	platelet activating factor receptor
PTCH1	patched 1
PTGER3	prostaglandin E receptor 3
PTH	parathyroid hormone
PTH1R	parathyroid hormone 1 receptor
PTK2	protein tyrosine kinase 2
PTK7	protein tyrosine kinase 7 (inactive)
PTN	pleiotrophin
PTPN1	protein tyrosine phosphatase, non-receptor type 1
PTPN11	protein tyrosine phosphatase, non-receptor type 11
PTPRD	protein tyrosine phosphatase, receptor type D
PTPRF	protein tyrosine phosphatase, receptor type F
PTPRG	protein tyrosine phosphatase, receptor type G
PTPRJ	protein tyrosine phosphatase, receptor type J
RAD1	RAD1 checkpoint DNA exonuclease
PWP2	PWP2 periodic tryptophan protein homolog (yeast)
RAD9A	RAD9 checkpoint clamp component A
RAD17	RAD17 checkpoint clamp loader component
RALA	RALA Ras like proto-oncogene A
RALB	RALB Ras like proto-oncogene B
RANBP1	RAN binding protein 1

RANGAP1	Ran GTPase activating protein 1
RAP1GAP	RAP1 GTPase activating protein
RAP2A	RAP2A, member of RAS oncogene family
RARB	retinoic acid receptor beta
RCVRN	recoverin
RET	ret proto-oncogene
RGS1	regulator of G-protein signaling 1
RGS2	regulator of G-protein signaling 2
RGS3	regulator of G-protein signaling 3
RGS12	regulator of G-protein signaling 12
RGS16	regulator of G-protein signaling 16
RHEB	Ras homolog enriched in brain
RHO	rhodopsin
GRK1	G protein-coupled receptor kinase 1
RIT1	Ras like without CAAX 1
ROCK1	Rho associated coiled-coil containing protein kinase 1
ROS1	ROS proto-oncogene 1, receptor tyrosine kinase
RPS6KA2	ribosomal protein S6 kinase A2
RREB1	ras responsive element binding protein 1
RTKN	rhotekin
RSU1	Ras suppressor protein 1
RYK	receptor-like tyrosine kinase
S100A11	S100 calcium binding protein A11
SAG	S-antigen; retina and pineal gland (arrestin)
SCTR	secretin receptor
CCL2	C-C motif chemokine ligand 2
CCL3	C-C motif chemokine ligand 3
CCL19	C-C motif chemokine ligand 19
CCL20	C-C motif chemokine ligand 20
CXCL5	C-X-C motif chemokine ligand 5
CX3CL1	C-X3-C motif chemokine ligand 1
SECTM1	secreted and transmembrane 1
MAP2K4	mitogen-activated protein kinase kinase 4
SFRP1	secreted frizzled related protein 1
SFRP5	secreted frizzled related protein 5
SH3BP2	SH3 domain binding protein 2
SHH	sonic hedgehog
SOD1	superoxide dismutase 1, soluble
SPG7	SPG7, paraplegin matrix AAA peptidase subunit
SRC	SRC proto-oncogene, non-receptor tyrosine kinase
SRP72	signal recognition particle 72kDa
SRPK1	SRSF protein kinase 1
SRPK2	SRSF protein kinase 2
SSTR1	somatostatin receptor 1
SSTR5	somatostatin receptor 5
STAT3	signal transducer and activator of transcription 3
STAT4	signal transducer and activator of transcription 4
STAT6	signal transducer and activator of transcription 6
STC1	stanniocalcin 1
STK3	serine/threonine kinase 3
TACR2	tachykinin receptor 2
TAC3	tachykinin 3
TACR1	tachykinin receptor 1
TACR3	tachykinin receptor 3
MAP3K7	mitogen-activated protein kinase kinase kinase 7
TBXA2R	thromboxane A2 receptor
TCF7L2	transcription factor 7 like 2
NR2F1	nuclear receptor subfamily 2 group F member 1

NR2F2	nuclear receptor subfamily 2 group F member 2
TGFA	transforming growth factor alpha
TGFB1	transforming growth factor beta 1
TGFBR3	transforming growth factor beta receptor 3
KLF10	Kruppel-like factor 10
TIE1	tyrosine kinase with immunoglobulin like and EGF like domains 1
TLE1	transducin like enhancer of split 1
TLE2	transducin like enhancer of split 2
TLE3	transducin like enhancer of split 3
TLR3	toll like receptor 3
TLR4	toll like receptor 4
TNF	tumor necrosis factor
TNFAIP3	TNF alpha induced protein 3
TNFAIP6	TNF alpha induced protein 6
TP53	tumor protein p53
TP73	tumor protein p73
TPTE	transmembrane phosphatase with tensin homology
TRAF3	TNF receptor associated factor 3
TRAF5	TNF receptor associated factor 5
TRIO	trio Rho guanine nucleotide exchange factor
TNFSF4	tumor necrosis factor superfamily member 4
TYRO3	TYRO3 protein tyrosine kinase
UCN	urocortin
VCP	valosin containing protein
VDAC1	voltage dependent anion channel 1
VEGFA	vascular endothelial growth factor A
VEGFC	vascular endothelial growth factor C
VIPR1	vasoactive intestinal peptide receptor 1
VIPR2	vasoactive intestinal peptide receptor 2
TRPV1	transient receptor potential cation channel subfamily V member 1
WNT5A	Wnt family member 5A
YWHAH	tyrosine 3-monooxygenase/tryptophan 5-monooxygenase activation protein eta
YWHAZ	tyrosine 3-monooxygenase/tryptophan 5-monooxygenase activation protein zeta
ZIC1	Zic family member 1
ZYX	zyxin
LRP8	LDL receptor related protein 8
CXCR4	C-X-C motif chemokine receptor 4
SCG2	secretogranin II
NR4A3	nuclear receptor subfamily 4 group A member 3
GPR68	G protein-coupled receptor 68
ANP32A	acidic nuclear phosphoprotein 32 family member A
TRRAP	transformation/transcription domain associated protein
OR1D5	olfactory receptor family 1 subfamily D member 5
PIP5K1A	phosphatidylinositol-4-phosphate 5-kinase type 1 alpha
ULK1	unc-51 like autophagy activating kinase 1
BCAR3	breast cancer anti-estrogen resistance 3
STK24	serine/threonine kinase 24
NSMAF	neutral sphingomyelinase activation associated factor
NCK2	NCK adaptor protein 2
DYRK2	dual specificity tyrosine phosphorylation regulated kinase 2
CDC42BPA	CDC42 binding protein kinase alpha
GALR3	galanin receptor 3
MAP4K3	mitogen-activated protein kinase kinase kinase kinase 3
PPFIA1	PTPRF interacting protein alpha 1
PIK3R3	phosphoinositide-3-kinase regulatory subunit 3
CNTNAP1	contactin associated protein 1

IFITM1	interferon induced transmembrane protein 1
CAMK1	calcium/calmodulin dependent protein kinase I
MADD	MAP kinase activating death domain
MKNK1	MAP kinase interacting serine/threonine kinase 1
RGS20	regulator of G-protein signaling 20
STC2	stanniocalcin 2
TP63	tumor protein p63
IRS2	insulin receptor substrate 2
TRADD	TNFRSF1A associated via death domain
TNFRSF25	tumor necrosis factor receptor superfamily member 25
RIPK1	receptor interacting serine/threonine kinase 1
TNFSF9	tumor necrosis factor superfamily member 9
CD164	CD164 molecule
RIPK2	receptor interacting serine/threonine kinase 2
FADD	Fas associated via death domain
TNFRSF18	tumor necrosis factor receptor superfamily member 18
RGS9	regulator of G-protein signaling 9
TNFRSF11A	tumor necrosis factor receptor superfamily member 11a
TNFRSF10A	tumor necrosis factor receptor superfamily member 10a
PEX11A	peroxisomal biogenesis factor 11 alpha
IL1RL2	interleukin 1 receptor like 2
GALR2	galanin receptor 2
FGF18	fibroblast growth factor 18
NRP1	neuropilin 1
CFLAR	CASP8 and FADD like apoptosis regulator
WISP3	WNT1 inducible signaling pathway protein 3
NR1I2	nuclear receptor subfamily 1 group I member 2
ARHGEF7	Rho guanine nucleotide exchange factor 7
SPHK1	sphingosine kinase 1
SQSTM1	sequestosome 1
CACNA1I	calcium voltage-gated channel subunit alpha1 I
FOXH1	forkhead box H1
SKAP2	src kinase associated phosphoprotein 2
BTRC	beta-transducin repeat containing E3 ubiquitin protein ligase
RPS6KA4	ribosomal protein S6 kinase A4
LIMD1	LIM domains containing 1
KALRN	kalirin, RhoGEF kinase
CDKL2	cyclin dependent kinase like 2
F2RL3	F2R like thrombin/trypsin receptor 3
MPZL1	myelin protein zero like 1
SOCS3	suppressor of cytokine signaling 3
DOK2	docking protein 2
ARTN	artemin
MTA1	metastasis associated 1
SMC3	structural maintenance of chromosomes 3
HGS	hepatocyte growth factor-regulated tyrosine kinase substrate
LPAR2	lysophosphatidic acid receptor 2
MAP3K13	mitogen-activated protein kinase kinase kinase 13
OSMR	oncostatin M receptor
VAPB	VAMP (vesicle-associated membrane protein)-associated protein B and C
VAPA	VAMP associated protein A
MAGI1	membrane associated guanylate kinase, WW and PDZ domain containing 1
MAPKAPK2	mitogen-activated protein kinase-activated protein kinase 2
STK17B	serine/threonine kinase 17b
GPR55	G protein-coupled receptor 55
SOCS6	suppressor of cytokine signaling 6
CD83	CD83 molecule
TRIP10	thyroid hormone receptor interactor 10

TGFBRAP1	transforming growth factor beta receptor associated protein 1
GRAP2	GRB2-related adaptor protein 2
ECEL1	endothelin converting enzyme-like 1
EIF2AK3	eukaryotic translation initiation factor 2 alpha kinase 3
HOMER3	homer scaffolding protein 3
HOMER2	homer scaffolding protein 2
AKAP7	A-kinase anchoring protein 7
SH3BP5	SH3 domain binding protein 5
MAPK8IP1	mitogen-activated protein kinase 8 interacting protein 1
ADAMTS1	ADAM metallopeptidase with thrombospondin type 1 motif 1
LITAF	lipopolysaccharide induced TNF factor
GDF15	growth differentiation factor 15
BCAR1	BCAR1, Cas family scaffolding protein
GTPBP1	GTP binding protein 1
GABBR2	gamma-aminobutyric acid type B receptor subunit 2
BRE	brain and reproductive organ-expressed (TNFRSF1A modulator)
CDC42BPB	CDC42 binding protein kinase beta
CARTPT	CART prepropeptide
RIN1	Ras and Rab interactor 1
GNA14	G protein subunit alpha 14
FEZ2	fasciculation and elongation protein zeta 2
IKBKE	inhibitor of kappa light polypeptide gene enhancer in B-cells, kinase epsilon
SOCS5	suppressor of cytokine signaling 5
CEP57	centrosomal protein 57
MTSS1	metastasis suppressor 1
ARHGEF11	Rho guanine nucleotide exchange factor 11
ELMO1	engulfment and cell motility 1
FARP2	FERM, ARH/RhoGEF and pleckstrin domain protein 2
MED24	mediator complex subunit 24
TLK1	tousled like kinase 1
MFN2	mitofusin 2
MED13	mediator complex subunit 13
CASP8AP2	caspase 8 associated protein 2
NR2E3	nuclear receptor subfamily 2 group E member 3
TANK	TRAF family member associated NFKB activator
MED16	mediator complex subunit 16
SH2D3C	SH2 domain containing 3C
PTPRU	protein tyrosine phosphatase, receptor type U
OPTN	optineurin
NAMPT	nicotinamide phosphoribosyltransferase
ARFRP1	ADP ribosylation factor related protein 1
G3BP1	G3BP stress granule assembly factor 1
WASF2	WAS protein family member 2
TNK2	tyrosine kinase non receptor 2
KLRG1	killer cell lectin like receptor G1
CNKSR1	connector enhancer of kinase suppressor of Ras 1
RAMP1	receptor activity modifying protein 1
AKAP8	A-kinase anchoring protein 8
NET1	neuroepithelial cell transforming 1
RGS19	regulator of G-protein signaling 19
APC2	adenomatosis polyposis coli 2
PAK4	p21 (RAC1) activated kinase 4
NMUR1	neuromedin U receptor 1
TLR6	toll like receptor 6
NOD1	nucleotide binding oligomerization domain containing 1
DLC1	DLC1 Rho GTPase activating protein
TAB1	TGF-beta activated kinase 1/MAP3K7 binding protein 1
BAIAP2	BAI1 associated protein 2

MERTK	MER proto-oncogene, tyrosine kinase
CAP2	CAP, adenylate cyclase-associated protein, 2 (yeast)
STK25	serine/threonine kinase 25
SORBS1	sorbin and SH3 domain containing 1
CDC42EP3	CDC42 effector protein 3
SH2B2	SH2B adaptor protein 2
TBL3	transducin beta like 3
RGS14	regulator of G-protein signaling 14
CAMKK2	calcium/calmodulin-dependent protein kinase kinase 2
RRAGA	Ras related GTP binding A
KLRA1P	killer cell lectin like receptor A1, pseudogene
CHL1	cell adhesion molecule L1 like
TRAF3IP2	TRAF3 interacting protein 2
PLK2	polo like kinase 2
NEK6	NIMA related kinase 6
IQGAP2	IQ motif containing GTPase activating protein 2
FRS3	fibroblast growth factor receptor substrate 3
FRS2	fibroblast growth factor receptor substrate 2
NPFFR2	neuropeptide FF receptor 2
RUNDC3A	RUN domain containing 3A
BRD8	bromodomain containing 8
LILRB3	leukocyte immunoglobulin like receptor B3
ADAP1	ArfGAP with dual PH domains 1
RAPGEF4	Rap guanine nucleotide exchange factor 4
PRDM4	PR domain 4
PTPRT	protein tyrosine phosphatase, receptor type T
FAF1	Fas associated factor 1
CHEK2	checkpoint kinase 2
AKAP11	A-kinase anchoring protein 11
AKAP10	A-kinase anchoring protein 10
DUSP10	dual specificity phosphatase 10
GPR176	G protein-coupled receptor 176
GPR45	G protein-coupled receptor 45
PARK7	Parkinsonism associated deglycase
STK38	serine/threonine kinase 38
MRAS	muscle RAS oncogene homolog
CARD8	caspase recruitment domain family member 8
KIFAP3	kinesin associated protein 3
ATF6	activating transcription factor 6
SCAP	SREBF chaperone
ACIN1	apoptotic chromatin condensation inducer 1
TNIK	TRAF2 and NCK interacting kinase
ERC1	ELKS/RAB6-interacting/CAST family member 1
PEG10	paternally expressed 10
TAB2	TGF-beta activated kinase 1/MAP3K7 binding protein 2
MAPK8IP3	mitogen-activated protein kinase 8 interacting protein 3
ITGB3BP	integrin subunit beta 3 binding protein
TNFRSF13B	tumor necrosis factor receptor superfamily member 13B
MAPK8IP2	mitogen-activated protein kinase 8 interacting protein 2
RBFOX2	RNA binding protein, fox-1 homolog 2
DDAH2	dimethylarginine dimethylaminohydrolase 2
ARL2BP	ADP ribosylation factor like GTPase 2 binding protein
DDAH1	dimethylarginine dimethylaminohydrolase 1
CBLC	Cbl proto-oncogene C
PRKD3	protein kinase D3
FKBP8	FK506 binding protein 8
CBY1	chibby homolog 1 (Drosophila)
PITPNC1	phosphatidylinositol transfer protein, cytoplasmic 1

FGF20	fibroblast growth factor 20
LATS2	large tumor suppressor kinase 2
RPS6KC1	ribosomal protein S6 kinase C1
ATP2C1	ATPase secretory pathway Ca2+ transporting 1
PDE7B	phosphodiesterase 7B
CIDEB	cell death-inducing DFFA-like effector b
IL17C	interleukin 17C
OXGR1	oxoglutarate receptor 1
CECR2	CECR2, histone acetyl-lysine reader
DBNL	drebrin like
HIPK2	homeodomain interacting protein kinase 2
TBK1	TANK binding kinase 1
CARD10	caspase recruitment domain family member 10
PKN3	protein kinase N3
TRHDE	thyrotropin releasing hormone degrading enzyme
MDFIC	MyoD family inhibitor domain containing
PILRA	paired immunoglobin like type 2 receptor alpha
KCNIP3	potassium voltage-gated channel interacting protein 3
KCNIP2	potassium voltage-gated channel interacting protein 2
KCNIP1	potassium voltage-gated channel interacting protein 1
PDE11A	phosphodiesterase 11A
SOST	sclerostin
NDUFA13	NADH:ubiquinone oxidoreductase subunit A13
APH1A	aph-1 homolog A, gamma-secretase subunit
RXFP3	relaxin/insulin like family peptide receptor 3
GMIP	GEM interacting protein
TAOK3	TAO kinase 3
UBR5	ubiquitin protein ligase E3 component n-recognin 5
CABP2	calcium binding protein 2
TRIAP1	TP53 regulated inhibitor of apoptosis 1
CXXC5	CXXC finger protein 5
STYXL1	serine/threonine/tyrosine interacting-like 1
NLK	nemo like kinase
ZAK	sterile alpha motif and leucine zipper containing kinase AZK
CALML5	calmodulin like 5
PRKAG3	protein kinase AMP-activated non-catalytic subunit gamma 3
DUOX1	dual oxidase 1
CYCS	cytochrome c, somatic
GPR85	G protein-coupled receptor 85
ZDHHC13	zinc finger DHHC-type containing 13
DLL4	delta like canonical Notch ligand 4
GTPBP2	GTP binding protein 2
ARHGEF10L	Rho guanine nucleotide exchange factor 10 like
MAML3	mastermind like transcriptional coactivator 3
CDC42BPG	CDC42 binding protein kinase gamma
CDKN2AIP	CDKN2A interacting protein
GNG12	G protein subunit gamma 12
PARD3	par-3 family cell polarity regulator
LTB4R2	leukotriene B4 receptor 2
CDC42SE1	CDC42 small effector 1
NMUR2	neuromedin U receptor 2
DUSP22	dual specificity phosphatase 22
OTUD7B	OTU deubiquitinase 7B
CDC42SE2	CDC42 small effector 2
CTNNBIP1	catenin beta interacting protein 1
SLC44A2	solute carrier family 44 member 2
SMURF1	SMAD specific E3 ubiquitin protein ligase 1
PLEKHG5	pleckstrin homology and RhoGEF domain containing G5

CASKIN1	CASK interacting protein 1
CALCOCO1	calcium binding and coiled-coil domain 1
MIER1	MIER1 transcriptional regulator
NLRC4	NLR family, CARD domain containing 4
SNX6	sorting nexin 6
SIGIRR	single immunoglobulin and toll-interleukin 1 receptor (TIR) domain
PROK2	prokineticin 2
MOAP1	modulator of apoptosis 1
RRAGC	Ras related GTP binding C
NOD2	nucleotide binding oligomerization domain containing 2
CARD9	caspase recruitment domain family member 9
ARAP3	ArfGAP with RhoGAP domain, ankyrin repeat and PH domain 3
CLEC7A	C-type lectin domain family 7 member A
WNK1	WNK lysine deficient protein kinase 1
WNK4	WNK lysine deficient protein kinase 4
WNK2	WNK lysine deficient protein kinase 2
WLS	wntless Wnt ligand secretion mediator
PREX2	phosphatidylinositol-3,4,5-trisphosphate dependent Rac exchange factor 2
NDFIP1	Nedd4 family interacting protein 1
DUSP16	dual specificity phosphatase 16
APOL3	apolipoprotein L3
VOPP1	vesicular, overexpressed in cancer, prosurvival protein 1
CCM2	CCM2 scaffolding protein
TRAF7	TNF receptor associated factor 7
MAML2	mastermind like transcriptional coactivator 2
BRSK1	BR serine/threonine kinase 1
KIAA1804	mixed lineage kinase 4
AFAP1L2	actin filament associated protein 1 like 2
NLRP12	NLR family, pyrin domain containing 12
RASGRP4	RAS guanyl releasing protein 4
ARAP1	ArfGAP with RhoGAP domain, ankyrin repeat and PH domain 1
CANT1	calcium activated nucleotidase 1
EDARADD	EDAR-associated death domain
MIB2	mindbomb E3 ubiquitin protein ligase 2
SIK1	salt inducible kinase 1
NFAM1	NFAT activating protein with ITAM motif 1
ROPN1B	rhophilin associated tail protein 1B
FGD5	FYVE, RhoGEF and PH domain containing 5
SPRED1	sprouty related EVH1 domain containing 1
DEDD2	death effector domain containing 2
EID2	EP300 interacting inhibitor of differentiation 2
NLRC3	NLR family, CARD domain containing 3
SPRED2	sprouty related EVH1 domain containing 2
ARHGAP27	Rho GTPase activating protein 27
FGD2	FYVE, RhoGEF and PH domain containing 2
RICTOR	RPTOR independent companion of MTOR complex 2
EPGN	epithelial mitogen
CRIPAK	cysteine rich PAK1 inhibitor
VWC2	von Willebrand factor C domain containing 2
AGRN	agrin
CCR2	C-C motif chemokine receptor 2
**Generation of neurons**	
**Gene Symbol**	**Name Gene**
ATP2B2	ATPase plasma membrane Ca2+ transporting 2
DTX1	deltex 1
GDNF	glial cell derived neurotrophic factor
GLI2	GLI family zinc finger 2
MAPT	microtubule associated protein tau

NRCAM	neuronal cell adhesion molecule
NRTN	neurturin
PAX2	paired box 2
SERPINF1	serpin family F member 1
ROBO1	roundabout guidance receptor 1
ROBO2	roundabout guidance receptor 2
RTN1	reticulon 1
SHH	sonic hedgehog
UBB	ubiquitin B
YWHAG	tyrosine 3-monooxygenase/tryptophan 5-monooxygenase activation protein gamma
SEMA3B	semaphorin 3B
UNC5C	unc-5 netrin receptor C
NRP2	neuropilin 2
NRP1	neuropilin 1
LDB1	LIM domain binding 1
BRSK2	BR serine/threonine kinase 2
ARTN	artemin
NRXN3	neurexin 3
NRXN1	neurexin 1
FEZ2	fasciculation and elongation protein zeta 2
FARP2	FERM, ARH/RhoGEF and pleckstrin domain protein 2
SPON2	spondin 2
BAIAP2	BAI1 associated protein 2
SEMA4F	ssemaphorin 4F
CIT	citron rho-interacting serine/threonine kinase
NTNG1	netrin G1
NLGN1	neuroligin 1
CYFIP1	cytoplasmic FMR1 interacting protein 1
NPTN	neuroplastin
RND1	Rho family GTPase 1
MAP1S	microtubule associated protein 1S
PARD3	par-3 family cell polarity regulator
RTN4	reticulon 4
LRRC4C	leucine rich repeat containing 4C
NTNG2	netrin G2
RTN4RL1	reticulon 4 receptor like 1
CNTN4	contactin 4
MDGA1	MAM domain containing glycosylphosphatidylinositol anchor 1
AGRN	agrin
**Neurogenesis**	
**Gene Symbol**	**Name Gene**
ATP2B2	ATPase plasma membrane Ca2+ transporting 2
CLN5	ceroid-lipofuscinosis, neuronal 5
DTX1	deltex 1
GDNF	glial cell derived neurotrophic factor
GLI2	GLI family zinc finger 2
MAPT	microtubule associated protein tau
NRCAM	neuronal cell adhesion molecule
NRTN	neurturin
PAX2	paired box 2
SERPINF1	serpin family F member 1
ROBO1	roundabout guidance receptor 1
ROBO2	roundabout guidance receptor 2
RTN1	reticulon 1
SHH	sonic hedgehog
SOD1	superoxide dismutase 1, soluble
UBB	ubiquitin B

YWHAG	tyrosine 3-monooxygenase/tryptophan 5-monooxygenase activation protein gamma
SEMA3B	semaphorin 3B
UNC5C	unc-5 netrin receptor C
NRP2	neuropilin 2
NRP1	neuropilin 1
LDB1	LIM domain binding 1
EIF2B3	eukaryotic translation initiation factor 2B subunit gamma
BRSK2	BR serine/threonine kinase 2
ARTN	artemin
NRXN3	neurexin 3
NRXN1	neurexin 1
FEZ2	fasciculation and elongation protein zeta 2
FARP2	FERM, ARH/RhoGEF and pleckstrin domain protein 2
SPON2	spondin 2
BAIAP2	BAI1 associated protein 2
SEMA4F	ssemaphorin 4F
CIT	citron rho-interacting serine/threonine kinase
NTNG1	netrin G1
NLGN1	neuroligin 1
CYFIP1	cytoplasmic FMR1 interacting protein 1
NPTN	neuroplastin
RND1	Rho family GTPase 1
MAP1S	microtubule associated protein 1S
PARD3	par-3 family cell polarity regulator
RTN4	reticulon 4
LRRC4C	leucine rich repeat containing 4C
NTNG2	netrin G2
RTN4RL1	reticulon 4 receptor like 1
CNTN4	contactin 4
MDGA1	MAM domain containing glycosylphosphatidylinositol anchor 1
AGRN	agrin
**Neuron diferentiation**	
**Gene Symbol**	**Name Gene**
ATP2B2	ATPase plasma membrane Ca2+ transporting 2
DTX1	deltex 1
GDNF	glial cell derived neurotrophic factor
GLI2	GLI family zinc finger 2
MAPT	microtubule associated protein tau
NRCAM	neuronal cell adhesion molecule
NRTN	neurturin
PAX2	paired box 2
ROBO1	roundabout guidance receptor 1
ROBO2	roundabout guidance receptor 2
RTN1	reticulon 1
SHH	sonic hedgehog
UBB	ubiquitin B
YWHAG	tyrosine 3-monooxygenase/tryptophan 5-monooxygenase activation protein gamma
SEMA3B	semaphorin 3B
UNC5C	unc-5 netrin receptor C
NRP2	neuropilin 2
NRP1	neuropilin 1
LDB1	LIM domain binding 1
BRSK2	BR serine/threonine kinase 2
NRXN3	neurexin 3
NRXN1	neurexin 1
FEZ2	fasciculation and elongation protein zeta 2

FARP2	FERM, ARH/RhoGEF and pleckstrin domain protein 2
SPON2	spondin 2
BAIAP2	BAI1 associated protein 2
SEMA4F	ssemaphorin 4F
NTNG1	netrin G1
NLGN1	neuroligin 1
CYFIP1	cytoplasmic FMR1 interacting protein 1
RND1	Rho family GTPase 1
MAP1S	microtubule associated protein 1S
PARD3	par-3 family cell polarity regulator
RTN4	reticulon 4
LRRC4C	leucine rich repeat containing 4C
NTNG2	netrin G2
RTN4RL1	reticulon 4 receptor like 1
CNTN4	contactin 4
MDGA1	MAM domain containing glycosylphosphatidylinositol anchor 1
VWC2	von Willebrand factor C domain containing 2
AGRN	agrin
**Neuron development**	
**Gene Symbol**	**Name Gene**
GDNF	glial cell derived neurotrophic factor
GLI2	GLI family zinc finger 2
MAPT	microtubule associated protein tau
NRCAM	neuronal cell adhesion molecule
NRTN	neurturin
PAX2	paired box 2
ROBO1	roundabout guidance receptor 1
ROBO2	roundabout guidance receptor 2
SHH	sonic hedgehog
UBB	ubiquitin B
SEMA3B	semaphorin 3B
UNC5C	unc-5 netrin receptor C
NRP2	neuropilin 2
NRP1	neuropilin 1
NRXN3	neurexin 3
NRXN1	neurexin 1
FEZ2	fasciculation and elongation protein zeta 2
FARP2	FERM, ARH/RhoGEF and pleckstrin domain protein 2
SPON2	spondin 2
BAIAP2	BAI1 associated protein 2
SEMA4F	ssemaphorin 4F
NTNG1	netrin G1
CYFIP1	cytoplasmic FMR1 interacting protein 1
RND1	Rho family GTPase 1
MAP1S	microtubule associated protein 1S
PARD3	par-3 family cell polarity regulator
RTN4	reticulon 4
LRRC4C	leucine rich repeat containing 4C
NTNG2	netrin G2
RTN4RL1	reticulon 4 receptor like 1
CNTN4	contactin 4
AGRN	agrin
	glial cell derived neurotrophic factor
	GLI family zinc finger 2
GDNF	microtubule associated protein tau
GLI2	neuronal cell adhesion molecule
MAPT	neurturin
NRCAM	paired box 2

NRTN	roundabout guidance receptor 1
PAX2	roundabout guidance receptor 2
ROBO1	sonic hedgehog
ROBO2	ubiquitin B
SHH	semaphorin 3B
UBB	unc-5 netrin receptor C
SEMA3B	neuropilin 2
UNC5C	neuropilin 1
NRP2	neurexin 3
NRP1	neurexin 1
NRXN3	fasciculation and elongation protein zeta 2
NRXN1	FERM, ARH/RhoGEF and pleckstrin domain protein 2
FEZ2	spondin 2
FARP2	BAI1 associated protein 2
SPON2	ssemaphorin 4F
BAIAP2	netrin G1
SEMA4F	cytoplasmic FMR1 interacting protein 1
NTNG1	Rho family GTPase 1
CYFIP1	microtubule associated protein 1S
RND1	par-3 family cell polarity regulator
MAP1S	reticulon 4
PARD3	leucine rich repeat containing 4C
RTN4	netrin G2
LRRC4C	reticulon 4 receptor like 1
NTNG2	contactin 4
RTN4RL1	agrin
CNTN4	glial cell derived neurotrophic factor
AGRN	GLI family zinc finger 2
**Negative regulation of biological process**	
**Gene Symbol**	**Name Gene**
ACTN1	actinin alpha 1
ACVR1	activin A receptor type 1
ACVRL1	activin A receptor like type 1
ADRA1A	adrenoceptor alpha 1A
ADRB3	adrenoceptor beta 3
ALOX15B	arachidonate 15-lipoxygenase, type B
ANG	angiogenin
APBB2	amyloid beta precursor protein binding family B member 2
FAS	Fas cell surface death receptor
RHOB	ras homolog family member B
ARHGDIA	Rho GDP dissociation inhibitor alpha
BCL3	B-cell CLL/lymphoma 3
BCL6	B-cell CLL/lymphoma 6
PRDM1	PR domain 1
BMP7	bone morphogenetic protein 7
BNIP2	BCL2/adenovirus E1B 19kDa interacting protein 2
BNIP3	BCL2/adenovirus E1B 19kDa interacting protein 3
BNIP3L	BCL2/adenovirus E1B 19kDa interacting protein 3-like
BRCA1	breast cancer 1
BRAF	B-Raf proto-oncogene, serine/threonine kinase
BTG1	B-cell translocation gene 1, anti-proliferative
CASP3	caspase 3
RUNX2	runt related transcription factor 2
RUNX3	runt related transcription factor 3
CD28	CD28 molecule
TNFRSF8	tumor necrosis factor receptor superfamily member 8
CD74	CD74 molecule
CDK1	cyclin-dependent kinase 1

CDH13	cadherin 13
CDKN1A	cyclin-dependent kinase inhibitor 1A
CENPF	centromere protein F
CHEK1	checkpoint kinase 1
FOXN3	forkhead box N3
COL4A2	collagen type IV alpha 2
COL4A3	collagen type IV alpha 3
CRYAA	crystallin alpha A
CTBP1	C-terminal binding protein 1
CTBP2	C-terminal binding protein 2
CUX1	cut like homeobox 1
DAD1	defender against cell death 1
DAXX	death-domain associated protein
GADD45A	growth arrest and DNA damage inducible alpha
DHCR24	24-dehydrocholesterol reductase
DNMT1	DNA (cytosine-5-)-methyltransferase 1
DTX1	deltex 1
EIF4G2	eukaryotic translation initiation factor 4 gamma 2
ELANE	elastase, neutrophil expressed
ELK3	ELK3, ETS transcription factor
ENO1	enolase 1
ERCC4	excision repair cross-complementation group 4
ERN1	endoplasmic reticulum to nucleus signaling 1
ETS1	ETS proto-oncogene 1, transcription factor
F2	coagulation factor II, thrombin
FABP6	fatty acid binding protein 6
FOXC1	forkhead box C1
FOXO1	forkhead box O1
FRK	fyn related Src family tyrosine kinase
IFI6	interferon alpha inducible protein 6
GCK	glucokinase
NR6A1	nuclear receptor subfamily 6 group A member 1
GDNF	glial cell derived neurotrophic factor
GHSR	growth hormone secretagogue receptor
GCLC	glutamate-cysteine ligase catalytic subunit
GLI2	GLI family zinc finger 2
GLRA1	glycine receptor alpha 1
GML	glycosylphosphatidylinositol anchored molecule like
ARHGAP35	Rho GTPase activating protein 35
GRM8	glutamate metabotropic receptor 8
NRG1	neuregulin 1
HMGB1	high mobility group box 1
IRF8	interferon regulatory factor 8
ID2	inhibitor of DNA binding 2, HLH protein
ID3	inhibitor of DNA binding 3, HLH protein
ID4	inhibitor of DNA binding 4, HLH protein
IGF1R	insulin like growth factor 1 receptor
IGFBP3	insulin like growth factor binding protein 3
IGFBP7	insulin like growth factor binding protein 7
RBPJ	recombination signal binding protein for immunoglobulin kappa J region
IL1B	interleukin 1 beta
IL6	interleukin 6
IL10	interleukin 10
TNFRSF9	tumor necrosis factor receptor superfamily member 9
ING1	inhibitor of growth family member 1
INHBA	inhibin beta A
INHBB	inhibin beta B
IRF2	interferon regulatory factor 2

IRF7	interferon regulatory factor 7
KRT18	keratin 18
LEP	leptin
SMAD3	SMAD family member 3
MCL1	myeloid cell leukemia 1
MDFI	MyoD family inhibitor
MEIS2	Meis homeobox 2
MEN1	menin 1
MNT	MAX network transcriptional repressor
MXI1	MAX interactor 1, dimerization protein
MYC	v-myc avian myelocytomatosis viral oncogene homolog
NAIP	NLR family, apoptosis inhibitory protein
NDUFS3	NADH:ubiquinone oxidoreductase core subunit S3
NFKBIE	NFKB inhibitor epsilon
NFKBIL1	NFKB inhibitor like 1
TONSL	tonsoku-like, DNA repair protein
NOTCH1	notch 1
NOTCH2	notch 2
NOTCH4	notch 4
NPM1	nucleophosmin (nucleolar phosphoprotein B23, numatrin)
NPPB	natriuretic peptide B
NPR1	natriuretic peptide receptor 1
OPA1	OPA1, mitochondrial dynamin like GTPase
OPRM1	opioid receptor mu 1
OSM	oncostatin M
SERPINE1	serpin family E member 1
PAWR	pro-apoptotic WT1 regulator
PAX7	paired box 7
CHMP1A	charged multivesicular body protein 1A
SERPINF1	serpin family F member 1
PEX14	peroxisomal biogenesis factor 14
PF4	platelet factor 4
ATP8B1	ATPase phospholipid transporting 8B1
SERPINB9	serpin family B member 9
PKHD1	polycystic kidney and hepatic disease 1 (autosomal recessive)
PLAGL1	PLAG1 like zinc finger 1
PLG	plasminogen
PML	promyelocytic leukemia
POU2F1	POU class 2 homeobox 1
PPARD	peroxisome proliferator activated receptor delta
PRKCZ	protein kinase C zeta
MAP2K6	mitogen-activated protein kinase kinase 6
EIF2AK2	eukaryotic translation initiation factor 2 alpha kinase 2
THAP12	THAP domain containing 12
PROC	protein C, inactivator of coagulation factors Va and VIIIa
PSEN1	presenilin 1
PTCH1	patched 1
RAD9A	RAD9 checkpoint clamp component A
RAD17	RAD17 checkpoint clamp loader component
RARRES1	retinoic acid receptor responder 1
RB1	retinoblastoma 1
RBBP4	retinoblastoma binding protein 4
TRIM27	tripartite motif containing 27
RTKN	rhotekin
S100A11	S100 calcium binding protein A11
ATXN1	ataxin 1
CCL2	C-C motif chemokine ligand 2
SFRP1	secreted frizzled related protein 1

SFTPD	surfactant protein D
SHH	sonic hedgehog
SNAI2	snail family transcriptional repressor 2
SMARCC2	SWI/SNF related, matrix associated, actin dependent regulator of chromatin subfamily c member 2
SMARCE1	SWI/SNF related, matrix associated, actin dependent regulator of chromatin, subfamily e, member 1
SNCA	synuclein alpha
SOD1	superoxide dismutase 1, soluble
SSTR5	somatostatin receptor 5
STAT3	signal transducer and activator of transcription 3
STK11	serine/threonine kinase 11
TBX2	T-box 2
TBX5	T-box 5
TBX3	T-box 3
VPS72	vacuolar protein sorting 72 homolog
PRDX2	peroxiredoxin 2
TGFB1I1	transforming growth factor beta 1 induced transcript 1
KLF10	Kruppel-like factor 10
TLE1	transducin like enhancer of split 1
TM4SF4	transmembrane 4 L six family member 4
TNF	tumor necrosis factor
TNFAIP3	TNF alpha induced protein 3
TNNI3	troponin I3, cardiac type
TNP1	transition protein 1
TWIST1	twist family bHLH transcription factor 1
NR1H2	nuclear receptor subfamily 1 group H member 2
VEGFA	vascular endothelial growth factor A
VHL	von Hippel-Lindau tumor suppressor
WARS	tryptophanyl-tRNA synthetase
YWHAH	tyrosine 3-monooxygenase/tryptophan 5-monooxygenase activation protein eta
YWHAZ	tyrosine 3-monooxygenase/tryptophan 5-monooxygenase activation protein zeta
ZBTB16	zinc finger and BTB domain containing 16
ZBTB17	zinc finger and BTB domain containing 17
ZNF189	zinc finger protein 189
BTG2	BTG family member 2
SCG2	secretogranin II
CUL5	cullin 5
BAP1	BRCA1 associated protein 1
CDC45	cell division cycle 45
NCK2	NCK adaptor protein 2
CUL4A	cullin 4A
CUL2	cullin 2
CUL1	cullin 1
PPM1D	protein phosphatase, Mg2+/Mn2+ dependent 1D
IFITM1	interferon induced transmembrane protein 1
CBX4	chromobox 4
TP63	tumor protein p63
HRK	harakiri, BCL2 interacting protein
CD164	CD164 molecule
TNFRSF18	tumor necrosis factor receptor superfamily member 18
CFLAR	CASP8 and FADD like apoptosis regulator
NR1I2	nuclear receptor subfamily 1 group I member 2
LDB1	LIM domain binding 1
IER3	immediate early response 3
SPHK1	sphingosine kinase 1

TAX1BP1	Tax1 binding protein 1
SOCS3	suppressor of cytokine signaling 3
HGS	hepatocyte growth factor-regulated tyrosine kinase substrate
ARHGEF2	Rho/Rac guanine nucleotide exchange factor 2
DEDD	death effector domain containing
DLG5	discs large homolog 5
AIMP1	aminoacyl tRNA synthetase complex interacting multifunctional protein 1
KLF4	Kruppel-like factor 4 (gut)
EIF2AK3	eukaryotic translation initiation factor 2 alpha kinase 3
ADAMTS1	ADAM metallopeptidase with thrombospondin type 1 motif 1
ZNF254	zinc finger protein 254
TP53I11	tumor protein p53 inducible protein 11
BRE	brain and reproductive organ-expressed (TNFRSF1A modulator)
CARTPT	CART prepropeptide
SOCS5	suppressor of cytokine signaling 5
HDAC9	histone deacetylase 9
HDAC4	histone deacetylase 4
SERTAD2	SERTA domain containing 2
MFN2	mitofusin 2
HDAC5	histone deacetylase 5
HIPK3	homeodomain interacting protein kinase 3
YAF2	YY1 associated factor 2
TOB1	transducer of ERBB2, 1
DHRS2	dehydrogenase/reductase (SDR family) member 2
SPEG	SPEG complex locus
KATNB1	katanin regulatory subunit B1
DLC1	DLC1 Rho GTPase activating protein
GPNMB	glycoprotein nmb
FST	follistatin
ERN2	endoplasmic reticulum to nucleus signaling 2
MXD4	MAX dimerization protein 4
CTCF	CCCTC-binding factor
ZMYND11	zinc finger MYND-type containing 11
SRSF10	serine/arginine-rich splicing factor 10
ARID5A	AT-rich interaction domain 5A
BTG3	BTG family member 3
WWP1	WW domain containing E3 ubiquitin protein ligase 1
FAF1	Fas associated factor 1
KLF12	Kruppel-like factor 12
B4GALT7	beta-1,4-galactosyltransferase 7
ZHX2	zinc fingers and homeoboxes 2
CARD8	caspase recruitment domain family member 8
MAPRE1	microtubule associated protein RP/EB family member 1
MYO16	myosin XVI
ERC1	ELKS/RAB6-interacting/CAST family member 1
PEG10	paternally expressed 10
ZNF423	zinc finger protein 423
CLASP1	cytoplasmic linker associated protein 1
PPP1R13B	protein phosphatase 1 regulatory subunit 13B
SIRT4	sirtuin 4
RYBP	RING1 and YY1 binding protein
ZNF281	zinc finger protein 281
RBFOX2	RNA binding protein, fox-1 homolog 2
GTPBP4	GTP binding protein 4
DDAH2	dimethylarginine dimethylaminohydrolase 2
CBLC	Cbl proto-oncogene C
PPP1R15A	protein phosphatase 1 regulatory subunit 15A
CBY1	chibby homolog 1 (Drosophila)

TNFAIP8	TNF alpha induced protein 8
BACE2	beta-site APP-cleaving enzyme 2
CLIC4	chloride intracellular channel 4
LATS2	large tumor suppressor kinase 2
AATF	apoptosis antagonizing transcription factor
EIF2AK1	eukaryotic translation initiation factor 2 alpha kinase 1
RND1	Rho family GTPase 1
UTP20	UTP20, small subunit processome component
PACSIN3	protein kinase C and casein kinase substrate in neurons 3
SERTAD3	SERTA domain containing 3
OSGIN1	oxidative stress induced growth inhibitor 1
NOX4	NADPH oxidase 4
SOST	sclerostin
SH3GLB1	SH3 domain containing GRB2 like endophilin B1
ING4	inhibitor of growth family member 4
BFAR	bifunctional apoptosis regulator
PHF21A	PHD finger protein 21A
TAOK3	TAO kinase 3
IP6K2	inositol hexakisphosphate kinase 2
TRIAP1	TP53 regulated inhibitor of apoptosis 1
PIAS4	protein inhibitor of activated STAT 4
NLK	nemo like kinase
ATP8A2	ATPase phospholipid transporting 8A2
ZAK	sterile alpha motif and leucine zipper containing kinase AZK
GATAD2A	GATA zinc finger domain containing 2A
CDHR2	cadherin related family member 2
CDKN2AIP	CDKN2A interacting protein
ATF7IP	activating transcription factor 7 interacting protein
LANCL2	LanC like 2
RPRM	reprimo, TP53 dependent G2 arrest mediator candidate
OTUD7B	OTU deubiquitinase 7B
RTN4	reticulon 4
SMURF1	SMAD specific E3 ubiquitin protein ligase 1
STIM2	stromal interaction molecule 2
PHF12	PHD finger protein 12
CREBZF	CREB/ATF bZIP transcription factor
SNX6	sorting nexin 6
SIGIRR	single immunoglobulin and toll-interleukin 1 receptor (TIR) domain
PROK2	prokineticin 2
DNAJC1	DnaJ heat shock protein family (Hsp40) member C1
NSD1	nuclear receptor binding SET domain protein 1
SUDS3	SDS3 homolog, SIN3A corepressor complex component
COL18A1	collagen type XVIII alpha 1
CDT1	chromatin licensing and DNA replication factor 1
ARID5B	AT-rich interaction domain 5B
ING5	inhibitor of growth family member 5
AKT1S1	AKT1 substrate 1
GLIS2	GLIS family zinc finger 2
SCIN	scinderin
NLRP12	NLR family, pyrin domain containing 12
ATPIF1	ATPase inhibitory factor 1
ARAP1	ArfGAP with RhoGAP domain, ankyrin repeat and PH domain 1
TWIST2	twist family bHLH transcription factor 2
B4GALNT2	beta-1,4-N-acetyl-galactosaminyltransferase 2
DMBX1	diencephalon/mesencephalon homeobox 1
UHMK1	U2AF homology motif (UHM) kinase 1
FAM3D	family with sequence similarity 3 member D
GLIS1	GLIS family zinc finger 1

CNTN4	contactin 4
EID2	EP300 interacting inhibitor of differentiation 2
TMPRSS6	transmembrane protease, serine 6
GLIS3	GLIS family zinc finger 3
HS3ST5	heparan sulfate-glucosamine 3-sulfotransferase 5
VWC2	von Willebrand factor C domain containing 2

Table S5. Descriptive data in the groups defined by trajectories of anxiety disorders

Table S5. Descriptive data in the groups defined by trajectories of anxiety disorders

Variables	TDC Group n=14	P-value	Persistent Group n=14	P-value	Incident Group n=11	P-value	Remittent Group n=8	P-value	P-value
Gender (female, %)	08 (57.14%)	.724[a]	08 (57.14%)	.724[a]	05 (45.45%)	.557[a]	04 (50%)	.843[a]	[b] 1.000; [c] .561; [d] .746; [e] .561 [f] .746 and [g] .845
Age in 2013 (mean ± SD)	17.96 ± 2.38		18.32 ± 2.34		17.27 ± 1.95		19.63 ± 2.71		[h] .950; [i] .531; [j] .758; [k] .568; [l] .716 and [m] .389
Ethnicity									
Caucasians	06 (50%)	.135	10 (71.43%)	.541	08 (72.72%)	.371	03 (37.5%)	.150	[n] .324; [o] .248; [p] .492; [q] .646; [r] .165 and [s] .077
Brazilian Africans	02 (16.66%)		01 (7.14%)		0 (0%)		03 (37.5%)		
Others	04 (33.33%)		03 (21.43%)		03 (27.27%)		02 (25%)		

[a] Chi-square test. Differences between male and females inside each group.
[b, c, d, e, f, g] Chi-square test. Comparisons between male and female in the four groups. ([b] TDC *vs* Persistent; [c] TDC *vs* Incident; [d] TDC *vs* Remittent; [e] Persistent *vs* Incident, [f] Persistent *vs* Remittent and [g] Incident *vs* Remittent).
[h, i, j, k, l, m] P-values from Student's t-test. Comparisons between ages in the four groups. ([h] TDC *vs* Persistent; [i] TDC *vs* Incident; [j] TDC *vs* Remittent; [k] Persistent *vs* Incident; [l] Persistent *vs* Remittent and [m] Incident *vs* Remittent).
[n, o, p, q, r, s] P-values from Chi-squared test. Comparisons between ethnicity in the four groups. ([n] TDC *vs* Persistent; [o] TDC *vs* Incident; [p] TDC *vs* Remittent; [q] Persistent *vs* Incident; [r] Persistent *vs* Remittent and [s] Incident *vs* Remittent).
Statistical significance: $P < 0.05$

Table S6. Descriptive data about adolescents evaluated in 2008 (n= 234)

Table S6. Descriptive data about adolescents evaluated in 2008 (n= 234)

	**Participants of Genome-wide methylation (n = 47)**			**Non-study participants (n = 187)**		
**Variables**	Anxiety Individuals (n=22)	Non-Anxious (n=25)	P-value	Anxiety Individuals (n=109)	Non-Anxious (n=78)	P-value
[a] Gender (female)	12 (54.5%)	13 (52%)	.861	84 (77.06%)	46 (58.97%)	.008
[b] Age (mean ± SD)[b]	13.79 ± 2.5	12.66 ± 2.18	.506	12.72 ± 4.06	11.98 ± 4.67	.442
[c] **Ethnicity**						
Caucasians	13 (59.1%)	14 (58.3%)	.481	69 (63.30%)	54 (69.23%)	.310
Brazilian Africans	04 (18.2%)	02 (8.3%)		16 (14.68%)	08 (10.26%)	
Others	05 22.7%)	07 (29.2%)		18 (16.51%)	08 (10.26%)	

[a] Chi-squared. Differences between anxiety individuals and non-anxious considering gender.
[b] Test t student. Differences between anxiety individual and non-anxious considering age.
[c] Chi-squared. Differences between anxiety individuals and non-anxious considering ethnicity. Statistical significance: $P< 0.05$.
